# Evidence of a large current of transcranial alternating current stimulation directly to deep brain regions

**DOI:** 10.1038/s41380-023-02150-8

**Published:** 2023-07-19

**Authors:** Yongzhi Shan, Hongxing Wang, Yanfeng Yang, Jiahao Wang, Wenfeng Zhao, Yuda Huang, Huang Wang, Bing Han, Na Pan, Xiukun Jin, Xiaotong Fan, Yunyun Liu, Jun Wang, Changming Wang, Huaqiang Zhang, Sichang Chen, Ting Liu, Tianyi Yan, Tianmei Si, Lu Yin, Xinmin Li, Fiammetta Cosci, Xiangyang Zhang, Guanghao Zhang, Keming Gao, Guoguang Zhao

**Affiliations:** 1 Department of Neurosurgery, Xuanwu Hospital, National Center for Neurological Disorders, National Clinical Research Center for Geriatric Diseases, Capital Medical University, Beijing, 100053 China; 2 China International Neuroscience Institute (CHINA-INI), Beijing, 100053 China; 3 Beijing Municipal Geriatric Medical Research Center, Beijing, 100053 China; 4 Division of Neuropsychiatry and Psychosomatics, Department of Neurology, Xuanwu Hospital, National Center for Neurological Disorders, National Clinical Research Center for Geriatric Diseases, Capital Medical University, Beijing, 100053 China; 5 Beijing Institute of Brain Disorders, Beijing, 100069 China; 6 Beijing Key Laboratory of Bioelectromagnetism, Institute of Electrical Engineering, Chinese Academy of Sciences, Beijing, 100190 China; 7 School of Electronic, Electrical and Communication Engineering, University of Chinese Academy of Sciences, Beijing, 100049 China; 8 School of Life Science, Beijing Institute of Technology, Beijing, 100081 China; 9 Peking University Sixth Hospital, Peking University Institute of Mental Health, National Clinical Research Center for Mental Disorders, Beijing, 100191 China; 10 Medical Research & Biometrics Centre, Fuwai Hospital, National Centre for Cardiovascular Diseases, Peking Union Medical College & Chinese Academy of Medical Sciences, Beijing, 102300 China; 11 Department of Psychiatry, Faculty of Medicine and Dentistry, University of Alberta, Edmonton, Albert, T6G 2B7 Canada; 12 Department of Health Sciences, University of Florence, Florence, 50135 Italy; 13 CAS Key Laboratory of Mental Health, Chinese Academy of Sciences, Beijing, 100101 China; 14 Department of Psychiatry, University Hospitals Cleveland Medical Center, Cleveland, Ohio, USA; Case Western Reserve University School of Medicine, Cleveland, OH 44106 USA; 15 Center of Epilepsy, Beijing Institute of Brain Disorders, Beijing, 100069 China

**Keywords:** Neuroscience, Biological techniques

## Abstract

Deep brain regions such as hippocampus, insula, and amygdala are involved in neuropsychiatric disorders, including chronic insomnia and depression. Our recent reports showed that transcranial alternating current stimulation (tACS) with a current of 15 mA and a frequency of 77.5 Hz, delivered through a montage of the forehead and both mastoids was safe and effective in intervening chronic insomnia and depression over 8 weeks. However, there is no physical evidence to support whether a large alternating current of 15 mA in tACS can send electrical currents to deep brain tissue in awake humans. Here, we directly recorded local field potentials (LFPs) in the hippocampus, insula and amygdala at different current strengths (1 to 15 mA) in 11 adult patients with drug-resistant epilepsy implanted with stereoelectroencephalography (SEEG) electrodes who received tACS at 77.5 Hz from 1 mA to 15 mA at 77.5 Hz for five minutes at each current for a total of 40 min. For the current of 15 mA at 77.5 Hz, additional 55 min were applied to add up a total of 60 min. Linear regression analysis revealed that the average LFPs for the remaining contacts on both sides of the hippocampus, insula, and amygdala of each patient were statistically associated with the given currents in each patient (*p* < 0.05–0.01), except for the left insula of one subject (*p* = 0.053). Alternating currents greater than 7 mA were required to produce significant differences in LFPs in the three brain regions compared to LFPs at 0 mA (*p* < 0.05). The differences remained significant after adjusting for multiple comparisons (*p* < 0.05). Our study provides direct evidence that the specific tACS procedures are capable of delivering electrical currents to deep brain tissues, opening a realistic avenue for modulating or treating neuropsychiatric disorders associated with hippocampus, insula, and amygdala.

## Introduction

Deep brain structures such as the hippocampus [1, 2], insula [3, 4], and amygdala [5, 6] are believed to underlie important neuropsychiatric disorders, such as depression and chronic insomnia. Transcranial alternating current stimulation (tACS) is a method of cranial electrotherapy stimulation, which provides brain stimulation by applying changing intensity electrical currents to the scalp to regulate cortical excitability and spontaneous brain activity [7–14]. Recenly, our studies showed the tACS with a current of 15 mA and a frequency of 77.5 Hz delivered through a montage of the forehead and both mastoids was safe and effective in interventing chronic insomnia [15] and depression [14, 16] over 8 weeks. However, other interventions using tACS currents less than 4 mA for depression and insomnia showed inconsistent results [14, 15, 17–20]. The stimulation currents less than 4 mA tend to locally stimulate superficial areas such as the cerebral cortex [17–21], and whether the 15 mA of tACS, as a large alternating current, can directly stimulate the deep brain area in awake human is lack of evidence.

The hippocampus [2], insula [4], and amygdala [6] are also thought to be frequently associated with drug-resistant epilepsy (DRE) [22, 23], and surgical resection and/or deep brain stimulation (DBS) are viable treatments for some patients with DRE [24]. For surgical resection and/or DBS planning, stereoelectroencephalography (SEEG) is essential to localize epileptogenic regions in DRE patients [25]. Also, SEEG offers a unique advantage in directly and precisely detecting electrical activity and identifying changes in local field potentials (LFPs) in deep brain structures involved in epilepsy [25, 26].

This small group of patients with DRE who are also indicated for SEEG provides a rare and unique opportunity to study the effect of pharmacological and non-pharmacological interventions on deep brain tissues. Electrical current stimulation, especially transcranial current stimulation, is less invasive, safe, and easy to manipulate. tACS is one form of the transcranial current stimulations. Therefore, it is reasonable to investigate the ability of tACS current delivery to deep brain tissues by using SEEG in DRE patients. In doing so, we may undertand where the tACS currents can reach during tACS treatment.

In this context, we hypothesized that the current of tACS in our previous reports [14, 15] may deliver electrical currents directly to deep brain regions, including the hippocampus, insula, and amygdala. Therefore, we used SEEG in awake DRE patients to examine whether there were any changes in LFPs in the hippocampus, insula, and amygdala during tACS at different currents, and explored the associations between them, providing direct evidence whether tACS can send electrical currents to deep brain regions.

## Materials and methods

### Study design

**Fig. 1:**
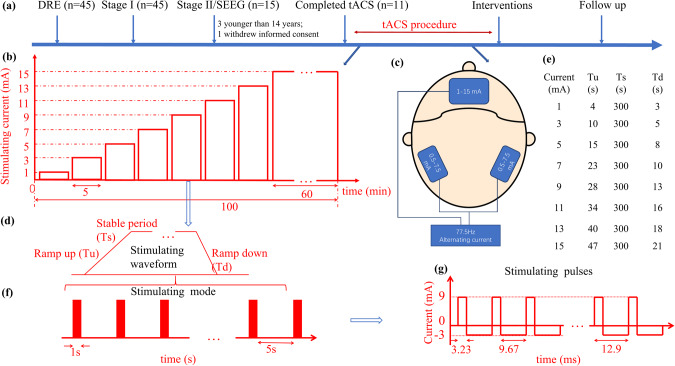
Study procedure overview. **a** Summary of study flow. 45 patients with drug-resistant epilepsy (DRE) were verified and entered the comprehensive evaluation of their treatments. Of them, 11 patients with DRE signed the informed content and experienced the tACS procedure, then received the corresponding interventions and followed up. **b** Structure of the tACS protocol. Each patient was presented with a set of alternating currents, beginning at 1 mA and stepwise increasing by 2 mA until reaching 15 mA. 15 mA persisted for 60 min. **c** Stimulating placements. An electrode was put on the forehead, and two electrodes were placed on the mastoid region of each side. **d** Sample of 9 mA stimulating waveform. Each stimulating waveform is composed of ramp-up (Tu), stable period (Ts), and ramp down (Td). **e** Details on ramp-up (Tu), stable period (Ts), and ramp down (Td) of various stimulating currents. **f** Sample of 9 mA stimulating model. The stimulation was a 1 s duration and 5 s stop. **g** Sample of 9 mA stimulating pulses. Each circle of the stimulating pulse was 12.9 ms with a square wave. SEEG stereoelectroencephalography, tACS transcranial alternating current stimulation.

This study was a part of a clinical trial registered at ClinicalTrials.gov (ID NCT04560959), in which the effect of tACS on functional responses of patients with epilepsy who had already received SEEG electrode implantation was assessed. Meanwhile, the electric potentials in 3 brain regions (hippocampus, insula, and amygdala) at different electrical current strengths were recorded with the SEEG electrodes. After the tACS stimulation, participants received personalized interventions, including vagus nerve stimulation, stereotactic radiofrequency thermocoagulation, or lobectomy. For the current study, only the results of local field potentials with SEEG recording were reported (Fig. 1). The researcher (GZ), who was responsible for calculating LFPs, did not know the details on the delivered different alternating currents.

### Study participants

Participants were recruited via outpatients clinic and posters. All patients underwent a systematic evaluation for surgical treatment of DRE at Xuanwu Hospital between September 2020 and April 2021. The study was approved by the Institutional Review Board in accordance with the ethical standards of the Declaration of Helsinki. An informed consent was obtained from each participant before any study-related procedures were performed.

The inclusion criteria were: (1) 14–60 years old, male or female, Han Chinese; (2) Met the criteria for DRE, defined as failure to respond to two adequate trials of antiepileptic drug treatments (monotherapies or combinations) with adequate dose(s) and duration(s) to render a 2-year free of seizure [27]; (3) Had distinct epilepsy types in each lobe determined by epileptic symptomatology, cranial computed tomography (CT), brain magnetic resonance imaging (MRI), video-electroencephalogram (EEG), positron emission tomography (PET), magnetoencephalography (MEG) and other comprehensive localization methods; (4) Required surgical implantation of SEEG electrodes to determine the anatomical proximity of the epileptogenic focus to the functional brain, including the motor and sensory cortex, and language areas and/or the epileptogenic foci.

The exclusion criteria were: (1) Had progressive encephalopathy or progressive structural damage in the central nervous system; (2) Had significant heart, liver, renal insufficiency, and other medical diseases; (3) Had severe side effects from taking antiepileptic drugs at the time of enrollment and made patients inappropriate for SEEG; (4) Had significant intellectual disability; (5) Had a previous or current alcohol and drug abuse; (6) Had any contraindication to MRI.

### Evaluation for SEEG electrode implantation

Since SEEG is an invasive procedure, the selection of patients for SEEG electrode implantation was decided by a multidisciplinary team at the Epilepsy Center of Xuanwu Hospital. The team included neurologists, neurosurgeons, neuropsychiatrists, pediatric neurologists, neuropsychological rater, patients, and their family members. The determination for SEEG electrodes was based on a comprehensive stage I evaluation of each patient, including a detailed epilepsy history, video-EEG recording, MRI, PET or MEG, and other non-invasive localization methods [28]. If the epileptogenic lesion(s) and epileptogenic zone for a patient could be localized after stage I evaluation and surgical resection would not affect the function of the relevant cerebral cortex, and a standard surgical resection procedure was performed for the patient.

For those who could not have the surgical procedure after the stage I evaluation, they would have stage II evaluation for SEEG. Patients would be considered for SEEG if they had one of the following conditions: (1) Head MRI did not show definite epileptogenic lesions or only had suspicious lesions; (2) Head MRI revealed a large lesion, including an important area of functional expression cortex or there was a risk of damage to functional areas during surgical resection; (3) The localization of the seizure onset zone revealed by clinical symptoms, video EEG findings, head MRI, FDG-PET, and other examination findings was inconsistent; (4) The individual had multiple epileptogenic zones; (5) The individual and his/her close family members required more precise cortical resection and were willing to accept the risk of implantation of SEEG electrodes.

### Implantation of SEEG electrodes

The SEEG electrodes are semi-rigid platinum/iridium depth electrodes (Marseille: Alcis, Besançon, France) with between 5 to 16 contacts. The contacts are 2 mm in length, 0.8 mm in diameter, and 1.5 mm apart. A high-resolution CT scan (Siemens) of the entire skull was acquired, and image fusion was performed with preoperative volumetric T1 MRI sequences (3.0 T, Siemens) using Robotic Stereotactic Assistance (Medtech, Montpellier, France). These brain imaging data were used to guide the implantation of SEEG electrodes. In addition, contrast-enhanced MRI (3.0 T, Siemens) was obtained and fused to avoid major blood vessel damage during the design of the electrode trajectories.

The design of the SEEG, including the number of electrodes and implantation sites, was conceptualized to explore a hypothetical localization of the seizure onset zone specific to each patient. The hypothetical localization of the seizure onset zone was “generated” with the information of the non-invasive evaluation and clinical justifications. After the hypothetical seizure zone was finalized, all SEEG electrodes were inserted one by one using an oblique approach in each participant under general anesthesia. Finally, all participants underwent CT scans after electrode implantation to confirm the exact location of each electrode and to assess potential postoperative complications. The slice thickness for T1 MRI and CT scans after electrode implantation were both l mm for precise electrode localization.

### Reconstruction of the path and location of depth electrodes in the brain

Postoperative 3D CT scans were first registered to the preoperative 3D T1-weighted MRI space using the general registration module in the 3D slicer software (https://www.slicer.org). Here, the T1 image was set as a fixed volume, while the CT image was defined as a moving volume. Then, the exact contact location of each SEEG electrode was reconstructed using the registered postoperative CT images by the markup module of the 3D Slicer software.

### Creation of a 3D atlas of SEEG electrodes in the hippocampus, insula, and amygdala

Based on preoperative T1 images, the cortex surface and subcortical structure segmentation were conducted with Freesurfer’s (http://surfer.nmr.mgh.harvard.edu) “recon-all” commands to produce individualized labels of the pia mater and white matter surfaces and brain regions. Then, the 3D views of the hippocampus, insula, and amygdala structures were reconstructed using the “threshold” effect in the “Segment Editor” module of the 3D Slicer software. The accurate positions of depth electrode contacts within the region of interest were depicted as 3D hippocampus, insula, and amygdala models based on Desikan-Killiany-Tourville Atlas [29].

### SEEG recording

SEEG was recorded continuously under video surveillance for 4 to 11 days to capture at least three habitual seizures to determine epileptical areas for treatment planining, and local field potentials (LFPs) were measured during tACS to study the effect of tACS in deep brain regions. SEEG signals were recorded by a 256-channel recording system Natus Neurology PK1171 (Nicolet, America) at a sampling frequency of 2,048 Hz with reference to common and ground contacts in the white matter or skull to avoid contact deficit in SEEG recordings [30, 31]. In patients with reference contacts in the skull, the SEEG signals were referenced against two contacts located in the vertex region of the scalp midline between the Cz and Fz electrode positions of the international 10/20 system [32]. The anterior one was common, and the other was ground. In those with two adjacent white matter contacts, the one near the cortical surface was common, and the other was ground [30].

In the recording system, the difference in electric potentials between the two contacts, i.e., an active and a common contact, were fed into the differential amplifier of the corresponding channel [33]. Additionally, SEEG recordings of all contacts were documented for each participant during the tACS stimulation procedure, which was performed the day after the participant had completed SEEG recordings for epileptogenic foci localization.

### tACS stimulation

The tACS stimulation procedure was performed using a similar method as described previously [14, 15]. Briefly, participants reclined comfortably on the hospital bed and received alternating current stimulation delivered by an FDA-approved tACS device (Nexalin Technology, Inc., Houston, TX, USA). The electrodes were equally distributed from the forehead to the mastoid regions (Fig. 1). The tACS was administered by trained nurses following standardized instructions. Patients were advised to remain in a relaxation state with minimal communication with the nurses. According to the international 10/20 system, a 4.45 cm × 9.53 cm electrode was placed on the forehead parallel to Fpz, Fp1, and Fp2. Two 3.18 cm × 3.81 cm electrodes were fixed on both sides of the mastoid region. The current was started at 1 mA and increased in 2 mA increments until a maximum of 15 mA (Fig. 1a). The duration of each current intensity was 300 seconds with different time intervals between different current intensities (Fig. 1b, c, d, e, f). 5-second intervals were used between currents of 1 and 3 mA, 3 and 5 mA, 5 and 7 mA, and 7 and 9 mA; 6-second intervals were used between currents of 9 mA and 11 mA; 7 seconds between 11 mA and 13 mA, and 10 seconds between 13 mA and 15 mA (Fig. 1e). The frequency of the currents was 77.5 Hz ( ± 10%). The stimulation ended after 60 min of current duration at 15 mA and 77.5 Hz (Fig. 1a). The stimulation current was a square wave and the amplitude was referred from zero to peak (Fig. 1).

### Data processing

SEEG raw data were first loaded into MATLAB (MathWorks Inc., Natick, MA) using EEGLAB (https://sccn.ucsd.edu/eeglab). Unless otherwise stated, data processing was performed using customized MATLAB codes with the following steps:

1. If nearly a full screen of saturation voltages with peak to peak voltages of more than 17 mV in the raw data of a contact recorded by Natus Neurology PK1171, the contact was considered to be a suspicious broken contact. After the frequency spectrum analysis of the raw signal on the suspicious broken contact using Fast Fourier Transform (FFT), a broken contact was confirmed if the fundamental frequency (50 Hz and 220 V) and its higher-order harmonics of 50 Hz were observed. Data on the broken contacts were not analyzed further.
2. The frequency spectrum of the raw signal at each active contact was calculated using FFT. Regardless of power frequency interference at 50 Hz and EEG activity at less than 30 Hz, the maximum frequency component was extracted to see whether it was equal to the frequency of stimulating currents at 77.5 Hz.
3. All signals were filtered with a band-pass filter, by using “bandpass” command in MATLAB, from 70 to 85 Hz to retain only the stimulating frequency of 77.5 Hz, as the 77.5 Hz frequency component was considered as delivered signals while other frequency components were all considered as noises.
4. To analyze the LFPs at different stimulation currents, the SEEG signal at each active contact was divided into nine sections according to Fig. 1, including no stimulation and stimulation starting from 1 mA and increasing in 2 mA increments until reaching 15 mA.
5. In each section, the root mean square (RMS) of the voltage was also calculated for each contact to verify the multiple relationships between the amplitudes of different stimulation currents and the LFPs. RMS voltages at 0 mA (i.e., no alternation current stimulation of the brain when the tACS device was turned on) and 1–15 mA currents were calculated using the artifact-free signal at each electrode’s active contact for a duration of 180 seconds, within the epoch of 300 seconds for each stimulating current.

We calculated the normalized LFPs, which were the RMS voltages elicited by different external currents at one contact divided by 1/15 of the RMS voltage elicited by a 15 mA stimulation current on the same contact, to better observe whether the intracranial voltages varied proportionally to the stimulation current.

### Statistical analysis

LFPs values were expressed as median (interquartile range, IQR). The mean LFPs of all contacts in the targeted regions (hippocampus, insula, and amygdala) were calculated. The association of the average LFPs in target regions with different extracranial currents of 0, 1, 3, 5, 7, 9, 11, 13, and 15 mA were explored by linear regression analysis. Furthermore, the variations of LFPs for each active contact at different current intensities in the target areas were detailed for each participant. Finally, the normalized LFPs of the deepest contact of each electrode in the target areas were presented. For other active contacts in the implanted electrodes of all participants, their LFPs were also calculated by utilizing the analytical procedure described above, and their association with the extracranial alternating currents was examined by linear regression analysis.

The Kruskal-Wallis test was used for overall comparisons of the mean LFPs in the target areas at different current intensities. The Dunnett method was used to rank the differences between the eight stimulation current groups and the 0 mA current group. Bonferroni adjustment was conducted for multiple testings. Data analysis was performed with SAS statistical software, version 9.4 (SAS Institute Inc., Cary, NC, USA). A value of *p* < 0.05 was considered statistically significant.

## Results

### Participants

Forty-five DRE patients met the criteria of DRE [27] were screened, and 15 were assessed by SEEG. Of the 15 patients with SEEG, three were excluded because they were younger than 14 years old, and one withdrew informed consent before the implementation of tACS procedure on the day (Fig. 1a). Finally, eleven patients (5 males, 6 females, 25.4 ± 5.5 years of age) completed this study. During the tACS, none of the patients experienced seizure occurrence. After the tACS, all patients received clinically individualized interventions, including that one received vagus nerve stimulation, three received stereotactic radiofrequency thermocoagulation, and seven received lobectomies. The baseline clinical features of all participants, including antiepileptic drugs, epileptic onset zones, and interventions are shown in Table 1.

**Table 1:** Demographic and clinical features of patients (*n* = 11).

Patient ID	Age/Sex (M/F)	Age onset(Y)	Disease duration(Y)	AEDs	Epileptic onset zone	Types of epileptic seizures	Interventions	Medical comorbidities
1	18/M	8	10	VBA, LEV	Bil-Par, Bil-Tem	FGTCS	VNS	None
2	26/F	17	9	CBZ, LTG, LEV	R-Tem	FIAS	RF-TC of Hippocampal complex	None
3	27/F	8	19	OXC, LEV	L-Fron	FGTCS	Inferior frontal lobectomy^a^	None
4	37/F	9	28	CBZ, VPA	R-Tem, R-Par, R-Occ	FGTCS	Anterior temporal lobectomy^a^	Hemianopsia
5	21/F	16	5	LEV, LTG	L-Par, L-Tem	FGTCS	Anterior temporal lobectomy^a^	None
6	31/M	26	5	OXC, LEV	L-Tem	FGTCS	Anterior temporal lobectomy^a^	None
7	24/M	21	3	OXC, LEV	L-Tem	FIAS	Anterior temporal lobectomy^a^	None
8	28/M	26	2	OXC, VPA	L-Tem	FIAS	RF-TC of Hippocampal complex	None
9	19/F	8	11	CBZ, VPA, LEV	R-Tem	FGTCS	RF-TC of Hippocampal complex	None
10	25/M	10	15	OXC, LEV	L-Tem	FGTCS	Anterior temporal lobectomy^a^	None
11	23/F	12	11	CBZ, PHT	Bil-Tem	FGTCS	Anterior temporal lobectomy^a^	None

*AEDs* Antiepileptic drugs, *Bil-Par* Bilateral parietal cortex, *Bil-Tem* Bilateral temporal cortex, *CBZ* Carbamazepine, *FCD* Focal cortical dysplasia, *F* female, *FIAS* focal impaired awareness seizure, *FGTCS* focal to generalized tonic-clonic seizure, *L* Left, *LEV* Levetiracetam, *L-Fron* Left frontal cortex, *L-Par* Left parietal cortex, *LTG* Lamotrigine, *L-Tem* Left temporal cortex, *M* Male, *NA* Not applicable, *OXC* Oxcarmazepine, *PHT* Phenytoin, *RF-TC* Radiofrequency thermocoagulation, *R-Occ* Right occipital cortex, *R-Par* Right parietal cortex, *R-Tem* Right temporal cortex, *VNS* Vagus nerve stimulation, *VPA* Valproate, *Y* Year.

^a^Lobectomy means lobe partial resection.

### Electrodes locations and contacts for all participants

**Fig. 2:**
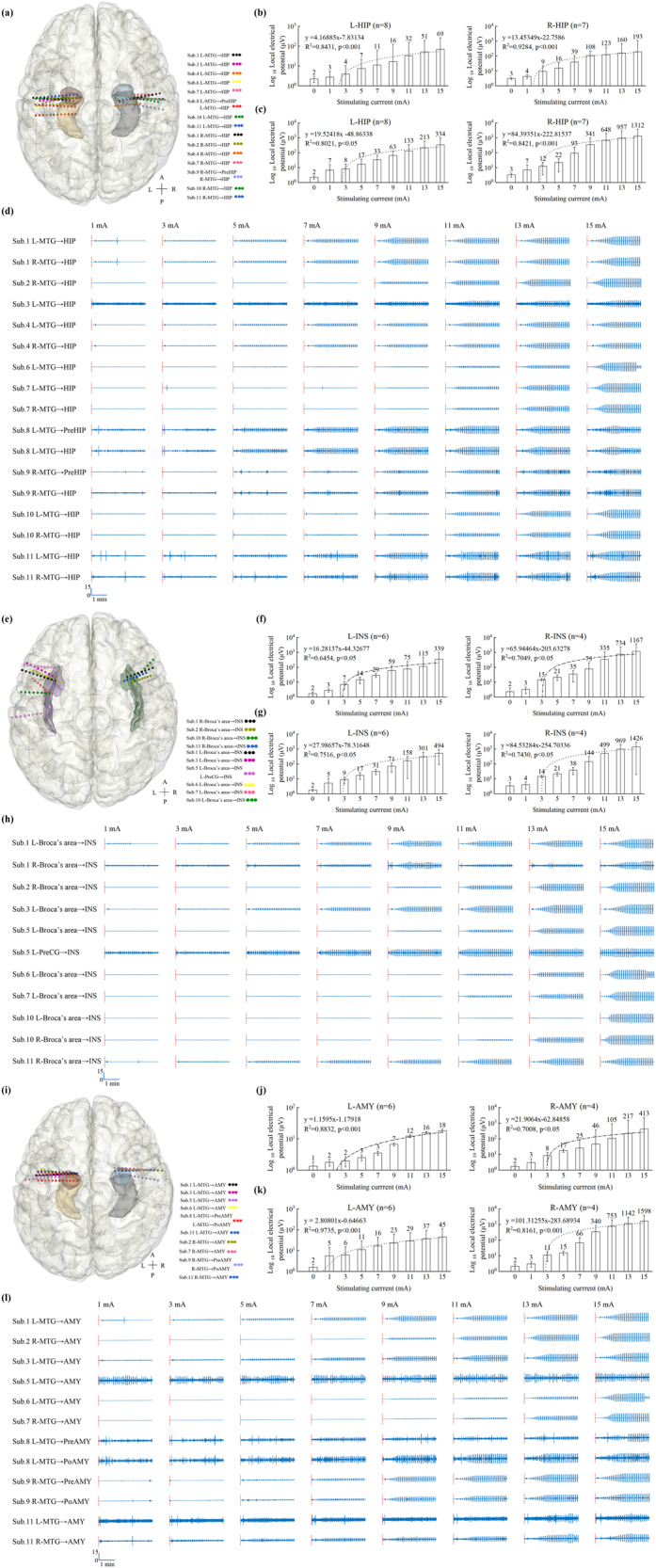
Depth electrodes and the average local field potentials in hippocampus (left, *n* = 8; right, *n* = 7), insula (left, *n* = 6; right, *n* = 4), and amygdala (left, *n* = 6; right, *n* = 4). **a**, **e**, **i** Total intracranial electrodes in the hippocampus, insula, and amygdala, respectively. **b**, **f**, **j** The average local field potentials, represented as median and interquartile range, were significantly linearly correlated with the increasing extracranial currents (all *p* < 0.05) in hippocampus, insula, and amygdala, respectively. **c**, **g**, **k** Positive correlations of the average local field potentials, expressed as the mean and standard deviation, with the increased stimulation currents were found in hippocampus, insula, and amygdala, respectively (all *p* < 0.05). **d**, **h**, and **l** The observable variations of local field potentials of the deepest contacts in hippocampus, insula, and amygdala within 180 seconds after the tACS intervention, respectively. All illustrations were standardized based on their raw SEEG signals. A anterior, AMY Amygdala, HIP Hippocampus, INS Insula, L left, MTG middle temporal gyrus, P posterior, PoAMY post-amygdala, PreAMY pre-amygdala, PreCG precentral gyrus, PreHIP pre-hippocampus, R right, Sub subject.

92 electrodes were available with 953 contacts in 11 patients (Supplementary Figs. S1–S12, Videos S1 and S2, and Table S1). The schematic diagram of SEEG electrode contact was illustrated in Supplementary Fig. S13. 136 contacts from 40 electrodes were located in the target regions: 36 contacts of 9 left hippocampus in 8 patients, 33 contacts of 8 right hippocampus in 7 patients (Of them, 1 contact was broken in patient No.4), 17 contacts of 7 left insula in 6 patients, 9 contacts of 4 right insula in 4 patients, 25 contacts of 7 left amygdala in 6 patients, and 16 contacts of 5 right amygdala in 4 patients, respectively (Table 2; Fig. 2a, e, i). None of the participants had surgery-related complications. During tACS, none of the patients experienced seizures. Also, we selected EEG data from 10 min before and after the tACS stimulation for epileptic spike counting, and there was no significant different on epileptic spikes between the pre- and post-tACS in 11 patients revealed by paired T test (*p* = 0.645) (Supplementary Table S2).

**Table 2:** Electrodes locations and contacts in the targeted brain areas of 11 patients.

Patient No.	Electrodes/total contacts	Trajectories of depth electrodes (contacts/total contacts)	Contacts/HIP, INS, and AMY	Ground & common contacts
1	5/54	TH: L-MTG → HIP (3/12); B: L-Broca’s area→INS (2/10); TA: L-MTG → AMY (4/12); TH’: R-MTG → HIP (4/12); B’: R-Broca’s area→INS(2/8).	3/L-HIP, 4/R-HIP; 2/L-INS, 2/R-INS; 4/L-AMY.	Skull^a^
2	3/32	TH’: R-MTG → HIP (4/12); B’: R-Broca’s area→INS (2/8); TA’: R-MTG → AMY (3/12).	4/R-HIP; 2/R-INS; 3/R-AMY.	Skull^a^
3	3/32	TH: L-MTG → HIP (5/12); B: L-Broca’s area→INS (2/8); TA: L-MTG → AMY (4/12).	5/L-HIP; 2/L-INS; 4/L-AMY.	6^th^ and 7^th^ contact of L-MTG → HIP, respectively
4	2/16	TH: L-MTG → HIP (4/8); TH’: R-MTG → HIP (5/8)^b^.	4/L-HIP; 5/R-HIP^b^ (of them, TH’-4 is broken).	Skull^a^
5	3/35	B: L-Broca’s area→INS (2/10); C: L-PreCG→INS (2/10); TA: L-MTG → AMY (4/15).	4/L-INS; 4/L-AMY.	7^th^ and 8^th^ contact of L-MTG → AMY, respectively
6	3/34	TH: L-MTG → HIP (4/12); B: L-Broca’s area→INS (5/10); TA: L-MTG → AMY (3/12).	4/L-HIP; 5/L-INS; 3/L-AMY.	5^th^ and 6^th^ contact of L-MTG → AMY, respectively
7	4/36	TH: L-MTG → HIP (4/10); B: L-Broca’s area→INS (2/8); TH’: R-MTG → HIP (5/10); TA’: R-MTG → AMY (3/8).	4/L-HIP, 5/R-HIP; 2/L-INS; 3/R-AMY.	6^th^ and 7^th^ contact of L-lORB→mORB
8	4/48	TH1: L-MTG→PreHIP (4/12); TH: L-MTG → HIP (4/12); TA1: L-MTG→PreAMY (4/12); TA2: L-MTG→PoAMY (3/12).	8/L-HIP; 7/L-AMY.	7^th^ and 8^th^ contact of L-MTG→PreAMY
9	4/48	TH1’: R-MTG→PreHIP (3/12); TH’: R-MTG → HIP (4/12); TA1’: R-MTG→PreAMY (3/12); TA2’: R-MTG→PoAMY (3/12).	7/R-HIP; 6/R-AMY.	14^th^ and 15^th^ contact of R-MTG→PreHIP
10	4/40	TH: L-MTG → HIP (4/12); B: L-Broca’s area→INS (2/8); TH’: R-MTG → HIP (4/12); B’: R-Broca’s area→INS (2/8).	4/L-HIP, 4/R-HIP; 2/L-INS, 2/R-INS.	7^th^ and 8^th^ contact of L-MTG → HIP, respectively
11	5/56	TH: L-MTG → HIP (4/12); TA: L-MTG → AMY (3/12); TH’: R-MTG → HIP (4/12); B’: R-Broca’s area→INS (3/8); TA’: R-MTG → AMY (4/12).	4/L-HIP, 4/R-HIP; 3/R-INS; 3/L-AMY, 4/R-AMY.	7^th^ and 8^th^ contact of L-MTG → AMY, respectively

*AMY* Amygdala, *HIP* Hippocampus, *INS* Insula, *L* Left, *l* lateral, *m* medial, *MTG* middle temporal gyrus, *No.* number, *ORB* orbital frontal cortex, *PoAMY* post-amygdala, *PreAMY* pre-amygdala, *PreCG* precentral gyrus, *PreHIP* pre-hippocampus, *R* right.

^a^Skull means that the ground and common reference contacts were placed in the top cranial midline at a 2 cm distance.

^b^4^th^ contact (i.e. TH’-4) in TH’: R-MTG → HIP was broken, its data was not included for the final analysis (Figure 2 and Supplementary Table S6).

### Changes in LFPs in the hippocampus, insula, and amygdala

After the frequency spectrum analysis process for SEEG raw data, three broken contacts were found and verified in all implanted electrode contacts. The remaining active contacts were of good quality, with LFP frequencies of 77.75–77.08 Hz and 77.58–77.08 Hz before and after the 70–85 Hz band-pass filter, respectively. These frequencies were consistent with the current frequencies for a given alternation current stimulus (Supplementary Table S3). An example of the frequency spectrum analysis process for SEEG raw data of the 1^st^ contact of the left middle temporal gyrus→hippocampus (TH) in Subject No. 4 is provided (Supplementary Fig. S14).

All LFPs on active contacts in the hippocampus, insula, and amygdala (Supplementary Table S4) and other brain areas (Supplementary Table S5) were calculated. The results of the average LFPs in the hippocampus, insula, and amygdala with the stepwise increase of the extracranial alternating current were shown in Fig. 2. Whether expressed as median and interquartile range (IQR) (Fig. 2b, f, j) or mean and standard deviation (SD) (Fig. 2c, g, k), the average changes of LFPs in the left hippocampus, right hippocampus, left insula, right insula, left amygdala, and right amygdala were linearly correlated with the magnitude of the extracranial current stimulation (all *p* < 0.05).

The changes of average LFPs in each subject’s hippocampus, insula, and amygdala showed that there were significant correlations on the average LFPs changes with the stepwise increase of the extracranial alternating currents in each subject’s left and right hippocampus (all *p* < 0.01), left and right insula (*p* < 0.01), except for the left insula of subject No.10 (*p* > 0.05), and left and right amygdala (all *p* < 0.01) (Supplementary Fig. S15 and Table S6).

The LFPs of the deepest intracranial electrode contacts increased significantly with increasing external currents, especially at 15 mA, and changes in LFPs were clearly visible in 17 contacts in the hippocampus in 10 patients (all *p* < 0.01), 11 contacts in the insula in 8 patients (*p* < 0.01), except for the deepest contact of left insula (close to the cerebrospinal fluid) of subject No.10 (*p* > 0.05), and 12 contacts in the insula in 9 patients (all *p* < 0.01) (Supplementary Table S7). For comparison purposes, all LFPs changes in the deepest contacts in the left and right hippocampus, insula, and amygdala were normalized at different current magnitudes. Although some artifacts were also seen in all deepest contacts embedded in the hippocampus of the 10 patients, the insula of the 8 patients, and the amygdala of the 9 patients, intracranial LFPs gradually increased with increasing extracranial currents, respectively (Fig. 2d, h, i).

We further explored the correlations between LFPs changes of other contacts of implanted electrodes in addition to the electrodes in hippocampus, insula, and amygdala and extracranial alternating currents in each subject by linear regression analysis. We found that LFPs changes at the most contacts linearly correlated with the increase of transcranial alternating currents (*p* < 0.05), with the exception of 8 ground contacts, 8 reference contacts, 3 broken contacts, 6 contacts near the cerebrospinal fluid, 1 contact in the sulcus, and 1 contact close to ground contact (Supplementary Table S7).

### Comparison of LFPs produced by different alternating currents in the hippocampus, insula, and amygdala with those at 0 mA

LFPs at 0, 1, 3, 5, 7, 9, 11, 13, and 15 mA in the left and right insula, left and right hippocampus, and left and right amygdala are presented (Table 3). The Kruskal-Wallis tests revealed statistically significant differences in LFPs on both sides of the hippocampus, insula, and amygdala at different currents (all *p* < 0.01). Moreover, Dunnett’s tests revealed that LFPs in these brain regions were significantly different in the left and right hippocampus, right insula, and right amygdala when stimulation currents exceeded 5 mA, with the left amygdala exceeding 7 mA and the left insula exceeding 3 mA, compared to 0 mA. Furthermore, Bonferroni adjustments confirmed that more than 7 mA was required for the left hippocampus, right insula, and right amygdala, more than 11 mA for the left amygdala, and more than 5 mA for the right hippocampus and left insula to produce statistical differences in LFPs in these brain regions in comparison to 0 mA.

**Table 3:** Comparisons of the average local field potentials induced by different stimulating currents in the hippocampus, insula, and amygdala.

Current (mA)	Local field potentials (uV)					
	Hippocampus		Insula		Amygdala	
	Left (*n* = 8) Median (IQR)	Right (*n* = 7) Median (IQR)	Left (*n* = 6) Median (IQR)	Right (*n* = 4) Median (IQR)	Left (*n* = 6) Median (IQR)	Right (*n* = 4) Median (IQR)
0	2.24 (1.73, 2.64)	3.07 (2.59, 3.31)	1.70 (1.41, 2.02)	2.30 (1.84, 4.83)	1.33 (1.21, 1.75)	1.67 (1.60, 2.55)
1	2.74 (2.40, 5.81)	4.30 (3.55, 4.94)	2.85 (2.63, 3.49)	3.22 (2.66, 5.41)	1.77 (1.55, 1.98)	2.99 (1.82, 4.21)
3	3.95 (2.44, 9.83)	9.36 (3.83, 24.62)	7.10 (5.99, 10.31)	14.72 (11.92, 16.30)	1.96 (1.92, 2.96)	8.31 (6.49, 15.62)
5	7.46 (4.37, 24.04)	15.68 (5.23, 34.29)	14.49 (11.18, 18.28)	21.32 (15.72, 26.57)	2.51 (2.00, 6.12)	17.37 (12.19, 18.64)
7	11.04 (7.47, 66.57)	39.45 (9.68,81.67)	28.93 (24.85, 35.58)	34.6 (21.21, 54.56)	3.48 (2.11, 10.30)	25.38 (14.23, 118.25)
9	16.25 (15.91,123.47)	108.05 (17.45,143.35)	59.01 (31.40, 113.12)	75.09 (37.78, 249.52)	6.61 (2.22, 15.66)	45.54 (21.99, 658.15)
11	32.46 (21.94, 144.5)	123.04 (19.76, 466.24)	75.28 (58.12, 274.83)	335.25 (47.44, 949.71)	12.18 (2.64, 20.34)	105.08 (23.9, 1481.23)
13	50.81 (26.61, 177.49)	159.78 (36.15, 927.36)	114.74 (71.80, 536.23)	734.37 (57.21, 881.52)	15.64 (2.65, 27.66)	217.24 (39.94, 2,244.93)
15	69.38 (29.59, 233.62)	193.45 (40.92, 1629.51)	351.60 (133.57, 901.03)	1166.88 (73.15, 2,778.20)	18.11 (3.86, 37.61)	412.79 (45.12, 3150.75)
*p* value^a^	<0.0001	<0.0001	<0.0001	0.0004	0.0032	0.0009
Dunnett results^b^	5mA-15mA	5mA-15mA	3mA-15mA	5mA-15mA	7mA-15mA	5mA-15mA
Bonferroni adjustments^c^	7mA-15mA	5mA-15mA	5mA-15mA	7mA-15mA	11mA-15mA	7mA-15mA

*IQR* interquartile range, *mA* milliampere, *uV* microvolts.

^a^*p* value were obtained from the Kruskal-Wallis test.

^b^Only significant comparisons were presented against 0mA current.

^c^Only significant pairwise comparisions were presented.

## Discussion

To our knowledge, this is the first study to record LFPs at the contacts of SEEG electrodes in the hippocampus, insula, and amygdala during tACS with a current of 77.5 Hz, suggesting that the external alternating currents from tACS can directly penetrate the skin, skull, and brain tissue to reach deeper brain regions. The amplitude of LFPs was positively correlated with the current intensity of tACS (from 1 to 15 mA), suggesting that deep brain tissue can receive different stimuli by manipulating the current of the tACS. More importantly, patients had no seizure activity during tACS.

We also found that the thresholds of stimulation currents that caused changes in LFPs in different brain regions were different, indicating that different alternative currents penetrated to different depths brain tissue. Although the nature of these differences remains unclear, a minimum of 7 mA is required to produce significant changes in LFPs simultaneously compared to 0 mA in three brain regions, suggesting that a current threshold may be necessay for tACS to treat insomnia [15], depression [14] and other disorders. Inconsistent reports of tACS currents below 4 mA for the treament of insomnia and depression [14, 15, 17–20] may be due to insufficient currents to modulate brain function.

An epileptic seizure is required for the efficacy of electroconvulsive therapy (ECT) in treating severe or treatment-resistant depression [34]. Although the alternating currents used for tACS in the current study [14, 15] were much smaller than those used in ECT, it remained unclear if they were safe in patients with DRE. The location of electrode placement is a major factor in any potential seizure with any electrical stimulation and transcranial magnetic stimulation [14, 35]. Seizures are caused by the diffussion of electrical or magnetic stimulation into the motor cortex [36]. To minimize this risk, previous studies of tACS intervention for depression and insomnia have placed electrodes on the earlobes, occipital region, mastoid processes, and temples [16, 21], whereas our study positioned three electrodes on the forehead plus both mastoid areas [15]. Although it remains unclear what happens to the currents used in the various studies in the brain, our results suggest that electrode placed on the forehead plus both mastoid regions with currents of 77.5 Hz and up to 15 mA of tACS [14, 16] is safe and able to deliver currents to deep brain tissue.

Stimulation currents can affect the amount of energy delivered to brain tissue [9, 14–16]. The maximum current we used was 15 mA [14, 16], which is still a low current compared to the larger current of 800–900 mA typical of electroconvulsive therapy [9]. Thus, the low current amplitude of tACS in our study may also be an important reason we did not see epileptic seizures in previous studies [14–16]. The frequency of stimulation might be another fundamental aspect of the overall current delivery. But, there is still a lack of study on the effect of different frequencies on the delivery of stimulation current of tACS to the deep brain structure. So far, it remains unclear what “dose” of tACS is safe and effective for altering brain function [9].

The strengths and weaknesses of our study included: (i) the use of SEEG, as an invasive procedure, to study tACS-related LFPs in awake DRE patients is innovative; and (ii) the use of different tACS currents to establish dose-dependent relationships between tACS currents and LFPs in different brain regions lays the foundation for future design and development of tACS in neuropsychiatric disorders, although the variability in electrodes locations within the same brain structure might be a potential confounder.

However, since all participants were DRE patients and they were taking anticonvulsants. It is unclear to what extent the recurrent epileptic activity and anticonvulsants affected the current delivery of tACS to the different brain regions. It is also unclear if the recurrent epileptic activity and anticonvulsants reduced the risk for tACS-induced epileptic activities. Therefore, the results from the current study may not be generable to patients without a DRE. In addition, we only measured the LFPs at the 77.5 Hz, but other LFPs changes at different frequencies related to the tACS could also occur. An analysis targeting LFPs chages at different frequencies before and after the tACS is worthy of further exploration. Although our previous studies [14–16] of using the maximal setting as at the current study (i.e., a current of 15 mA and a frequency of 77.5 Hz) for the treatment MDD did not show serious side effect, in future studies, the data of the discomfort or interoceptive experience during tACS at different current intensities should be collected. In the current study, no seizure occurred.

Notably, the areas of the tACS electrodes in the current study are innervated by the V1 branch of the trigeminal nerve [37] and the great auricular nerve (originated from cervical plexus) [38]. Stimulating these nerves has been used to treat neuropsychiatric disorders as such migraine headache and depression. From a therapeutic point of view, the therapeutic effect with the tACS in our previous studies [14–16] that used the tACS at the maximal stimulation parameters and the same electrode placements as the current study is likely through the direct stimulation of brain tissues and direct stimulation of peripheral nerves (indirect stimulation to the brain). From a physical point of view, the indirect stimulation to the brain through the direct stimulation of the peripheral nerves could affect the LFPs in brain regions. However, it is unclear how the direct stimulation of the peripheral nerves in the current study affected the LFPs changes at the 77.5 Hz in different brain regions.

In conclusion, the SEEG in our study did provide direct evidence in humans that tACS at 15 mA and 77.5 Hz with a montage of the forehead and both mastoids can deliver currents to different deep brain regions. Since the larger current of ECT with a seizure has a therapeutic effect for depression and some other mental illnesses, there is a threshold range of energy stimulation that can be therapeutically effective without a seizure. Therefore, based on the existing evidence, we speculate that there should be an alternating current intensity range between the weak current such as 2 mA or more used in tACS [14] and the larger current such as 800–900 mA or less applied in ECT [39] that can have a therapeutic effect in reducing depressive or other psychiatric symptoms without causing seizures. The dose-dependent relationship between tACS currents and LFPs suggests that manipulation of tACS currents can alter physiological activity in different brain regions, which is opening a non-invasive avenue to develop a therapeutic approach that will achieve clinically meaningful improvements in individuals suffering from a wide range of brain disorders or abnormal brain function involving the insula, amygdala, and hippocampus.

### Supplementary information

Video S1 All electrodes in an average brain

Video S2 The targeted electrodes in hippocampus, insula and amygdala in an average brain

Supplementary materials
