# Supplementary material for: Evidence of a large current of transcranial alternating current stimulation directly to deep brain regions
Source: Mol Psychiatry. 2023 Jul 19;28(12):5402–10. doi: 10.1038/s41380-023-02150-8 (PMC11041720; doi:10.1038/s41380-023-02150-8)
Supplement: Supplementary file 3 — Supplementary materials [file 41380_2023_2150_MOESM3_ESM.doc]

***Supplementary material for***

**Evidence of a large current of transcranial alternating current stimulation directly to deep brain region**

*Yongzhi Shan^1,2,3*^, Hongxing Wang^4,5*✉^, Yanfeng Yang^1,2,3^, Jiahao Wang^6,7^, Wenfeng Zhao^4^, Yuda Huang^1,2,3^, Huang Wang^4^, Bing Han^4^, Na Pan^4^, Xiukun Jin^4^, Xiaotong Fan^1,2,3^, Yunyun Liu^1,2,3^, Jun Wang^1,2,3^, Changming Wang^1,2,3^, Huaqiang Zhang^1,2,3^, Sichang Chen^1,2,3^, Ting Liu^1,2,3^, Tianyi Yan^8^, Tianmei Si^9^, Lu Yin^10^, Xinmin Li^11^, Fiammetta Cosci^12✉^, Xiangyang Zhang^13✉^, Guanghao Zhang^6,7✉^, Keming Gao^14✉^ and Guoguang Zhao^1,2,3,15✉†^*

†Lead correspondence: ggzhao@vip.sina.com

**Supplementary Figures**

**Fig. S1-S11.** Each individual’s 3D electrodes locations in native space.

**Fig. S12.** Reconstruction of depth electrodes in an “average brain”.

**Fig. S13.** The schematic diagram of SEEG electrode contact.

**Fig. S14.** An example of the frequency spectrum analysis process on SEEG raw data for 1st contact of TH (left middle temporal gyrus→hippocampus) of Subject No. 4.

**Fig. S15**. The changes of average local field potentials in each subject's hippocampus, insula, and amygdala.

**Supplementary Tables**

**Table S1.** Details on electrode contacts.

**Table S2.** Comparison on epileptic spikes between the pre- and post- tACS intervention in 11 patients.

**Table S3.** Primary frequency component at pre & post-the 70-85 Hz bandpass filter.

**Table S4.** Local field potentials of all contacts in hippocampus, insula, and amygdala (uV).

**Table S5.** Local field potentials of other contacts except for hippocampus, insula, and amygdala (uV).

**Table S6.** The linear regression equations on the between the local field potentials in hippocampus, insula, and amygdala with the extracranial currents in an increase.

**Table S7.** Correlations of the local field potentials of all contacts with the alternating currents in all 11 subjects (*p* value).

**Supplementary Videos**

**Video S1.** All electrodes in an "average brain".

**Video S2.** The targeted electrodes in hippocampus, insula and amygdala in an "average brain".

A, Anterior; P, Posterior; L, Left; R, Right.


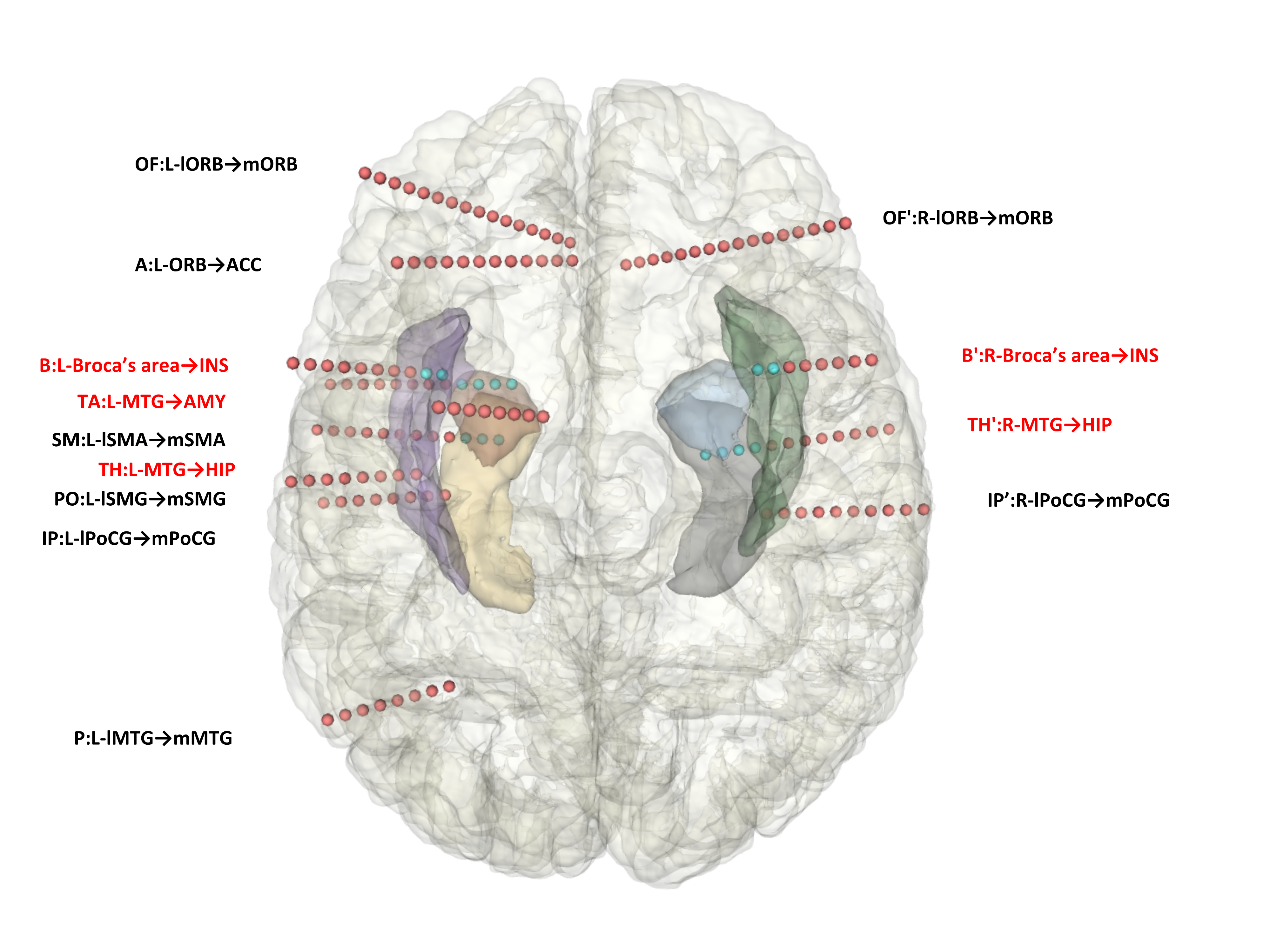


Fig. S1. No. 1 Patient’s 3D electrodes (contacts in targeted brain areas/total contacts). 9 depth electrodes in the left brain and 4 depth electrodes in the right brain in subject No. 1. The electrode marked in blue font indicates that it is in the targeted brain area.

OF, L-lORB→mORB, left-lateral orbital frontal cortex→medial orbital frontal cortex (0/16);

A, L-ORB→ACC, left-orbital frontal cortex→anterior cingulate cortex (0/12);

B, L-Broca's area→INS, left-Broca's area→Insula (2/10);

TA, L-MTG→AMY, left-middle temporal gyrus→Amygdala (4/12);

SM, L-lSMA→mSMA, left-lateral supplementary motor area→medial supplementary motor area (0/8);

TH, L-MTG→HIP, left-middle temporal gyrus→Hippocampus (3/12);

PO, L-lSMG→mSMG, left-lateral supramarginal gyrus→medial supramarginal gyrus (0/8);

IP, L-lPoCG→mPoCG, left-lateral postcentral gyrus→medial postcentral gyrus (0/8);

P, L-lMTG→mMTG, left- lateral middle temporal gyrus→medial middle temporal gyrus (0/8);

OF', R-lORB→mORB, right-lateral orbital frontal cortex→medial orbital frontal cortex (0/16);

B', R-Broca's area→INS, right-Broca’s area→Insula (2/8);

TH', R-MTG→HIP, right-middle temporal gyrus→Hippocampus (4/12);

IP', R-lPoCG→mPoCG, right-lateral postcentral gyrus→medial postcentral gyrus (0/10).

**
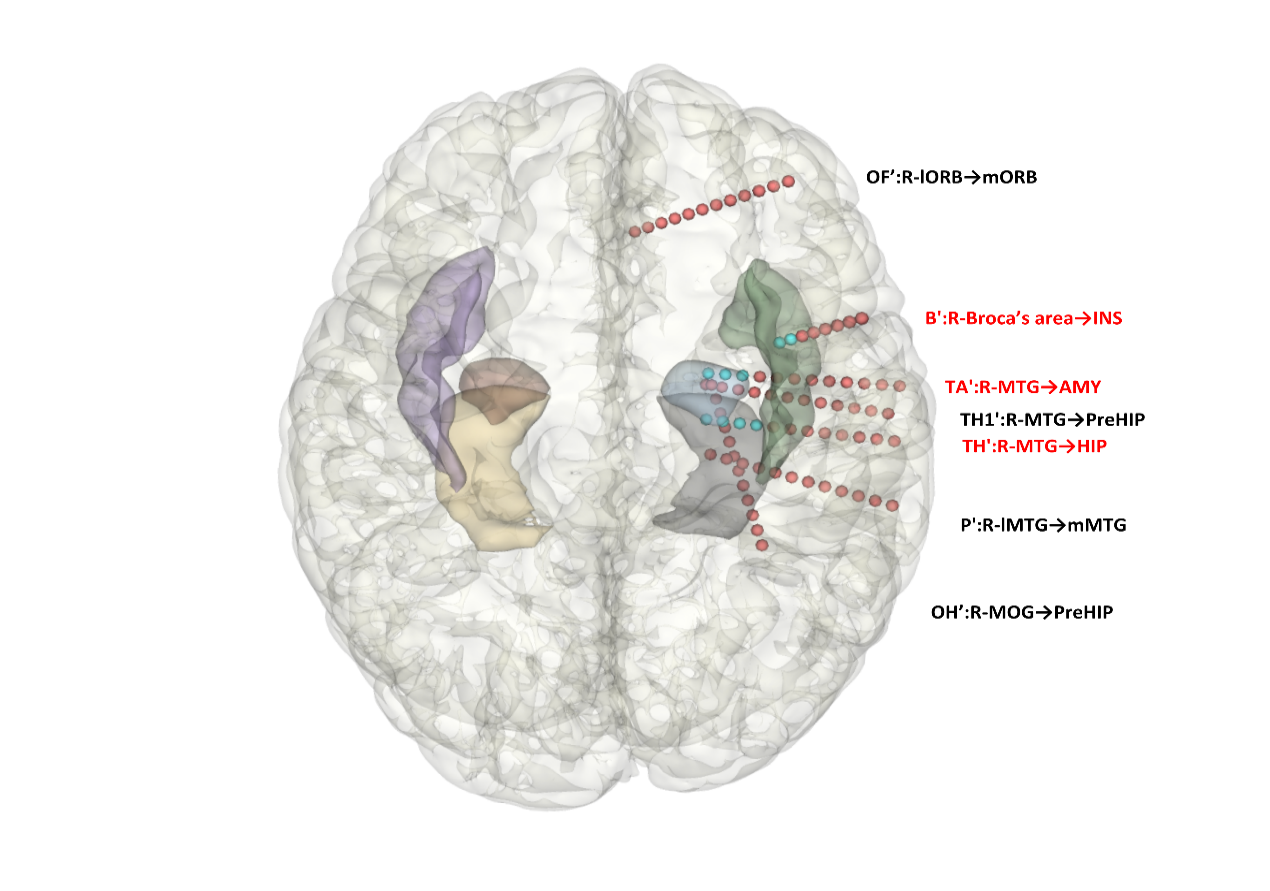
**

Fig. S2. No. 2 Patient’s 3D electrodes (contacts in targeted brain areas/total contacts). 7 depth electrodes in the right brain in subject No. 2. The electrode marked in blue font indicates that it is in the targeted brain area.

OF', R-lORB→mORB, right-lateral orbital frontal cortex→medial orbital frontal cortex (0/12);

B', R-Broca's area→INS, right-Broca's area→Insula (2/8);

TA', R-MTG→AMY, right-middle temporal gyrus→Amygdala (3/12);

TH1', R-MTG→PreHIP, right-middle temporal gyrus→pre-hippocampus (0/12), TH1’ missed in hippocampus;

TH', R-MTG→HIP, right-middle temporal gyrus→Hippocampus (4/12);

P', R-lMTG→mMTG, right-lateral middle temporal gyrus→medial middle temporal gyrus (0/12);

OH', R-MOG→PreHIP, right-middle occipital gyrus→pre-hippocampus (0/12).

**
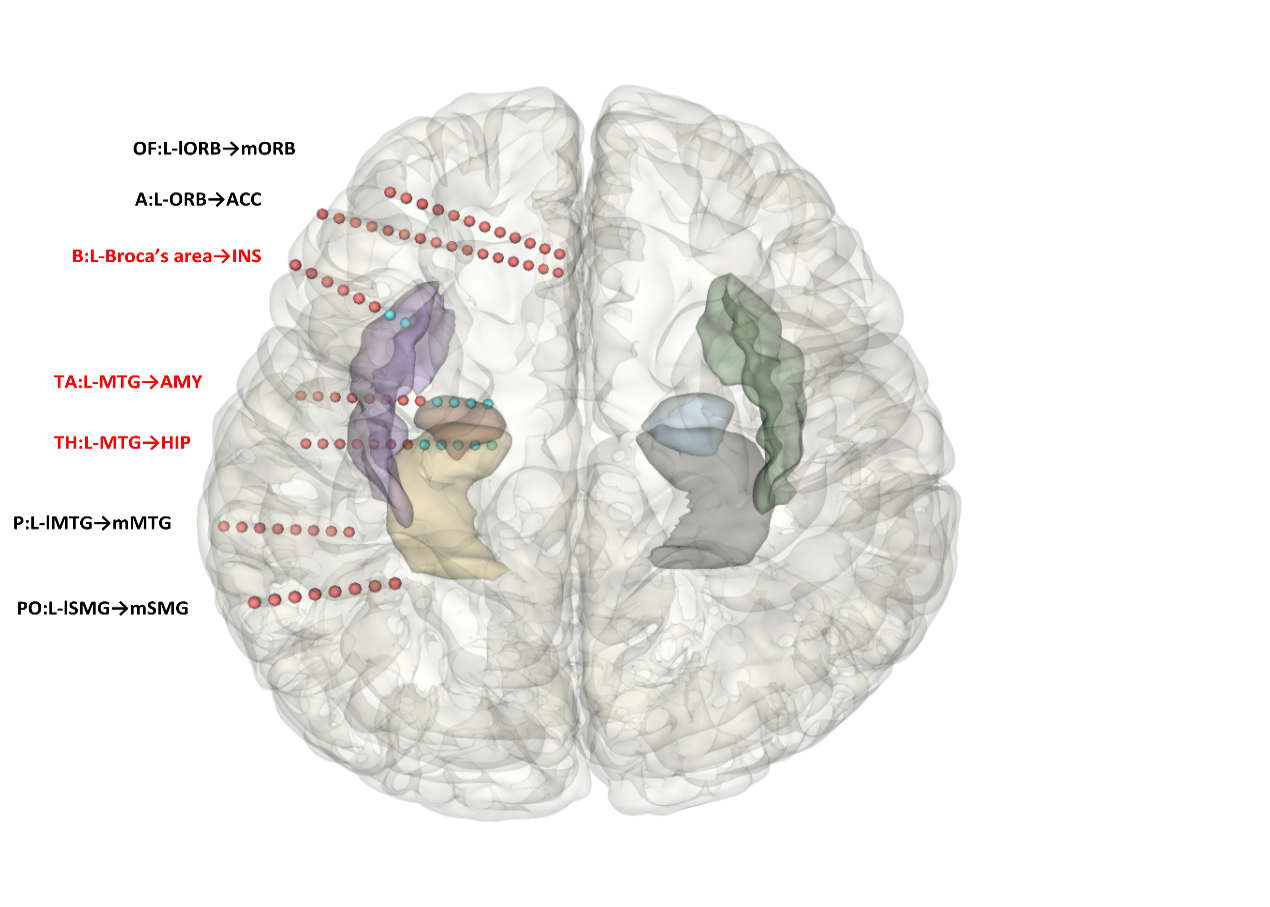
**

Fig. S3. No. 3 Patient’s 3D electrodes (contacts in targeted brain areas/total contacts). 7 depth electrodes in the left brain in subject No. 3. The electrode marked in blue font indicates that it is in the targeted brain area.

OF, L-lORB→mORB, left-lateral orbital frontal cortex→medial orbital frontal cortex (0/16);

A, L-ORB→ACC, left-orbital frontal cortex→anterior cingulate cortex (0/12);

B, L-Broca's area→INS, left-Broca's area→Insula (2/8);

TA, L-MTG→AMY, left-middle temporal gyrus→Amygdala (4/12);

TH, L-MTG→HIP, left-middle temporal gyrus→Hippocampus (5/12);

P, L-lMTG→mMTG, left- lateral middle temporal gyrus→medial middle temporal gyrus (0/8);

PO, L-lSMG→mSMG, left-lateral supramarginal gyrus→medial supramarginal gyrus (0/8).

**
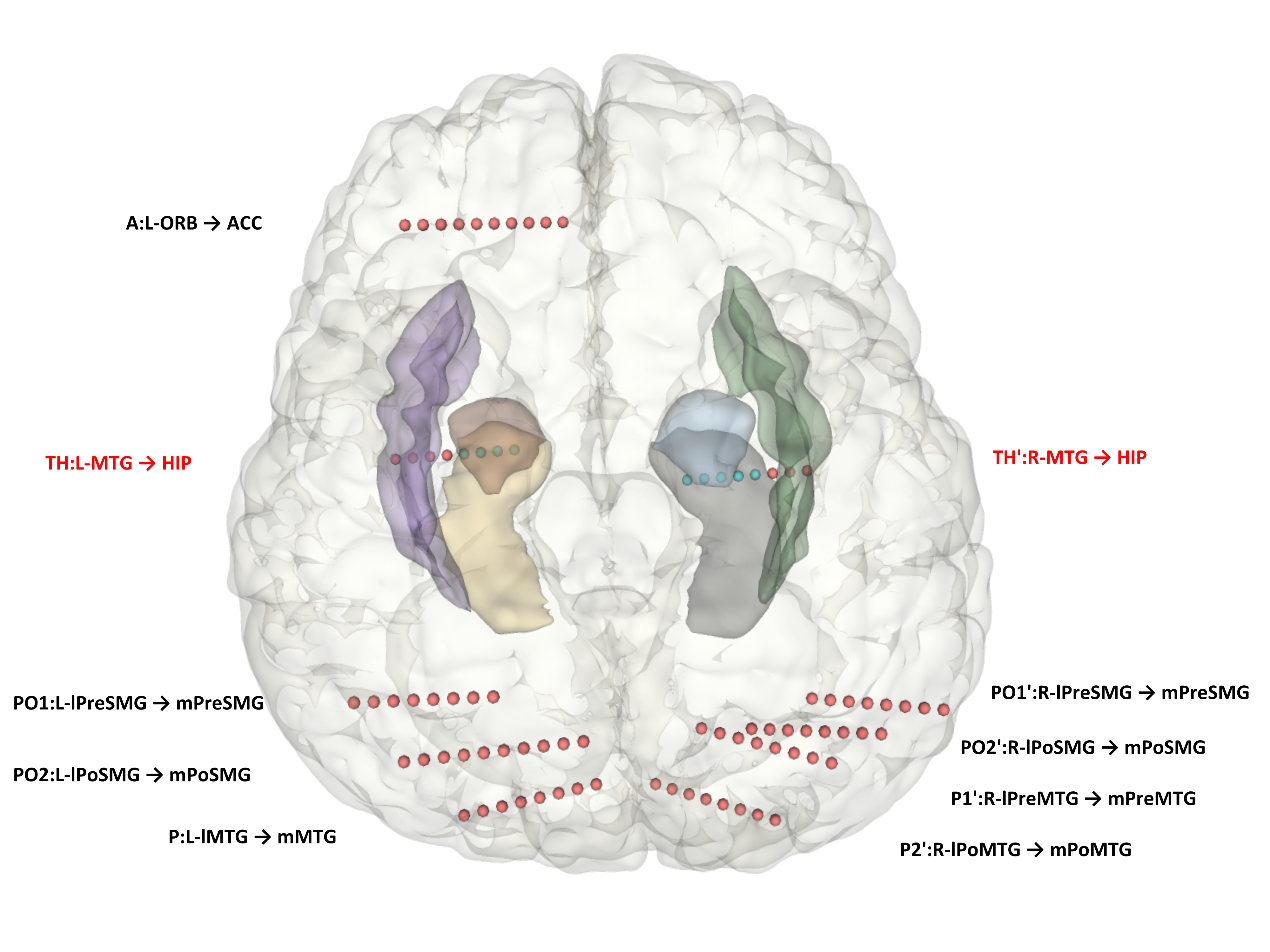
**

Fig. S4. No. 4 Patient’s 3D electrodes (contacts in targeted brain areas/total contacts). 5 depth electrodes in the left brain and 5 depth electrodes in the right brain in subject No. 4. The electrode marked in blue font indicates that it is in the targeted brain area.

A, L-ORB→ACC, left-orbital frontal cortex→anterior cingulate cortex (0/10);

TH, L-MTG→HIP, left-middle temporal gyrus→Hippocampus (4/8);

PO1, L-lPreSMG→mPreSMG, left-lateral pre-supramarginal gyrus→medial pre-supramarginal gyrus (0/8);

PO2, L-lPoSMG→mPoSMG, left-lateral post-supramarginal gyrus→medial post-supramarginal gyrus (0/10);

P, L-lMTG→mMTG, left- lateral middle temporal gyrus→medial middle temporal gyrus (0/8);

TH', R-MTG→HIP, right-middle temporal gyrus→Hippocampus (5/8);

PO1', R-lPreSMG→mPreSMG, right-lateral pre-supramarginal gyrus→medial pre-supramarginal gyrus (0/8);

PO2', R-lPoSMG→mPoSMG, right-lateral post-supramarginal gyrus→medial post-supramarginal gyrus (0/8);

P1', R-lPreMTG→mPreMTG, right-lateral pre-middle temporal gyrus→medial pre-middle temporal gyrus (0/8);

P2', R-lPoMTG→mPoMTG, right-lateral post-middle temporal gyrus→medial post-middle temporal gyrus (0/8).

**
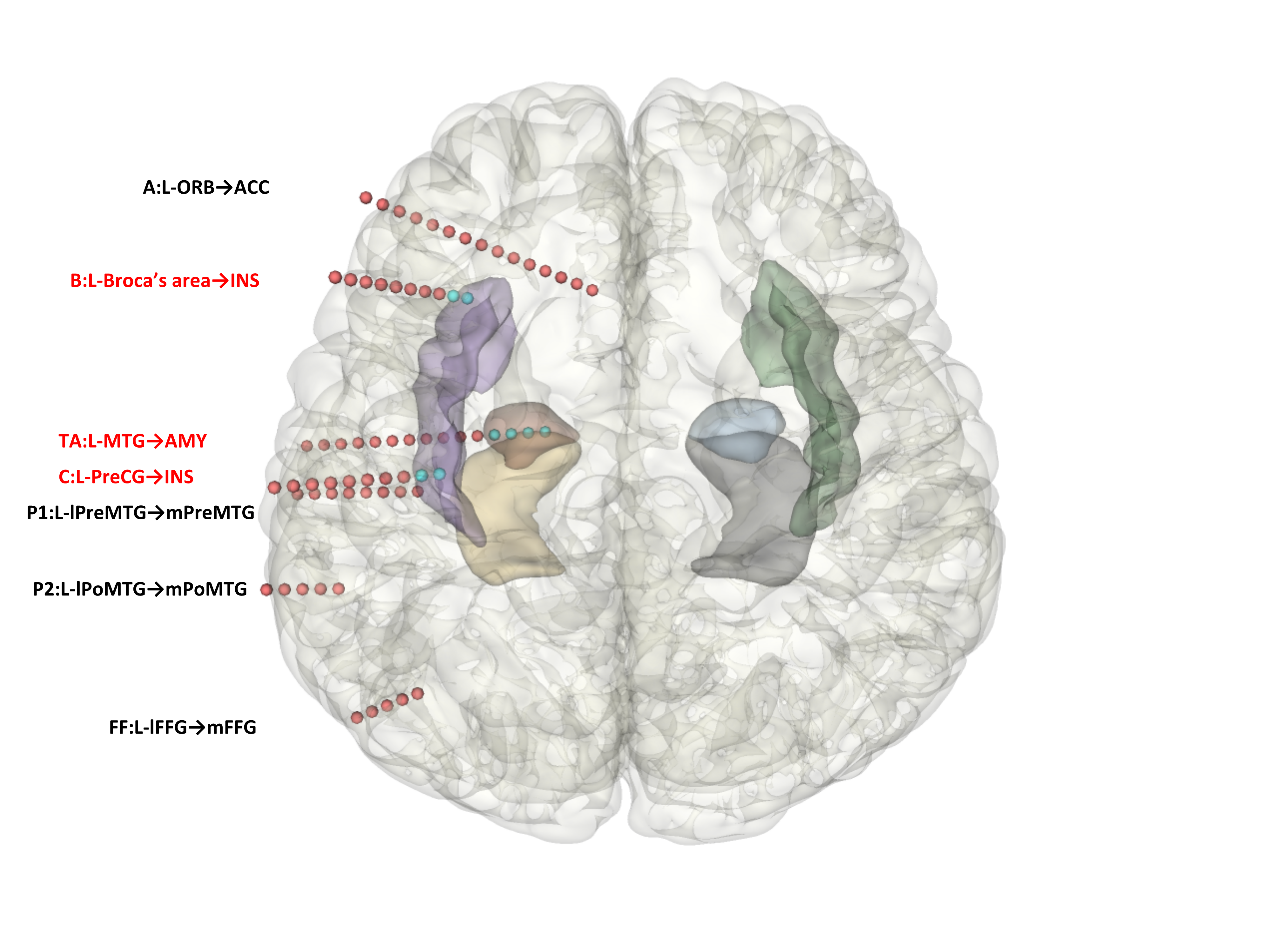
**

Fig. S5. No. 5 Patient’s 3D electrodes (contacts in targeted brain areas/total contacts). 7 depth electrodes in the left brain in subject No. 5. The electrode marked in blue font indicates that it is in the targeted brain area.

A, L-ORB→ACC, left-orbital frontal cortex→anterior cingulate cortex (0/15);

B, L-Broca's area→INS, left-Broca's area→Insula (2/10);

TA, L-MTG→AMY, left-middle temporal gyrus→Amygdala (4/15);

C, L-PreCG→INS, left-precentral gyrus→Insula (2/10);

P1, L-lPreMTG→mPreMTG, left-lateral pre-middle temporal gyrus→medial pre-middle temporal gyrus (0/8);

P2, L-lPoMTG→mPoMTG, left-lateral post-middle temporal gyrus→medial post-middle temporal gyrus (0/5);

FF, L-lFFG→mFFG, left-lateral fusiform gyrus→medial fusiform gyrus (0/5).

**
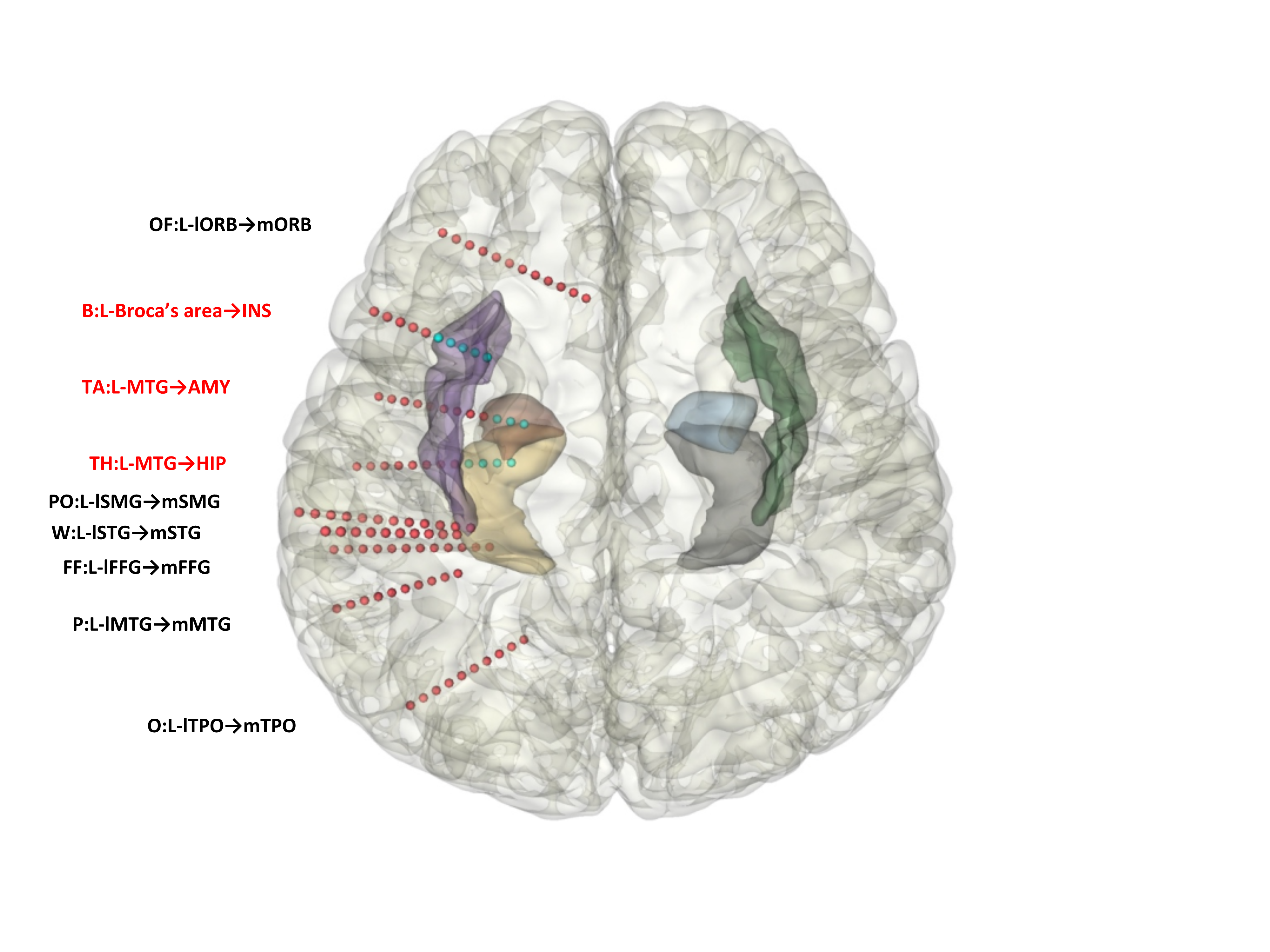
**

Fig. S6. No. 6 Patient’s 3D electrodes (contacts in targeted brain areas/total contacts). 9 depth electrodes in the left brain in subject No. 6. The electrode marked in blue font indicates that it is in the targeted brain area.

OF, L-lORB→mORB, left-lateral orbital frontal cortex→medial orbital frontal cortex (0/12);

B, L-Broca's area→INS, left-Broca's area→Insula (5/10);

TA, L-MTG→AMY, left-middle temporal gyrus→Amygdala (3/12);

TH, L-MTG→HIP, left-middle temporal gyrus→Hippocampus (4/12);

PO, L-lSMG→mSMG, left-lateral supramarginal gyrus→medial supramarginal gyrus (0/10);

W, L-lSTG→mSTG, left-lateral superior temporal gyrus→medial superior temporal gyrus (0/12);

FF, L-lFFG→mFFG, left-lateral fusiform gyrus→medial fusiform gyrus (0/10);

P, L-lMTG→mMTG, left- lateral middle temporal gyrus→medial middle temporal gyrus (0/12);

O, L-lTPO→mTPO, left-lateral temporal occipital junction→medial temporal occipital junction (0/10).

**
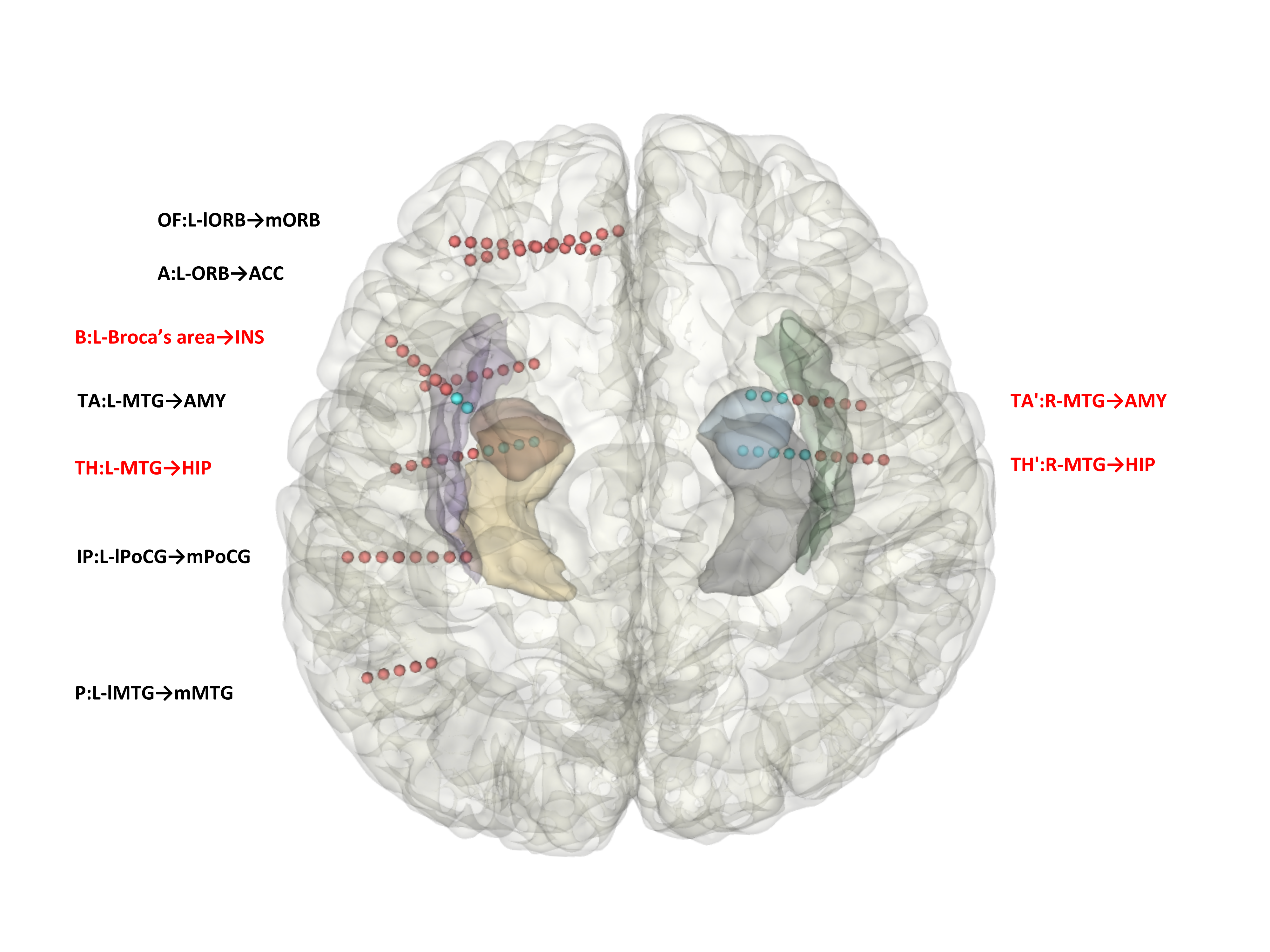
**

Fig. S7. No. 7 Patient’s 3D electrodes (contacts in targeted brain areas/total contacts). 7 depth electrodes in the left brain and 2 depth electrodes in the right brain in subject No. 7. The electrode marked in blue font indicates that it is in the targeted brain area.

OF, L-lORB→mORB, left-lateral orbital frontal cortex→medial orbital frontal cortex (0/10);

A, L-ORB→ACC, left-orbital frontal cortex→anterior cingulate cortex (0/10);

B, L-Broca's area→INS, left-Broca's area→Insula (2/8);

TA, L-MTG→AMY, left-middle temporal gyrus→Amygdala (0/8). The electrode of TA missed in amygdala;

TH, L-MTG→HIP, left-middle temporal gyrus→Hippocampus (4/10);

IP, L-lPoCG→mPoCG, left-lateral postcentral gyrus→medial postcentral gyrus (0/8);

P, L-lMTG→mMTG, left- lateral middle temporal gyrus→medial middle temporal gyrus (0/5);

TA', R-MTG→AMY, right-middle temporal gyrus→Amygdala (3/8);

TH', R-MTG→HIP, right-middle temporal gyrus→Hippocampus (5/10).


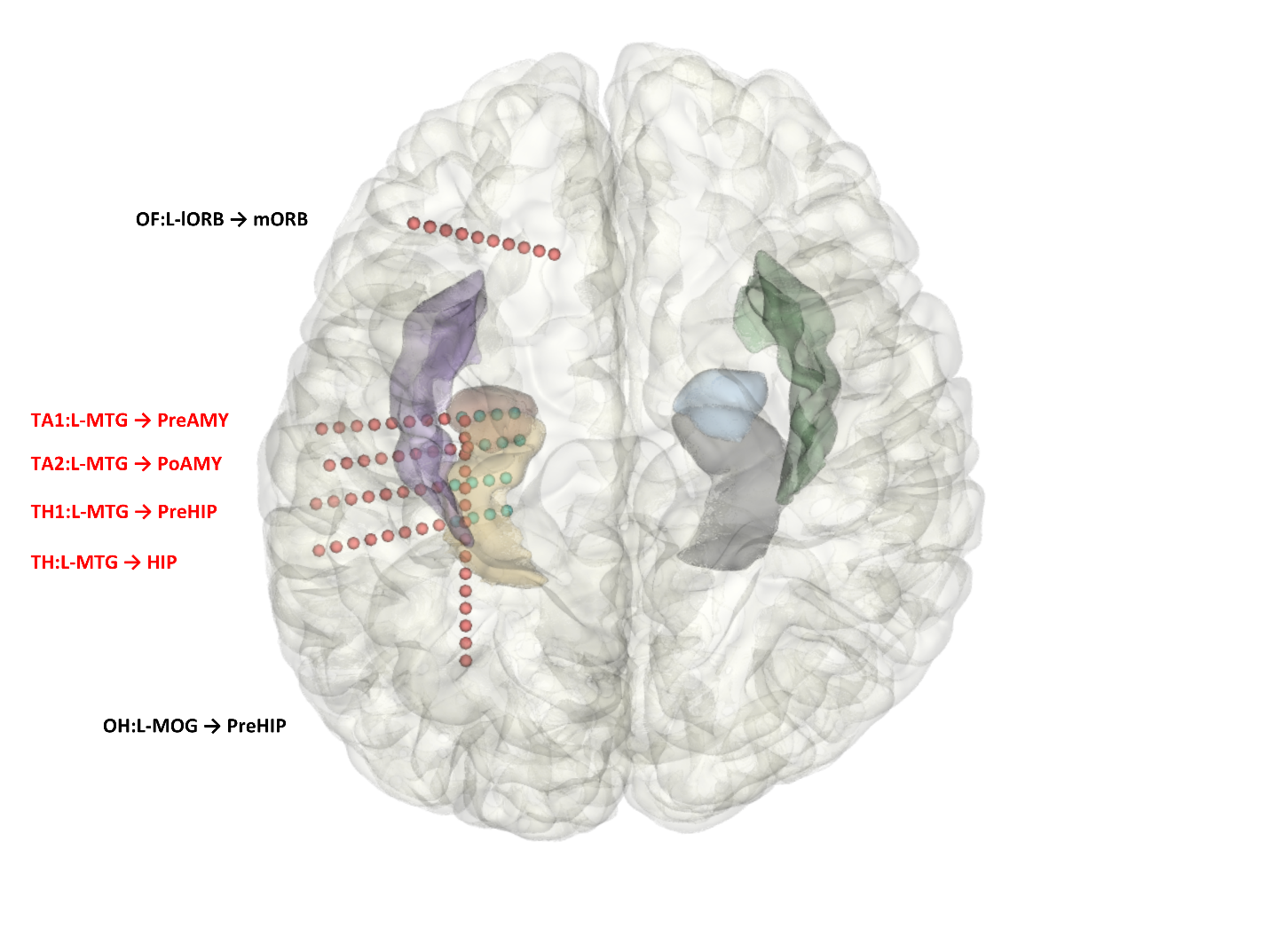


Fig. S8. No. 8 Patient’s 3D electrodes (contacts in targeted brain areas/total contacts). 6 depth electrodes in the left brain in subject No. 8. The electrode marked in blue font indicates that it is in the targeted brain area.

OF, L-lORB→mORB, left-lateral orbital frontal cortex→medial orbital frontal cortex (0/10);

TA1, L-MTG→PreAMY, left-middle temporal gyrus→pre-amygdala (4/12);

TA2, L-MTG→PoAMY, left-middle temporal gyrus→post-amygdala (3/12);

TH1, L-MTG→PreHIP, left-middle temporal gyrus→pre-hippocampus (4/12);

TH, L-MTG→HIP, left-middle temporal gyrus→Hippocampus (4/12);

OH, L-MOG→PreHIP, left-middle occipital gyrus→pre-hippocampus (0/15).

**
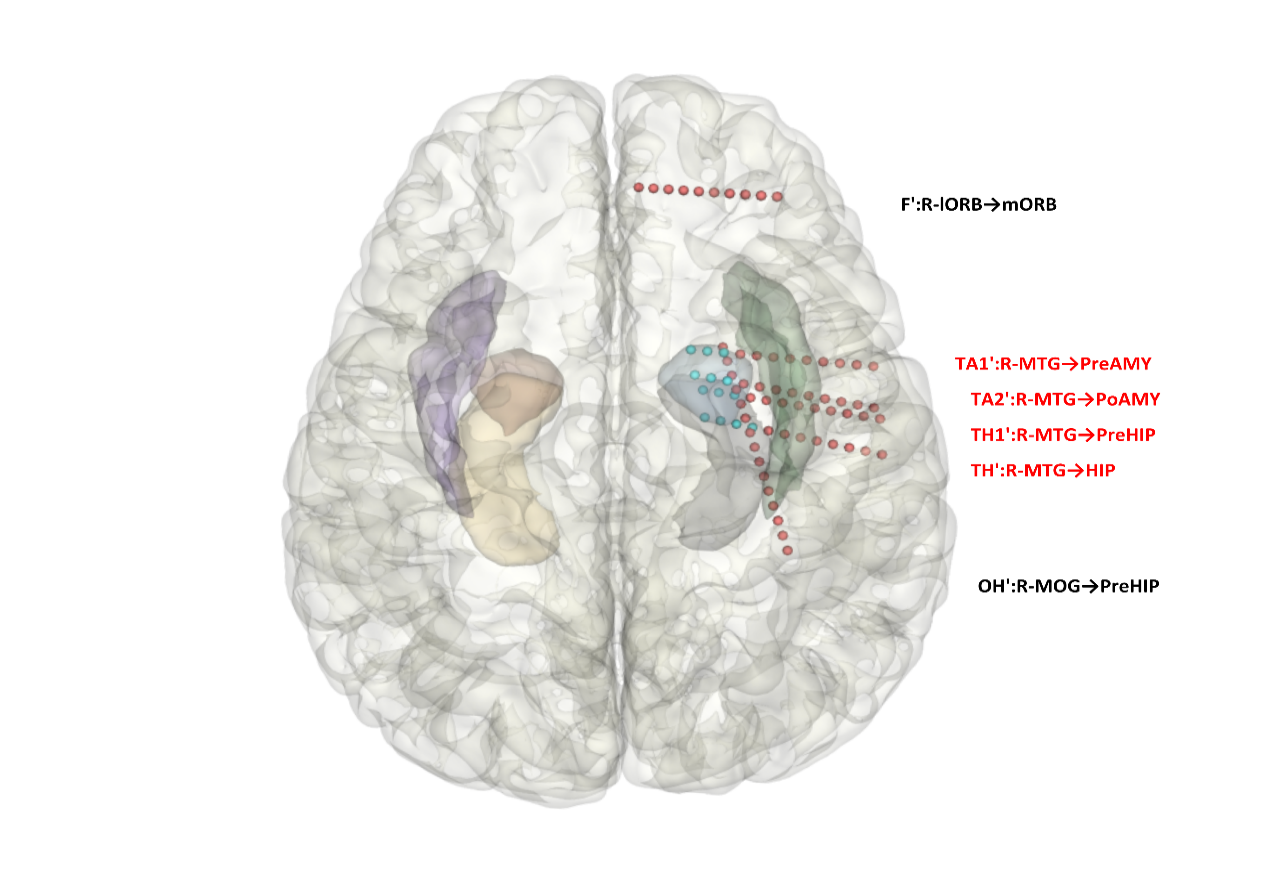
**

Fig. S9. No. 9 Patient’s 3D electrodes (contacts in targeted brain areas/total contacts). 6 depth electrodes in the right brain in subject No. 9. The electrode marked in blue font indicates that it is in the targeted brain area.

OF', R-lORB→mORB, right-lateral orbital frontal cortex→medial orbital frontal cortex (0/10);

TA1', R-MTG→PreAMY, right-middle temporal gyrus→pre-amygdala (3/12);

TA2', R-MTG→PoAMY, right-middle temporal gyrus→post-amygdala (3/12);

TH1', R-MTG→PreHIP, right-middle temporal gyrus→pre-hippocampus (3/12);

TH', R-MTG→HIP, right-middle temporal gyrus→Hippocampus (4/12);

OH', R-MOG→PreHIP, right-middle occipital gyrus→pre-hippocampus (0/15).

**
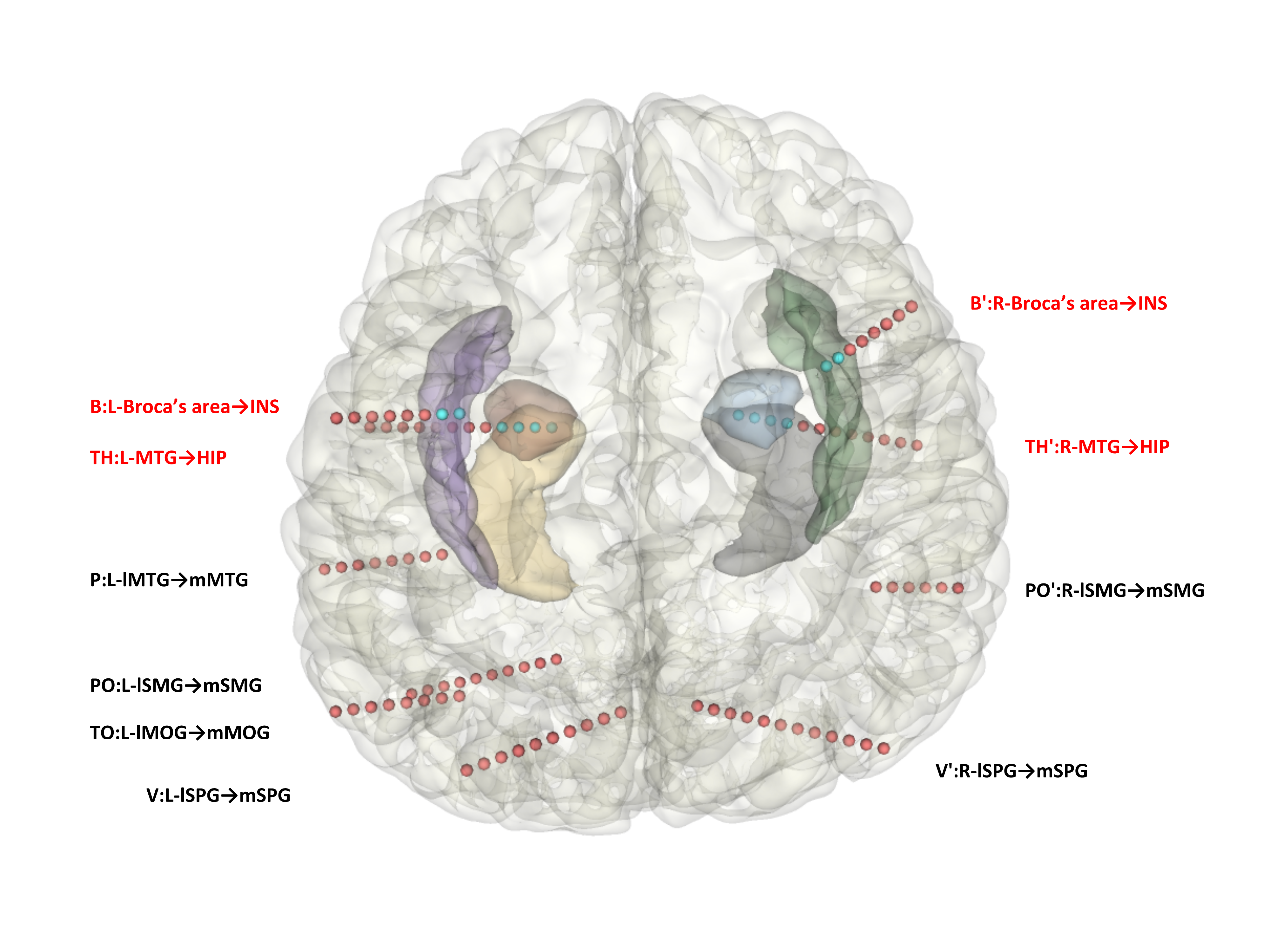
**

Fig. S10. No. 10 Patient’s 3D electrodes (contacts in targeted brain areas/total contacts). 6 depth electrodes in the left brain and 4 depth electrodes in the right brain in subject No. 10. (The electrode marked in blue font indicates that it is in the targeted brain area.)

B, L-Broca’s area→INS, left-Broca’s area→Insula (2/8);

TH, L-MTG→HIP, left-middle temporal gyrus→Hippocampus (4/12);

P, L-lMTG→mMTG, left-lateral middle temporal gyrus→medial middle temporal gyrus (0/8);

PO, L-lSMG→mSMG, left-lateral supramarginal gyrus→medial supramarginal gyrus (0/8);

TO, L-lMOG→mMOG, left-lateral middle occipital gyrus→medial middle occipital gyrus (0/10);

V, L-lSPG→mSPG, left-lateral superior parietal gyrus→medial superior parietal gyrus (0/10);

B', R-Broca's area→INS, right-Broca's area→Insula (2/8);

TH', R-MTG→HIP, right-middle temporal gyrus→Hippocampus (4/12);

PO', R-lSMG→mSMG, right-lateral supramarginal gyrus→medial supramarginal gyrus (0/6);

V', R-lSPG→mSPG, right-lateral superior parietal gyrus→medial superior parietal gyrus (0/12).


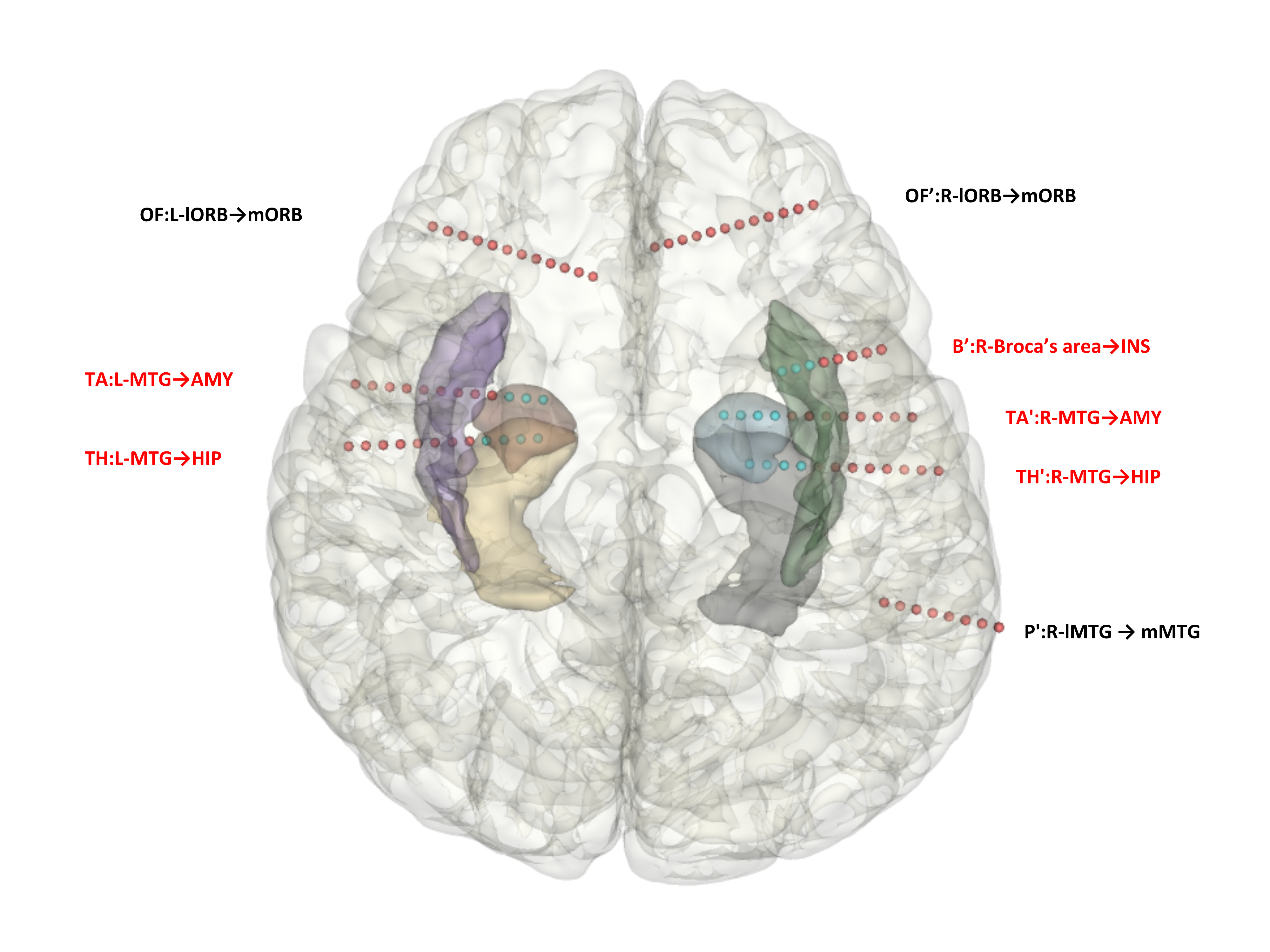


Fig. S11. No. 11 Patient’s 3D electrodes (contacts in targeted brain areas/total contacts). 3 depth electrodes in the left brain and 5 depth electrodes in the right brain in subject No. 11. The electrode marked in blue font indicates that it is in the targeted brain area.

OF, L-lORB→mORB, left-lateral orbital frontal cortex→medial orbital frontal cortex (0/12);

TA, L-MTG→AMY, left-middle temporal gyrus→Amygdala (3/12);

TH, L-MTG→HIP, left-middle temporal gyrus→Hippocampus (4/12);

OF', R-lORB→mORB, right-lateral orbital frontal cortex→medial orbital frontal cortex (0/12);

B', R-Broca's area→INS, right-Broca's area→Insula (3/8);

TA', R-MTG→AMY, right-middle temporal gyrus→Amygdala (4/12);

TH', R-MTG→HIP, right-middle temporal gyrus→Hippocampus (4/12);

P', R-lMTG→mMTG, right-lateral middle temporal gyrus→medial middle temporal gyrus (0/8).

**
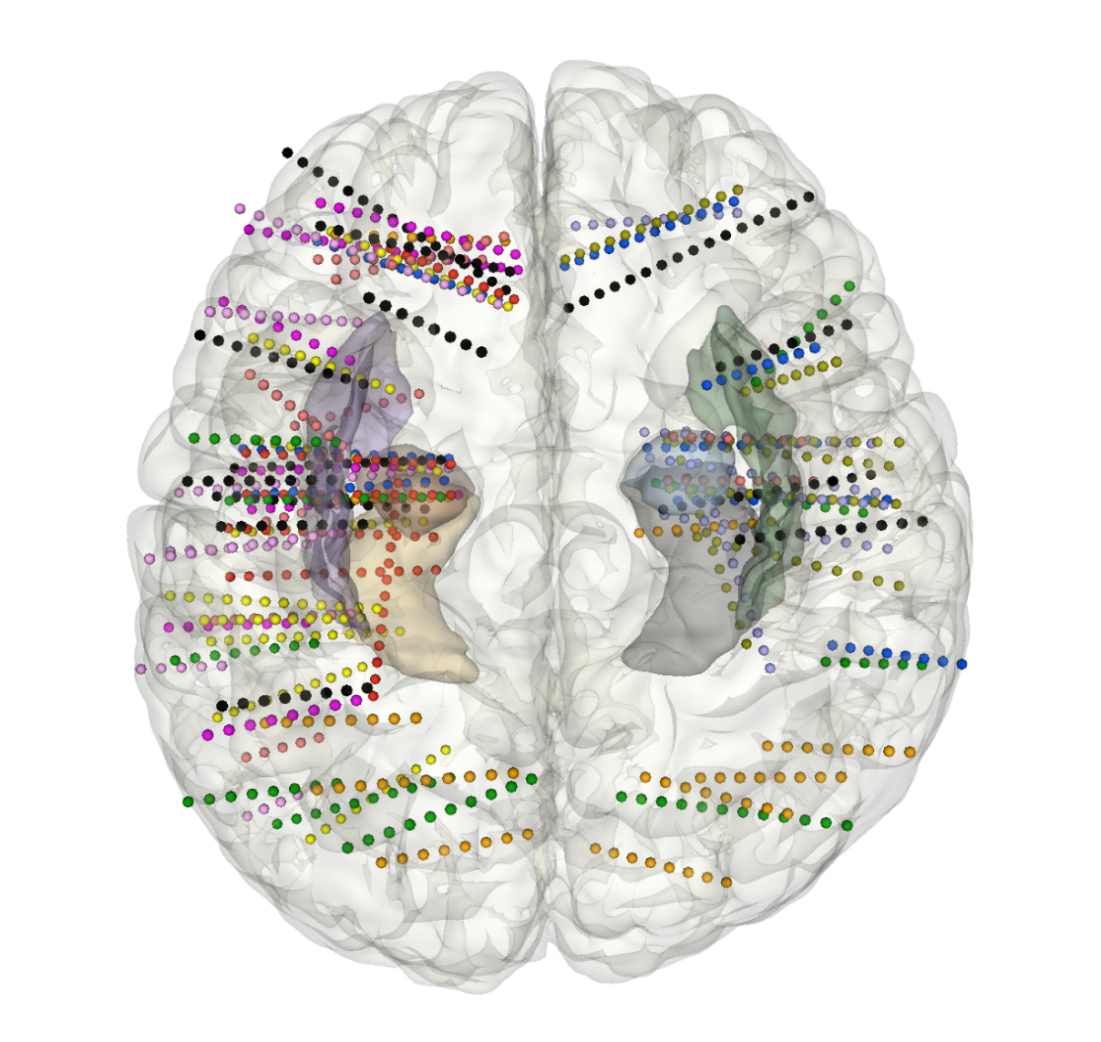
**

Fig. S12. Reconstruction of depth electrodes in an “average brain”. The images show the reconstruction of depth electrodes into the brain of all 11 patients. The different highlighted colors represented the left and right hippocampus, insula, and amygdala (the yellow and grey for left and right hippocampus, respectively; the purple and green for left and right insula, respectively; the brown and blue for left and right amygdala, respectively).

**
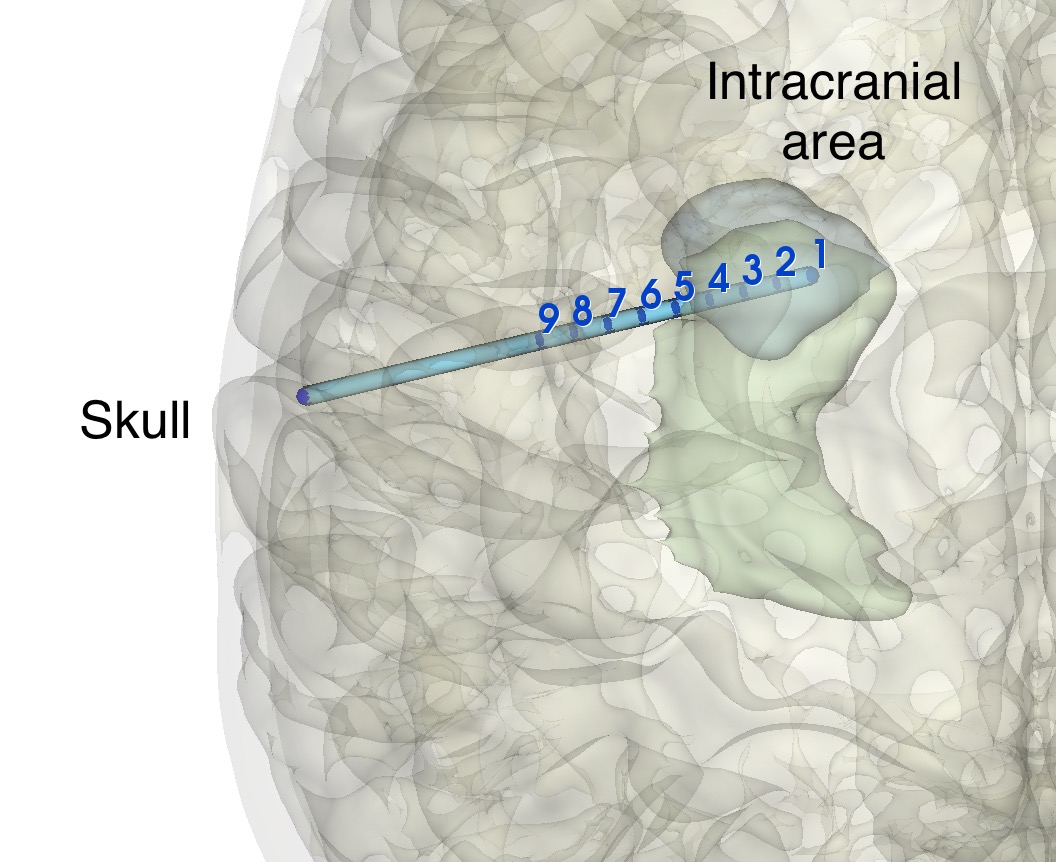
**

Fig. S13. The schematic diagram of SEEG electrode contact. The numbering convention on the contact is that the deepest contact is named contact "1", and the most superficial contact of each electrode has the highest number.


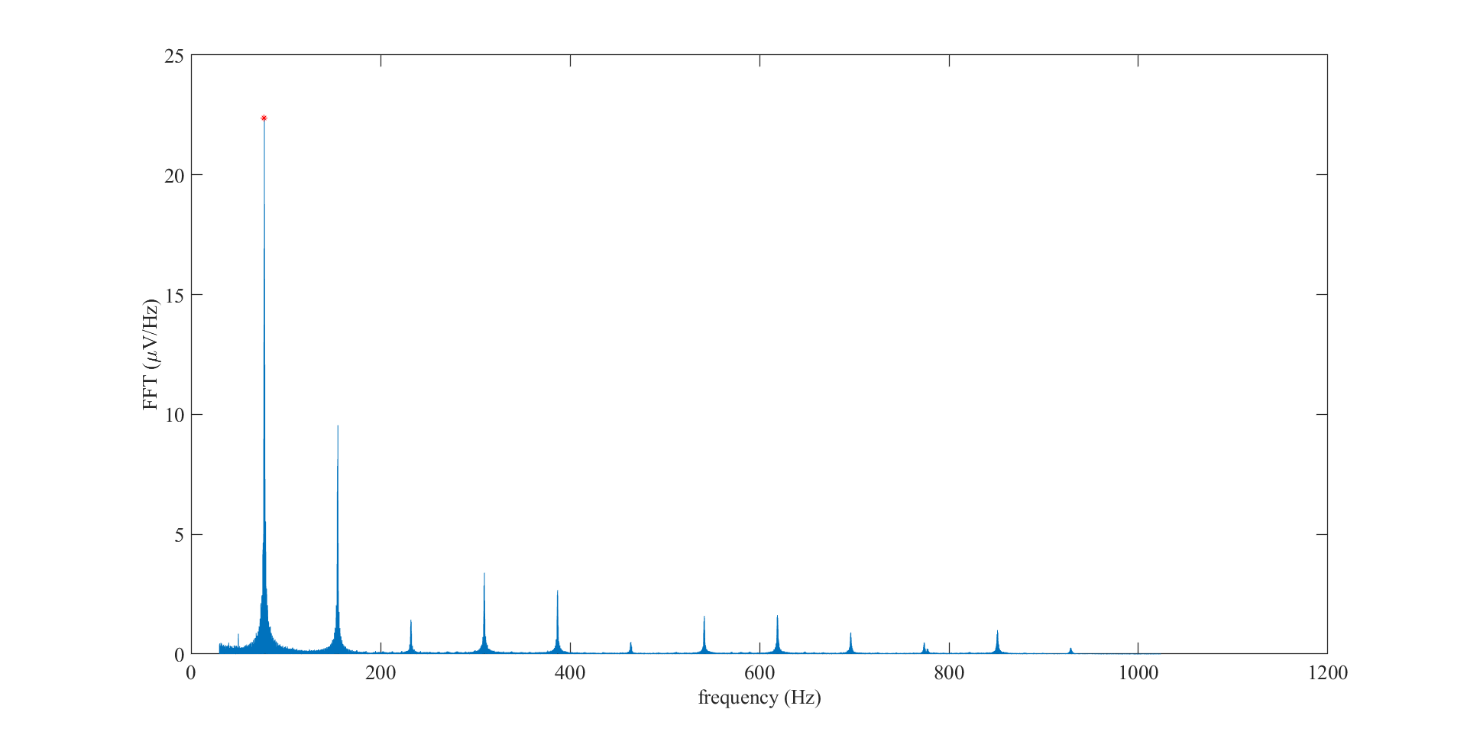


**Fig. S14.** An example of the frequency spectrum analysis process on SEEG raw data for 1^st^ contact of TH (left middle temporal gyrus→hippocampus) of Subject No. 4.

Note: *77.41 Hz; FFT, Fast Fourier Transform.

The single-sided frequency spectrum of SEEG raw data at the 1^st^ contact (i.e. the deepest contact) of TH (left middle temporal gyrus→hippocampus) of Subject No. 4 showed that the waveforms of stimulating currents was a 77.5 Hz square wave, which was equal to the sum of a series of sinusoidal waves with a fundamental frequency of 77.5 Hz and higher harmonics with frequencies that were multiples of 77.5 Hz. Because the sampling frequency of the SEEG recording system was 2,048 Hz, the maximum frequency component in the recorded signals should not exceed half of the sampling frequency according to the Nyquist sampling theorem. The frequency components were all less than 1,024 Hz. The peaks in **Fig. S14.** appeared at frequencies equal to multiples of 77.41 Hz. The frequency spectrum analysis demonstrated that the primary frequency component of the SEEG raw data was very close to 77.5 HZ.

A bandpass filter with a narrow pass band of 70-85 Hz was used to remove as much noise as possible, so the higher frequency components caused by the stimulating current were also removed. **Fig. S14.** showed that the peak amplitude of the frequency spectrum near 77.5 Hz was more than twice the amplitude of the frequency spectrum at the other extreme value points.

By analyzing the frequency spectrum of the raw signal at each electrode contact using Fast Fourier Transform before the 70-85 Hz bandpass filter, the maximum frequency components of the other active contacts were appeared in the range from 77.24 to 77.58 Hz, except for 15 contacts in 3 subjects appeared to be far from 77.5 Hz due to the low signal-to-noise ratio *[the 1^st^, 2^nd^, 3^rd^, 4^th^, 5^th^, 6^th^, 9^th^, 10^th^, and 11^th^ of TA (left middle temporal gyrus→amygdala) of Subject No.5, the 8^th^, 9^th^, and 10^th^ contact of OF (left lateral orbital frontal cortex→medial orbital frontal cortex) of Subject No.7, the 1^st^, 5^th^, and 6^th^ of TA (left middle temporal gyrus→amygdala) of Subject No.11]*. After a bandpass filter at 70-85 Hz, only 3 contacts (the 1^st^, 5^th^, and 6^th^) of TA (left middle temporal gyrus→amygdala) in Subject No.7 showed that their maximum frequency spectrums on the peak amplitudes were still far from 77.5 Hz, and the maximum frequency components changed to 77.5 Hz after bandpass filtering at 70-85 Hz for the above-mentioned contacts in Subjects No.5 and No.11. These results suggested that the data for these electrode contacts in Subject No.7 were unreliable (see **Table S3. Primary frequency component at pre & post the 70-85 Hz bandpass filter**).


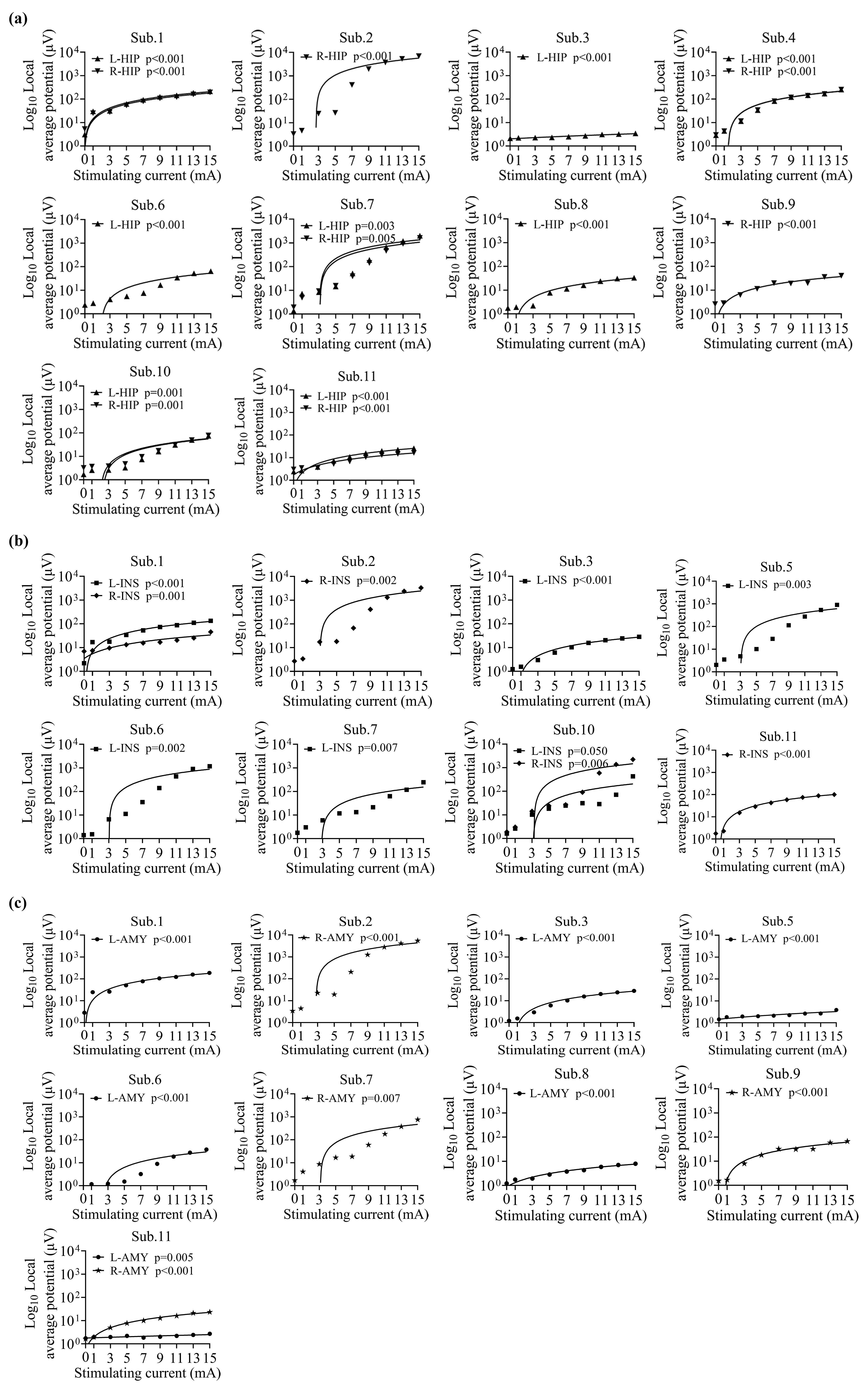


**Fig. S15.** The changes of average local field potentials in each subject's hippocampus, insula, and amygdala. **(a)** left and right hippocampus. **(b)** left and right insula. **(c)** left and right amygdala. Significant correlations on the average local field potentials changes with the stepwise increase of the extracranial alternating currents were found in each subject's left and right hippocampus (all *p* < 0.01), left and right insula (*p* < 0.01), except for the left insula of subject No.10 (*p* > 0.05), and left and right amygdala (all *p* < 0.01). AMY, Amygdala; HIP, hippocampus; INS, insula; L, left; R, right; Sub., subject.

Table S1. Details on electrode contacts

| **Sub.No.** | **side** | **electrode** | **trajectories of depth electrodes** | **details on electrode trajectories** | **contacts** | **contacts in targets** |
| --- | --- | --- | --- | --- | --- | --- |
| 1 | L | OF | L-lORB→mORB | left-lateral orbital frontal cortex→medial orbital frontal cortex | 16 | 0 |
| 1 | L | A | L-ORB→ACC | left-orbital frontal cortex→anterior cingulate cortex | 12 | 0 |
| 1 | L | B | L-Broca’s area→INS | left-Broca’s area→Insula | 10 | 2 |
| 1 | L | TA | L-MTG→AMY | left-middle temporal gyrus→Amygdala | 12 | 4 |
| 1 | L | SM | L-lSMA→mSMA | left-lateral supplementary motor area→medial supplementary motor area | 8 | 0 |
| 1 | L | TH | L-MTG→HIP | left-middle temporal gyrus→Hippocampus | 12 | 3 |
| 1 | L | PO | L-lSMG→mSMG | left-lateral supramarginal gyrus→medial supramarginal gyrus | 8 | 0 |
| 1 | L | IP | L-lPoCG→mPoCG | left-lateral postcentral gyrus→medial postcentral gyrus | 8 | 0 |
| 1 | L | P | L-lMTG→mMTG | left- lateral middle temporal gyrus→medial middle temporal gyrus | 8 | 0 |
| 1 | R | OF' | R-lORB→mORB | right-lateral orbital frontal cortex→medial orbital frontal cortex | 16 | 0 |
| 1 | R | B' | R-Broca’s area→INS | right-Broca’s area→Insula | 8 | 2 |
| 1 | R | TH' | R-MTG→HIP | right-middle temporal gyrus→Hippocampus | 12 | 4 |
| 1 | R | IP' | R-lPoCG→mPoCG | right-lateral postcentral gyrus→medial postcentral gyrus | 10 | 0 |
| 2 | R | OF' | R-lORB→mORB | right-lateral orbital frontal cortex→medial orbital frontal cortex | 12 | 0 |
| 2 | R | B' | R-Broca’s area→INS | right-Broca’s area→Insula | 8 | 2 |
| 2 | R | TA' | R-MTG→AMY | right-middle temporal gyrus→Amygdala | 12 | 3 |
| 2 | R | TH1' | R-MTG→PreHIP | right-middle temporal gyrus→pre-hippocampus | 12 | Miss |
| 2 | R | TH' | R-MTG→HIP | right-middle temporal gyrus→Hippocampus | 12 | 4 |
| 2 | R | P' | R-lMTG→mMTG | right-lateral middle temporal gyrus→medial middle temporal gyrus | 12 | 0 |
| 2 | R | OH' | R-MOG→PreHIP | right-middle occipital gyrus→pre-hippocampus | 12 | 0 |
| 3 | L | OF | L-lORB→mORB | left-lateral orbital frontal cortex→medial orbital frontal cortex | 16 | 0 |
| 3 | L | A | L-ORB→ACC | left-orbital frontal cortex→anterior cingulate cortex | 12 | 0 |
| 3 | L | B | L-Broca’s area→INS | left-Broca’s area→Insula | 8 | 2 |
| 3 | L | TA | L-MTG→AMY | left-middle temporal gyrus→Amygdala | 12 | 4 |
| 3 | L | TH | L-MTG→HIP | left-middle temporal gyrus→Hippocampus | 12 | 5 |
| 3 | L | P | L-lMTG→mMTG | left- lateral middle temporal gyrus→medial middle temporal gyrus | 8 | 0 |
| 3 | L | PO | L-lSMG→mSMG | left-lateral supramarginal gyrus→medial supramarginal gyrus | 8 | 0 |
| 4 | L | A | L-ORB→ACC | left-orbital frontal cortex→anterior cingulate cortex | 10 | 0 |
| 4 | L | TH | L-MTG→HIP | left-middle temporal gyrus→Hippocampus | 8 | 4 |
| 4 | L | PO1 | L-lPreSMG→mPreSMG | left-lateral pre-supramarginal gyrus→medial pre-supramarginal gyrus | 8 | 0 |
| 4 | L | PO2 | L-lPoSMG→mPoSMG | left-lateral post-supramarginal gyrus→medial post-supramarginal gyrus | 10 | 0 |
| 4 | L | P | L-lMTG→mMTG | left- lateral middle temporal gyrus→medial middle temporal gyrus | 8 | 0 |
| 4 | R | TH' | R-MTG→HIP | right-middle temporal gyrus→Hippocampus | 8 | 5 (of them, 1 was broken) |
| 4 | R | PO1' | R-lPreSMG→mPreSMG | right-lateral pre-supramarginal gyrus→medial pre-supramarginal gyrus | 8 | 0 |
| 4 | R | PO2' | R-lPoSMG→mPoSMG | right-lateral post-supramarginal gyrus→medial post-supramarginal gyrus | 8 | 0 |
| 4 | R | P1' | R-lPreMTG→mPreMTG | right-lateral pre-middle temporal gyrus→medial pre-middle temporal gyrus | 8 | 0 |
| 4 | R | P2' | R-lPoMTG→mPoMTG | right-lateral post-middle temporal gyrus→medial post-middle temporal gyrus | 8 | 0 |
| 5 | L | A | L-ORB→ACC | left-orbital frontal cortex→anterior cingulate cortex | 15 | 0 |
| 5 | L | B | L-Broca’s area→INS | left-Broca’s area→Insula | 10 | 2 |
| 5 | L | TA | L-MTG→AMY | left-middle temporal gyrus→Amygdala | 15 | 4 |
| 5 | L | C | L-PreCG→INS | left-precentral gyrus→Insula | 10 | 2 |
| 5 | L | P1 | L-lPreMTG→mPreMTG | left-lateral pre-middle temporal gyrus→medial pre-middle temporal gyrus | 8 | 0 |
| 5 | L | P2 | L-lPoMTG→mPoMTG | left-lateral post-middle temporal gyrus→medial post-middle temporal gyrus | 5 | 0 |
| 5 | L | FF | L-lFFG→mFFG | left-lateral fusiform gyrus→medial fusiform gyrus | 5 | 0 |
| 6 | L | OF | L-lORB→mORB | left-lateral orbital frontal cortex→medial orbital frontal cortex | 12 | 0 |
| 6 | L | B | L-Broca’s area→INS | left-Broca’s area→Insula | 10 | 5 |
| 6 | L | TA | L-MTG→AMY | left-middle temporal gyrus→Amygdala | 12 | 3 |
| 6 | L | TH | L-MTG→HIP | left-middle temporal gyrus→Hippocampus | 12 | 4 |
| 6 | L | PO | L-lSMG→mSMG | left-lateral supramarginal gyrus→medial supramarginal gyrus | 10 | 0 |
| 6 | L | W | L-lSTG→mSTG | left-lateral superior temporal gyrus→medial superior temporal gyrus | 12 | 0 |
| 6 | L | FF | L-lFFG→mFFG | left-lateral fusiform gyrus→medial fusiform gyrus | 10 | 0 |
| 6 | L | P | L-lMTG→mMTG | left- lateral middle temporal gyrus→medial middle temporal gyrus | 12 | 0 |
| 6 | L | O | L-lTPO→mTPO | left-lateral temporal occipital junction→medial temporal occipital junction | 10 | 0 |
| 7 | L | OF | L-lORB→mORB | left-lateral orbital frontal cortex→medial orbital frontal cortex | 10 | 0 |
| 7 | L | A | L-ORB→ACC | left-orbital frontal cortex→anterior cingulate cortex | 10 | 0 |
| 7 | L | B | L-Broca’s area→INS | left-Broca’s area→Insula | 8 | 2 |
| 7 | L | TA | L-MTG→AMY | left-middle temporal gyrus→Amygdala | 8 | Miss |
| 7 | L | TH | L-MTG→HIP | left-middle temporal gyrus→Hippocampus | 10 | 4 |
| 7 | L | IP | L-lPoCG→mPoCG | left-lateral postcentral gyrus→medial postcentral gyrus | 8 | 0 |
| 7 | L | P | L-lMTG→mMTG | left- lateral middle temporal gyrus→medial middle temporal gyrus | 5 | 0 |
| 7 | R | TA' | R-MTG→AMY | right-middle temporal gyrus→Amygdala | 8 | 3 |
| 7 | R | TH' | R-MTG→HIP | right-middle temporal gyrus→Hippocampus | 10 | 5 |
| 8 | L | OF | L-lORB→mORB | left-lateral orbital frontal cortex→medial orbital frontal cortex | 10 | 0 |
| 8 | L | TA1 | L-MTG→PreAMY | left-middle temporal gyrus→pre-amygdala | 12 | 4 |
| 8 | L | TA2 | L-MTG→PoAMY | left-middle temporal gyrus→post-amygdala | 12 | 3 |
| 8 | L | TH1 | L-MTG→PreHIP | left-middle temporal gyrus→pre-hippocampus | 12 | 4 |
| 8 | L | TH | L-MTG→HIP | left-middle temporal gyrus→Hippocampus | 12 | 4 |
| 8 | L | OH | L-MOG→PreHIP | left-middle occipital gyrus→pre-hippocampus | 15 | 0 |
| 9 | R | OF' | R-lORB→mORB | right-lateral orbital frontal cortex→medial orbital frontal cortex | 10 | 0 |
| 9 | R | TA1' | R-MTG→PreAMY | right-middle temporal gyrus→pre-amygdala | 12 | 3 |
| 9 | R | TA2' | R-MTG→PoAMY | right-middle temporal gyrus→post-amygdala | 12 | 3 |
| 9 | R | TH1' | R-MTG→PreHIP | right-middle temporal gyrus→pre-hippocampus | 12 | 3 |
| 9 | R | TH' | R-MTG→HIP | right-middle temporal gyrus→Hippocampus | 12 | 4 |
| 9 | R | OH' | R-MOG→PreHIP | right-middle occipital gyrus→pre-hippocampus | 15 | 0 |
| 10 | L | B | L-Broca’s area→INS | left-Broca’s area→Insula | 8 | 2 |
| 10 | L | TH | L-MTG→HIP | left-middle temporal gyrus→Hippocampus | 12 | 4 |
| 10 | L | P | L-lMTG→mMTG | left- lateral middle temporal gyrus→medial middle temporal gyrus | 8 | 0 |
| 10 | L | PO | L-lSMG→mSMG | left-lateral supramarginal gyrus→medial supramarginal gyrus | 8 | 0 |
| 10 | L | TO | L-lMOG→mMOG | left-lateral middle occipital gyrus→medial middle occipital gyrus | 10 | 0 |
| 10 | L | V | L-lSPG→mSPG | left-lateral superior parietal gyrus→medial superior parietal gyrus | 10 | 0 |
| 10 | R | B' | R-Broca’s area→INS | right-Broca’s area→Insula | 8 | 2 |
| 10 | R | TH' | R-MTG→HIP | right-middle temporal gyrus→Hippocampus | 12 | 4 |
| 10 | R | PO' | R-lSMG→mSMG | right-lateral supramarginal gyrus→medial supramarginal gyrus | 6 | 0 |
| 10 | R | V' | R-lSPG→mSPG | right-lateral superior parietal gyrus→medial superior parietal gyrus | 12 | 0 |
| 11 | L | OF | L-lORB→mORB | left-lateral orbital frontal cortex→medial orbital frontal cortex | 12 | 0 |
| 11 | L | TA | L-MTG→AMY | left-middle temporal gyrus→Amygdala | 12 | 3 |
| 11 | L | TH | L-MTG→HIP | left-middle temporal gyrus→Hippocampus | 12 | 4 |
| 11 | R | OF' | R-lORB→mORB | right-lateral orbital frontal cortex→medial orbital frontal cortex | 12 | 0 |
| 11 | R | B' | R-Broca’s area→INS | right-Broca’s area→Insula | 8 | 3 |
| 11 | R | TA' | R-MTG→AMY | right-middle temporal gyrus→Amygdala | 12 | 4 |
| 11 | R | TH' | R-MTG→HIP | right-middle temporal gyrus→Hippocampus | 12 | 4 |
| 11 | R | P' | R-lMTG→mMTG | right-lateral middle temporal gyrus→medial middle temporal gyrus | 8 | 0 |
| total | | | | | 953 | 136 (of them, 1 contact was broken) |

Table S2. Comparison on epileptic spikes between the pre- and post- tACS intervention in 11 patients

| **Patient No.** | **1** | **2** | **3** | **4** | **5** | **6** | **7** | **8** | **9** | **10** | **11** | **12** | **13** |
| --- | --- | --- | --- | --- | --- | --- | --- | --- | --- | --- | --- | --- | --- |
| **Epileptic spikes**  **at pre-tACS** | 25 | 2 | 101 | 2 | 11 | 14 | 33 | 87 | 3 | 23 | 19 | 8 | 24 |
| **Epileptic spikes**  **at post-tACS** | 26 | 2 | 77 | 2 | 5 | 16 | 19 | 132 | 4 | 28 | 18 | 6 | 45 |

We selected EEG data from 10 minutes before and after the tACS stimulation for epileptic spike counting, and there was no significant different on epileptic spikes between the pre- and post-tACS in 11 patients revealed by paired T test (*p* = 0.645).

**Table S3.** Primary frequency component at pre & post-the 70-85 Hz bandpass filter

| **patient number** | **contact** | **primary frequency component (Hz)** | | **note** |
| --- | --- | --- | --- | --- |
|  |  | **raw data/ pre-the 70-85 Hz bandpass filter** | **post-the 70-85Hz bandpass filter** |  |
| 1 | OF-1 | 77.41555023 | 77.41555023 |  |
| 1 | OF-2 | 77.41555023 | 77.41555023 |  |
| 1 | OF-3 | 77.41555023 | 77.41555023 |  |
| 1 | OF-4 | 77.41555023 | 77.41555023 |  |
| 1 | OF-5 | 77.41555023 | 77.41555023 |  |
| 1 | OF-6 | 77.41555023 | 77.41555023 |  |
| 1 | OF-7 | 77.41555023 | 77.41555023 |  |
| 1 | OF-8 | 77.41555023 | 77.41555023 |  |
| 1 | OF-9 | 77.41555023 | 77.41555023 |  |
| 1 | OF-10 | 77.41191864 | 77.41191864 |  |
| 1 | OF-11 | 77.41191864 | 77.41191864 |  |
| 1 | OF-12 | 77.41191864 | 77.41191864 |  |
| 1 | OF-13 | 77.41191864 | 77.41191864 |  |
| 1 | OF-14 | 77.41191864 | 77.41191864 |  |
| 1 | OF-15 | 77.41191864 | 77.41191864 |  |
| 1 | OF-16 | 77.24953461 | 77.24953461 |  |
| 1 | A-1 | 77.41555023 | 77.41555023 |  |
| 1 | A-2 | 77.41555023 | 77.41555023 |  |
| 1 | A-3 | 77.41555023 | 77.41555023 |  |
| 1 | A-4 | 77.41555023 | 77.41555023 |  |
| 1 | A-5 | 77.41555023 | 77.41555023 |  |
| 1 | A-6 | 77.41555023 | 77.41555023 |  |
| 1 | A-7 | 77.41555023 | 77.41555023 |  |
| 1 | A-8 | 77.41555023 | 77.41555023 |  |
| 1 | A-9 | 77.41555023 | 77.41555023 |  |
| 1 | A-10 | 77.41555023 | 77.41555023 |  |
| 1 | A-11 | 77.41555023 | 77.41555023 |  |
| 1 | A-12 | 77.41555023 | 77.41555023 |  |
| 1 | B-1 | 77.58267212 | 77.58267212 |  |
| 1 | B-2 | 77.58267212 | 77.58267212 |  |
| 1 | B-3 | 77.58267212 | 77.58267212 |  |
| 1 | B-4 | 77.58267212 | 77.58267212 |  |
| 1 | B-5 | 77.58267212 | 77.58267212 |  |
| 1 | B-6 | 77.58267212 | 77.58267212 |  |
| 1 | B-7 | 77.58267212 | 77.58267212 |  |
| 1 | B-8 | 77.58267212 | 77.58267212 |  |
| 1 | B-9 | 77.58267212 | 77.58267212 |  |
| 1 | B-10 | 77.58267212 | 77.58267212 |  |
| 1 | TA-1 | 77.58267212 | 77.58267212 |  |
| 1 | TA-2 | 77.58267212 | 77.58267212 |  |
| 1 | TA-3 | 77.58267212 | 77.58267212 |  |
| 1 | TA-4 | 77.58267212 | 77.58267212 |  |
| 1 | TA-5 | 77.58267212 | 77.58267212 |  |
| 1 | TA-6 | 77.58267212 | 77.58267212 |  |
| 1 | TA-7 | 77.58267212 | 77.58267212 |  |
| 1 | TA-8 | 77.58267212 | 77.58267212 |  |
| 1 | TA-9 | 77.58267212 | 77.58267212 |  |
| 1 | TA-10 | 77.58267212 | 77.58267212 |  |
| 1 | TA-11 | 77.58267212 | 77.58267212 |  |
| 1 | TA-12 | 77.58267212 | 77.58267212 |  |
| 1 | SM-1 | 77.41555023 | 77.41555023 |  |
| 1 | SM-2 | 77.41555023 | 77.41555023 |  |
| 1 | SM-3 | 77.41555023 | 77.41555023 |  |
| 1 | SM-4 | 77.41555023 | 77.41555023 |  |
| 1 | SM-5 | 77.41555023 | 77.41555023 |  |
| 1 | SM-6 | 77.41555023 | 77.41555023 |  |
| 1 | SM-7 | 77.41555023 | 77.41555023 |  |
| 1 | SM-8 | 77.41555023 | 77.41555023 |  |
| 1 | TH-1 | 77.58267212 | 77.58267212 |  |
| 1 | TH-2 | 77.58267212 | 77.58267212 |  |
| 1 | TH-3 | 77.58267212 | 77.58267212 |  |
| 1 | TH-4 | 77.58267212 | 77.58267212 |  |
| 1 | TH-5 | 77.58267212 | 77.58267212 |  |
| 1 | TH-6 | 77.58267212 | 77.58267212 |  |
| 1 | TH-7 | 77.58267212 | 77.58267212 |  |
| 1 | TH-8 | 77.58267212 | 77.58267212 |  |
| 1 | TH-9 | 77.58267212 | 77.58267212 |  |
| 1 | TH-10 | 77.58267212 | 77.58267212 |  |
| 1 | TH-11 | 77.58267212 | 77.58267212 |  |
| 1 | TH-12 | 77.58267212 | 77.58267212 |  |
| 1 | PO-1 | 77.58267212 | 77.58267212 |  |
| 1 | PO-2 | 77.58267212 | 77.58267212 |  |
| 1 | PO-3 | 77.58267212 | 77.58267212 |  |
| 1 | PO-4 | 77.58267212 | 77.58267212 |  |
| 1 | PO-5 | 77.58267212 | 77.58267212 |  |
| 1 | PO-6 | 77.58267212 | 77.58267212 |  |
| 1 | PO-7 | 77.58267212 | 77.58267212 |  |
| 1 | PO-8 | 77.58267212 | 77.58267212 |  |
| 1 | IP-1 | 77.58267212 | 77.58267212 |  |
| 1 | IP-2 | 77.58267212 | 77.58267212 |  |
| 1 | IP-3 | 77.58267212 | 77.58267212 |  |
| 1 | IP-4 | 77.58267212 | 77.58267212 |  |
| 1 | IP-5 | 77.58267212 | 77.58267212 |  |
| 1 | IP-6 | 77.58267212 | 77.58267212 |  |
| 1 | IP-7 | 77.58267212 | 77.58267212 |  |
| 1 | IP-8 | 77.58267212 | 77.58267212 |  |
| 1 | P-1 | 77.58267212 | 77.58267212 |  |
| 1 | P-2 | 77.58267212 | 77.58267212 |  |
| 1 | P-3 | 77.58267212 | 77.58267212 |  |
| 1 | P-4 | 77.58267212 | 77.58267212 |  |
| 1 | P-5 | 77.58267212 | 77.58267212 |  |
| 1 | P-6 | 77.58267212 | 77.58267212 |  |
| 1 | P-7 | 77.58267212 | 77.58267212 |  |
| 1 | P-8 | 77.58267212 | 77.58267212 |  |
| 1 | OF'-1 | 77.41555023 | 77.41555023 |  |
| 1 | OF'-2 | 77.41555023 | 77.41555023 |  |
| 1 | OF'-3 | 77.41555023 | 77.41555023 |  |
| 1 | OF'-4 | 77.41555023 | 77.41555023 |  |
| 1 | OF'-5 | 77.41555023 | 77.41555023 |  |
| 1 | OF'-6 | 77.41555023 | 77.41555023 |  |
| 1 | OF'-7 | 77.41555023 | 77.41555023 |  |
| 1 | OF'-8 | 77.41555023 | 77.41555023 |  |
| 1 | OF'-9 | 77.41555023 | 77.41555023 |  |
| 1 | OF'-10 | 77.41555023 | 77.41555023 |  |
| 1 | OF'-11 | 77.24702454 | 77.24702454 |  |
| 1 | OF'-12 | 77.58435059 | 77.58435059 |  |
| 1 | OF'-13 | 77.41191864 | 77.41191864 |  |
| 1 | OF'-14 | 77.41191864 | 77.41191864 |  |
| 1 | OF'-15 | 77.41191864 | 77.41191864 |  |
| 1 | OF'-16 | 77.41191864 | 77.41191864 |  |
| 1 | B'-1 | 77.58267212 | 77.24953461 |  |
| 1 | B'-2 | 77.58267212 | 77.24953461 |  |
| 1 | B'-3 | 77.58267212 | 77.41638184 |  |
| 1 | B'-4 | 77.58267212 | 77.58351135 |  |
| 1 | B'-5 | 77.58267212 | 77.58351135 |  |
| 1 | B'-6 | 77.58267212 | 77.58351135 |  |
| 1 | B'-7 | 77.58267212 | 77.58351135 |  |
| 1 | B'-8 | 77.58267212 | 77.58351135 |  |
| 1 | TH'-1 | 77.58267212 | 77.58267212 |  |
| 1 | TH'-2 | 77.58267212 | 77.58267212 |  |
| 1 | TH'-3 | 77.58267212 | 77.58267212 |  |
| 1 | TH'-4 | 77.58267212 | 77.58267212 |  |
| 1 | TH'-5 | 77.58267212 | 77.58267212 |  |
| 1 | TH'-6 | 77.58267212 | 77.58267212 |  |
| 1 | TH'-7 | 77.58267212 | 77.58267212 |  |
| 1 | TH'-8 | 77.58267212 | 77.58267212 |  |
| 1 | TH'-9 | 77.58267212 | 77.58267212 |  |
| 1 | TH'-10 | 77.58267212 | 77.58267212 |  |
| 1 | TH'-11 | 77.58267212 | 77.58267212 |  |
| 1 | TH'-12 | 77.58267212 | 77.58267212 |  |
| 1 | IP'-1 | 77.58267212 | 77.58267212 |  |
| 1 | IP'-2 | 77.58267212 | 77.58267212 |  |
| 1 | IP'-3 | 77.58267212 | 77.58267212 |  |
| 1 | IP'-4 | 77.58267212 | 77.58267212 |  |
| 1 | IP'-5 | 77.58267212 | 77.58267212 |  |
| 1 | IP'-6 | 77.58267212 | 77.58267212 |  |
| 1 | IP'-7 | 77.58267212 | 77.58267212 |  |
| 1 | IP'-8 | 77.58267212 | 77.58267212 |  |
| 1 | IP'-9 | 77.58267212 | 77.58267212 |  |
| 1 | IP'-10 | 77.58267212 | 77.58267212 |  |
| 2 | OF'-1 | 77.2452774 | 77.2452774 |  |
| 2 | OF'-2 | 77.2452774 | 77.2452774 |  |
| 2 | OF'-3 | 77.2452774 | 77.2452774 |  |
| 2 | OF'-4 | 77.2452774 | 77.2452774 |  |
| 2 | OF'-5 | 77.41166687 | 77.41166687 |  |
| 2 | OF'-6 | 77.24500275 | 77.24500275 |  |
| 2 | OF'-7 | 77.24500275 | 77.24500275 |  |
| 2 | OF'-8 | 77.58110809 | 77.58110809 |  |
| 2 | OF'-9 | 77.2452774 | 77.2452774 |  |
| 2 | OF'-10 | 77.2452774 | 77.2452774 |  |
| 2 | OF'-11 | 77.41194153 | 77.41194153 |  |
| 2 | OF'-12 | 77.41194153 | 77.41194153 |  |
| 2 | B'-1 | 77.2452774 | 77.2452774 |  |
| 2 | B'-2 | 77.2452774 | 77.2452774 |  |
| 2 | B'-3 | 77.2452774 | 77.2452774 |  |
| 2 | B'-4 | 77.41194153 | 77.41194153 |  |
| 2 | B'-5 | 77.2452774 | 77.2452774 |  |
| 2 | B'-6 | 77.2452774 | 77.2452774 |  |
| 2 | B'-7 | 77.2452774 | 77.2452774 |  |
| 2 | B'-8 | 77.2452774 | 77.2452774 |  |
| 2 | TA'-1 | 77.2452774 | 77.2452774 |  |
| 2 | TA'-2 | 77.2452774 | 77.2452774 |  |
| 2 | TA'-3 | 77.2452774 | 77.2452774 |  |
| 2 | TA'-4 | 77.2452774 | 77.2452774 |  |
| 2 | TA'-5 | 77.2452774 | 77.2452774 |  |
| 2 | TA'-6 | 77.2452774 | 77.2452774 |  |
| 2 | TA'-7 | 77.2452774 | 77.2452774 |  |
| 2 | TA'-8 | 77.2452774 | 77.2452774 |  |
| 2 | TA'-9 | 77.2452774 | 77.2452774 |  |
| 2 | TA'-10 | 77.2452774 | 77.2452774 |  |
| 2 | TA'-11 | 77.2452774 | 77.2452774 |  |
| 2 | TA'-12 | 77.2452774 | 77.2452774 |  |
| 2 | TH1'-1 | 77.2452774 | 77.2452774 |  |
| 2 | TH1'-2 | 77.2452774 | 77.2452774 |  |
| 2 | TH1'-3 | 77.2452774 | 77.2452774 |  |
| 2 | TH1'-4 | 77.2452774 | 77.2452774 |  |
| 2 | TH1'-5 | 77.2452774 | 77.2452774 |  |
| 2 | TH1'-6 | 77.2452774 | 77.2452774 |  |
| 2 | TH1'-7 | 77.2452774 | 77.2452774 |  |
| 2 | TH1'-8 | 77.2452774 | 77.2452774 |  |
| 2 | TH1'-9 | 77.2452774 | 77.2452774 |  |
| 2 | TH1'-10 | 77.2452774 | 77.2452774 |  |
| 2 | TH1'-11 | 77.2452774 | 77.2452774 |  |
| 2 | TH1'-12 | 77.2452774 | 77.2452774 |  |
| 2 | TH'-1 | 77.2452774 | 77.2452774 |  |
| 2 | TH'-2 | 77.2452774 | 77.2452774 |  |
| 2 | TH'-3 | 77.2452774 | 77.2452774 |  |
| 2 | TH'-4 | 77.2452774 | 77.2452774 |  |
| 2 | TH'-5 | 77.2452774 | 77.2452774 |  |
| 2 | TH'-6 | 77.2452774 | 77.2452774 |  |
| 2 | TH'-7 | 77.2452774 | 77.2452774 |  |
| 2 | TH'-8 | 77.2452774 | 77.2452774 |  |
| 2 | TH'-9 | 77.2452774 | 77.2452774 |  |
| 2 | TH'-10 | 77.2452774 | 77.2452774 |  |
| 2 | TH'-11 | 77.2452774 | 77.2452774 |  |
| 2 | TH'-12 | 77.2452774 | 77.2452774 |  |
| 2 | P'-1 | 77.2452774 | 77.2452774 |  |
| 2 | P'-2 | 77.2452774 | 77.2452774 |  |
| 2 | P'-3 | 77.2452774 | 77.2452774 |  |
| 2 | P'-4 | 77.2452774 | 77.2452774 |  |
| 2 | P'-5 | 77.2452774 | 77.2452774 |  |
| 2 | P'-6 | 77.2452774 | 77.2452774 |  |
| 2 | P'-7 | 77.2452774 | 77.2452774 |  |
| 2 | P'-8 | 77.2452774 | 77.2452774 |  |
| 2 | P'-9 | 77.2452774 | 77.2452774 |  |
| 2 | P'-10 | 77.2452774 | 77.2452774 |  |
| 2 | P'-11 | 77.2452774 | 77.2452774 |  |
| 2 | P'-12 | 77.2452774 | 77.2452774 |  |
| 2 | OH'-1 | 77.2452774 | 77.2452774 |  |
| 2 | OH'-2 | 77.2452774 | 77.2452774 |  |
| 2 | OH'-3 | 77.2452774 | 77.2452774 |  |
| 2 | OH'-4 | 77.2452774 | 77.2452774 |  |
| 2 | OH'-5 | 77.2452774 | 77.2452774 |  |
| 2 | OH'-6 | 77.2452774 | 77.2452774 |  |
| 2 | OH'-7 | 77.2452774 | 77.2452774 |  |
| 2 | OH'-8 | 77.2452774 | 77.2452774 |  |
| 2 | OH'-9 | 77.2452774 | 77.2452774 |  |
| 2 | OH'-10 | 77.2452774 | 77.2452774 |  |
| 2 | OH'-11 | 77.2452774 | 77.2452774 |  |
| 2 | OH'-12 | 77.2452774 | 77.2452774 |  |
| 3 | OF-1 | 77.25179291 | 77.25179291 |  |
| 3 | OF-2 | 77.25179291 | 77.25179291 |  |
| 3 | OF-3 | 77.25179291 | 77.25179291 |  |
| 3 | OF-4 | 77.25179291 | 77.25179291 |  |
| 3 | OF-5 | 77.25179291 | 77.25179291 |  |
| 3 | OF-6 | 77.25179291 | 77.25179291 |  |
| 3 | OF-7 | 77.25179291 | 77.25179291 |  |
| 3 | OF-8 | 77.25179291 | 77.25179291 |  |
| 3 | OF-9 | 77.25179291 | 77.25179291 |  |
| 3 | OF-10 | 77.25179291 | 77.25179291 |  |
| 3 | OF-11 | 77.25179291 | 77.25179291 |  |
| 3 | OF-12 | 77.25179291 | 77.25179291 |  |
| 3 | OF-13 | 77.25179291 | 77.25179291 |  |
| 3 | OF-14 | 77.25179291 | 77.25179291 |  |
| 3 | OF-15 | 77.25179291 | 77.25179291 |  |
| 3 | OF-16 | 77.25179291 | 77.25179291 |  |
| 3 | A-1 | 77.25179291 | 77.25179291 |  |
| 3 | A-2 | 77.25179291 | 77.25179291 |  |
| 3 | A-3 | 77.25179291 | 77.25179291 |  |
| 3 | A-4 | 77.25179291 | 77.25179291 |  |
| 3 | A-5 | 77.25179291 | 77.25179291 |  |
| 3 | A-6 | 77.25179291 | 77.25179291 |  |
| 3 | A-7 | 77.25179291 | 77.25179291 |  |
| 3 | A-8 | 77.25179291 | 77.25179291 |  |
| 3 | A-9 | 77.25179291 | 77.25179291 |  |
| 3 | A-10 | 77.25179291 | 77.25179291 |  |
| 3 | A-11 | 77.25179291 | 77.25179291 |  |
| 3 | A-12 | 77.25179291 | 77.25179291 |  |
| 3 | B-1 | 77.24565125 | 77.24565125 |  |
| 3 | B-2 | 77.24565125 | 77.24565125 |  |
| 3 | B-3 | 77.24565125 | 77.24565125 |  |
| 3 | B-4 | 77.24565125 | 77.24565125 |  |
| 3 | B-5 | 77.24565125 | 77.24565125 |  |
| 3 | B-6 | 77.24565125 | 77.24565125 |  |
| 3 | B-7 | 77.24565125 | 77.24565125 |  |
| 3 | B-8 | 77.25179291 | 77.25179291 |  |
| 3 | TA-1 | 77.24565125 | 77.24565125 |  |
| 3 | TA-2 | 77.24565125 | 77.24565125 |  |
| 3 | TA-3 | 77.24565125 | 77.24565125 |  |
| 3 | TA-4 | 77.24565125 | 77.24565125 |  |
| 3 | TA-5 | 77.24565125 | 77.24565125 |  |
| 3 | TA-6 | 77.24565125 | 77.24565125 |  |
| 3 | TA-7 | 77.24565125 | 77.24565125 |  |
| 3 | TA-8 | 77.24565125 | 77.24565125 |  |
| 3 | TA-9 | 77.24565125 | 77.24565125 |  |
| 3 | TA-10 | 77.24565125 | 77.24565125 |  |
| 3 | TA-11 | 77.24565125 | 77.24565125 |  |
| 3 | TA-12 | 77.24565125 | 77.24565125 |  |
| 3 | TH-1 | 77.24565125 | 77.24565125 |  |
| 3 | TH-2 | 77.58390808 | 77.58390808 |  |
| 3 | TH-3 | 77.58390808 | 77.58390808 |  |
| 3 | TH-4 | 77.08573151 | 77.08573151 |  |
| 3 | TH-5 | 77.24565125 | 77.24565125 |  |
| 3 | TH-6 | 77.24565125 | 77.24565125 | ground |
| 3 | TH-7 | 77.24565125 | 77.24565125 | common |
| 3 | TH-8 | 77.24565125 | 77.24565125 |  |
| 3 | TH-9 | 77.24565125 | 77.24565125 |  |
| 3 | TH-10 | 77.24565125 | 77.24565125 |  |
| 3 | TH-11 | 77.24565125 | 77.24565125 |  |
| 3 | TH-12 | 77.24565125 | 77.24565125 |  |
| 3 | P-1 | 77.4119873 | 77.4119873 |  |
| 3 | P-2 | 77.58390808 | 77.58390808 |  |
| 3 | P-3 | 77.58390808 | 77.58390808 |  |
| 3 | P-4 | 77.58390808 | 77.58390808 |  |
| 3 | P-5 | 77.58390808 | 77.58390808 |  |
| 3 | P-6 | 77.58390808 | 77.58390808 |  |
| 3 | P-7 | 77.58390808 | 77.58390808 |  |
| 3 | P-8 | 77.4119873 | 77.4119873 |  |
| 3 | PO-1 | 77.24565125 | 77.24565125 |  |
| 3 | PO-2 | 77.24565125 | 77.24565125 |  |
| 3 | PO-3 | 77.24565125 | 77.24565125 |  |
| 3 | PO-4 | 77.24565125 | 77.24565125 |  |
| 3 | PO-5 | 77.24565125 | 77.24565125 |  |
| 3 | PO-6 | 77.24565125 | 77.24565125 |  |
| 3 | PO-7 | 77.24565125 | 77.24565125 |  |
| 3 | PO-8 | 77.24565125 | 77.24565125 |  |
| 4 | A-1 | 77.41283417 | 77.41283417 |  |
| 4 | A-2 | 77.41283417 | 77.41283417 |  |
| 4 | A-3 | 77.41283417 | 77.41283417 |  |
| 4 | A-4 | 77.41283417 | 77.41283417 |  |
| 4 | A-5 | 77.41283417 | 77.41283417 |  |
| 4 | A-6 | 77.41283417 | 77.41283417 |  |
| 4 | A-7 | 77.41283417 | 77.41283417 |  |
| 4 | A-8 | 77.41283417 | 77.41283417 |  |
| 4 | A-9 | 77.41283417 | 77.41283417 |  |
| 4 | A-10 | 77.41283417 | 77.41283417 |  |
| 4 | TH-1 | 77.41311646 | 77.41311646 |  |
| 4 | TH-2 | 77.41311646 | 77.41311646 |  |
| 4 | TH-3 | 77.41311646 | 77.41311646 |  |
| 4 | TH-4 | 77.41311646 | 77.41311646 |  |
| 4 | TH-5 | 77.41311646 | 77.41311646 |  |
| 4 | TH-6 | 77.41311646 | 77.41311646 |  |
| 4 | TH-7 | 77.41311646 | 77.41311646 |  |
| 4 | TH-8 | 77.41311646 | 77.41311646 |  |
| 4 | PO1-1 | 77.41311646 | 77.41311646 |  |
| 4 | PO1-2 | 77.41311646 | 77.41311646 |  |
| 4 | PO1-3 | 77.41311646 | 77.41311646 |  |
| 4 | PO1-4 | 77.41311646 | 77.41311646 |  |
| 4 | PO1-5 | 77.41311646 | 77.41311646 |  |
| 4 | PO1-6 | 77.41311646 | 77.41311646 |  |
| 4 | PO1-7 | 77.41311646 | 77.41311646 |  |
| 4 | PO1-8 | 77.41311646 | 77.41311646 |  |
| 4 | PO2-1 | 77.41311646 | 77.41311646 |  |
| 4 | PO2-2 | 77.41311646 | 77.41311646 |  |
| 4 | PO2-3 | 77.41311646 | 77.41311646 |  |
| 4 | PO2-4 | 77.41311646 | 77.41311646 |  |
| 4 | PO2-5 | 77.41311646 | 77.41311646 |  |
| 4 | PO2-6 | 77.41311646 | 77.41311646 |  |
| 4 | PO2-7 | 77.41311646 | 77.41311646 |  |
| 4 | PO2-8 | 77.41311646 | 77.41311646 |  |
| 4 | PO2-9 | 77.41311646 | 77.41311646 |  |
| 4 | PO2-10 | 77.41311646 | 77.41311646 |  |
| 4 | P-1 | 77.41311646 | 77.41311646 |  |
| 4 | P-2 | 77.41311646 | 77.41311646 |  |
| 4 | P-3 | 77.41311646 | 77.41311646 |  |
| 4 | P-4 | 77.41311646 | 77.41311646 |  |
| 4 | P-5 | 77.41311646 | 77.41311646 |  |
| 4 | P-6 | 77.41311646 | 77.41311646 |  |
| 4 | P-7 | 77.41311646 | 77.41311646 |  |
| 4 | P-8 | 77.41311646 | 77.41311646 |  |
| 4 | TH'-1 | 77.41311646 | 77.41311646 |  |
| 4 | TH'-2 | 77.41311646 | 77.41311646 |  |
| 4 | TH'-3 | 77.41311646 | 77.41311646 |  |
| 4 | TH'-4 | 77.41311646 | 77.41311646 | broken |
| 4 | TH'-5 | 77.41311646 | 77.41311646 |  |
| 4 | TH'-6 | 77.41311646 | 77.41311646 |  |
| 4 | TH'-7 | 77.41311646 | 77.41311646 |  |
| 4 | TH'-8 | 77.41311646 | 77.41311646 |  |
| 4 | PO1'-1 | 77.41311646 | 77.41311646 |  |
| 4 | PO1'-2 | 77.41311646 | 77.41311646 |  |
| 4 | PO1'-3 | 77.41311646 | 77.41311646 |  |
| 4 | PO1'-4 | 77.41311646 | 77.41311646 |  |
| 4 | PO1'-5 | 77.41311646 | 77.41311646 |  |
| 4 | PO1'-6 | 77.41311646 | 77.41311646 |  |
| 4 | PO1'-7 | 77.41311646 | 77.41311646 |  |
| 4 | PO1'-8 | 77.41311646 | 77.41311646 |  |
| 4 | PO2'-1 | 77.41311646 | 77.41311646 |  |
| 4 | PO2'-2 | 77.41311646 | 77.41311646 |  |
| 4 | PO2'-3 | 77.41311646 | 77.41311646 |  |
| 4 | PO2'-4 | 77.41311646 | 77.41311646 |  |
| 4 | PO2'-5 | 77.41311646 | 77.41311646 |  |
| 4 | PO2'-6 | 77.41311646 | 77.41311646 |  |
| 4 | PO2'-7 | 77.41311646 | 77.41311646 |  |
| 4 | PO2'-8 | 77.41311646 | 77.41311646 |  |
| 4 | P1'-1 | 77.41311646 | 77.41311646 |  |
| 4 | P1'-2 | 77.41311646 | 77.41311646 |  |
| 4 | P1'-3 | 77.41311646 | 77.41311646 |  |
| 4 | P1'-4 | 77.41311646 | 77.41311646 |  |
| 4 | P1'-5 | 77.41311646 | 77.41311646 |  |
| 4 | P1'-6 | 77.41311646 | 77.41311646 |  |
| 4 | P1'-7 | 77.41311646 | 77.41311646 |  |
| 4 | P1'-8 | 77.41311646 | 77.41311646 |  |
| 4 | P2'-1 | 77.41311646 | 77.41311646 |  |
| 4 | P2'-2 | 77.41311646 | 77.41311646 |  |
| 4 | P2'-3 | 77.41311646 | 77.41311646 |  |
| 4 | P2'-4 | 77.41311646 | 77.41311646 |  |
| 4 | P2'-5 | 77.41311646 | 77.41311646 |  |
| 4 | P2'-6 | 77.41311646 | 77.41311646 |  |
| 4 | P2'-7 | 77.41311646 | 77.41311646 |  |
| 4 | P2'-8 | 77.41311646 | 77.41311646 |  |
| 5 | A-1 | 77.41388702 | 77.41388702 |  |
| 5 | A-2 | 77.41388702 | 77.41388702 |  |
| 5 | A-3 | 77.41388702 | 77.41388702 |  |
| 5 | A-4 | 77.41388702 | 77.41388702 |  |
| 5 | A-5 | 77.41388702 | 77.41388702 |  |
| 5 | A-6 | 77.41388702 | 77.41388702 |  |
| 5 | A-7 | 77.41388702 | 77.41388702 |  |
| 5 | A-8 | 77.41388702 | 77.41388702 |  |
| 5 | A-9 | 77.41388702 | 77.41388702 |  |
| 5 | A-10 | 77.41388702 | 77.41388702 |  |
| 5 | A-11 | 77.41388702 | 77.41388702 |  |
| 5 | A-12 | 77.41388702 | 77.41388702 |  |
| 5 | A-13 | 77.41388702 | 77.41388702 |  |
| 5 | A-14 | 77.41388702 | 77.41388702 |  |
| 5 | A-15 | 77.41388702 | 77.41388702 |  |
| 5 | B-1 | 77.41388702 | 77.41388702 |  |
| 5 | B-2 | 77.41388702 | 77.41388702 |  |
| 5 | B-3 | 77.41388702 | 77.41388702 |  |
| 5 | B-4 | 77.41388702 | 77.41388702 |  |
| 5 | B-5 | 77.41388702 | 77.41388702 |  |
| 5 | B-6 | 77.41388702 | 77.41388702 |  |
| 5 | B-7 | 77.41388702 | 77.41388702 |  |
| 5 | B-8 | 77.41388702 | 77.41388702 |  |
| 5 | B-9 | 77.41388702 | 77.41388702 |  |
| 5 | B-10 | 77.41388702 | 77.41388702 |  |
| 5 | TA-1 | 30.3330555 | 77.41388702 |  |
| 5 | TA-2 | 30.3330555 | 77.41388702 |  |
| 5 | TA-3 | 30.3330555 | 77.41388702 |  |
| 5 | TA-4 | 30.3330555 | 77.41416931 |  |
| 5 | TA-5 | 30.3330555 | 77.41408408 |  |
| 5 | TA-6 | 30.3330555 | 77.41408408 |  |
| 5 | TA-7 | 29.99972153 | 66.00888824 | ground |
| 5 | TA-8 | 29.99972153 | 66.00888824 | common |
| 5 | TA-9 | 30.3330555 | 77.41388702 |  |
| 5 | TA-10 | 30.3330555 | 77.41388702 |  |
| 5 | TA-11 | 30.3330555 | 77.24666595 |  |
| 5 | TA-12 | 77.24666595 | 77.24666595 |  |
| 5 | TA-13 | 77.24666595 | 77.24666595 |  |
| 5 | TA-14 | 77.24666595 | 77.24666595 |  |
| 5 | TA-15 | 77.24666595 | 77.24666595 |  |
| 5 | C-1 | 77.24666595 | 77.24666595 |  |
| 5 | C-2 | 77.7472229 | 77.24666595 |  |
| 5 | C-3 | 77.7472229 | 77.24666595 |  |
| 5 | C-4 | 77.24666595 | 77.24666595 |  |
| 5 | C-5 | 77.24666595 | 77.24666595 |  |
| 5 | C-6 | 77.24666595 | 77.24666595 |  |
| 5 | C-7 | 77.41388702 | 77.41388702 |  |
| 5 | C-8 | 77.41388702 | 77.41388702 |  |
| 5 | C-9 | 77.41388702 | 77.41388702 |  |
| 5 | C-10 | 77.41388702 | 77.41388702 |  |
| 5 | P1-1 | 77.24666595 | 77.24666595 |  |
| 5 | P1-2 | 77.24666595 | 77.24666595 |  |
| 5 | P1-3 | 77.24666595 | 77.24666595 |  |
| 5 | P1-4 | 77.24666595 | 77.24666595 |  |
| 5 | P1-5 | 77.24666595 | 77.24666595 |  |
| 5 | P1-6 | 77.24666595 | 77.24666595 |  |
| 5 | P1-7 | 77.24666595 | 77.24666595 |  |
| 5 | P1-8 | 77.24666595 | 77.24666595 |  |
| 5 | P2-1 | 77.24666595 | 77.24666595 |  |
| 5 | P2-2 | 77.24666595 | 77.24666595 |  |
| 5 | P2-3 | 77.24666595 | 77.24666595 |  |
| 5 | P2-4 | 77.24666595 | 77.24666595 |  |
| 5 | P2-5 | 77.24666595 | 77.24666595 |  |
| 5 | FF-1 | 77.24666595 | 77.24666595 |  |
| 5 | FF-2 | 77.41388702 | 77.41388702 |  |
| 5 | FF-3 | 77.41388702 | 77.41388702 |  |
| 5 | FF-4 | 77.41388702 | 77.41388702 |  |
| 5 | FF-5 | 77.41388702 | 77.41388702 |  |
| 6 | OF-1 | 77.58055878 | 77.58055878 |  |
| 6 | OF-2 | 77.58055878 | 77.58055878 |  |
| 6 | OF-3 | 77.58055878 | 77.58055878 |  |
| 6 | OF-4 | 77.58055878 | 77.58055878 |  |
| 6 | OF-5 | 77.58055878 | 77.58055878 |  |
| 6 | OF-6 | 77.58055878 | 77.58055878 |  |
| 6 | OF-7 | 77.58055878 | 77.58055878 |  |
| 6 | OF-8 | 77.58055878 | 77.58055878 |  |
| 6 | OF-9 | 77.58055878 | 77.58055878 |  |
| 6 | OF-10 | 77.58055878 | 77.58055878 |  |
| 6 | OF-11 | 77.58055878 | 77.58055878 |  |
| 6 | OF-12 | 77.58055878 | 77.58055878 |  |
| 6 | B-1 | 77.58055878 | 77.58055878 |  |
| 6 | B-2 | 77.58055878 | 77.58055878 |  |
| 6 | B-3 | 77.58055878 | 77.58055878 |  |
| 6 | B-4 | 77.58055878 | 77.58055878 |  |
| 6 | B-5 | 77.58055878 | 77.58055878 |  |
| 6 | B-6 | 77.58055878 | 77.58055878 |  |
| 6 | B-7 | 77.58055878 | 77.58055878 |  |
| 6 | B-8 | 77.58055878 | 77.58055878 |  |
| 6 | B-9 | 77.58055878 | 77.58055878 |  |
| 6 | B-10 | 77.58055878 | 77.58055878 |  |
| 6 | TA-1 | 77.58055878 | 77.58055878 |  |
| 6 | TA-2 | 77.58055878 | 77.58055878 |  |
| 6 | TA-3 | 77.58055878 | 77.58055878 |  |
| 6 | TA-4 | 77.58055878 | 77.58055878 |  |
| 6 | TA-5 | 77.58055878 | 77.58055878 | ground |
| 6 | TA-6 | 77.58055878 | 77.58055878 | common |
| 6 | TA-7 | 77.58055878 | 77.58055878 |  |
| 6 | TA-8 | 77.58055878 | 77.58055878 |  |
| 6 | TA-9 | 77.58055878 | 77.58055878 |  |
| 6 | TA-10 | 77.58055878 | 77.58055878 |  |
| 6 | TA-11 | 77.58055878 | 77.58055878 |  |
| 6 | TA-12 | 77.58055878 | 77.58055878 |  |
| 6 | TH-1 | 77.58055878 | 77.58055878 |  |
| 6 | TH-2 | 77.58055878 | 77.58055878 |  |
| 6 | TH-3 | 77.58055878 | 77.58055878 |  |
| 6 | TH-4 | 77.58055878 | 77.58055878 |  |
| 6 | TH-5 | 77.58055878 | 77.58055878 |  |
| 6 | TH-6 | 77.58055878 | 77.58055878 |  |
| 6 | TH-7 | 77.58055878 | 77.58055878 |  |
| 6 | TH-8 | 77.58055878 | 77.58055878 |  |
| 6 | TH-9 | 77.58055878 | 77.58055878 |  |
| 6 | TH-10 | 77.58055878 | 77.58055878 |  |
| 6 | TH-11 | 77.58055878 | 77.58055878 |  |
| 6 | TH-12 | 77.58055878 | 77.58055878 |  |
| 6 | PO-1 | 77.08055878 | 77.08055878 |  |
| 6 | PO-2 | 77.08055878 | 77.08055878 |  |
| 6 | PO-3 | 77.58083344 | 77.58083344 |  |
| 6 | PO-4 | 77.58083344 | 77.58083344 |  |
| 6 | PO-5 | 77.58083344 | 77.58083344 |  |
| 6 | PO-6 | 29.99972153 | 66.00888824 | broken |
| 6 | PO-7 | 77.58055878 | 77.58055878 |  |
| 6 | PO-8 | 77.58055878 | 77.58055878 |  |
| 6 | PO-9 | 77.58055878 | 77.58055878 |  |
| 6 | PO-10 | 77.58055878 | 77.58055878 |  |
| 6 | W-1 | 77.58055878 | 77.58055878 |  |
| 6 | W-2 | 77.58055878 | 77.58055878 |  |
| 6 | W-3 | 77.58055878 | 77.58055878 |  |
| 6 | W-4 | 77.58055878 | 77.58055878 |  |
| 6 | W-5 | 77.58055878 | 77.58055878 |  |
| 6 | W-6 | 77.41361237 | 77.41361237 |  |
| 6 | W-7 | 77.58055878 | 77.58055878 |  |
| 6 | W-8 | 77.41361237 | 77.41361237 |  |
| 6 | W-9 | 77.41361237 | 77.41361237 |  |
| 6 | W-10 | 77.58055878 | 77.58055878 |  |
| 6 | W-11 | 77.58055878 | 77.58055878 |  |
| 6 | W-12 | 77.58055878 | 77.58055878 |  |
| 6 | FF-1 | 77.58055878 | 77.58055878 |  |
| 6 | FF-2 | 77.58055878 | 77.58055878 |  |
| 6 | FF-3 | 77.58055878 | 77.58055878 |  |
| 6 | FF-4 | 77.58055878 | 77.58055878 |  |
| 6 | FF-5 | 77.58055878 | 77.58055878 |  |
| 6 | FF-6 | 77.58055878 | 77.58055878 |  |
| 6 | FF-7 | 77.58055878 | 77.58055878 |  |
| 6 | FF-8 | 77.58055878 | 77.58055878 |  |
| 6 | FF-9 | 77.58055878 | 77.58055878 |  |
| 6 | FF-10 | 77.58055878 | 77.58055878 |  |
| 6 | P-1 | 77.58055878 | 77.58055878 |  |
| 6 | P-2 | 77.58055878 | 77.58055878 |  |
| 6 | P-3 | 77.58055878 | 77.58055878 |  |
| 6 | P-4 | 77.58055878 | 77.58055878 |  |
| 6 | P-5 | 77.58055878 | 77.58055878 |  |
| 6 | P-6 | 77.58055878 | 77.58055878 |  |
| 6 | P-7 | 77.58055878 | 77.58055878 |  |
| 6 | P-8 | 77.58055878 | 77.58055878 |  |
| 6 | P-9 | 77.58055878 | 77.58055878 |  |
| 6 | P-10 | 77.58055878 | 77.58055878 |  |
| 6 | P-11 | 77.58055878 | 77.58055878 |  |
| 6 | P-12 | 77.58055878 | 77.58055878 |  |
| 6 | O-1 | 77.58055878 | 77.58055878 |  |
| 6 | O-2 | 77.58055878 | 77.58055878 |  |
| 6 | O-3 | 77.58055878 | 77.58055878 |  |
| 6 | O-4 | 77.58055878 | 77.58055878 |  |
| 6 | O-5 | 77.58055878 | 77.58055878 |  |
| 6 | O-6 | 77.58055878 | 77.58055878 |  |
| 6 | O-7 | 77.58055878 | 77.58055878 |  |
| 6 | O-8 | 77.58055878 | 77.58055878 |  |
| 6 | O-9 | 77.58055878 | 77.58055878 |  |
| 6 | O-10 | 77.58055878 | 77.58055878 |  |
| 7 | OF-1 | 77.58361053 | 77.58361053 |  |
| 7 | OF-2 | 77.58361053 | 77.41666412 |  |
| 7 | OF-3 | 77.41666412 | 77.41666412 |  |
| 7 | OF-4 | 77.41666412 | 77.41666412 |  |
| 7 | OF-5 | 77.41638947 | 77.58361053 |  |
| 7 | OF-6 | 30.99972153 | 70.16805267 | ground |
| 7 | OF-7 | 30.99972153 | 74.30305481 | common |
| 7 | OF-8 | 31.50027847 | 70.49861145 | unreliable |
| 7 | OF-9 | 31.50027847 | 70.49861145 | unreliable |
| 7 | OF-10 | 29.99972153 | 69.9991684 | unreliable |
| 7 | A-1 | 77.58361053 | 77.58361053 |  |
| 7 | A-2 | 77.58361053 | 77.58361053 |  |
| 7 | A-3 | 77.58361053 | 77.41666412 |  |
| 7 | A-4 | 77.41666412 | 77.41666412 |  |
| 7 | A-5 | 77.41666412 | 77.41666412 |  |
| 7 | A-6 | 77.58361053 | 77.58361053 |  |
| 7 | A-7 | 77.41666412 | 77.41666412 |  |
| 7 | A-8 | 77.58361053 | 77.58361053 |  |
| 7 | A-9 | 77.24861145 | 77.24861145 |  |
| 7 | A-10 | 77.58361053 | 77.58361053 |  |
| 7 | B-1 | 77.58361053 | 77.58361053 |  |
| 7 | B-2 | 77.58361053 | 77.58361053 |  |
| 7 | B-3 | 77.58361053 | 77.58361053 |  |
| 7 | B-4 | 77.58361053 | 77.58361053 |  |
| 7 | B-5 | 77.58361053 | 77.58361053 |  |
| 7 | B-6 | 77.58361053 | 77.58361053 |  |
| 7 | B-7 | 77.58361053 | 77.58361053 |  |
| 7 | B-8 | 77.58361053 | 77.58361053 |  |
| 7 | TA-1 | 77.41666412 | 77.41666412 |  |
| 7 | TA-2 | 77.58361053 | 77.58361053 |  |
| 7 | TA-3 | 77.41666412 | 77.41666412 |  |
| 7 | TA-4 | 77.58361053 | 77.58361053 |  |
| 7 | TA-5 | 77.41666412 | 77.58361053 |  |
| 7 | TA-6 | 77.41666412 | 77.41666412 |  |
| 7 | TA-7 | 77.41666412 | 77.41666412 |  |
| 7 | TA-8 | 77.41666412 | 77.41666412 |  |
| 7 | TH-1 | 77.58361053 | 77.58361053 |  |
| 7 | TH-2 | 77.58361053 | 77.58361053 |  |
| 7 | TH-3 | 77.58361053 | 77.58361053 |  |
| 7 | TH-4 | 77.58361053 | 77.58361053 |  |
| 7 | TH-5 | 77.58361053 | 77.58361053 |  |
| 7 | TH-6 | 77.58361053 | 77.58361053 |  |
| 7 | TH-7 | 77.58361053 | 77.58361053 |  |
| 7 | TH-8 | 77.41666412 | 77.41666412 |  |
| 7 | TH-9 | 77.58361053 | 77.58361053 |  |
| 7 | TH-10 | 77.58361053 | 77.58361053 |  |
| 7 | IP-1 | 77.41666412 | 77.41666412 |  |
| 7 | IP-2 | 77.41666412 | 77.41666412 |  |
| 7 | IP-3 | 77.41666412 | 77.41666412 |  |
| 7 | IP-4 | 77.41666412 | 77.41666412 |  |
| 7 | IP-5 | 77.41666412 | 77.41666412 |  |
| 7 | IP-6 | 77.41666412 | 77.41666412 |  |
| 7 | IP-7 | 77.41666412 | 77.41666412 |  |
| 7 | IP-8 | 77.41666412 | 77.41666412 |  |
| 7 | P-1 | 77.58361053 | 77.58361053 |  |
| 7 | P-2 | 77.58361053 | 77.58361053 |  |
| 7 | P-3 | 77.58361053 | 77.58361053 |  |
| 7 | P-4 | 77.58361053 | 77.58361053 |  |
| 7 | P-5 | 77.58361053 | 77.58361053 |  |
| 7 | TA'-1 | 77.41666412 | 77.41666412 |  |
| 7 | TA'-2 | 77.41666412 | 77.41666412 |  |
| 7 | TA'-3 | 77.58361053 | 77.58361053 |  |
| 7 | TA'-4 | 77.41666412 | 77.41666412 |  |
| 7 | TA'-5 | 77.41666412 | 77.41666412 |  |
| 7 | TA'-6 | 77.41666412 | 77.41666412 |  |
| 7 | TA'-7 | 77.41666412 | 77.41666412 |  |
| 7 | TA'-8 | 77.41666412 | 77.41666412 |  |
| 7 | TH'-1 | 77.41666412 | 77.41666412 |  |
| 7 | TH'-2 | 77.41666412 | 77.41666412 |  |
| 7 | TH'-3 | 77.58361053 | 77.58361053 |  |
| 7 | TH'-4 | 77.41666412 | 77.41666412 |  |
| 7 | TH'-5 | 77.58361053 | 77.58361053 |  |
| 7 | TH'-6 | 77.58361053 | 77.58361053 |  |
| 7 | TH'-7 | 77.58361053 | 77.58361053 |  |
| 7 | TH'-8 | 77.58361053 | 77.58361053 |  |
| 7 | TH'-9 | 77.58361053 | 77.58361053 |  |
| 7 | TH'-10 | 77.41666412 | 77.41666412 |  |
| 8 | OF-1 | 77.41055298 | 77.41055298 |  |
| 8 | OF-2 | 77.41055298 | 77.41055298 |  |
| 8 | OF-3 | 77.41055298 | 77.41055298 |  |
| 8 | OF-4 | 77.41055298 | 77.41055298 |  |
| 8 | OF-5 | 77.41055298 | 77.41055298 |  |
| 8 | OF-6 | 77.41055298 | 77.41055298 |  |
| 8 | OF-7 | 77.41055298 | 77.41055298 |  |
| 8 | OF-8 | 77.41055298 | 77.41055298 |  |
| 8 | OF-9 | 77.41055298 | 77.41055298 |  |
| 8 | OF-10 | 77.41055298 | 77.41055298 |  |
| 8 | TA1-1 | 77.57749939 | 77.57749939 |  |
| 8 | TA1-2 | 77.57749939 | 77.57749939 |  |
| 8 | TA1-3 | 77.2436142 | 77.41055298 |  |
| 8 | TA1-4 | 77.2436142 | 77.2436142 |  |
| 8 | TA1-5 | 77.2436142 | 77.41055298 |  |
| 8 | TA1-6 | 77.41055298 | 77.41055298 |  |
| 8 | TA1-7 | 77.41027832 | 77.41027832 | ground |
| 8 | TA1-8 | 77.41027832 | 77.41027832 | common |
| 8 | TA1-9 | 77.41027832 | 77.41027832 |  |
| 8 | TA1-10 | 77.41027832 | 77.41027832 |  |
| 8 | TA1-11 | 77.41027832 | 77.41027832 |  |
| 8 | TA1-12 | 77.41027832 | 77.41027832 |  |
| 8 | TA2-1 | 77.41027832 | 77.41027832 |  |
| 8 | TA2-2 | 77.41027832 | 77.41027832 |  |
| 8 | TA2-3 | 77.41027832 | 77.41027832 |  |
| 8 | TA2-4 | 77.41027832 | 77.41027832 |  |
| 8 | TA2-5 | 77.41027832 | 77.41027832 |  |
| 8 | TA2-6 | 77.41027832 | 77.41027832 |  |
| 8 | TA2-7 | 77.41027832 | 77.41027832 |  |
| 8 | TA2-8 | 77.41027832 | 77.41027832 |  |
| 8 | TA2-9 | 77.41027832 | 77.41027832 |  |
| 8 | TA2-10 | 77.41027832 | 77.41027832 |  |
| 8 | TA2-11 | 77.41027832 | 77.41027832 |  |
| 8 | TA2-12 | 77.41027832 | 77.41027832 |  |
| 8 | TH1-1 | 77.41027832 | 77.41027832 |  |
| 8 | TH1-2 | 77.41027832 | 77.41027832 |  |
| 8 | TH1-3 | 77.41027832 | 77.41027832 |  |
| 8 | TH1-4 | 77.41027832 | 77.41027832 |  |
| 8 | TH1-5 | 77.41027832 | 77.41027832 |  |
| 8 | TH1-6 | 77.41027832 | 77.41027832 |  |
| 8 | TH1-7 | 77.41027832 | 77.41027832 |  |
| 8 | TH1-8 | 77.41027832 | 77.41027832 |  |
| 8 | TH1-9 | 77.41027832 | 77.41027832 |  |
| 8 | TH1-10 | 77.41027832 | 77.41027832 |  |
| 8 | TH1-11 | 77.41027832 | 77.41027832 |  |
| 8 | TH1-12 | 77.41027832 | 77.41027832 |  |
| 8 | TH-1 | 77.41027832 | 77.41027832 |  |
| 8 | TH-2 | 77.41027832 | 77.41027832 |  |
| 8 | TH-3 | 77.41027832 | 77.41027832 |  |
| 8 | TH-4 | 77.41027832 | 77.41027832 |  |
| 8 | TH-5 | 77.41027832 | 77.41027832 |  |
| 8 | TH-6 | 77.41027832 | 77.41027832 |  |
| 8 | TH-7 | 77.41027832 | 77.41027832 |  |
| 8 | TH-8 | 77.41027832 | 77.41027832 |  |
| 8 | TH-9 | 77.41027832 | 77.41027832 |  |
| 8 | TH-10 | 77.41027832 | 77.41027832 |  |
| 8 | TH-11 | 77.41027832 | 77.41027832 |  |
| 8 | TH-12 | 77.41027832 | 77.41027832 |  |
| 8 | OH-1 | 77.41027832 | 77.41027832 |  |
| 8 | OH-2 | 77.41027832 | 77.41027832 |  |
| 8 | OH-3 | 77.41027832 | 77.41027832 |  |
| 8 | OH-4 | 77.41027832 | 77.41027832 |  |
| 8 | OH-5 | 77.41027832 | 77.41027832 |  |
| 8 | OH-6 | 77.41027832 | 77.41027832 |  |
| 8 | OH-7 | 77.41027832 | 77.41027832 |  |
| 8 | OH-8 | 77.41027832 | 77.41027832 |  |
| 8 | OH-9 | 77.41027832 | 77.41027832 |  |
| 8 | OH-10 | 77.41027832 | 77.41027832 |  |
| 8 | OH-11 | 77.24610901 | 77.24610901 |  |
| 8 | OH-12 | 77.41027832 | 77.41027832 |  |
| 8 | OH-13 | 77.24610901 | 77.24610901 |  |
| 8 | OH-14 | 77.24583435 | 77.24583435 |  |
| 8 | OH-15 | 77.24583435 | 77.24583435 |  |
| 9 | OF'-1 | 77.41338348 | 77.41338348 |  |
| 9 | OF'-2 | 77.41338348 | 77.41338348 |  |
| 9 | OF'-3 | 77.41338348 | 77.41338348 |  |
| 9 | OF'-4 | 547.0178833 | 79.66297913 | broken |
| 9 | OF'-5 | 77.41338348 | 77.41338348 |  |
| 9 | OF'-6 | 77.58008575 | 77.58008575 |  |
| 9 | OF'-7 | 77.58008575 | 77.58008575 |  |
| 9 | OF'-8 | 77.58008575 | 77.58008575 |  |
| 9 | OF'-9 | 77.58008575 | 77.58008575 |  |
| 9 | OF'-10 | 77.58008575 | 77.58008575 |  |
| 9 | TA1'-1 | 77.24278259 | 77.24278259 |  |
| 9 | TA1'-2 | 77.24278259 | 77.24278259 |  |
| 9 | TA1'-3 | 77.24278259 | 77.24278259 |  |
| 9 | TA1'-4 | 77.24278259 | 77.24278259 |  |
| 9 | TA1'-5 | 77.24278259 | 77.24278259 |  |
| 9 | TA1'-6 | 77.24278259 | 77.24278259 |  |
| 9 | TA1'-7 | 77.24278259 | 77.24278259 |  |
| 9 | TA1'-8 | 77.24278259 | 77.24278259 |  |
| 9 | TA1'-9 | 77.24278259 | 77.24278259 |  |
| 9 | TA1'-10 | 77.24278259 | 77.24278259 |  |
| 9 | TA1'-11 | 77.24278259 | 77.24278259 |  |
| 9 | TA1'-12 | 77.24278259 | 77.24278259 |  |
| 9 | TA2'-1 | 77.24278259 | 77.24278259 |  |
| 9 | TA2'-2 | 77.24278259 | 77.24278259 |  |
| 9 | TA2'-3 | 77.24278259 | 77.24278259 |  |
| 9 | TA2'-4 | 77.24278259 | 77.24278259 |  |
| 9 | TA2'-5 | 77.24278259 | 77.24278259 |  |
| 9 | TA2'-6 | 77.24278259 | 77.24278259 |  |
| 9 | TA2'-7 | 77.24278259 | 77.24278259 |  |
| 9 | TA2'-8 | 77.24278259 | 77.24278259 |  |
| 9 | TA2'-9 | 77.24278259 | 77.24278259 |  |
| 9 | TA2'-10 | 77.24278259 | 77.24278259 |  |
| 9 | TA2'-11 | 77.24278259 | 77.24278259 |  |
| 9 | TA2'-12 | 77.24278259 | 77.24278259 |  |
| 9 | TH1'-1 | 77.24278259 | 77.24278259 |  |
| 9 | TH1'-2 | 77.24278259 | 77.24278259 |  |
| 9 | TH1'-3 | 77.24278259 | 77.24278259 |  |
| 9 | TH1'-4 | 77.24278259 | 77.24278259 |  |
| 9 | TH1'-5 | 77.24278259 | 77.24278259 |  |
| 9 | TH1'-6 | 77.24278259 | 77.24278259 |  |
| 9 | TH1'-7 | 77.24278259 | 77.24278259 |  |
| 9 | TH1'-8 | 77.24278259 | 77.24278259 |  |
| 9 | TH1'-9 | 77.24278259 | 77.24278259 |  |
| 9 | TH1'-10 | 77.24278259 | 77.24278259 |  |
| 9 | TH1'-11 | 77.24278259 | 77.24278259 |  |
| 9 | TH1'-12 | 77.24278259 | 77.24278259 |  |
| 9 | TH'-1 | 77.24278259 | 77.24278259 |  |
| 9 | TH'-2 | 77.24278259 | 77.24278259 |  |
| 9 | TH'-3 | 77.24278259 | 77.24278259 |  |
| 9 | TH'-4 | 77.24278259 | 77.24278259 |  |
| 9 | TH'-5 | 77.24278259 | 77.24278259 |  |
| 9 | TH'-6 | 77.24278259 | 77.24278259 |  |
| 9 | TH'-7 | 77.24278259 | 77.24278259 |  |
| 9 | TH'-8 | 77.24278259 | 77.24278259 |  |
| 9 | TH'-9 | 77.24278259 | 77.24278259 |  |
| 9 | TH'-10 | 77.24278259 | 77.24278259 |  |
| 9 | TH'-11 | 77.24278259 | 77.24278259 |  |
| 9 | TH'-12 | 77.24278259 | 77.24278259 |  |
| 9 | OH'-1 | 77.24278259 | 77.24278259 |  |
| 9 | OH'-2 | 77.24278259 | 77.24278259 |  |
| 9 | OH'-3 | 77.24278259 | 77.24278259 |  |
| 9 | OH'-4 | 77.24278259 | 77.24278259 |  |
| 9 | OH'-5 | 77.24278259 | 77.24278259 |  |
| 9 | OH'-6 | 77.24278259 | 77.24278259 |  |
| 9 | OH'-7 | 77.24278259 | 77.24278259 |  |
| 9 | OH'-8 | 77.24278259 | 77.24278259 |  |
| 9 | OH'-9 | 77.24278259 | 77.24278259 |  |
| 9 | OH'-10 | 77.24278259 | 77.24278259 |  |
| 9 | OH'-11 | 77.24278259 | 77.24278259 |  |
| 9 | OH'-12 | 77.24278259 | 77.24278259 |  |
| 9 | OH'-13 | 77.24278259 | 77.24278259 |  |
| 9 | OH'-14 | 797.1834106 | 77.24278259 | ground |
| 9 | OH'-15 | 999.3712769 | 69.56349945 | common |
| 10 | B-1 | 77.24416351 | 77.24416351 |  |
| 10 | B-2 | 77.24416351 | 77.24416351 |  |
| 10 | B-3 | 77.24416351 | 77.24416351 |  |
| 10 | B-4 | 77.41222382 | 77.41222382 |  |
| 10 | B-5 | 77.41194153 | 77.41194153 |  |
| 10 | B-6 | 77.41194153 | 77.41194153 |  |
| 10 | B-7 | 77.41194153 | 77.41194153 |  |
| 10 | B-8 | 77.41194153 | 77.41194153 |  |
| 10 | TH-1 | 77.41194153 | 77.41194153 |  |
| 10 | TH-2 | 77.41194153 | 77.41194153 |  |
| 10 | TH-3 | 77.41194153 | 77.41194153 |  |
| 10 | TH-4 | 77.41194153 | 77.41194153 |  |
| 10 | TH-5 | 77.41194153 | 77.41194153 |  |
| 10 | TH-6 | 77.41194153 | 77.41194153 |  |
| 10 | TH-7 | 77.41194153 | 77.41194153 | ground |
| 10 | TH-8 | 77.41194153 | 77.41194153 | common |
| 10 | TH-9 | 77.41194153 | 77.41194153 |  |
| 10 | TH-10 | 77.41194153 | 77.41194153 |  |
| 10 | TH-11 | 77.41194153 | 77.41194153 |  |
| 10 | TH-12 | 77.41194153 | 77.41194153 |  |
| 10 | P-1 | 77.41194153 | 77.41194153 |  |
| 10 | P-2 | 77.41194153 | 77.41194153 |  |
| 10 | P-3 | 77.41194153 | 77.41194153 |  |
| 10 | P-4 | 77.41194153 | 77.41194153 |  |
| 10 | P-5 | 77.24388885 | 77.24388885 |  |
| 10 | P-6 | 77.24388885 | 77.24388885 |  |
| 10 | P-7 | 77.24388885 | 77.24388885 |  |
| 10 | P-8 | 77.24388885 | 77.24388885 |  |
| 10 | PO-1 | 77.41194153 | 77.41194153 |  |
| 10 | PO-2 | 77.41194153 | 77.41194153 |  |
| 10 | PO-3 | 77.41194153 | 77.41194153 |  |
| 10 | PO-4 | 77.41194153 | 77.41194153 |  |
| 10 | PO-5 | 77.41194153 | 77.41194153 |  |
| 10 | PO-6 | 77.41194153 | 77.41194153 |  |
| 10 | PO-7 | 77.41194153 | 77.41194153 |  |
| 10 | PO-8 | 77.24388885 | 77.24388885 |  |
| 10 | TO-1 | 77.41222382 | 77.41222382 |  |
| 10 | TO-2 | 77.24388885 | 77.24388885 |  |
| 10 | TO-3 | 77.41222382 | 77.41222382 |  |
| 10 | TO-4 | 77.24388885 | 77.24388885 |  |
| 10 | TO-5 | 77.24388885 | 77.24388885 |  |
| 10 | TO-6 | 77.41222382 | 77.41222382 |  |
| 10 | TO-7 | 77.41222382 | 77.41222382 |  |
| 10 | TO-8 | 77.24388885 | 77.24388885 |  |
| 10 | TO-9 | 77.24388885 | 77.24388885 |  |
| 10 | TO-10 | 77.24388885 | 77.24388885 |  |
| 10 | V-1 | 77.41194153 | 77.41194153 |  |
| 10 | V-2 | 77.41194153 | 77.41194153 |  |
| 10 | V-3 | 77.41194153 | 77.41194153 |  |
| 10 | V-4 | 77.41194153 | 77.41194153 |  |
| 10 | V-5 | 77.41194153 | 77.41194153 |  |
| 10 | V-6 | 77.41194153 | 77.41194153 |  |
| 10 | V-7 | 77.41194153 | 77.41194153 |  |
| 10 | V-8 | 77.41194153 | 77.41194153 |  |
| 10 | V-9 | 77.41194153 | 77.41194153 |  |
| 10 | V-10 | 77.41194153 | 77.41194153 |  |
| 10 | B'-1 | 77.41194153 | 77.41194153 |  |
| 10 | B'-2 | 77.41194153 | 77.41194153 |  |
| 10 | B'-3 | 77.41194153 | 77.41194153 |  |
| 10 | B'-4 | 77.41194153 | 77.41194153 |  |
| 10 | B'-5 | 77.41194153 | 77.41194153 |  |
| 10 | B'-6 | 77.41194153 | 77.41194153 |  |
| 10 | B'-7 | 77.41194153 | 77.41194153 |  |
| 10 | B'-8 | 77.24416351 | 77.24416351 |  |
| 10 | TH'-1 | 77.41194153 | 77.41194153 |  |
| 10 | TH'-2 | 77.41194153 | 77.41194153 |  |
| 10 | TH'-3 | 77.41194153 | 77.41194153 |  |
| 10 | TH'-4 | 77.41194153 | 77.41194153 |  |
| 10 | TH'-5 | 77.41194153 | 77.41194153 |  |
| 10 | TH'-6 | 77.41194153 | 77.41194153 |  |
| 10 | TH'-7 | 77.41194153 | 77.41194153 |  |
| 10 | TH'-8 | 77.41194153 | 77.41194153 |  |
| 10 | TH'-9 | 77.41194153 | 77.41194153 |  |
| 10 | TH'-10 | 77.41194153 | 77.41194153 |  |
| 10 | TH'-11 | 77.41194153 | 77.41194153 |  |
| 10 | TH'-12 | 77.41194153 | 77.41194153 |  |
| 10 | PO'-1 | 77.41194153 | 77.41194153 |  |
| 10 | PO'-2 | 77.41194153 | 77.41194153 |  |
| 10 | PO'-3 | 77.41194153 | 77.41194153 |  |
| 10 | PO'-4 | 77.41194153 | 77.41194153 |  |
| 10 | PO'-5 | 77.41194153 | 77.41194153 |  |
| 10 | PO'-6 | 77.41194153 | 77.41194153 |  |
| 10 | V'-1 | 77.24388885 | 77.24388885 |  |
| 10 | V'-2 | 77.24388885 | 77.24388885 |  |
| 10 | V'-3 | 77.24388885 | 77.24388885 |  |
| 10 | V'-4 | 77.24388885 | 77.24388885 |  |
| 10 | V'-5 | 77.41194153 | 77.41194153 |  |
| 10 | V'-6 | 77.41194153 | 77.41194153 |  |
| 10 | V'-7 | 77.41194153 | 77.41194153 |  |
| 10 | V'-8 | 77.41194153 | 77.41194153 |  |
| 10 | V'-9 | 77.41194153 | 77.41194153 |  |
| 10 | V'-10 | 77.41194153 | 77.41194153 |  |
| 10 | V'-11 | 77.41194153 | 77.41194153 |  |
| 10 | V'-12 | 77.41194153 | 77.41194153 |  |
| 11 | OF-1 | 77.57831573 | 77.57831573 |  |
| 11 | OF-2 | 77.57831573 | 77.57831573 |  |
| 11 | OF-3 | 77.57831573 | 77.57831573 |  |
| 11 | OF-4 | 77.57831573 | 77.57831573 |  |
| 11 | OF-5 | 77.57831573 | 77.57831573 |  |
| 11 | OF-6 | 77.57831573 | 77.57831573 |  |
| 11 | OF-7 | 77.57831573 | 77.57831573 |  |
| 11 | OF-8 | 77.57831573 | 77.57831573 |  |
| 11 | OF-9 | 77.57831573 | 77.57831573 |  |
| 11 | OF-10 | 77.57831573 | 77.57831573 |  |
| 11 | OF-11 | 77.57831573 | 77.57831573 |  |
| 11 | OF-12 | 77.57831573 | 77.57831573 |  |
| 11 | TA-1 | 45.45732117 | 77.24373627 |  |
| 11 | TA-2 | 77.41493225 | 77.41493225 |  |
| 11 | TA-3 | 77.41493225 | 77.41493225 |  |
| 11 | TA-4 | 77.41493225 | 77.41493225 |  |
| 11 | TA-5 | 45.21893311 | 77.41493225 |  |
| 11 | TA-6 | 45.23733521 | 77.41493225 |  |
| 11 | TA-7 | 999.3289185 | 70.05419159 | ground |
| 11 | TA-8 | 999.3534546 | 83.99219513 | common |
| 11 | TA-9 | 77.2454071 | 77.2454071 |  |
| 11 | TA-10 | 77.2454071 | 77.2454071 |  |
| 11 | TA-11 | 77.2454071 | 77.2454071 |  |
| 11 | TA-12 | 77.2454071 | 77.2454071 |  |
| 11 | TH-1 | 77.2454071 | 77.2454071 |  |
| 11 | TH-2 | 77.2454071 | 77.2454071 |  |
| 11 | TH-3 | 77.2454071 | 77.2454071 |  |
| 11 | TH-4 | 77.2454071 | 77.2454071 |  |
| 11 | TH-5 | 77.57831573 | 77.57831573 |  |
| 11 | TH-6 | 77.57831573 | 77.57831573 |  |
| 11 | TH-7 | 77.57831573 | 77.57831573 |  |
| 11 | TH-8 | 77.57831573 | 77.57831573 |  |
| 11 | TH-9 | 77.57831573 | 77.57831573 |  |
| 11 | TH-10 | 77.57831573 | 77.57831573 |  |
| 11 | TH-11 | 77.57831573 | 77.57831573 |  |
| 11 | TH-12 | 77.57831573 | 77.57831573 |  |
| 11 | OF'-1 | 77.57831573 | 77.57831573 |  |
| 11 | OF'-2 | 77.57831573 | 77.57831573 |  |
| 11 | OF'-3 | 77.57831573 | 77.57831573 |  |
| 11 | OF'-4 | 77.57831573 | 77.57831573 |  |
| 11 | OF'-5 | 77.57831573 | 77.57831573 |  |
| 11 | OF'-6 | 77.57831573 | 77.57831573 |  |
| 11 | OF'-7 | 77.57831573 | 77.57831573 |  |
| 11 | OF'-8 | 77.57831573 | 77.57831573 |  |
| 11 | OF'-9 | 77.57831573 | 77.57831573 |  |
| 11 | OF'-10 | 77.57831573 | 77.57831573 |  |
| 11 | OF'-11 | 77.57831573 | 77.57831573 |  |
| 11 | OF'-12 | 77.57831573 | 77.57831573 |  |
| 11 | B'-1 | 77.57831573 | 77.57831573 |  |
| 11 | B'-2 | 77.57831573 | 77.57831573 |  |
| 11 | B'-3 | 77.57831573 | 77.57831573 |  |
| 11 | B'-4 | 77.57831573 | 77.57831573 |  |
| 11 | B'-5 | 77.57831573 | 77.57831573 |  |
| 11 | B'-6 | 77.57831573 | 77.57831573 |  |
| 11 | B'-7 | 77.57831573 | 77.57831573 |  |
| 11 | B'-8 | 77.57831573 | 77.57831573 |  |
| 11 | TA'-1 | 77.57831573 | 77.57831573 |  |
| 11 | TA'-2 | 77.57831573 | 77.57831573 |  |
| 11 | TA'-3 | 77.57831573 | 77.57831573 |  |
| 11 | TA'-4 | 77.57831573 | 77.57831573 |  |
| 11 | TA'-5 | 77.57831573 | 77.57831573 |  |
| 11 | TA'-6 | 77.57831573 | 77.57831573 |  |
| 11 | TA'-7 | 77.57831573 | 77.57831573 |  |
| 11 | TA'-8 | 77.57831573 | 77.57831573 |  |
| 11 | TA'-9 | 77.57831573 | 77.57831573 |  |
| 11 | TA'-10 | 77.57831573 | 77.57831573 |  |
| 11 | TA'-11 | 77.57831573 | 77.57831573 |  |
| 11 | TA'-12 | 77.57831573 | 77.57831573 |  |
| 11 | TH'-1 | 77.57831573 | 77.57831573 |  |
| 11 | TH'-2 | 77.57831573 | 77.57831573 |  |
| 11 | TH'-3 | 77.57831573 | 77.57831573 |  |
| 11 | TH'-4 | 77.57831573 | 77.57831573 |  |
| 11 | TH'-5 | 77.57831573 | 77.57831573 |  |
| 11 | TH'-6 | 77.57831573 | 77.57831573 |  |
| 11 | TH'-7 | 77.57831573 | 77.57831573 |  |
| 11 | TH'-8 | 77.57831573 | 77.57831573 |  |
| 11 | TH'-9 | 77.57831573 | 77.57831573 |  |
| 11 | TH'-10 | 77.57831573 | 77.57831573 |  |
| 11 | TH'-11 | 77.57831573 | 77.57831573 |  |
| 11 | TH'-12 | 77.57831573 | 77.57831573 |  |
| 11 | P'-1 | 77.57831573 | 77.57831573 |  |
| 11 | P'-2 | 77.57831573 | 77.57831573 |  |
| 11 | P'-3 | 77.57831573 | 77.57831573 |  |
| 11 | P'-4 | 77.57831573 | 77.57831573 |  |
| 11 | P'-5 | 77.57831573 | 77.57831573 |  |
| 11 | P'-6 | 77.57831573 | 77.57831573 |  |
| 11 | P'-7 | 77.57831573 | 77.57831573 |  |
| 11 | P'-8 | 77.57831573 | 77.57831573 |  |

“Ground”, “Common”, and “Broken” meant Ground reference contact, Common reference contact, and Broken contact, respectively.

A:L-ORB→ACC, left orbital frontal cortex→anterior cingulate cortex;

B:L-Broca’s area→INS, left-Broca’s area→insula;

B':R-Broca’s area→INS, right-Broca’s area→insula;

C:L-PreCG→INS; left precentral gyrus→Insula;

FF:L-lFFG→mFFG, left lateral fusiform gyrus→medial fusiform gyrus;

IP:L-lPoCG→mPoCG, left-lateral postcentral gyrus→medial postcentral gyrus;

IP':R-lPoCG→mPoCG, right-lateral postcentral gyrus→medial postcentral gyrus;

O:L-lTPO→mTPO, left-lateral temporal occipital junction→medial temporal occipital junction;

OF:L-lORB→mORB, left-lateral orbital frontal cortex→medial orbital frontal cortex;

OF':R-lORB→mORB, right-lateral orbital frontal cortex→medial orbital frontal cortex;

OH:L-MOG→PreHIP, left-middle occipital gyrus→pre-hippocampus;

OH':R-MOG→PreHIP, right-middle occipital gyrus→pre-hippocampus;

P:L-lMTG→mMTG, left-lateral middle temporal gyrus→medial middle temporal gyrus;

P':R-lMTG→mMTG, right-lateral middle temporal gyrus→medial middle temporal gyrus;

P1:L-lPreMTG→mPreMTG, left lateral pre-middle temporal gyrus→medial pre-middle temporal gyrus;

P2:L-lPoMTG→mPoMTG, left lateral post-middle temporal gyrus→medial post-middle temporal gyrus;

P1':R-lPreMTG→mPreMTG, right-lateral pre-middle temporal gyrus→medial pre-middle temporal gyrus;

P2':R-lPoMTG→mPoMTG, right-lateral post-middle temporal gyrus→medial post-middle temporal gyrus;

PO:L-lSMG→mSMG, left-lateral supramarginal gyrus→medial supramarginal gyrus;

PO':R-lSMG→mSMG; right-lateral supramarginal gyrus→medial supramarginal gyrus;

PO1:L-lPreSMG→mPreSMG, left-lateral pre-supramarginal gyrus→medial pre-supramarginal gyrus;

PO1':R-lPreSMG→mPreSMG, right-lateral pre-supramarginal gyrus→medial pre-supramarginal gyrus;

PO2:L-lPoSMG→mPoSMG, left-lateral post-supramarginal gyrus→medial post-supramarginal gyrus;

PO2':R-lPoSMG→mPoSMG, right-lateral post-supramarginal gyrus→medial post-supramarginal gyrus;

SM:L-lSMA→mSMA, left-lateral supplementary motor area→medial supplementary motor area;

TA:L-MTG→AMY, left-middle temporal gyrus→amygdala;

TA':R-MTG→AMY, right-middle temporal gyrus→amygdala;

TA1:L-MTG→PreAMY, left-middle temporal gyrus→pre-amygdala;

TA1':R-MTG→PreAMY, right-middle temporal gyrus→pre-amygdala;

TA2:L-MTG→PoAMY, left-middle temporal gyrus→post-amygdala;

TA2':R-MTG→PoAMY, right-middle temporal gyrus→post-amygdala;

TH:L-MTG→HIP, left-middle temporal gyrus→hippocampus;

TH':R-MTG→HIP, right-middle temporal gyrus→hippocampus;

TH1:L-MTG→PreHIP, left-middle temporal gyrus→pre-hippocampus;

TH1':R-MTG→PreHIP, right-middle temporal gyrus→pre-hippocampus;

TO:L-lMOG→mMOG, left-lateral middle occipital gyrus→medial middle occipital gyrus;

V:L-lSPG→mSPG, left-lateral superior parietal gyrus→medial superior parietal gyrus;

V':R-lSPG→mSPG, right-lateral superior parietal gyrus→medial superior parietal gyrus;

W:L-lSTG→mSTG, left-lateral superior temporal gyrus→medial superior temporal gyrus.

Table S4. Local field potentials of all contacts in hippocampus, insula, and amygdala (uV)

| **Patient number** | **Contact** | **Stimulating currents** | | | | | | | | |
| --- | --- | --- | --- | --- | --- | --- | --- | --- | --- | --- |
|  |  | **0mA** | **1mA** | **3mA** | **5mA** | **7mA** | **9mA** | **11mA** | **13mA** | **15mA** |
| 1 | TH-1 | 3.0520182 | 30.099022 | 31.808615 | 62.457893 | 93.340691 | 126.08901 | 144.48213 | 185.8058 | 216.08475 |
| 1 | TH-2 | 3.2234592 | 29.870214 | 31.369013 | 61.629192 | 92.223671 | 124.06214 | 141.64243 | 181.44037 | 209.71329 |
| 1 | TH-3 | 2.9858546 | 29.750011 | 31.174997 | 61.459454 | 92.158791 | 124.66285 | 143.53261 | 184.46948 | 214.43188 |
| 1 | TH'-1 | 5.1265268 | 26.332708 | 29.038706 | 56.173721 | 83.781258 | 112.75741 | 128.45004 | 166.64267 | 200.49005 |
| 1 | TH'-2 | 5.2594109 | 25.70043 | 28.473232 | 54.40966 | 81.11541 | 108.68633 | 123.14835 | 159.47032 | 191.7843 |
| 1 | TH'-3 | 5.4942474 | 25.037149 | 27.904045 | 53.006252 | 79.40554 | 107.00594 | 122.51985 | 159.50517 | 194.21466 |
| 1 | TH'-4 | 5.3974094 | 24.05022 | 26.926945 | 51.369785 | 77.199791 | 103.76788 | 118.04688 | 153.52124 | 187.31032 |
| 1 | B-1 | 2.281297 | 17.235106 | 18.02387 | 34.286011 | 53.702629 | 74.230484 | 88.687958 | 112.4409 | 135.76262 |
| 1 | B-2 | 2.1175787 | 16.827578 | 17.570044 | 33.378231 | 52.451488 | 72.438599 | 86.459846 | 109.13592 | 131.379 |
| 1 | B'-1 | 6.9269876 | 7.4734812 | 9.6735754 | 13.567916 | 16.10244 | 17.45067 | 20.47888 | 26.451181 | 47.320183 |
| 1 | B'-2 | 7.0150447 | 7.3989272 | 9.4086351 | 12.903373 | 15.070273 | 16.066267 | 20.097017 | 24.054115 | 44.132813 |
| 1 | TA-1 | 3.1095958 | 24.671034 | 26.250996 | 51.075256 | 77.688103 | 105.85535 | 123.40961 | 159.26555 | 190.33113 |
| 1 | TA-2 | 2.8172579 | 24.634914 | 26.061468 | 50.825069 | 77.342903 | 105.51568 | 123.15855 | 158.63702 | 189.03036 |
| 1 | TA-3 | 2.9073734 | 24.673172 | 25.937105 | 50.59285 | 77.031868 | 105.02122 | 122.44695 | 157.36101 | 186.70714 |
| 1 | TA-4 | 2.5683131 | 24.715614 | 25.909157 | 50.632446 | 77.026436 | 104.97785 | 122.16785 | 156.68031 | 184.951 |
| 2 | TH'-1 | 4.0297694 | 5.2698655 | 26.164976 | 23.482281 | 355.97351 | 1871.9579 | 3645.3616 | 5245.8184 | 6864.3535 |
| 2 | TH'-2 | 3.329592 | 4.6311588 | 24.704041 | 27.186907 | 497.8353 | 2130.2317 | 3914.6121 | 5534.4136 | 7158.4053 |
| 2 | TH'-3 | 3.0019345 | 4.3255529 | 21.983629 | 32.894897 | 344.80508 | 1868.6333 | 3640.4453 | 5232.437 | 6846.772 |
| 2 | TH'-4 | 2.8777723 | 4.2028093 | 25.638111 | 23.100018 | 455.87207 | 2011.2151 | 3766.3088 | 5375.5889 | 7010.3037 |
| 2 | B'-1 | 2.7299783 | 3.4656413 | 18.137226 | 19.538034 | 78.71051 | 471.73294 | 1517.0195 | 2682.5374 | 3503.4885 |
| 2 | B'-2 | 2.6473527 | 3.3064778 | 16.767256 | 16.876678 | 54.767353 | 343.57053 | 1089.9619 | 2084.3884 | 3142.9619 |
| 2 | TA'-1 | 3.6962447 | 4.6447473 | 23.810595 | 19.254049 | 184.13776 | 1229.0288 | 2726.4329 | 4045.4182 | 5470.7441 |
| 2 | TA'-2 | 3.341831 | 4.3445306 | 21.939741 | 19.205856 | 180.99667 | 983.56134 | 2624.6628 | 3954.1465 | 5365.3818 |
| 2 | TA'-3 | 3.152936 | 4.1254034 | 22.041702 | 19.102619 | 247.47699 | 1556.8248 | 3001.3889 | 4342.877 | 5791.9502 |
| 3 | TH-1 | 1.0957377 | 1.459807 | 1.5341827 | 1.6791221 | 1.9913838 | 2.4176202 | 3.3763065 | 3.6070094 | 4.6006489 |
| 3 | TH-2 | 3.1874781 | 3.2745852 | 3.3058643 | 3.264523 | 3.3781357 | 3.4018941 | 3.7950861 | 3.5247684 | 3.5057371 |
| 3 | TH-3 | 2.1816216 | 2.5965753 | 2.66222 | 2.691051 | 2.8984401 | 3.1655297 | 3.4237914 | 3.6197641 | 3.6284635 |
| 3 | TH-4 | 2.5718212 | 2.5348551 | 2.5884039 | 2.6093454 | 2.8245776 | 3.0351541 | 3.3823178 | 3.6662943 | 3.7932875 |
| 3 | TH-5 | 1.546429 | 1.2496164 | 1.2736666 | 1.2698865 | 1.3277131 | 1.4431522 | 1.5027066 | 1.5689347 | 1.6359142 |
| 3 | B-1 | 1.5133102 | 2.6847625 | 7.5303626 | 16.988276 | 29.078411 | 44.177456 | 57.54089 | 68.230324 | 79.422073 |
| 3 | B-2 | 1.2098315 | 2.652379 | 7.6906576 | 17.399145 | 29.72941 | 45.189335 | 58.691334 | 69.478073 | 80.541069 |
| 3 | TA-1 | 1.1740829 | 1.6590532 | 3.0266795 | 6.0286574 | 10.091104 | 15.309774 | 19.67231 | 23.514772 | 27.111568 |
| 3 | TA-2 | 1.1775792 | 1.6305513 | 2.989949 | 6.0727787 | 10.165176 | 15.458087 | 20.042864 | 23.854202 | 27.779707 |
| 3 | TA-3 | 1.4406515 | 1.4472365 | 2.9253163 | 6.1447101 | 10.382136 | 15.822925 | 20.603651 | 24.558981 | 28.789854 |
| 3 | TA-4 | 1.1268442 | 1.4639223 | 2.916661 | 6.2392116 | 10.561049 | 16.068485 | 21.032982 | 25.086571 | 29.536366 |
| 4 | TH-1 | 3.3747723 | 5.2325439 | 11.838253 | 34.053558 | 82.219727 | 122.56654 | 145.71191 | 170.60204 | 253.40434 |
| 4 | TH-2 | 2.9408486 | 4.3586454 | 11.339203 | 33.711258 | 82.022133 | 123.02737 | 147.34393 | 173.4669 | 258.2059 |
| 4 | TH-3 | 2.8598461 | 4.3048415 | 11.297587 | 33.420979 | 81.209259 | 121.91001 | 146.3597 | 172.51566 | 256.68723 |
| 4 | TH-4 | 2.3390133 | 3.6856201 | 10.930576 | 33.03656 | 80.418465 | 120.52902 | 143.69269 | 167.71605 | 246.98798 |
| 4 | TH'-1 | 2.8197498 | 4.1506929 | 11.671747 | 34.674564 | 83.089874 | 121.42674 | 143.08363 | 169.85365 | 258.78433 |
| 4 | TH'-2 | 3.1905096 | 4.5001688 | 11.652594 | 34.439129 | 82.074471 | 119.26289 | 139.68666 | 164.71608 | 251.04991 |
| 4 | TH'-3 | 3.502279 | 4.8367825 | 11.72854 | 34.501671 | 81.389694 | 118.37173 | 139.5383 | 165.99698 | 255.09978 |
| 4 | TH'-4  (Broken) | 381.2681 | 140.11063 | 72.714912 | 144.34956 | 331.16147 | 529.70837 | 638.05347 | 772.65381 | 829.58734 |
| 4 | TH'-5 | 2.4117374 | 3.7279539 | 11.152408 | 33.546715 | 80.144592 | 116.63345 | 137.45554 | 164.96463 | 257.73404 |
| 5 | B-1 | 1.8922478 | 4.3976846 | 6.0584588 | 13.161912 | 47.715981 | 207.74281 | 496.97858 | 989.76361 | 1684.6036 |
| 5 | B-2 | 2.1305137 | 4.4706302 | 6.1493816 | 13.354481 | 49.248119 | 222.30046 | 577.52631 | 1131.15 | 1889.7935 |
| 5 | C-1 | 2.1057692 | 2.5575316 | 3.7867761 | 7.1008534 | 8.7366276 | 11.744663 | 12.984501 | 12.480097 | 14.484129 |
| 5 | C-2 | 1.954115 | 2.521939 | 3.5006354 | 6.6857581 | 8.1411114 | 10.708062 | 11.81665 | 11.536889 | 15.219328 |
| 5 | TA-1 | 1.7263923 | 2.0248823 | 2.123754 | 2.1967533 | 2.2931383 | 2.3756824 | 3.748683 | 3.0539401 | 3.2863042 |
| 5 | TA-2 | 1.5466216 | 1.87058 | 2.0000322 | 2.0535748 | 2.1672227 | 2.3533249 | 2.4268458 | 2.5637727 | 4.3151164 |
| 5 | TA-3 | 1.3794783 | 1.798355 | 1.9316969 | 1.9692289 | 2.0816753 | 2.2586677 | 2.3487124 | 2.6466753 | 4.3506575 |
| 5 | TA-4 | 1.0916158 | 1.5386019 | 1.7346196 | 1.7690257 | 1.878265 | 1.9085726 | 2.0280638 | 2.3231423 | 3.4826071 |
| 6 | TH-1 | 3.0605574 | 3.5939047 | 4.9360838 | 6.5753489 | 8.0431385 | 15.001668 | 31.510757 | 48.519855 | 62.358833 |
| 6 | TH-2 | 2.9324486 | 3.4037836 | 4.5237818 | 5.832305 | 7.6964908 | 16.44446 | 34.350357 | 51.681198 | 63.800957 |
| 6 | TH-3 | 2.0731463 | 2.6361637 | 3.8259025 | 5.0562921 | 7.4765639 | 17.330605 | 35.806267 | 53.726082 | 65.014824 |
| 6 | TH-4 | 1.4047668 | 1.6753229 | 3.0740175 | 4.4651937 | 7.0550141 | 17.194603 | 35.978535 | 54.004444 | 64.731102 |
| 6 | B-1 | 0.7629936 | 0.977355 | 5.9358792 | 9.7733088 | 28.396664 | 136.4547 | 436.96695 | 1154.3644 | 1160.4124 |
| 6 | B-2 | 0.9402477 | 1.1595507 | 6.2306333 | 10.579558 | 33.465755 | 130.02463 | 410.00488 | 763.29218 | 1074.7676 |
| 6 | B-3 | 1.2796711 | 1.4451301 | 6.656951 | 11.162054 | 34.602657 | 124.39786 | 380.15863 | 724.3689 | 1090.1487 |
| 6 | B-4 | 1.7790667 | 1.8026913 | 6.9903746 | 11.700933 | 37.366337 | 147.18201 | 459.48569 | 913.59161 | 1280.3529 |
| 6 | B-5 | 2.2636123 | 2.2657766 | 7.1730227 | 12.701725 | 44.075302 | 161.47801 | 480.60831 | 932.65363 | 1247.3218 |
| 6 | TA-1 | 1.1928973 | 1.4359084 | 1.5206887 | 1.6907194 | 2.7988992 | 8.6517229 | 19.265076 | 29.78159 | 42.893509 |
| 6 | TA-2 | 0.9170787 | 1.0689214 | 1.1142215 | 1.2760855 | 2.8476603 | 8.557271 | 18.285788 | 27.24452 | 37.731205 |
| 6 | TA-3 | 0.8491102 | 0.9775025 | 1.0284724 | 1.5598122 | 3.9840965 | 9.6040573 | 18.089022 | 25.939465 | 32.190804 |
| 7 | TH-1 | 1.3777566 | 6.9775829 | 8.7617941 | 14.637324 | 48.004505 | 175.6609 | 641.72229 | 1163.3032 | 1960.594 |
| 7 | TH-2 | 1.3792942 | 7.0126848 | 8.3048487 | 14.416173 | 52.444752 | 198.02817 | 709.52277 | 1246.3606 | 2065.335 |
| 7 | TH-3 | 1.2837658 | 7.8369617 | 7.8711004 | 14.571454 | 56.881351 | 193.43318 | 592.67108 | 1110.9135 | 1913.6512 |
| 7 | TH-4 | 1.3839786 | 7.0410261 | 8.2870922 | 14.488964 | 49.398495 | 198.42859 | 705.16003 | 1235.5627 | 2062.2742 |
| 7 | TH'-1 | 1.9335719 | 4.9142122 | 9.5235109 | 16.072735 | 37.539963 | 135.98016 | 435.29874 | 890.96942 | 1570.3436 |
| 7 | TH'-2 | 1.9410247 | 4.9329071 | 9.3344126 | 15.477513 | 40.576523 | 145.27585 | 418.07001 | 849.66357 | 1572.8528 |
| 7 | TH'-3 | 1.9364996 | 4.9606743 | 9.1259651 | 15.252475 | 41.422302 | 148.28972 | 470.04092 | 939.46704 | 1637.177 |
| 7 | TH'-4 | 1.9455882 | 4.9442673 | 9.3472481 | 15.464455 | 41.005363 | 147.9873 | 461.53064 | 930.24231 | 1629.4614 |
| 7 | TH'-5 | 1.9255133 | 4.9477534 | 9.4520597 | 16.156086 | 36.692657 | 139.20868 | 546.26917 | 1026.4584 | 1737.7246 |
| 7 | B-1 | 1.8122323 | 3.0237596 | 6.2318983 | 12.450269 | 14.077349 | 20.666862 | 60.870968 | 114.82282 | 238.82286 |
| 7 | B-2 | 1.7618539 | 3.0234807 | 5.7496181 | 11.114408 | 12.586406 | 22.242325 | 65.099854 | 122.57177 | 253.37428 |
| 7 | TA'-1 | 1.8085165 | 4.0692768 | 8.683012 | 16.620958 | 18.448141 | 62.417034 | 189.7892 | 457.70728 | 903.73627 |
| 7 | TA'-2 | 1.7116332 | 4.047379 | 8.7323856 | 16.895647 | 18.405701 | 57.556797 | 172.46178 | 341.14746 | 720.03229 |
| 7 | TA'-3 | 1.5863853 | 4.0369949 | 8.538578 | 16.423429 | 18.530455 | 59.539513 | 172.61877 | 328.27716 | 652.66418 |
| 8 | TH1-1 | 3.0297334 | 3.1844723 | 3.4378629 | 8.0337029 | 11.450951 | 15.598334 | 22.969786 | 29.316051 | 32.142231 |
| 8 | TH1-2 | 2.3306375 | 2.4295237 | 2.7272129 | 7.3625646 | 10.849833 | 14.74812 | 22.078665 | 28.909489 | 32.377636 |
| 8 | TH1-3 | 1.4948109 | 1.7710925 | 2.0179291 | 6.4464965 | 9.5997667 | 13.440301 | 20.333601 | 26.912386 | 30.556181 |
| 8 | TH1-4 | 0.9155224 | 1.0327563 | 1.3011378 | 5.7337785 | 8.3598108 | 12.239715 | 18.312176 | 23.437672 | 25.630026 |
| 8 | TH-1 | 2.3760893 | 2.5592475 | 2.9996884 | 9.3819809 | 13.676792 | 19.1504 | 28.448896 | 35.627426 | 37.528282 |
| 8 | TH-2 | 1.9002019 | 1.9731872 | 2.1888936 | 8.6452951 | 12.764685 | 18.18235 | 27.106344 | 34.423515 | 36.604862 |
| 8 | TH-3 | 1.1256217 | 1.3159865 | 1.5591414 | 8.0674839 | 11.867176 | 17.375078 | 25.927965 | 32.567562 | 34.250423 |
| 8 | TH-4 | 0.9071712 | 1.0411228 | 1.3124341 | 7.7983131 | 11.5239 | 17.064243 | 25.631083 | 32.703281 | 34.79776 |
| 8 | TA1-1 | 0.8807442 | 1.0966763 | 1.2654212 | 1.9969877 | 2.5889275 | 2.3498271 | 3.0457442 | 3.1547606 | 3.1608045 |
| 8 | TA1-2 | 1.0324012 | 1.185781 | 1.423299 | 1.8148301 | 2.4306183 | 2.2864866 | 2.601084 | 2.6281729 | 2.5969968 |
| 8 | TA1-3 | 0.9885537 | 1.0391583 | 1.3054131 | 1.345942 | 1.6917733 | 1.6882802 | 1.627166 | 2.1066985 | 2.6020379 |
| 8 | TA1-4 | 1.1652496 | 1.029588 | 1.2705063 | 1.3004323 | 1.3932387 | 1.3946655 | 1.3866994 | 1.5095005 | 2.5362244 |
| 8 | TA2-1 | 1.837806 | 3.6570122 | 3.8789351 | 5.329535 | 7.3494668 | 8.5601444 | 12.30883 | 15.786714 | 18.034651 |
| 8 | TA2-2 | 1.4576142 | 2.5568385 | 2.7042501 | 4.2803535 | 5.8801246 | 7.1189642 | 10.198298 | 12.317611 | 13.693 |
| 8 | TA2-3 | 1.0958265 | 1.5406408 | 1.6230763 | 3.6179764 | 4.9818439 | 6.6189766 | 9.5535307 | 11.624957 | 12.753326 |
| 9 | TH1'-1 | 3.469229 | 3.7579539 | 7.767293 | 12.704734 | 22.181265 | 21.161831 | 21.952837 | 40.409435 | 45.237583 |
| 9 | TH1'-2 | 2.1209707 | 2.3518784 | 6.771317 | 12.926753 | 23.246565 | 22.421278 | 23.188011 | 42.765659 | 48.400501 |
| 9 | TH1'-3 | 1.3551853 | 1.4670526 | 6.0070662 | 13.707499 | 24.643887 | 23.879129 | 24.466887 | 45.290161 | 51.691368 |
| 9 | TH'-1 | 4.0139141 | 4.6838908 | 6.6738834 | 9.643692 | 15.965556 | 15.134287 | 15.764327 | 28.06469 | 31.246853 |
| 9 | TH'-2 | 4.2410278 | 4.4895892 | 6.8356099 | 10.364165 | 17.367689 | 16.568924 | 17.23122 | 30.748119 | 34.734131 |
| 9 | TH'-3 | 1.8415757 | 2.0009046 | 5.4871297 | 9.9715643 | 17.831121 | 17.176634 | 17.692352 | 32.384499 | 36.944141 |
| 9 | TH'-4 | 1.0692645 | 1.1251513 | 4.3710303 | 10.07428 | 18.137913 | 17.542175 | 18.025385 | 33.38414 | 38.183445 |
| 9 | TA1'-1 | 1.3220658 | 1.5750853 | 8.1155052 | 19.145346 | 34.045696 | 32.842915 | 33.462944 | 61.581799 | 69.852737 |
| 9 | TA1'-2 | 1.4876574 | 1.6269386 | 8.6587582 | 20.029039 | 35.697876 | 34.569447 | 35.184036 | 64.809204 | 73.727539 |
| 9 | TA1'-3 | 1.5019705 | 1.5695235 | 8.7254772 | 20.672047 | 36.874531 | 35.801773 | 36.351467 | 66.90818 | 75.969482 |
| 9 | TA2'-1 | 1.9297162 | 2.0266228 | 7.4363732 | 15.960215 | 28.46183 | 27.399488 | 28.063139 | 51.857365 | 58.731918 |
| 9 | TA2'-2 | 1.6262327 | 1.8637662 | 7.7058935 | 16.312597 | 29.201015 | 28.194805 | 28.843691 | 53.24297 | 60.530449 |
| 9 | TA2'-3 | 1.4241428 | 1.5128582 | 7.2082062 | 16.462893 | 29.544624 | 28.621368 | 29.290287 | 54.15918 | 61.77544 |
| 10 | TH-1 | 1.7871234 | 2.5795031 | 2.5788045 | 3.1712685 | 6.6910248 | 14.015043 | 26.627285 | 43.50106 | 67.448532 |
| 10 | TH-2 | 1.835674 | 2.6995685 | 2.7550364 | 3.461091 | 7.8717823 | 16.772736 | 31.705925 | 51.35714 | 76.893875 |
| 10 | TH-3 | 1.7268908 | 2.5647442 | 2.6609008 | 3.4870455 | 7.7204046 | 16.232521 | 30.133188 | 48.37149 | 73.205536 |
| 10 | TH-4 | 1.4788522 | 2.4414613 | 2.4685924 | 2.9048522 | 7.1759763 | 16.973314 | 33.529278 | 55.289391 | 81.606773 |
| 10 | TH'-1 | 3.1144032 | 3.7316997 | 3.946187 | 4.5430474 | 8.1664629 | 14.523757 | 26.438736 | 43.483402 | 71.282593 |
| 10 | TH'-2 | 2.926291 | 3.6714888 | 3.6825004 | 4.5931921 | 9.6542454 | 17.430914 | 30.973284 | 49.994774 | 79.720665 |
| 10 | TH'-3 | 3.4899054 | 3.7727187 | 3.9191589 | 5.0508699 | 10.36219 | 18.987032 | 33.828228 | 52.958843 | 83.096298 |
| 10 | TH'-4 | 3.3820193 | 3.5492082 | 3.7690306 | 4.9919586 | 10.520561 | 18.842506 | 33.139004 | 52.463188 | 82.389557 |
| 10 | B-1 | 1.6489947 | 2.6110938 | 10.202686 | 17.932276 | 24.379534 | 34.010357 | 45.374428 | 33.476414 | 488.4614 |
| 10 | B-2 | 1.5751939 | 2.6541846 | 10.4132 | 18.621668 | 25.316872 | 28.791929 | 12.195176 | 110.11466 | 375.20233 |
| 10 | B'-1 | 1.9541632 | 3.0991061 | 14.096375 | 24.438934 | 27.756321 | 42.996151 | 277.99106 | 1063.3866 | 1902.717 |
| 10 | B'-2 | 1.8726778 | 3.0053461 | 14.502569 | 24.407249 | 25.911375 | 139.77176 | 913.84943 | 1695.7596 | 2563.6309 |
| 11 | TH-1 | 2.8074827 | 3.0981655 | 3.9956083 | 7.7832756 | 11.697987 | 17.324083 | 21.795712 | 24.375456 | 28.048853 |
| 11 | TH-2 | 2.3896799 | 2.6883388 | 3.9709504 | 7.391489 | 10.948184 | 16.070431 | 20.307709 | 22.97393 | 26.25561 |
| 11 | TH-3 | 3.2336669 | 3.6591897 | 4.3219147 | 7.6568751 | 11.034304 | 15.963704 | 20.183109 | 23.029375 | 26.889154 |
| 11 | TH-4 | 1.209334 | 1.1869979 | 2.9547753 | 6.0986576 | 9.6008234 | 14.031511 | 17.856367 | 20.545347 | 23.589495 |
| 11 | TH'-1 | 2.4864454 | 3.2149408 | 2.7119377 | 4.9409575 | 7.5696864 | 12.120289 | 15.208089 | 16.209547 | 18.791584 |
| 11 | TH'-2 | 2.8133881 | 2.9808397 | 3.3011816 | 5.229784 | 6.6985641 | 10.58458 | 13.215651 | 14.433052 | 16.300653 |
| 11 | TH'-3 | 3.9971035 | 4.701776 | 4.9189777 | 6.308023 | 7.0757275 | 10.770644 | 12.65058 | 14.173694 | 15.534707 |
| 11 | TH'-4 | 2.9952142 | 3.3065338 | 3.3963623 | 4.4396305 | 5.7731886 | 9.0982561 | 11.342988 | 13.195586 | 14.323326 |
| 11 | B'-1 | 1.2427893 | 1.8458878 | 14.53485 | 27.578533 | 40.682854 | 56.39933 | 71.60939 | 85.633026 | 96.854233 |
| 11 | B'-2 | 1.7979231 | 2.2307541 | 15.20415 | 28.849174 | 42.618835 | 59.129768 | 74.98204 | 89.535156 | 100.96301 |
| 11 | B'-3 | 2.2403643 | 2.7273467 | 15.694064 | 29.724018 | 43.826302 | 60.88879 | 77.172142 | 92.35466 | 103.92228 |
| 11 | TA-1 | 2.4015682 | 2.6407015 | 2.7682674 | 2.9542615 | 2.3102853 | 2.8028419 | 3.0844302 | 2.8284311 | 2.9289753 |
| 11 | TA-2 | 1.3460952 | 1.5386952 | 1.5205904 | 1.6115824 | 1.5222474 | 1.5255868 | 1.6682431 | 2.1015255 | 2.4134903 |
| 11 | TA-3 | 1.5116313 | 1.7460188 | 1.6405232 | 2.0459836 | 1.7391282 | 1.7507224 | 1.8932341 | 2.3753707 | 2.8444917 |
| 11 | TA'-1 | 1.8547301 | 2.3500934 | 4.216929 | 6.1012325 | 7.5213213 | 9.331316 | 11.525078 | 15.721609 | 17.442516 |
| 11 | TA'-2 | 1.7068461 | 1.9415032 | 4.7716746 | 7.2950106 | 9.3067398 | 11.785804 | 14.739962 | 19.611372 | 21.810783 |
| 11 | TA'-3 | 1.600345 | 1.7889729 | 5.3208342 | 8.374383 | 10.980069 | 14.097387 | 17.67487 | 23.359825 | 25.950811 |
| 11 | TA'-4 | 1.4059166 | 1.6649706 | 5.6847796 | 9.1758547 | 12.155159 | 15.722183 | 19.779316 | 25.765005 | 28.690672 |

“Broken” meant Broken contact.

Left hippocampus included TH: L-MTG→HIP and TH1: L-MTG→PreHIP;

Right hippocampus included TH': R-MTG→HIP and TH1': R-MTG→PreHIP;

Left insula included B: L-Broca’s area→INS and C: L-PreCG→INS;

Right insula had B': R-Broca’s area→INS;

Left amygdala had TA: L-MTG→AMY, TA1: L-MTG→PreAMY, and TA2: L-MTG→PoAMY;

Right amygdala included TA': R-MTG→AMY, TA1': R-MTG→PreAMY, and TA2': R-MTG→PoAMY.

B:L-Broca’s area→INS, left-Broca’s area→insula;

B':R-Broca’s area→INS, right-Broca’s area→insula;

C:L-PreCG→INS; left precentral gyrus→Insula;

TA:L-MTG→AMY, left-middle temporal gyrus→amygdala;

TA':R-MTG→AMY, right-middle temporal gyrus→amygdala;

TA1:L-MTG→PreAMY, left-middle temporal gyrus→pre-amygdala;

TA1':R-MTG→PreAMY, right-middle temporal gyrus→pre-amygdala;

TA2:L-MTG→PoAMY, left-middle temporal gyrus→post-amygdala;

TA2':R-MTG→PoAMY, right-middle temporal gyrus→post-amygdala;

TH:L-MTG→HIP, left-middle temporal gyrus→hippocampus;

TH':R-MTG→HIP, right-middle temporal gyrus→hippocampus;

TH1:L-MTG→PreHIP, left-middle temporal gyrus→pre-hippocampus;

TH1':R-MTG→PreHIP, right-middle temporal gyrus→pre-hippocampus.

Table S5. Local field potentials of other contacts except for hippocampus, insula, and amygdala (uV)

| **patient number** | **contact** | **Stimulating currents** | | | | | | | | |
| --- | --- | --- | --- | --- | --- | --- | --- | --- | --- | --- |
|  |  | **0mA** | **1mA** | **3mA** | **5mA** | **7mA** | **9mA** | **11mA** | **13mA** | **15mA** |
| 1 | OF-1 | 2.7587743 | 5.7608981 | 6.949204 | 10.332477 | 17.697853 | 24.755026 | 28.013308 | 33.00127 | 48.266476 |
| 1 | OF-2 | 2.5770667 | 5.2447276 | 6.2951012 | 9.0027266 | 15.926199 | 22.476532 | 25.58172 | 29.320162 | 43.73959 |
| 1 | OF-3 | 2.5328803 | 5.0819244 | 6.1018839 | 8.3516836 | 15.060071 | 21.29273 | 24.298346 | 27.273455 | 40.957321 |
| 1 | OF-4 | 2.2455597 | 4.6935821 | 5.614809 | 7.4305606 | 13.926983 | 19.912947 | 22.803843 | 25.045494 | 37.852623 |
| 1 | OF-5 | 2.2411571 | 4.2187929 | 5.0338502 | 6.0786891 | 12.25703 | 17.873974 | 20.867622 | 21.973938 | 33.682442 |
| 1 | OF-6 | 2.3521202 | 3.9029582 | 4.703968 | 5.0564971 | 10.890022 | 16.20829 | 19.315237 | 19.505621 | 30.485914 |
| 1 | OF-7 | 2.4081686 | 3.3746521 | 4.1032572 | 3.724514 | 9.1243782 | 13.913528 | 16.749847 | 15.848316 | 25.446522 |
| 1 | OF-8 | 2.4202931 | 2.9776382 | 3.7176132 | 2.7500372 | 7.5906277 | 12.071115 | 15.010714 | 13.281816 | 21.94046 |
| 1 | OF-9 | 2.2989488 | 2.051264 | 2.7649822 | 2.2701817 | 5.0177674 | 9.1236258 | 11.833802 | 10.035887 | 16.73065 |
| 1 | OF-10 | 2.2301111 | 2.0786347 | 2.522135 | 5.9177785 | 5.39784 | 8.6232386 | 9.888319 | 12.538876 | 12.445061 |
| 1 | OF-11 | 2.3915448 | 3.4219234 | 3.6515982 | 9.7634802 | 9.6703806 | 13.106376 | 13.424204 | 20.866249 | 18.197546 |
| 1 | OF-12 | 2.4712522 | 4.0264349 | 4.1839933 | 11.291272 | 11.26209 | 14.807161 | 14.763692 | 22.686697 | 19.631153 |
| 1 | OF-13 | 2.4699624 | 4.3834934 | 4.5250282 | 12.146394 | 12.196191 | 15.716499 | 15.445181 | 23.147013 | 20.323301 |
| 1 | OF-14 | 2.3655021 | 4.8144584 | 4.9509892 | 13.382252 | 13.515878 | 17.01734 | 16.409985 | 23.353142 | 21.034697 |
| 1 | OF-15 | 2.6604433 | 16.264006 | 16.390038 | 39.870411 | 45.33786 | 53.533695 | 47.440407 | 65.366859 | 54.440895 |
| 1 | OF-16 | 6.06811 | 47.274986 | 52.184071 | 100.51208 | 74.856514 | 300.41733 | 1009.5962 | 296.60608 | 340.26489 |
| 1 | A-1 | 2.2167404 | 5.1453686 | 6.1760921 | 9.2386265 | 15.723513 | 21.952246 | 25.226936 | 29.367554 | 43.269276 |
| 1 | A-2 | 2.1572387 | 4.8457613 | 5.7886572 | 8.3044529 | 14.466537 | 20.300781 | 23.465017 | 26.542658 | 39.662647 |
| 1 | A-3 | 1.9415646 | 4.4658117 | 5.3548884 | 7.4278984 | 13.426881 | 18.878862 | 21.876289 | 24.358292 | 36.484322 |
| 1 | A-4 | 1.9175318 | 4.421248 | 5.2618694 | 7.1790075 | 13.351947 | 18.856785 | 22.387552 | 24.605211 | 36.41753 |
| 1 | A-5 | 1.9736016 | 4.4044685 | 5.2297606 | 6.9434171 | 13.30544 | 18.787331 | 22.562691 | 24.368837 | 35.563332 |
| 1 | A-6 | 1.9344375 | 4.145812 | 4.9031706 | 6.1572256 | 12.466598 | 17.870928 | 21.965977 | 23.401531 | 34.103405 |
| 1 | A-7 | 1.898327 | 3.5644834 | 4.2706456 | 4.6186461 | 10.586032 | 15.520496 | 19.595407 | 19.678961 | 29.23209 |
| 1 | A-8 | 1.8532648 | 2.9150975 | 3.5714979 | 2.9269428 | 8.253561 | 12.567372 | 16.492561 | 15.054337 | 22.924927 |
| 1 | A-9 | 2.1313684 | 2.6903307 | 3.370739 | 2.4239767 | 6.9095573 | 10.994816 | 14.99105 | 13.15608 | 20.279276 |
| 1 | A-10 | 2.2905111 | 2.9684346 | 3.5349751 | 2.6658356 | 7.441154 | 11.671917 | 15.99693 | 14.169182 | 21.296419 |
| 1 | A-11 | 2.0144122 | 2.8320417 | 3.441556 | 2.6506262 | 7.9694757 | 12.553787 | 17.311863 | 15.791227 | 23.423044 |
| 1 | A-12 | 2.0991819 | 2.9613557 | 3.6113107 | 2.6339374 | 7.7065859 | 12.139288 | 16.808084 | 14.957264 | 21.89962 |
| 1 | B-3 | 2.4916306 | 16.449541 | 17.128202 | 32.427807 | 51.019588 | 70.310326 | 83.771156 | 105.55196 | 126.78521 |
| 1 | B-4 | 2.1428986 | 16.107555 | 16.721107 | 31.634996 | 49.984497 | 68.988922 | 82.567001 | 103.7616 | 124.57552 |
| 1 | B-5 | 2.0717754 | 15.937505 | 16.503462 | 31.193844 | 49.366806 | 68.088158 | 81.568871 | 102.22094 | 122.41323 |
| 1 | B-6 | 2.1230597 | 15.550871 | 16.101913 | 30.332687 | 48.146267 | 66.496101 | 79.851364 | 99.796959 | 119.44707 |
| 1 | B-7 | 2.0637023 | 14.921219 | 15.425953 | 28.850199 | 45.969086 | 63.305401 | 75.718246 | 93.963638 | 111.96683 |
| 1 | B-8 | 2.0644448 | 14.491615 | 14.924838 | 27.83427 | 44.791195 | 62.175987 | 75.323235 | 93.45417 | 112.11194 |
| 1 | B-9 | 1.8597227 | 13.913826 | 14.308201 | 26.537743 | 43.117367 | 60.077709 | 73.185333 | 90.376205 | 108.36263 |
| 1 | B-10 | 2.046926 | 11.386589 | 11.631784 | 20.394562 | 36.256222 | 52.995201 | 68.061974 | 81.89373 | 97.207237 |
| 1 | TA-5 | 2.4091067 | 24.683268 | 25.781673 | 50.429436 | 76.841301 | 104.95316 | 122.52059 | 157.19914 | 185.38228 |
| 1 | TA-6 | 2.2716537 | 24.854334 | 25.816416 | 50.689133 | 77.490295 | 106.49479 | 125.51203 | 161.35165 | 190.81372 |
| 1 | TA-7 | 2.1699052 | 24.990549 | 25.827785 | 50.762966 | 77.365128 | 105.81748 | 123.70188 | 158.26817 | 185.12227 |
| 1 | TA-8 | 2.2127237 | 25.502586 | 26.198875 | 51.657494 | 78.764427 | 107.95065 | 126.52266 | 161.86307 | 188.7235 |
| 1 | TA-9 | 2.3563602 | 25.825024 | 26.473429 | 52.3274 | 79.707878 | 109.29186 | 128.16139 | 163.93419 | 190.69518 |
| 1 | TA-10 | 2.3070104 | 26.377682 | 26.987404 | 53.425861 | 81.389999 | 111.65129 | 131.02437 | 167.56956 | 194.22418 |
| 1 | TA-11 | 2.1920929 | 27.427677 | 27.893545 | 55.502647 | 84.164597 | 114.93861 | 133.99449 | 170.72864 | 195.71841 |
| 1 | TA-12 | 1.9255737 | 29.121214 | 29.39481 | 58.938934 | 88.938507 | 121.08597 | 140.6644 | 178.53119 | 201.90945 |
| 1 | SM-1 | 2.3260515 | 10.607068 | 11.587404 | 21.230577 | 33.157249 | 45.141457 | 54.002899 | 67.632576 | 85.026161 |
| 1 | SM-2 | 1.7997634 | 10.121894 | 11.06216 | 20.097652 | 31.614122 | 42.878201 | 51.241207 | 63.653851 | 79.847679 |
| 1 | SM-3 | 1.6951993 | 9.7600861 | 10.652347 | 19.200857 | 30.542162 | 41.524242 | 49.973873 | 61.685635 | 77.208267 |
| 1 | SM-4 | 1.7276406 | 9.3513908 | 10.164775 | 18.122988 | 29.237677 | 39.938736 | 48.618439 | 59.606926 | 74.636642 |
| 1 | SM-5 | 2.0431061 | 8.9763861 | 9.7207508 | 16.993496 | 27.701267 | 37.824574 | 46.197742 | 56.106075 | 69.984741 |
| 1 | SM-6 | 2.0620937 | 8.8437843 | 9.5266323 | 16.486507 | 27.051466 | 37.094791 | 45.61665 | 55.118027 | 68.825066 |
| 1 | SM-7 | 1.8919241 | 8.7289295 | 9.4013472 | 16.28175 | 26.777279 | 36.752918 | 45.165745 | 54.531849 | 67.936554 |
| 1 | SM-8 | 1.6030258 | 8.2397718 | 8.8623257 | 15.323668 | 25.58993 | 35.12537 | 43.541161 | 52.260681 | 65.090744 |
| 1 | TH-4 | 2.1589434 | 29.776525 | 31.025675 | 61.314957 | 92.034988 | 124.60883 | 143.27759 | 184.02435 | 213.09505 |
| 1 | TH-5 | 2.0703337 | 29.972281 | 31.10026 | 61.589912 | 92.249741 | 124.52881 | 142.53458 | 182.46802 | 209.49553 |
| 1 | TH-6 | 2.2436662 | 30.207163 | 31.252796 | 62.10854 | 93.207863 | 126.47169 | 146.04514 | 187.3783 | 215.91739 |
| 1 | TH-7 | 1.9820225 | 29.939405 | 30.820005 | 61.359905 | 92.216522 | 125.40512 | 145.27242 | 186.36086 | 214.53142 |
| 1 | TH-8 | 1.9371558 | 30.224573 | 30.981304 | 61.825424 | 92.676559 | 125.67527 | 144.84953 | 185.1351 | 211.17223 |
| 1 | TH-9 | 2.0191054 | 30.806393 | 31.472607 | 63.029003 | 94.449104 | 128.35452 | 148.35519 | 189.73187 | 216.03146 |
| 1 | TH-10 | 2.3108907 | 31.346096 | 31.893272 | 64.060234 | 96.039337 | 130.74644 | 151.57298 | 193.87326 | 220.40895 |
| 1 | TH-11 | 2.2536883 | 31.819956 | 32.24696 | 64.852165 | 96.917 | 131.31458 | 151.37827 | 192.93253 | 217.23732 |
| 1 | TH-12 | 1.8297899 | 33.664036 | 33.966331 | 68.85672 | 102.40775 | 138.20595 | 158.49609 | 200.96935 | 222.89789 |
| 1 | PO-1 | 2.0555475 | 23.379606 | 24.261204 | 47.557388 | 72.352509 | 98.989578 | 116.17084 | 149.01048 | 174.89493 |
| 1 | PO-2 | 1.8382268 | 23.528162 | 24.39069 | 47.848854 | 72.950073 | 100.1471 | 118.00848 | 151.48508 | 177.76936 |
| 1 | PO-3 | 2.0793529 | 23.662624 | 24.436255 | 48.016544 | 73.225616 | 100.43182 | 118.36465 | 151.79477 | 177.92 |
| 1 | PO-4 | 2.1675389 | 24.25738 | 25.012299 | 49.164661 | 74.765572 | 102.21546 | 119.86948 | 153.4474 | 178.5564 |
| 1 | PO-5 | 2.2488372 | 24.747309 | 25.477961 | 50.16729 | 76.231911 | 104.34589 | 122.47127 | 156.77979 | 181.91618 |
| 1 | PO-6 | 1.9672295 | 24.89649 | 25.554226 | 50.511757 | 76.660652 | 104.89417 | 122.98407 | 157.28133 | 181.80513 |
| 1 | PO-7 | 2.0035601 | 25.076223 | 25.67211 | 50.868542 | 77.329956 | 106.2969 | 125.30283 | 160.53421 | 186.0215 |
| 1 | PO-8 | 1.7364465 | 26.524906 | 26.873838 | 53.623463 | 81.123634 | 111.11372 | 129.96466 | 165.73656 | 188.71486 |
| 1 | IP-1 | 1.9450616 | 26.249678 | 27.362509 | 53.863377 | 81.295837 | 110.81892 | 128.94177 | 165.94177 | 193.65823 |
| 1 | IP-2 | 2.2569554 | 26.393579 | 27.404106 | 54.033897 | 81.534233 | 111.28835 | 129.61668 | 166.67226 | 193.94441 |
| 1 | IP-3 | 2.1753719 | 26.414328 | 27.342087 | 53.946182 | 81.536781 | 111.40606 | 129.9801 | 167.11928 | 194.22368 |
| 1 | IP-4 | 1.8780432 | 26.489807 | 27.389439 | 54.195591 | 82.011665 | 112.47461 | 131.90674 | 169.87703 | 197.96706 |
| 1 | IP-5 | 2.0259721 | 26.699781 | 27.548105 | 54.488697 | 82.304626 | 112.51713 | 131.39699 | 168.90404 | 195.83014 |
| 1 | IP-6 | 1.7965428 | 27.337574 | 28.08399 | 55.772156 | 84.154373 | 115.08862 | 134.44443 | 172.72823 | 199.55978 |
| 1 | IP-7 | 1.8578193 | 27.871336 | 28.523851 | 56.801285 | 85.649475 | 117.19641 | 136.98303 | 175.93542 | 202.68837 |
| 1 | IP-8 | 1.9996612 | 28.842075 | 29.375866 | 58.760883 | 88.554222 | 121.30658 | 142.12337 | 182.67596 | 209.99409 |
| 1 | P-1 | 1.6610866 | 29.489979 | 30.675459 | 60.878872 | 90.563576 | 122.53207 | 140.85687 | 182.00661 | 209.64038 |
| 1 | P-2 | 1.7745433 | 29.737154 | 30.857609 | 61.354492 | 91.370743 | 123.88444 | 142.89386 | 184.67329 | 212.63808 |
| 1 | P-3 | 1.692075 | 29.988926 | 31.097078 | 61.90694 | 92.335175 | 125.69186 | 145.79459 | 188.91888 | 218.26202 |
| 1 | P-4 | 1.8224803 | 30.529919 | 31.560484 | 62.909615 | 93.451347 | 126.49949 | 145.42249 | 187.51866 | 214.39491 |
| 1 | P-5 | 1.9726734 | 30.739504 | 31.718161 | 63.267838 | 93.998268 | 127.20853 | 146.07249 | 188.25981 | 214.56148 |
| 1 | P-6 | 1.847006 | 30.83531 | 31.762884 | 63.468361 | 94.328239 | 127.80373 | 147.25127 | 189.90218 | 216.6221 |
| 1 | P-7 | 1.7411846 | 31.017603 | 31.907074 | 63.812332 | 94.807228 | 128.53563 | 148.1662 | 191.17076 | 217.80731 |
| 1 | P-8 | 1.6057684 | 31.67663 | 32.513702 | 65.154213 | 96.488678 | 130.38593 | 149.36108 | 191.93407 | 216.33098 |
| 1 | OF'-1 | 3.2850752 | 7.5630083 | 9.0544853 | 15.456375 | 24.091951 | 32.795715 | 36.899551 | 45.986526 | 65.324417 |
| 1 | OF'-2 | 3.1008625 | 7.1003423 | 8.5787964 | 14.462566 | 22.484489 | 30.550201 | 33.997864 | 42.160892 | 60.754726 |
| 1 | OF'-3 | 2.8396509 | 6.6796575 | 8.145299 | 13.593102 | 21.127144 | 28.737617 | 31.850172 | 39.420856 | 57.736649 |
| 1 | OF'-4 | 2.6399519 | 6.0513 | 7.5550685 | 12.56528 | 19.32922 | 26.004246 | 28.234301 | 34.861584 | 52.601971 |
| 1 | OF'-5 | 2.6781099 | 5.527514 | 7.0367284 | 11.465017 | 17.436209 | 23.265696 | 24.859715 | 30.745451 | 48.294529 |
| 1 | OF'-6 | 2.7737632 | 4.8698773 | 6.2761164 | 9.9078445 | 14.77998 | 19.568834 | 20.581411 | 24.895397 | 41.782192 |
| 1 | OF'-7 | 2.6349468 | 4.2925582 | 5.5228138 | 8.2550955 | 12.309254 | 16.278315 | 16.518196 | 19.833773 | 35.942165 |
| 1 | OF'-8 | 2.4048371 | 3.9325578 | 5.1781626 | 7.7180219 | 11.351238 | 14.977087 | 15.053843 | 17.919319 | 33.788021 |
| 1 | OF'-9 | 2.1982439 | 3.5287709 | 4.7505617 | 7.1881838 | 10.455247 | 13.646009 | 13.382508 | 15.826576 | 31.37775 |
| 1 | OF'-10 | 2.1472433 | 3.1027067 | 4.1722026 | 6.1182742 | 8.7955503 | 11.576106 | 11.199415 | 13.038529 | 28.365559 |
| 1 | OF'-11 | 2.0781338 | 2.4836507 | 3.0233412 | 3.6965711 | 5.1732774 | 7.3438377 | 6.6118231 | 7.0548635 | 19.314194 |
| 1 | OF'-12 | 2.1951721 | 2.6110723 | 2.5102444 | 2.5918493 | 3.8644168 | 6.5556688 | 7.1945124 | 8.5820665 | 12.441556 |
| 1 | OF'-13 | 7.0165501 | 13.552155 | 10.514394 | 17.588535 | 29.175903 | 41.574898 | 57.811829 | 52.300644 | 35.968124 |
| 1 | OF'-14 | 7.0812197 | 14.077079 | 10.9756 | 18.670322 | 30.872915 | 43.759712 | 59.731728 | 54.46719 | 37.702923 |
| 1 | OF'-15 | 6.9707513 | 14.822907 | 11.612618 | 20.011847 | 33.071796 | 46.390266 | 61.664433 | 56.086437 | 37.922031 |
| 1 | OF'-16 | 6.9877963 | 19.44521 | 16.027037 | 28.440485 | 45.481026 | 59.507271 | 68.584435 | 59.122112 | 35.72464 |
| 1 | B'-3 | 7.1105032 | 7.306953 | 9.2363663 | 12.445518 | 14.406075 | 15.064303 | 19.98303 | 22.077835 | 41.656513 |
| 1 | B'-4 | 6.9467564 | 7.2393856 | 8.8883495 | 11.733395 | 13.350737 | 13.697486 | 19.945246 | 19.269901 | 37.86097 |
| 1 | B'-5 | 7.0220919 | 7.2153311 | 8.5952568 | 11.001081 | 12.346032 | 12.520362 | 20.09161 | 17.157055 | 35.149223 |
| 1 | B'-6 | 6.856091 | 7.2539439 | 8.2665482 | 10.042123 | 10.978827 | 11.135586 | 20.915543 | 13.763807 | 29.565102 |
| 1 | B'-7 | 7.0327172 | 7.3422747 | 8.0050573 | 9.3311577 | 10.061653 | 10.573293 | 21.822639 | 11.705912 | 25.757238 |
| 1 | B'-8 | 6.9169946 | 7.4350529 | 7.8014579 | 8.8915882 | 9.4489412 | 10.327625 | 22.600346 | 10.563637 | 22.753117 |
| 1 | TH'-5 | 2.2231939 | 23.066097 | 26.072117 | 50.08485 | 75.543373 | 102.35464 | 117.76696 | 153.93791 | 190.28093 |
| 1 | TH'-6 | 2.138592 | 22.460005 | 25.531399 | 48.81815 | 73.410736 | 98.669983 | 112.08025 | 145.94611 | 180.05246 |
| 1 | TH'-7 | 2.155617 | 21.780268 | 24.938011 | 47.587681 | 71.564453 | 96.308266 | 109.50449 | 142.77696 | 176.96996 |
| 1 | TH'-8 | 2.0487285 | 20.698444 | 23.917004 | 45.593628 | 68.876671 | 93.111191 | 106.8918 | 139.98068 | 175.81631 |
| 1 | TH'-9 | 2.1556549 | 20.06382 | 23.356316 | 44.32452 | 66.987282 | 90.431458 | 103.47018 | 135.586 | 171.14076 |
| 1 | TH'-10 | 2.3134806 | 19.690607 | 23.007282 | 43.629223 | 65.992607 | 88.867996 | 101.65902 | 133.18471 | 168.55824 |
| 1 | TH'-11 | 2.3215783 | 19.269667 | 22.605778 | 42.741661 | 64.602119 | 86.939499 | 99.307991 | 130.26573 | 165.61395 |
| 1 | TH'-12 | 2.2884791 | 18.907064 | 22.342106 | 42.069916 | 63.503777 | 85.020966 | 96.208542 | 125.80691 | 159.92401 |
| 1 | IP'-1 | 1.9615009 | 21.486439 | 24.05649 | 46.21035 | 69.488632 | 93.591019 | 107.48505 | 140.04126 | 172.08235 |
| 1 | IP'-2 | 1.9600692 | 20.88134 | 23.542982 | 45.18401 | 68.164589 | 92.254761 | 106.80562 | 139.55663 | 172.92149 |
| 1 | IP'-3 | 1.9095066 | 20.786421 | 23.505384 | 44.900089 | 67.265396 | 89.993919 | 102.11021 | 132.57883 | 162.759 |
| 1 | IP'-4 | 1.8959305 | 20.632423 | 23.428394 | 44.832432 | 67.501732 | 91.025993 | 104.6868 | 136.68404 | 169.61499 |
| 1 | IP'-5 | 1.9139638 | 20.410635 | 23.271124 | 44.426662 | 66.780411 | 89.746864 | 102.60289 | 133.69673 | 165.69913 |
| 1 | IP'-6 | 1.9408216 | 20.278488 | 23.180384 | 44.299362 | 66.887238 | 90.62912 | 105.01711 | 137.66611 | 172.41908 |
| 1 | IP'-7 | 2.0275943 | 20.087648 | 23.00436 | 43.791885 | 65.867714 | 88.734795 | 101.74551 | 133.00085 | 166.04861 |
| 1 | IP'-8 | 2.1585343 | 19.991837 | 23.000021 | 43.671494 | 65.749046 | 88.672562 | 101.8519 | 133.32234 | 167.15134 |
| 1 | IP'-9 | 2.2561128 | 19.873575 | 23.048353 | 43.484062 | 65.403481 | 87.954628 | 100.5336 | 131.70287 | 165.67838 |
| 1 | IP'-10 | 2.7085056 | 19.4331 | 23.2742 | 43.109077 | 64.786545 | 86.112244 | 95.407265 | 124.51768 | 158.5296 |
| 2 | OF'-1 | 2.2415149 | 2.3206949 | 2.5371859 | 5.1900458 | 18.116827 | 75.277069 | 271.61487 | 465.52536 | 717.01501 |
| 2 | OF'-2 | 2.0091977 | 2.1507895 | 2.3799806 | 3.8191922 | 18.011314 | 74.319519 | 257.39029 | 406.9697 | 618.4798 |
| 2 | OF'-3 | 1.7009771 | 1.8312886 | 1.9226377 | 4.5174813 | 17.458828 | 88.263939 | 259.52963 | 462.9451 | 810.80286 |
| 2 | OF'-4 | 1.731831 | 1.8226223 | 2.0067406 | 3.6915448 | 16.754211 | 55.948544 | 85.635101 | 149.01369 | 233.12622 |
| 2 | OF'-5 | 2.1045251 | 2.087676 | 2.1197662 | 4.2204747 | 15.466234 | 37.860001 | 152.55836 | 90.680397 | 192.53928 |
| 2 | OF'-6 | 2.1867504 | 2.1650133 | 2.5499563 | 8.0419807 | 26.818504 | 73.184296 | 277.68176 | 664.36353 | 682.11627 |
| 2 | OF'-7 | 1.9437269 | 1.9964747 | 2.1217976 | 5.9548368 | 19.999622 | 44.478138 | 281.7262 | 475.8826 | 427.12497 |
| 2 | OF'-8 | 1.4476978 | 1.448203 | 1.6239319 | 4.8525953 | 15.031003 | 14.514158 | 252.89513 | 142.1293 | 58.080139 |
| 2 | OF'-9 | 3.5704417 | 3.4498494 | 35.204254 | 122.99228 | 366.75214 | 784.5105 | 984.90747 | 1047.5852 | 1046.5728 |
| 2 | OF'-10 | 3.515028 | 3.3895755 | 34.56889 | 121.63602 | 348.36221 | 487.50659 | 491.69467 | 498.60666 | 493.02075 |
| 2 | OF'-11 | 1.4525905 | 1.4822139 | 2.6313124 | 5.9707789 | 6.2178826 | 25.165426 | 368.31403 | 383.06061 | 1245.6548 |
| 2 | OF'-12 | 2.4264877 | 2.3567708 | 3.2897332 | 7.4017701 | 4.7597232 | 69.822197 | 598.75867 | 789.6781 | 1287.7896 |
| 2 | B'-3 | 2.6652036 | 3.2172205 | 16.136541 | 16.918409 | 73.396057 | 385.63077 | 1204.5387 | 2488.9172 | 3335.252 |
| 2 | B'-4 | 43.473198 | 99.810715 | 56.422443 | 94.658134 | 165.87538 | 154.38478 | 554.09344 | 1334.9412 | 2495.7427 |
| 2 | B'-5 | 2.5676327 | 2.8694177 | 13.833731 | 12.684999 | 49.064735 | 276.84399 | 725.80969 | 1646.1648 | 2900.1956 |
| 2 | B'-6 | 2.8709681 | 3.0076127 | 14.024 | 14.097053 | 34.484982 | 229.19962 | 670.67126 | 1835.8705 | 2950.3088 |
| 2 | B'-7 | 2.841491 | 3.1194942 | 13.478901 | 13.320038 | 30.931026 | 170.82526 | 506.44702 | 1059.9114 | 2073.459 |
| 2 | B'-8 | 2.4824879 | 2.8819637 | 12.578749 | 11.932656 | 34.568443 | 207.98186 | 553.33405 | 1202.1141 | 2409.5605 |
| 2 | TA'-4 | 2.9103022 | 3.967278 | 21.988482 | 18.999275 | 146.21458 | 568.35297 | 1697.6416 | 3073.6296 | 4537.3872 |
| 2 | TA'-5 | 2.8503497 | 3.9103467 | 23.057671 | 19.453625 | 150.58051 | 821.26843 | 2467.5479 | 3755.4302 | 5164.0601 |
| 2 | TA'-6 | 2.72542 | 3.8349731 | 23.673328 | 20.119778 | 147.3452 | 896.4967 | 2456.8884 | 3709.2913 | 5120.1631 |
| 2 | TA'-7 | 2.708524 | 3.7872138 | 24.034941 | 20.635756 | 236.90823 | 1443.0901 | 2742.6011 | 4015.6428 | 5483.1821 |
| 2 | TA'-8 | 2.6476655 | 3.7472417 | 23.684374 | 20.383442 | 156.60576 | 932.04126 | 2402.53 | 3667.3445 | 5116.562 |
| 2 | TA'-9 | 2.9307775 | 3.9852538 | 23.821598 | 19.91993 | 134.92081 | 700.41144 | 2198.7341 | 3528.5867 | 4968.96 |
| 2 | TA'-10 | 2.8353565 | 3.8995926 | 24.732271 | 20.548639 | 187.17451 | 1244.941 | 2566.3582 | 3876.9153 | 5372.5845 |
| 2 | TA'-11 | 2.6117027 | 3.7690248 | 22.882982 | 21.654999 | 244.32448 | 1347.7991 | 2785.863 | 4112.0015 | 5650.5356 |
| 2 | TA'-12 | 2.5978878 | 3.8598762 | 24.731878 | 22.128138 | 246.66049 | 1474.234 | 2841.2993 | 4216.7993 | 5798.5635 |
| 2 | TH1'-1 | 3.7443721 | 4.9166012 | 24.721918 | 21.542349 | 230.55823 | 1661.0925 | 3322.614 | 4865.4395 | 6440.5156 |
| 2 | TH1'-2 | 3.0906842 | 4.329824 | 22.050186 | 25.772326 | 275.83813 | 1551.4506 | 3301.7786 | 4839.4922 | 6392.1045 |
| 2 | TH1'-3 | 3.0071526 | 4.1968632 | 21.906372 | 28.535913 | 316.52859 | 1774.3568 | 3469.5413 | 5007.2002 | 6570.8491 |
| 2 | TH1'-4 | 2.8142498 | 4.0973201 | 23.181221 | 22.772394 | 225.07005 | 1368.0903 | 3114.2739 | 4630.9395 | 6209.46 |
| 2 | TH1'-5 | 3.292515 | 4.4334593 | 26.589966 | 21.915138 | 202.7744 | 1434.2635 | 3130.0737 | 4648.0049 | 6225.1704 |
| 2 | TH1'-6 | 2.7789724 | 4.0623803 | 23.976067 | 23.006418 | 277.97946 | 1734.2269 | 3377.884 | 4891.0864 | 6470.9976 |
| 2 | TH1'-7 | 2.7136388 | 3.9692287 | 23.010641 | 24.411646 | 238.90067 | 1219.1433 | 2994.0925 | 4486.9189 | 6066.7373 |
| 2 | TH1'-8 | 2.6844811 | 3.9782095 | 22.647625 | 27.763479 | 383.67923 | 1884.4501 | 3525.7258 | 5038.082 | 6639.6084 |
| 2 | TH1'-9 | 3.0263844 | 4.1546683 | 24.927475 | 22.991079 | 261.95483 | 1562.4093 | 3226.5725 | 4727.708 | 6324.1382 |
| 2 | TH1'-10 | 2.8489118 | 4.0845256 | 26.47296 | 22.41267 | 390.71481 | 1854.7318 | 3481.093 | 5003.9673 | 6637.439 |
| 2 | TH1'-11 | 2.8387914 | 4.1277809 | 26.711267 | 22.890158 | 308.45972 | 1709.9138 | 3307.8796 | 4799.877 | 6452.8823 |
| 2 | TH1'-12 | 2.8348773 | 4.2338586 | 26.251909 | 25.948828 | 271.75931 | 1652.4275 | 3304.4006 | 4823.4834 | 6524.2217 |
| 2 | TH'-5 | 2.9565065 | 4.2538228 | 25.887327 | 23.140863 | 440.95163 | 2031.9547 | 3760.3176 | 5355.9976 | 7007.8281 |
| 2 | TH'-6 | 2.4995131 | 3.8659306 | 20.469879 | 35.302536 | 308.84885 | 1725.6519 | 3463.0962 | 5011.0942 | 6615.8979 |
| 2 | TH'-7 | 2.7168889 | 4.0570436 | 24.220814 | 24.534063 | 314.68478 | 1843.5328 | 3518.6636 | 5058.9995 | 6706.4399 |
| 2 | TH'-8 | 2.6278822 | 3.9818261 | 21.678368 | 30.675552 | 264.96552 | 1189.2422 | 3013.0598 | 4579.6138 | 6202.2842 |
| 2 | TH'-9 | 3.2447643 | 4.4902539 | 28.933519 | 23.925907 | 270.59839 | 1678.3905 | 3303.1113 | 4825.8574 | 6475.9604 |
| 2 | TH'-10 | 2.9367249 | 4.2066774 | 25.844793 | 25.048145 | 414.08847 | 1970.0203 | 3627.9504 | 5167.5493 | 6825.0732 |
| 2 | TH'-11 | 2.9833424 | 4.2203021 | 26.699406 | 27.596237 | 684.2348 | 2125.0146 | 3845.2285 | 5419.9937 | 7095.0127 |
| 2 | TH'-12 | 2.9841723 | 4.217536 | 25.74905 | 25.939789 | 346.11401 | 1859.9266 | 3514.7322 | 5053.6104 | 6770.1919 |
| 2 | P'-1 | 4.3771753 | 5.6088138 | 24.051302 | 39.544552 | 495.3237 | 2219.6499 | 4150.7974 | 5849.144 | 7563.9434 |
| 2 | P'-2 | 3.3627868 | 4.8602266 | 24.759583 | 43.098827 | 675.48999 | 2453.2581 | 4396.0972 | 6124.1621 | 7852.0669 |
| 2 | P'-3 | 3.071008 | 4.5033851 | 24.334969 | 39.36113 | 502.08008 | 2225.0515 | 4150.7793 | 5853.9365 | 7585.8169 |
| 2 | P'-4 | 2.5922697 | 4.2010622 | 21.786434 | 57.641109 | 734.8725 | 2580.209 | 4510.7197 | 6215.7734 | 7947.4326 |
| 2 | P'-5 | 3.0890977 | 4.626483 | 25.477852 | 45.680519 | 718.40143 | 2498.2976 | 4427.7476 | 6146.0342 | 7904.0938 |
| 2 | P'-6 | 2.8730965 | 4.4784269 | 26.116234 | 35.845284 | 435.75177 | 2126.4763 | 4030.0122 | 5730.7744 | 7502.9312 |
| 2 | P'-7 | 2.7247996 | 4.2948918 | 23.570686 | 50.783485 | 689.50195 | 2498.8271 | 4399.5327 | 6098.6455 | 7862.6724 |
| 2 | P'-8 | 2.7105918 | 4.3056483 | 24.843769 | 43.754578 | 730.07544 | 2431.1155 | 4334.7969 | 6045.7861 | 7829.606 |
| 2 | P'-9 | 2.8193395 | 4.3649106 | 23.411652 | 48.978935 | 473.25558 | 2115.313 | 4046.8254 | 5719.8687 | 7487.8271 |
| 2 | P'-10 | 2.8722856 | 4.4469256 | 27.130388 | 58.409149 | 939.73706 | 2587.6707 | 4520.4146 | 6232.6211 | 8005.2759 |
| 2 | P'-11 | 2.8699996 | 4.3892517 | 23.175259 | 50.204151 | 453.40045 | 2138.7583 | 4047.9182 | 5729.689 | 7524.3667 |
| 2 | P'-12 | 2.8183379 | 4.4207559 | 25.174376 | 54.623833 | 866.02783 | 2558.2927 | 4485.1563 | 6203.9932 | 8013.1831 |
| 2 | OH'-1 | 3.7721732 | 4.7460523 | 24.410734 | 21.775167 | 255.35852 | 1628.3026 | 3374.8899 | 4910.0488 | 6460.6025 |
| 2 | OH'-2 | 3.0724237 | 4.3647723 | 22.163685 | 30.780592 | 379.91312 | 1992.8623 | 3723.1643 | 5301.5068 | 6886.8799 |
| 2 | OH'-3 | 3.3931024 | 4.525589 | 22.964508 | 28.785656 | 283.00995 | 1325.3835 | 3253.8137 | 4874.7393 | 6488.0718 |
| 2 | OH'-4 | 2.9383101 | 4.2994499 | 21.30397 | 36.881706 | 299.97806 | 1344.3541 | 3294.4844 | 4942.1396 | 6578.1802 |
| 2 | OH'-5 | 3.2662745 | 4.6127343 | 22.591852 | 53.773289 | 781.33673 | 2517.2183 | 4433.5859 | 6128.688 | 7805.0786 |
| 2 | OH'-6 | 2.932056 | 4.4775681 | 21.670033 | 68.277901 | 940.58276 | 2685.0967 | 4667.8799 | 6404.252 | 8115.0283 |
| 2 | OH'-7 | 3.0862322 | 4.6820798 | 24.234425 | 85.197502 | 1177.9297 | 2935.6672 | 4983.5557 | 6749.6943 | 8486.7012 |
| 2 | OH'-8 | 2.9072556 | 4.5938597 | 23.576488 | 57.854244 | 737.4563 | 2640.5022 | 4683.6899 | 6486.6675 | 8302.8477 |
| 2 | OH'-9 | 3.3093429 | 4.9912486 | 27.393482 | 52.565796 | 750.54175 | 2652.8984 | 4715.647 | 6538.1865 | 8384.541 |
| 2 | OH'-10 | 2.8607352 | 4.735229 | 23.893028 | 64.179878 | 674.14667 | 2587.6892 | 4697.7852 | 6530.8735 | 8351.7295 |
| 2 | OH'-11 | 2.7946444 | 4.7243862 | 21.49399 | 84.089134 | 703.68756 | 2560.7534 | 4739.4111 | 6605.0752 | 8494.6084 |
| 2 | OH'-12 | 2.5341761 | 4.6330109 | 20.429417 | 99.865555 | 871.35406 | 2880.6392 | 5096.2383 | 6994.7402 | 8923.9141 |
| 3 | OF-1 | 1.1734952 | 4.6648536 | 13.932146 | 31.480095 | 52.751675 | 78.093323 | 98.178345 | 112.62722 | 127.0925 |
| 3 | OF-2 | 1.250037 | 4.7137351 | 13.739168 | 30.993225 | 52.016155 | 77.328369 | 97.642677 | 112.49799 | 127.33118 |
| 3 | OF-3 | 1.2954434 | 4.3964748 | 13.457008 | 30.489502 | 51.214581 | 76.158089 | 96.177223 | 110.89733 | 125.57952 |
| 3 | OF-4 | 1.201515 | 4.2956262 | 13.125363 | 29.765272 | 49.960003 | 74.119453 | 93.511871 | 107.78983 | 121.86441 |
| 3 | OF-5 | 0.9600594 | 4.1843324 | 12.949085 | 29.357599 | 49.408859 | 73.503761 | 93.101921 | 107.62995 | 121.79644 |
| 3 | OF-6 | 1.4087707 | 4.1381755 | 12.660025 | 28.676495 | 48.480339 | 72.531837 | 92.519745 | 107.55706 | 122.33591 |
| 3 | OF-7 | 1.5149899 | 4.1969357 | 12.441864 | 28.132946 | 47.40799 | 70.718254 | 89.913872 | 104.20158 | 118.08659 |
| 3 | OF-8 | 1.2288246 | 4.0499535 | 12.257729 | 27.710594 | 46.658188 | 69.610481 | 88.532829 | 102.62116 | 116.24156 |
| 3 | OF-9 | 1.7276933 | 4.1773634 | 12.192771 | 27.36001 | 46.09943 | 68.909454 | 87.925102 | 102.33516 | 116.37238 |
| 3 | OF-10 | 1.73069 | 3.9587789 | 11.900537 | 26.795536 | 45.25626 | 67.706039 | 86.513214 | 100.77422 | 114.75597 |
| 3 | OF-11 | 0.7640574 | 3.7340677 | 11.689801 | 26.545158 | 44.872768 | 67.09948 | 85.759804 | 99.87413 | 113.58212 |
| 3 | OF-12 | 1.076269 | 3.7323999 | 11.734307 | 26.604532 | 45.085011 | 67.588989 | 86.683678 | 101.19806 | 115.45954 |
| 3 | OF-13 | 1.3093289 | 3.7440453 | 11.665698 | 26.451845 | 44.778591 | 67.023743 | 85.820175 | 100.12278 | 114.09134 |
| 3 | OF-14 | 1.158432 | 3.7164516 | 11.65101 | 26.459698 | 44.738098 | 66.708809 | 85.108932 | 99.074287 | 112.43884 |
| 3 | OF-15 | 1.3256598 | 3.7286119 | 11.836677 | 26.913902 | 45.448257 | 67.54464 | 86.039192 | 99.986641 | 112.97803 |
| 3 | OF-16 | 2.4340315 | 4.5359716 | 14.484778 | 32.605778 | 54.021332 | 78.087433 | 98.31115 | 115.78126 | 137.60883 |
| 3 | A-1 | 1.4754965 | 4.372889 | 13.565335 | 30.787806 | 51.64436 | 76.428139 | 95.78627 | 110.23803 | 124.31157 |
| 3 | A-2 | 1.1129544 | 4.3590822 | 13.696907 | 31.118752 | 52.31543 | 77.661469 | 97.714699 | 112.81879 | 127.50608 |
| 3 | A-3 | 0.8875054 | 4.3541398 | 13.783792 | 31.340391 | 52.773407 | 78.48877 | 98.968338 | 114.44391 | 129.48988 |
| 3 | A-4 | 0.9072244 | 4.29252 | 13.596744 | 30.870327 | 51.893131 | 76.957695 | 96.780022 | 111.73276 | 126.21274 |
| 3 | A-5 | 0.8294521 | 4.2292719 | 13.432417 | 30.538742 | 51.413239 | 76.396744 | 96.302406 | 111.40754 | 125.86609 |
| 3 | A-6 | 0.8444795 | 4.1932025 | 13.337567 | 30.367819 | 51.226978 | 76.328812 | 96.565239 | 111.99333 | 126.89019 |
| 3 | A-7 | 0.82804 | 4.1689382 | 13.269758 | 30.208427 | 50.8978 | 75.671463 | 95.494987 | 110.5505 | 124.93214 |
| 3 | A-8 | 0.8995833 | 4.1606216 | 13.278684 | 30.247311 | 51.033802 | 76.008072 | 96.172348 | 111.5401 | 126.22439 |
| 3 | A-9 | 0.9373715 | 4.1749058 | 13.247839 | 30.13843 | 50.771942 | 75.402878 | 95.156624 | 110.17839 | 124.5006 |
| 3 | A-10 | 1.1830896 | 4.2535725 | 13.129535 | 29.900074 | 50.370888 | 74.77047 | 94.429665 | 109.24657 | 123.50667 |
| 3 | A-11 | 1.7360564 | 4.3484206 | 13.189645 | 29.920128 | 50.399952 | 74.698517 | 94.237526 | 109.05006 | 123.13477 |
| 3 | A-12 | 0.9937224 | 4.1983457 | 13.163584 | 29.920292 | 50.401424 | 74.774712 | 94.341255 | 109.16624 | 123.40589 |
| 3 | B-3 | 1.3399584 | 2.987782 | 7.9210038 | 17.73436 | 30.221189 | 45.879917 | 59.550877 | 70.51104 | 81.799919 |
| 3 | B-4 | 1.082577 | 2.8968511 | 8.1428585 | 18.357428 | 31.310564 | 47.485546 | 61.622032 | 72.895851 | 84.471825 |
| 3 | B-5 | 1.3842407 | 3.0465345 | 8.3813982 | 18.891617 | 32.186176 | 48.700504 | 63.178818 | 74.473808 | 86.043907 |
| 3 | B-6 | 1.6716667 | 2.8981886 | 8.5553284 | 19.398464 | 33.037739 | 49.974018 | 64.711334 | 76.274536 | 87.96711 |
| 3 | B-7 | 1.4091655 | 2.8464434 | 8.7441864 | 19.899338 | 33.843544 | 50.958363 | 65.66217 | 77.076004 | 88.603218 |
| 3 | B-8 | 1.0388763 | 2.9118001 | 9.0933666 | 20.744587 | 35.23328 | 52.838268 | 67.845261 | 79.402412 | 90.7388 |
| 3 | TA-5 | 0.7725428 | 1.1499677 | 2.8631928 | 6.3297443 | 10.808493 | 16.484852 | 21.677809 | 25.84186 | 30.564026 |
| 3 | TA-6 | 0.6674718 | 1.0833589 | 2.849335 | 6.392066 | 10.934425 | 16.69705 | 22.049236 | 26.251163 | 31.148289 |
| 3 | TA-7 | 0.5995627 | 1.0538077 | 2.8854749 | 6.5084581 | 11.153886 | 17.055536 | 22.627907 | 26.902851 | 31.989304 |
| 3 | TA-8 | 0.5628227 | 1.0666531 | 2.8979797 | 6.5276222 | 11.189538 | 17.09547 | 22.761765 | 27.035851 | 32.213936 |
| 3 | TA-9 | 0.6038774 | 1.1098276 | 2.8924296 | 6.4635692 | 11.078037 | 16.913511 | 22.615238 | 26.832495 | 32.031887 |
| 3 | TA-10 | 1.4572645 | 1.3258935 | 3.0062327 | 6.5264249 | 11.139533 | 17.015341 | 22.775122 | 27.046322 | 32.359917 |
| 3 | TA-11 | 1.4820997 | 1.5782357 | 3.0749567 | 6.4468551 | 10.932334 | 16.664705 | 22.352425 | 26.480991 | 31.776014 |
| 3 | TA-12 | 1.4965994 | 1.499274 | 2.9393981 | 6.2028174 | 10.541234 | 16.110718 | 21.71587 | 25.652498 | 30.869179 |
| 3 | TH-6  (Ground) | 1.6228536 | 18.73217 | 44.4356 | 98.104065 | 167.64021 | 254.63522 | 333.77069 | 397.87366 | 418.56967 |
| 3 | TH-7  (Common) | 1.8986354 | 18.354246 | 46.697174 | 103.77512 | 178.08652 | 271.71198 | 357.08994 | 426.78595 | 482.35687 |
| 3 | TH-8 | 1.0141789 | 1.1317148 | 1.1685328 | 1.2494833 | 1.4775144 | 1.8616883 | 2.3688836 | 2.7361791 | 3.2631347 |
| 3 | TH-9 | 1.2672404 | 1.5290681 | 1.4886628 | 1.6134987 | 1.9144105 | 2.3964958 | 3.1203387 | 3.5790606 | 4.191637 |
| 3 | TH-10 | 0.6735102 | 0.807091 | 0.9690858 | 1.4569132 | 2.2790072 | 3.3895845 | 4.6823816 | 5.5261106 | 6.6326137 |
| 3 | TH-11 | 0.6769325 | 0.8689884 | 1.0922481 | 1.693893 | 2.7229202 | 4.0917921 | 5.7198792 | 6.724607 | 8.0768585 |
| 3 | TH-12 | 0.9023928 | 0.9983124 | 1.2094926 | 1.805702 | 2.8433933 | 4.2374573 | 5.9722581 | 7.000453 | 8.4040689 |
| 3 | P-1 | 0.9594525 | 0.8876577 | 0.9901699 | 1.182565 | 1.4854681 | 1.8592911 | 2.6384721 | 2.5915244 | 3.5881925 |
| 3 | P-2 | 0.9027662 | 0.8420338 | 0.8996747 | 0.9375975 | 0.9956647 | 1.0918124 | 1.1806116 | 1.1741999 | 1.0958085 |
| 3 | P-3 | 0.8713056 | 0.8870142 | 0.9489599 | 0.9816849 | 1.0468445 | 1.1825459 | 1.2591172 | 1.3765424 | 1.1646062 |
| 3 | P-4 | 0.9320072 | 1.0083566 | 1.062693 | 1.0829182 | 1.1402566 | 1.2464787 | 1.3374326 | 1.4449688 | 1.2241931 |
| 3 | P-5 | 0.9925879 | 1.0984986 | 1.1345894 | 1.1626309 | 1.2025359 | 1.2875814 | 1.3730985 | 1.5005895 | 1.2162571 |
| 3 | P-6 | 0.6208909 | 1.102949 | 1.1341776 | 1.1650946 | 1.2149522 | 1.3235096 | 1.409936 | 1.5719099 | 1.2054473 |
| 3 | P-7 | 0.712394 | 1.0955588 | 1.1217948 | 1.1475412 | 1.1885502 | 1.2674906 | 1.3648759 | 1.5425938 | 1.0923554 |
| 3 | P-8 | 0.8538959 | 1.0336115 | 1.0742439 | 1.1282092 | 1.1611409 | 1.2244122 | 1.3049309 | 1.3328205 | 1.7490119 |
| 3 | PO-1 | 2.3567863 | 2.467551 | 3.5898843 | 6.6393676 | 11.113282 | 17.010397 | 21.447836 | 25.874882 | 30.223885 |
| 3 | PO-2 | 1.8544647 | 2.5671515 | 3.7276347 | 6.6759429 | 11.06713 | 16.989231 | 21.395607 | 25.785151 | 30.021893 |
| 3 | PO-3 | 1.4130691 | 2.1173012 | 3.3920023 | 6.4873848 | 10.888021 | 16.663033 | 21.159296 | 25.514376 | 29.672241 |
| 3 | PO-4 | 1.1823058 | 1.7890191 | 3.1609006 | 6.3199396 | 10.724384 | 16.391264 | 20.919739 | 25.230242 | 29.339357 |
| 3 | PO-5 | 1.0233699 | 1.4966004 | 2.948529 | 6.0572004 | 10.406518 | 15.923882 | 20.330835 | 24.531168 | 28.552458 |
| 3 | PO-6 | 0.9981667 | 1.4387432 | 2.8259201 | 5.8102093 | 10.010423 | 15.324489 | 19.580942 | 23.622654 | 27.513096 |
| 3 | PO-7 | 1.0948076 | 1.4451337 | 2.7955966 | 5.720778 | 9.8466282 | 15.058577 | 19.252443 | 23.229141 | 26.958366 |
| 3 | PO-8 | 1.0545135 | 1.3861799 | 2.7416444 | 5.6373649 | 9.7118111 | 14.868959 | 19.020138 | 22.985291 | 26.546171 |
| 4 | A-1 | 1.4716264 | 3.6247211 | 5.849483 | 18.030844 | 45.218506 | 70.430222 | 91.12394 | 118.54208 | 191.04198 |
| 4 | A-2 | 1.4243429 | 15.362507 | 5.7636557 | 17.845695 | 44.809456 | 69.902359 | 90.485229 | 117.5425 | 189.09534 |
| 4 | A-3 | 1.3792691 | 3.4699244 | 5.7506919 | 17.826357 | 44.741734 | 69.714745 | 89.902672 | 116.05101 | 186.1151 |
| 4 | A-4 | 1.3520316 | 2.9126167 | 5.7714224 | 17.998701 | 45.187626 | 70.474709 | 90.829254 | 116.84683 | 186.62663 |
| 4 | A-5 | 1.3469356 | 2.7979612 | 5.8081627 | 18.026127 | 45.259331 | 70.724449 | 91.327393 | 117.47414 | 186.91023 |
| 4 | A-6 | 1.3780917 | 2.7551408 | 5.7601604 | 17.98864 | 45.341331 | 71.365395 | 93.217804 | 121.0702 | 191.90384 |
| 4 | A-7 | 1.4163617 | 2.7525792 | 5.7612929 | 17.874691 | 44.944279 | 70.496445 | 91.291275 | 117.20728 | 185.34277 |
| 4 | A-8 | 1.4123638 | 2.7377813 | 5.8156953 | 18.177601 | 45.706993 | 71.841576 | 93.281334 | 119.72663 | 188.43115 |
| 4 | A-9 | 1.502429 | 2.7526586 | 5.8830185 | 18.305397 | 46.13871 | 72.865211 | 95.456169 | 123.76559 | 194.88205 |
| 4 | A-10 | 1.8339927 | 2.9250598 | 6.0224361 | 18.530598 | 46.770298 | 74.072105 | 97.490791 | 126.64814 | 198.35497 |
| 4 | TH-5 | 2.3977623 | 3.4903147 | 10.923188 | 33.136364 | 81.14003 | 122.59618 | 148.15221 | 175.34431 | 260.71115 |
| 4 | TH-6 | 1.9795284 | 3.4204118 | 10.817643 | 32.997356 | 80.737968 | 121.79857 | 146.4183 | 171.80312 | 252.96234 |
| 4 | TH-7 | 1.7795624 | 3.3396738 | 10.667792 | 32.381088 | 79.127403 | 118.95528 | 141.92435 | 165.36504 | 242.75383 |
| 4 | TH-8 | 1.772826 | 3.3269339 | 10.564162 | 32.345329 | 79.421844 | 120.60953 | 146.18929 | 172.62965 | 255.015 |
| 4 | PO1-1 | 2.5824127 | 3.1178753 | 10.30509 | 30.997145 | 75.450523 | 113.25906 | 137.40179 | 165.9086 | 254.67757 |
| 4 | PO1-2 | 2.3278179 | 3.1005421 | 10.296853 | 31.059374 | 75.446556 | 112.75694 | 135.46329 | 161.39854 | 245.09918 |
| 4 | PO1-3 | 2.4920225 | 3.0709214 | 10.270079 | 30.870382 | 75.257492 | 112.77453 | 135.77257 | 162.16422 | 246.55794 |
| 4 | PO1-4 | 2.2647562 | 2.8943863 | 10.187901 | 30.963676 | 75.486137 | 113.3951 | 137.03909 | 163.85985 | 248.72766 |
| 4 | PO1-5 | 1.9820049 | 2.795413 | 10.22675 | 30.951763 | 75.825699 | 114.67891 | 140.2645 | 169.96559 | 258.59543 |
| 4 | PO1-6 | 1.6546 | 2.579597 | 10.095325 | 30.779285 | 75.167839 | 113.00321 | 136.60814 | 163.51237 | 248.49504 |
| 4 | PO1-7 | 1.4424596 | 2.4196665 | 10.078528 | 30.689863 | 74.840355 | 112.37315 | 135.26727 | 161.01137 | 243.55518 |
| 4 | PO1-8 | 1.4051356 | 2.3735623 | 10.05394 | 30.826849 | 75.389511 | 113.82111 | 138.21796 | 165.77887 | 251.5275 |
| 4 | PO2-1 | 2.5082767 | 3.2350674 | 10.622298 | 31.527481 | 76.297379 | 112.68401 | 133.57988 | 158.58545 | 242.37955 |
| 4 | PO2-2 | 2.1525471 | 2.9549918 | 10.513335 | 31.659359 | 76.725822 | 113.85708 | 135.93086 | 162.30605 | 248.68396 |
| 4 | PO2-3 | 1.3819313 | 2.5454283 | 10.39546 | 31.572287 | 76.677315 | 114.01537 | 136.5136 | 163.51637 | 251.09532 |
| 4 | PO2-4 | 1.391712 | 2.5248146 | 10.364652 | 31.492191 | 76.216682 | 112.55534 | 132.79208 | 156.06166 | 235.60945 |
| 4 | PO2-5 | 2.0945225 | 2.9327922 | 10.453706 | 31.48037 | 76.433105 | 113.77678 | 135.99196 | 161.77881 | 245.64507 |
| 4 | PO2-6 | 2.3167312 | 3.0717182 | 10.525033 | 31.646875 | 77.077538 | 115.39971 | 138.99503 | 166.24709 | 251.62425 |
| 4 | PO2-7 | 1.6539395 | 2.641504 | 10.383109 | 31.531988 | 76.808022 | 115.01687 | 138.6306 | 165.9482 | 251.80626 |
| 4 | PO2-8 | 1.8772626 | 2.7316444 | 10.389455 | 31.425455 | 76.532204 | 114.38545 | 137.37717 | 163.52657 | 247.19257 |
| 4 | PO2-9 | 1.7243751 | 2.6352534 | 10.330785 | 31.354164 | 76.574928 | 115.05869 | 139.42574 | 167.66257 | 254.99994 |
| 4 | PO2-10 | 1.4438109 | 2.4196517 | 10.296292 | 31.53336 | 77.165245 | 116.55563 | 142.30164 | 172.12576 | 261.57141 |
| 4 | P-1 | 3.5170269 | 4.1452565 | 11.55715 | 33.987206 | 81.811226 | 120.62299 | 142.85609 | 168.97704 | 254.19313 |
| 4 | P-2 | 2.5561748 | 3.4697657 | 11.317492 | 33.987419 | 82.217148 | 121.84047 | 145.28877 | 172.80833 | 261.2428 |
| 4 | P-3 | 4.0666046 | 4.4667253 | 11.618482 | 33.742165 | 81.458961 | 120.05672 | 141.4639 | 166.44492 | 249.60889 |
| 4 | P-4 | 2.5114884 | 3.3491113 | 11.307242 | 33.915703 | 82.076324 | 121.65228 | 144.33406 | 170.44719 | 254.88455 |
| 4 | P-5 | 2.3206744 | 3.1920726 | 11.266506 | 33.777134 | 81.875084 | 121.29366 | 144.02879 | 170.15662 | 254.63873 |
| 4 | P-6 | 2.1613219 | 3.0850837 | 11.271276 | 33.805149 | 81.839256 | 121.08047 | 143.07893 | 167.93329 | 247.97516 |
| 4 | P-7 | 1.9059651 | 2.8678188 | 11.221372 | 33.739826 | 81.860481 | 121.11996 | 142.84462 | 166.84906 | 245.76549 |
| 4 | P-8 | 1.7708579 | 2.7530422 | 11.170392 | 33.876007 | 82.106445 | 121.66524 | 143.63507 | 167.84253 | 247.49097 |
| 4 | TH'-6 | 1.7714165 | 3.262794 | 10.995263 | 33.355236 | 80.088821 | 116.43991 | 137.14458 | 164.70056 | 257.83493 |
| 4 | TH'-7 | 1.6397547 | 3.2386303 | 10.975629 | 33.099876 | 79.388832 | 115.01639 | 134.86467 | 161.26128 | 251.60875 |
| 4 | TH'-8 | 1.540494 | 3.1231573 | 10.773014 | 32.800755 | 78.624664 | 114.01164 | 134.14421 | 161.6702 | 255.27797 |
| 4 | PO1'-1 | 1.8887519 | 3.0313973 | 10.850098 | 32.794323 | 78.954926 | 115.41802 | 136.45595 | 164.62306 | 258.51797 |
| 4 | PO1'-2 | 1.7193218 | 2.7805266 | 10.777746 | 32.75753 | 78.838135 | 115.13147 | 136.00694 | 164.01759 | 257.58624 |
| 4 | PO1'-3 | 6.3116121 | 8.684392 | 10.918024 | 32.793175 | 78.379265 | 113.83483 | 133.93979 | 160.99242 | 252.86378 |
| 4 | PO1'-4 | 1.4183475 | 13.603767 | 10.693117 | 32.663925 | 78.344955 | 113.76414 | 133.25081 | 159.57565 | 249.99216 |
| 4 | PO1'-5 | 1.710566 | 12.455506 | 10.729987 | 32.605892 | 78.207108 | 113.63576 | 133.36403 | 160.32353 | 251.76627 |
| 4 | PO1'-6 | 11.629619 | 12.332099 | 10.925562 | 32.82967 | 78.65033 | 114.27834 | 133.97668 | 161.28458 | 253.39793 |
| 4 | PO1'-7 | 6.3587852 | 6.4743385 | 10.922042 | 32.756565 | 78.493683 | 113.577 | 132.8475 | 158.8394 | 248.77621 |
| 4 | PO1'-8 | 1.2983705 | 2.3970006 | 11.246593 | 34.302765 | 81.808525 | 117.14721 | 136.35977 | 164.75365 | 256.45932 |
| 4 | PO2'-1 | 1.9408081 | 3.0378458 | 10.948226 | 33.03714 | 79.679237 | 117.10563 | 139.37048 | 168.37221 | 261.58188 |
| 4 | PO2'-2 | 1.9160581 | 3.0830834 | 10.940593 | 33.196957 | 80.073792 | 117.61769 | 139.81056 | 168.37408 | 260.06378 |
| 4 | PO2'-3 | 2.2261746 | 3.1647511 | 10.953082 | 32.927528 | 79.059044 | 115.32603 | 135.59082 | 162.05659 | 251.46645 |
| 4 | PO2'-4 | 2.0930376 | 3.0821221 | 10.88436 | 32.937294 | 79.067642 | 115.26949 | 135.57063 | 162.43925 | 253.75529 |
| 4 | PO2'-5 | 2.2496693 | 3.296083 | 10.99077 | 32.91061 | 78.929016 | 114.85632 | 134.82208 | 161.35507 | 252.40202 |
| 4 | PO2'-6 | 2.1896169 | 3.1567762 | 10.886009 | 32.810867 | 78.878494 | 114.91969 | 135.21332 | 162.6535 | 256.01691 |
| 4 | PO2'-7 | 2.4363291 | 3.3086526 | 10.914682 | 32.64167 | 78.062065 | 112.60532 | 130.1938 | 153.42824 | 236.74173 |
| 4 | PO2'-8 | 1.6298791 | 2.7113442 | 10.719707 | 32.735855 | 78.793747 | 114.92035 | 135.75711 | 164.07668 | 258.25876 |
| 4 | P1'-1 | 1.3355258 | 2.6749663 | 10.814552 | 32.837627 | 79.138855 | 115.87818 | 136.52211 | 162.66187 | 250.58299 |
| 4 | P1'-2 | 1.3141234 | 2.6649051 | 10.830116 | 33.04847 | 79.816887 | 117.33057 | 139.42921 | 167.81772 | 259.47223 |
| 4 | P1'-3 | 1.6694994 | 2.8685558 | 10.88667 | 32.976971 | 79.591805 | 116.47842 | 137.55269 | 164.74536 | 255.19717 |
| 4 | P1'-4 | 1.7534288 | 2.9359281 | 10.912971 | 33.042141 | 79.716499 | 116.61508 | 137.52547 | 164.60452 | 255.76483 |
| 4 | P1'-5 | 1.9197171 | 2.9366214 | 10.947315 | 33.055737 | 79.688629 | 116.32722 | 136.85406 | 163.62502 | 253.22604 |
| 4 | P1'-6 | 1.7903471 | 2.9221876 | 10.980762 | 33.600342 | 81.187256 | 119.49651 | 142.64519 | 173.12933 | 269.2821 |
| 4 | P1'-7 | 1.6972123 | 2.8544664 | 10.995242 | 33.193363 | 79.624962 | 115.4687 | 134.54689 | 159.46997 | 245.7975 |
| 4 | P1'-8 | 1.5598142 | 2.8416708 | 11.010016 | 33.468208 | 80.059708 | 116.00426 | 135.17712 | 160.65451 | 248.96983 |
| 4 | P2'-1 | 2.2607367 | 3.2811947 | 11.682021 | 35.067589 | 84.014809 | 122.30183 | 142.25612 | 165.76234 | 243.51343 |
| 4 | P2'-2 | 2.118892 | 3.2671671 | 11.587541 | 34.989384 | 83.791832 | 121.88329 | 141.646 | 165.59355 | 246.95567 |
| 4 | P2'-3 | 1.7442572 | 3.4022222 | 11.475619 | 34.707706 | 83.174469 | 120.73319 | 140.29683 | 164.51897 | 247.31593 |
| 4 | P2'-4 | 2.6744344 | 79.662506 | 11.701958 | 34.899395 | 83.834702 | 122.42124 | 143.76627 | 170.28596 | 257.26285 |
| 4 | P2'-5 | 2.7245376 | 3.9752772 | 11.740595 | 34.633347 | 82.932251 | 120.20868 | 139.64291 | 164.1517 | 247.70287 |
| 4 | P2'-6 | 2.0324099 | 3.262548 | 11.596834 | 34.780396 | 83.318039 | 121.00212 | 140.8497 | 166.00665 | 251.45654 |
| 4 | P2'-7 | 1.4864564 | 2.9177668 | 11.538564 | 34.926472 | 84.008438 | 122.69521 | 144.52124 | 171.89259 | 257.39755 |
| 4 | P2'-8 | 1.4006065 | 2.7583251 | 11.486993 | 34.970016 | 83.860741 | 121.82455 | 142.27875 | 168.20023 | 254.612 |
| 5 | A-1 | 1.2855486 | 4.9056807 | 7.0309443 | 14.231771 | 59.673588 | 348.53519 | 831.7641 | 1555.8203 | 2411.5361 |
| 5 | A-2 | 1.3301464 | 4.9731913 | 7.1489906 | 14.607268 | 65.981613 | 475.38959 | 1123.2843 | 1916.6785 | 2831.6809 |
| 5 | A-3 | 1.2020793 | 5.035522 | 6.7539067 | 17.209522 | 74.827599 | 440.61288 | 1030.1138 | 1841.2704 | 2779.6855 |
| 5 | A-4 | 1.2001085 | 5.1147923 | 6.6748242 | 19.355873 | 81.270256 | 435.82437 | 1019.3425 | 1822.4232 | 2780.6311 |
| 5 | A-5 | 1.2864982 | 5.2846565 | 6.2656837 | 24.799665 | 103.45966 | 580.45276 | 1362.6855 | 2302.5752 | 3353.8572 |
| 5 | A-6 | 1.3955089 | 5.5264645 | 6.1663613 | 31.60745 | 125.19215 | 649.05121 | 1466.4617 | 2470.9988 | 3573.3545 |
| 5 | A-7 | 1.6770861 | 5.6608291 | 6.1704068 | 36.403091 | 145.16078 | 790.12579 | 1669.7069 | 2695.4912 | 3826.2424 |
| 5 | A-8 | 1.4965599 | 5.5185089 | 6.1629333 | 34.949894 | 158.65631 | 898.18231 | 1863.2412 | 2892.6768 | 4042.7686 |
| 5 | A-9 | 1.4628108 | 5.6819272 | 6.3834181 | 42.255833 | 158.8438 | 792.51501 | 1734.4429 | 2805.49 | 3977.0078 |
| 5 | A-10 | 1.4914451 | 5.8635602 | 6.780127 | 46.20755 | 207.61682 | 1085.0741 | 2172.0488 | 3297.7954 | 4536.2764 |
| 5 | A-11 | 1.5227249 | 6.1386085 | 7.8535933 | 58.683823 | 250.23456 | 1275.3217 | 2469.8896 | 3674.8745 | 4986.7246 |
| 5 | A-12 | 1.7621706 | 6.5349984 | 9.7310076 | 72.132156 | 255.77171 | 1187.8079 | 2374.0559 | 3637.9634 | 4976.7988 |
| 5 | A-13 | 2.4788861 | 6.8443661 | 10.397616 | 80.98391 | 428.00226 | 1807.8572 | 3168.9146 | 4516.2036 | 5943.6011 |
| 5 | A-14 | 1.5998996 | 7.4173183 | 21.189314 | 146.06877 | 517.61884 | 2003.6675 | 3629.0972 | 5219.1128 | 6824.666 |
| 5 | A-15 | 1.9904495 | 20.106745 | 204.77917 | 1774.9359 | 3904.324 | 8128.7598 | 11338.486 | 13925.313 | 16367.094 |
| 5 | B-3 | 1.6723933 | 4.3359184 | 6.0520673 | 14.153502 | 52.02766 | 225.51575 | 554.32062 | 1070.5598 | 1774.6976 |
| 5 | B-4 | 1.3086873 | 4.2376685 | 5.8051257 | 16.188852 | 61.436787 | 253.35783 | 523.03381 | 1011.4022 | 1698.9512 |
| 5 | B-5 | 1.9595019 | 4.7847915 | 6.1284823 | 17.549852 | 68.467422 | 385.49448 | 929.42798 | 1669.6594 | 2572.8357 |
| 5 | B-6 | 2.0251844 | 4.6903081 | 6.1147327 | 18.009541 | 70.434738 | 373.52228 | 931.61389 | 1694.024 | 2621.0127 |
| 5 | B-7 | 2.0851972 | 4.8472176 | 6.1273308 | 19.293619 | 77.565887 | 488.54721 | 1200.906 | 2005.6892 | 2977.4915 |
| 5 | B-8 | 2.149549 | 4.9679475 | 6.0095773 | 21.365669 | 82.404121 | 429.92746 | 1054.1359 | 1893.265 | 2860.3882 |
| 5 | B-9 | 1.5911255 | 4.6417031 | 5.785059 | 24.396238 | 96.44928 | 556.37219 | 1274.7667 | 2183.9753 | 3211.0579 |
| 5 | B-10 | 1.359385 | 4.8859477 | 6.8357701 | 44.198555 | 154.56166 | 707.22949 | 1608.4502 | 2674.5061 | 3864.9841 |
| 5 | TA-5 | 0.8450591 | 1.4240594 | 1.6435454 | 1.6828799 | 1.7917202 | 1.7461334 | 1.8438791 | 2.0411694 | 2.7892561 |
| 5 | TA-6 | 0.7099369 | 1.3425915 | 1.5805187 | 1.6180842 | 1.7211652 | 1.6897715 | 1.6673677 | 1.877762 | 2.690747 |
| 5 | TA-7  (Ground) | 0.025353 | 0.0253529 | 0.0253529 | 0.0253529 | 0.0253529 | 0.0253529 | 0.025353 | 0.0253529 | 0.0253529 |
| 5 | TA-8  (Common) | 0.025353 | 0.0253529 | 0.0253529 | 0.0253529 | 0.0253529 | 0.0253529 | 0.025353 | 0.0253529 | 0.0253529 |
| 5 | TA-9 | 0.5841551 | 1.2855812 | 1.5262259 | 1.5347439 | 1.6773968 | 1.8373014 | 2.33407 | 3.1377964 | 4.5696974 |
| 5 | TA-10 | 0.7585194 | 1.3623853 | 1.6271671 | 1.6772789 | 1.8054175 | 1.8174387 | 1.6440127 | 1.8797143 | 2.6990476 |
| 5 | TA-11 | 0.9694269 | 1.4957494 | 1.785954 | 2.03671 | 2.3326201 | 2.8550491 | 2.5318334 | 2.6980844 | 3.6480987 |
| 5 | TA-12 | 1.0887278 | 1.5931518 | 1.9614677 | 2.5937302 | 3.1619778 | 4.4986811 | 4.3307672 | 4.5296111 | 5.4394054 |
| 5 | TA-13 | 1.0605177 | 1.6208715 | 2.4076953 | 4.1376939 | 5.4530864 | 8.6478548 | 8.9610624 | 9.6359062 | 11.212855 |
| 5 | TA-14 | 1.79805 | 2.8318353 | 5.4734521 | 11.579441 | 15.459808 | 23.904572 | 25.608538 | 26.751545 | 26.831835 |
| 5 | TA-15 | 1.8335751 | 2.8662701 | 7.2643213 | 15.720875 | 21.383915 | 34.281055 | 37.013523 | 39.522198 | 40.74321 |
| 5 | C-3 | 2.3837762 | 2.7882876 | 3.9209044 | 6.7797723 | 8.1427822 | 10.765712 | 11.813084 | 11.491923 | 14.25185 |
| 5 | C-4 | 1.9525547 | 2.4693599 | 3.5564125 | 6.7105675 | 8.2013569 | 10.971442 | 12.183421 | 11.720999 | 14.137849 |
| 5 | C-5 | 2.0645797 | 2.5382087 | 3.7385285 | 6.8929482 | 8.4227314 | 11.404737 | 12.795774 | 12.551805 | 13.78865 |
| 5 | C-6 | 1.547935 | 2.108417 | 3.4729097 | 6.8310251 | 8.433444 | 11.304605 | 12.578926 | 12.527659 | 16.083906 |
| 5 | C-7 | 1.6188716 | 2.1641116 | 3.5646212 | 6.9103651 | 8.2426319 | 10.330616 | 11.381194 | 14.146821 | 25.276474 |
| 5 | C-8 | 1.6711509 | 2.2506874 | 3.6248531 | 7.0425568 | 8.4310341 | 10.657248 | 11.805101 | 14.252936 | 24.950842 |
| 5 | C-9 | 1.6134175 | 2.2532668 | 3.5779083 | 6.8825951 | 8.0779724 | 10.072788 | 11.569392 | 16.582918 | 31.003897 |
| 5 | C-10 | 1.5953996 | 2.1468008 | 3.5521841 | 6.8800035 | 8.2805433 | 10.505406 | 11.930778 | 16.109234 | 28.744257 |
| 5 | P1-1 | 0.8183219 | 1.5233871 | 2.4802015 | 4.8463697 | 6.2809939 | 9.5154886 | 12.210241 | 13.618969 | 13.431437 |
| 5 | P1-2 | 0.7985998 | 1.5118881 | 2.511524 | 4.9296455 | 6.4720755 | 10.023495 | 12.46552 | 13.943594 | 13.957279 |
| 5 | P1-3 | 1.1454679 | 1.7618055 | 2.6906633 | 5.0913239 | 6.6159158 | 10.103827 | 12.171303 | 13.339955 | 13.332561 |
| 5 | P1-4 | 1.4142655 | 1.8292333 | 2.8637569 | 5.4707575 | 7.0211287 | 10.689699 | 12.564385 | 13.711755 | 13.818555 |
| 5 | P1-5 | 1.2328947 | 1.8835694 | 3.0499091 | 5.8873549 | 7.6193652 | 11.601481 | 13.385345 | 14.509006 | 14.903028 |
| 5 | P1-6 | 1.1188009 | 1.8154262 | 3.2852261 | 6.6892786 | 8.7480545 | 13.484857 | 15.237255 | 16.583553 | 17.385588 |
| 5 | P1-7 | 1.1506208 | 2.0029459 | 4.2793312 | 8.9936628 | 11.596919 | 17.433897 | 18.741018 | 21.104868 | 28.008125 |
| 5 | P1-8 | 1.597212 | 3.03352 | 7.9238167 | 17.013451 | 21.899288 | 31.023043 | 30.60556 | 28.907621 | 22.207581 |
| 5 | P2-1 | 1.4323075 | 2.0961554 | 3.1999695 | 6.3760009 | 8.2756329 | 12.653731 | 15.810064 | 17.407864 | 17.128502 |
| 5 | P2-2 | 1.3167781 | 1.9949814 | 3.2757812 | 6.7457347 | 8.779583 | 13.395641 | 16.344122 | 17.809584 | 17.62356 |
| 5 | P2-3 | 1.3197138 | 2.0106597 | 3.5220151 | 7.4248247 | 9.6254969 | 14.562163 | 17.140537 | 18.380184 | 18.651144 |
| 5 | P2-4 | 1.4029907 | 2.4518816 | 5.4633818 | 11.972379 | 15.278464 | 21.972151 | 23.224352 | 22.759916 | 22.95113 |
| 5 | P2-5 | 1.4054129 | 2.4598382 | 5.446085 | 11.847964 | 14.985491 | 21.171329 | 21.936539 | 21.382133 | 22.808208 |
| 5 | FF-1 | 1.5550326 | 2.384712 | 4.5298262 | 10.01424 | 12.607977 | 18.188084 | 20.97403 | 21.048296 | 22.578768 |
| 5 | FF-2 | 1.7113949 | 2.6124005 | 4.8697491 | 10.440742 | 12.809834 | 17.7812 | 19.390244 | 21.572292 | 28.60568 |
| 5 | FF-3 | 2.1881387 | 2.9048421 | 5.2781296 | 10.848272 | 12.830613 | 16.925478 | 18.512291 | 27.008007 | 42.731434 |
| 5 | FF-4 | 3.9172468 | 3.7878389 | 6.2308416 | 11.682566 | 13.349966 | 16.73185 | 20.238844 | 36.933819 | 59.946686 |
| 5 | FF-5 | 2.1509302 | 3.0454783 | 5.7662063 | 11.577085 | 13.050303 | 17.60771 | 27.457918 | 56.946156 | 90.04258 |
| 6 | OF-1 | 1.0153563 | 1.3497958 | 7.7043333 | 16.405062 | 67.913361 | 329.1181 | 1049.9673 | 1971.2356 | 1720.7458 |
| 6 | OF-2 | 1.2508492 | 1.537784 | 7.8959403 | 18.314766 | 93.135941 | 496.14438 | 1678.5454 | 2472.6367 | 2101.0662 |
| 6 | OF-3 | 0.9787878 | 1.2808623 | 7.7856493 | 22.023382 | 100.79463 | 445.66364 | 1570.7682 | 2417.0015 | 2063.3911 |
| 6 | OF-4 | 0.9008154 | 1.1579314 | 7.7772622 | 27.257929 | 116.49934 | 408.26245 | 1399.7694 | 2329.9595 | 2054.9111 |
| 6 | OF-5 | 1.0428618 | 1.3714507 | 8.5406418 | 26.173216 | 156.09593 | 659.96613 | 2065.147 | 2996.5059 | 2479.4419 |
| 6 | OF-6 | 1.7625074 | 2.0198464 | 8.6361284 | 31.535641 | 167.67168 | 685.9054 | 2086.3088 | 3159.1201 | 2605.7136 |
| 6 | OF-7 | 2.2318408 | 2.4628608 | 9.0268583 | 33.505676 | 182.89357 | 767.84161 | 2314.8667 | 3329.3518 | 2719.9421 |
| 6 | OF-8 | 2.1111236 | 2.332094 | 9.2363205 | 33.108822 | 147.74008 | 596.17993 | 1570.5157 | 2841.2429 | 2473.8447 |
| 6 | OF-9 | 2.2685318 | 2.1852355 | 8.9358034 | 43.836281 | 188.75455 | 676.82074 | 2033.1357 | 3135.6243 | 2637.9492 |
| 6 | OF-10 | 1.9330717 | 1.9925632 | 9.1451302 | 46.882416 | 209.10738 | 830.54437 | 2330.1448 | 3414.533 | 2837.292 |
| 6 | OF-11 | 1.4273576 | 1.6262232 | 9.6063633 | 54.877899 | 248.10762 | 929.1795 | 2432.2002 | 3576.8577 | 2944.9553 |
| 6 | OF-12 | 1.5964612 | 1.7924933 | 10.24338 | 65.997993 | 309.94943 | 1256.1206 | 2919.3555 | 4125.6152 | 3327.2688 |
| 6 | B-6 | 1.7079458 | 1.9378583 | 7.0617967 | 14.610906 | 56.367455 | 211.79654 | 611.94116 | 1210.3878 | 1423.5908 |
| 6 | B-7 | 1.9751714 | 2.1517849 | 7.7812495 | 14.978297 | 75.48819 | 392.72269 | 1432.7319 | 2166.5012 | 1916.9755 |
| 6 | B-8 | 1.7708246 | 2.0682852 | 7.5243025 | 17.712515 | 78.394775 | 351.62863 | 1387.7346 | 2240.209 | 1983.7518 |
| 6 | B-9 | 1.5333104 | 1.7392364 | 7.6283751 | 19.042421 | 80.566063 | 338.80911 | 1134.115 | 2225.6055 | 1976.3018 |
| 6 | B-10 | 1.6808403 | 1.8780164 | 8.240097 | 18.788279 | 79.900253 | 355.11734 | 1419.1255 | 2372.7996 | 2074.7048 |
| 6 | TA-4 | 0.5932155 | 0.6613928 | 0.6929557 | 1.3372751 | 3.8438864 | 10.311192 | 20.742947 | 30.091131 | 35.716858 |
| 6 | TA-5  (Ground) | 1.1548847 | 1.2316419 | 1.3045104 | 1.4398264 | 2.3665316 | 4.5369287 | 8.3403835 | 11.730711 | 15.12394 |
| 6 | TA-6 (Common) | 1.1598589 | 1.232478 | 1.3060921 | 1.4464633 | 2.3934219 | 4.6495156 | 8.5763817 | 12.086222 | 15.732835 |
| 6 | TA-7 | 0.2147928 | 0.4419001 | 1.2459431 | 2.8323433 | 5.012886 | 7.8267455 | 11.001001 | 14.136572 | 14.68681 |
| 6 | TA-8 | 0.3698147 | 0.5745587 | 1.6253269 | 3.6774154 | 6.6345096 | 11.200363 | 16.581932 | 21.262175 | 21.081196 |
| 6 | TA-9 | 0.5844516 | 0.7477579 | 1.6455534 | 3.4853382 | 6.1644444 | 10.550931 | 16.649618 | 22.473841 | 24.925131 |
| 6 | TA-10 | 0.7859735 | 0.916765 | 1.5398979 | 3.048193 | 5.1927419 | 8.6124058 | 12.263888 | 14.709143 | 7.3984275 |
| 6 | TA-11 | 0.7884251 | 0.9936132 | 1.6208222 | 3.3617096 | 5.9900436 | 10.099309 | 14.822284 | 19.276373 | 12.192394 |
| 6 | TA-12 | 0.9216687 | 1.0398834 | 1.6561002 | 3.2732463 | 5.6363811 | 9.3544998 | 13.408854 | 16.789543 | 10.018744 |
| 6 | TH-5 | 1.2229429 | 1.3916061 | 2.9670689 | 4.5585399 | 6.0899949 | 12.655235 | 26.830751 | 41.168484 | 49.289703 |
| 6 | TH-6 | 1.0029937 | 1.0879695 | 2.5267441 | 3.8654835 | 5.7248697 | 12.91461 | 26.631063 | 40.242653 | 46.235973 |
| 6 | TH-7 | 1.3926954 | 1.3715612 | 2.5576475 | 3.6103661 | 5.8105359 | 14.178926 | 29.495211 | 44.415791 | 48.827114 |
| 6 | TH-8 | 1.4333688 | 1.4036597 | 2.4016211 | 3.4298313 | 5.7219005 | 13.48883 | 27.343433 | 40.988457 | 44.953758 |
| 6 | TH-9 | 0.8041663 | 0.9316571 | 1.8509275 | 2.6983376 | 5.8368354 | 15.830491 | 32.217842 | 48.319973 | 54.201 |
| 6 | TH-10 | 1.0689255 | 1.2740639 | 1.7693858 | 2.3599904 | 4.9343367 | 12.56263 | 25.043425 | 37.626945 | 41.77187 |
| 6 | TH-11 | 1.8311945 | 2.0005896 | 2.3874249 | 2.8233619 | 5.8645225 | 14.576198 | 28.626081 | 42.88821 | 48.204502 |
| 6 | TH-12 | 1.8375281 | 1.8647896 | 2.3538299 | 3.051913 | 7.122355 | 18.935066 | 37.613964 | 55.930782 | 58.74567 |
| 6 | PO-1 | 3.8800638 | 3.9680552 | 3.8475916 | 4.6439476 | 5.6822891 | 7.4291019 | 8.9357853 | 9.0034628 | 10.056497 |
| 6 | PO-2 | 3.1191149 | 3.079524 | 3.2262673 | 4.3516836 | 5.563242 | 6.9213676 | 7.8266945 | 7.4084206 | 12.840199 |
| 6 | PO-3 | 2.5829697 | 2.4477632 | 2.9588559 | 4.4290481 | 5.9565434 | 6.9386787 | 7.6724091 | 9.7231627 | 27.729738 |
| 6 | PO-4 | 1.7349584 | 1.7955312 | 2.5505204 | 4.2195292 | 5.5463657 | 6.241756 | 7.8585453 | 18.214727 | 42.6535 |
| 6 | PO-5 | 1.4591297 | 1.533013 | 2.5167766 | 4.4220142 | 6.1881514 | 7.4481168 | 8.2040777 | 11.144398 | 24.573849 |
| 6 | PO-6 (Broken) | 0.025353 | 0.0253529 | 0.0253529 | 0.0253529 | 0.0253529 | 0.0253529 | 0.0253529 | 0.0253529 | 0.0253529 |
| 6 | PO-7 | 1.5382304 | 1.5378884 | 2.633158 | 4.2470565 | 5.4838881 | 7.398942 | 13.207546 | 21.782631 | 33.145523 |
| 6 | PO-8 | 1.5771599 | 1.7833291 | 2.8789859 | 4.6197777 | 5.8369007 | 8.1101027 | 15.568596 | 27.828152 | 42.907063 |
| 6 | PO-9 | 1.2938843 | 1.372861 | 3.0461545 | 5.2037511 | 6.6420016 | 8.8428001 | 19.311037 | 38.063805 | 62.975086 |
| 6 | PO-10 | 1.1708273 | 1.299257 | 3.246222 | 5.5887022 | 6.9847493 | 9.6104717 | 23.826645 | 50.030319 | 82.283661 |
| 6 | W-1 | 0.9423174 | 1.1359463 | 1.0992118 | 1.6121886 | 3.6121972 | 8.1555986 | 14.634732 | 20.164917 | 22.501907 |
| 6 | W-2 | 1.3655421 | 1.5869435 | 1.6652093 | 2.5384209 | 4.6285405 | 9.0775127 | 15.043227 | 19.606865 | 25.296579 |
| 6 | W-3 | 1.9557955 | 2.0579028 | 2.1457365 | 2.9884508 | 4.80232 | 8.698555 | 13.888304 | 17.395008 | 16.495081 |
| 6 | W-4 | 3.1983402 | 2.5623097 | 2.8866656 | 3.9315879 | 5.7353921 | 9.2499084 | 13.412868 | 15.302382 | 12.025536 |
| 6 | W-5 | 3.6211178 | 2.8953068 | 3.2073331 | 4.4253483 | 6.4828115 | 10.686989 | 16.621552 | 21.115808 | 21.572371 |
| 6 | W-6 | 2.8692162 | 2.7867708 | 3.1070092 | 4.5519633 | 6.341826 | 9.5254574 | 13.110004 | 15.183917 | 9.2672968 |
| 6 | W-7 | 1.8435229 | 1.8787814 | 2.3463051 | 3.9987898 | 6.4463773 | 10.555809 | 15.612678 | 20.136938 | 23.652769 |
| 6 | W-8 | 1.4867104 | 1.4993784 | 1.9648957 | 3.4822605 | 5.5203929 | 8.0062571 | 10.568092 | 12.371156 | 11.437054 |
| 6 | W-9 | 1.4025027 | 1.5672536 | 1.716687 | 2.8354094 | 4.3635912 | 6.1071558 | 7.8820024 | 8.4200325 | 7.0805998 |
| 6 | W-10 | 1.3014779 | 1.6680104 | 1.7679009 | 3.2719643 | 5.4504166 | 8.4283705 | 11.703699 | 12.841298 | 5.9778843 |
| 6 | W-11 | 1.2416136 | 1.5979857 | 1.7601693 | 3.2991052 | 5.6244602 | 8.9561691 | 12.948209 | 15.533822 | 7.8918509 |
| 6 | W-12 | 1.0937157 | 1.3669844 | 1.6961348 | 3.3252196 | 5.9157186 | 10.156753 | 15.557119 | 19.759363 | 12.769492 |
| 6 | FF-1 | 1.8611428 | 2.1038334 | 7.1964788 | 12.087331 | 16.720022 | 32.575153 | 69.073952 | 108.96245 | 113.29401 |
| 6 | FF-2 | 1.8551741 | 2.0068984 | 6.8329582 | 11.473115 | 15.943201 | 31.604383 | 67.241447 | 106.14868 | 114.26048 |
| 6 | FF-3 | 0.9663962 | 1.1683838 | 6.2709494 | 10.364113 | 15.011878 | 31.624676 | 67.033684 | 105.05286 | 106.89027 |
| 6 | FF-4 | 0.9430825 | 1.125052 | 5.7705617 | 9.4076004 | 13.87273 | 30.385546 | 64.771591 | 101.41524 | 103.42125 |
| 6 | FF-5 | 2.1322293 | 2.4184556 | 6.025476 | 9.8268204 | 13.115091 | 21.259977 | 42.495495 | 67.434662 | 70.519051 |
| 6 | FF-6 | 2.1346714 | 1.9955482 | 5.6285071 | 9.1902075 | 12.0707 | 21.059345 | 43.481136 | 68.864037 | 71.330063 |
| 6 | FF-7 | 2.3719492 | 2.1339819 | 5.2119565 | 8.3985672 | 11.334041 | 20.968025 | 43.042526 | 67.401428 | 69.736565 |
| 6 | FF-8 | 2.0978706 | 1.9712368 | 4.9778509 | 8.0484314 | 10.80884 | 19.44142 | 39.513988 | 61.872253 | 64.343491 |
| 6 | FF-9 | 1.6227607 | 1.7833798 | 4.6286731 | 7.203763 | 10.916912 | 25.151903 | 52.36874 | 80.925629 | 78.795807 |
| 6 | FF-10 | 1.724593 | 1.9564772 | 4.7482772 | 7.3871017 | 10.51652 | 21.48307 | 44.499157 | 70.881264 | 74.299538 |
| 6 | P-1 | 1.3751124 | 1.7838345 | 7.1639361 | 12.353868 | 16.809561 | 29.067091 | 59.619499 | 93.706863 | 104.53198 |
| 6 | P-2 | 1.2033333 | 1.5303124 | 6.6584291 | 11.462029 | 15.637513 | 27.537823 | 56.509251 | 88.743835 | 99.026939 |
| 6 | P-3 | 1.5015929 | 1.6185106 | 6.2753367 | 10.566163 | 14.695546 | 28.035683 | 58.411175 | 91.213661 | 98.525513 |
| 6 | P-4 | 1.5535556 | 1.556309 | 5.951056 | 10.06074 | 13.744412 | 23.352165 | 46.583141 | 72.712013 | 83.097664 |
| 6 | P-5 | 0.9650764 | 1.2995038 | 5.4141827 | 9.0534258 | 12.62118 | 24.596096 | 51.155846 | 79.994766 | 88.639458 |
| 6 | P-6 | 1.2687296 | 1.3796823 | 4.7943091 | 7.6611075 | 11.926713 | 27.492643 | 57.527855 | 88.23407 | 92.228188 |
| 6 | P-7 | 1.3947241 | 1.4843957 | 4.6921864 | 7.4529047 | 10.844012 | 23.290615 | 48.541889 | 74.909813 | 80.344299 |
| 6 | P-8 | 0.9907542 | 1.1291751 | 3.961607 | 6.168138 | 10.024979 | 24.393444 | 50.866333 | 77.314178 | 80.932426 |
| 6 | P-9 | 1.625576 | 1.738435 | 3.8584895 | 6.1350503 | 8.1392984 | 14.136705 | 28.215996 | 43.945389 | 47.731941 |
| 6 | P-10 | 1.7134365 | 1.8121179 | 3.6282446 | 5.4690948 | 7.6818047 | 14.949391 | 29.991789 | 46.266994 | 49.484165 |
| 6 | P-11 | 1.9797784 | 1.9057919 | 3.5205648 | 5.2565622 | 7.3985543 | 13.76519 | 26.981728 | 41.723145 | 45.803566 |
| 6 | P-12 | 1.8940228 | 1.9729657 | 3.4290779 | 5.0592546 | 7.5293527 | 15.96664 | 32.430046 | 49.474907 | 49.730072 |
| 6 | O-1 | 2.3373818 | 2.2763677 | 8.2679377 | 14.037257 | 18.227125 | 34.424107 | 76.11927 | 121.11632 | 135.70993 |
| 6 | O-2 | 3.0974891 | 2.7380276 | 8.254117 | 13.730698 | 18.164839 | 35.872974 | 78.514236 | 125.43324 | 168.88472 |
| 6 | O-3 | 2.8396308 | 2.9525995 | 8.6092615 | 14.492144 | 18.288464 | 32.402275 | 71.430588 | 114.70451 | 125.41282 |
| 6 | O-4 | 1.9062794 | 2.357348 | 8.2157364 | 13.805286 | 17.926527 | 33.155533 | 72.644569 | 116.18277 | 127.35713 |
| 6 | O-5 | 1.7675333 | 2.023319 | 8.1768169 | 13.615577 | 17.895786 | 35.509159 | 79.175491 | 126.55439 | 131.97661 |
| 6 | O-6 | 1.2204142 | 1.5537341 | 7.9750967 | 13.190838 | 17.58972 | 36.396378 | 81.509193 | 130.2005 | 134.06404 |
| 6 | O-7 | 1.1396953 | 1.3707755 | 7.5815144 | 12.305116 | 17.288244 | 38.537697 | 85.663193 | 136.60793 | 164.68318 |
| 6 | O-8 | 1.4439281 | 1.7107089 | 7.557858 | 12.203445 | 17.150335 | 38.646923 | 86.26226 | 137.96233 | 167.20483 |
| 6 | O-9 | 1.9343038 | 2.2423513 | 7.82163 | 12.838429 | 16.869049 | 32.779999 | 73.046532 | 132.87843 | 179.66814 |
| 6 | O-10 | 1.5275564 | 1.8026556 | 7.4471502 | 12.057734 | 16.630413 | 36.836544 | 82.040947 | 131.5215 | 127.64198 |
| 7 | OF-1 | 1.3683201 | 1.7948205 | 1.9591072 | 2.7075305 | 4.4497948 | 8.206337 | 12.249219 | 14.899647 | 35.084656 |
| 7 | OF-2 | 1.1775759 | 1.6272367 | 1.8032668 | 2.3587258 | 3.6712847 | 6.8214169 | 10.210299 | 13.786499 | 38.758232 |
| 7 | OF-3 | 1.1261473 | 1.5615847 | 1.736542 | 1.9016645 | 2.5361452 | 4.2919717 | 6.0714216 | 14.468707 | 39.005184 |
| 7 | OF-4 | 1.3894191 | 2.0478153 | 2.2985399 | 1.9949014 | 1.9937147 | 2.8938301 | 3.5980341 | 4.910111 | 15.399926 |
| 7 | OF-5 | 1.4623315 | 2.3480842 | 2.5334065 | 2.3145835 | 2.1828239 | 2.5388401 | 3.5360985 | 5.4820156 | 7.1040354 |
| 7 | OF-6 (Ground) | 493.73227 | 608.28778 | 587.11511 | 453.52875 | 552.22388 | 382.10999 | 509.41617 | 411.79391 | 385.19916 |
| 7 | OF-7 (Common) | 258.08786 | 355.47916 | 378.01334 | 338.56772 | 379.13803 | 311.18881 | 371.37814 | 305.52335 | 292.81064 |
| 7 | OF-8 | 0.0279808 | 1033.8043 | 930.00458 | 828.30798 | 760.71417 | 688.83563 | 647.51733 | 620.7688 | 565.83423 |
| 7 | OF-9 | 1.9476746 | 1190.6273 | 1105.6964 | 1026.5239 | 991.14117 | 949.90393 | 942.21088 | 931.6402 | 880.49854 |
| 7 | OF-10 | 0.025353 | 922.06268 | 788.50922 | 664.34247 | 593.79327 | 536.8526 | 514.63855 | 504.38171 | 475.27405 |
| 7 | A-1 | 1.7476815 | 2.4826114 | 2.8127468 | 4.3784485 | 10.866024 | 29.700279 | 71.569206 | 147.34872 | 263.73694 |
| 7 | A-2 | 1.5863781 | 2.3682108 | 2.7171652 | 3.7817862 | 8.3462143 | 22.65435 | 68.864204 | 142.79327 | 250.43327 |
| 7 | A-3 | 1.7350391 | 2.3471329 | 2.6641533 | 3.6036937 | 7.3985543 | 18.10494 | 34.358475 | 70.216957 | 137.164 |
| 7 | A-4 | 1.3582948 | 2.0801964 | 2.2871895 | 3.0384278 | 5.6643739 | 14.389003 | 36.338219 | 86.96714 | 164.94476 |
| 7 | A-5 | 1.0332447 | 1.6837199 | 1.8103819 | 2.0418062 | 2.8687723 | 5.2314663 | 7.0930824 | 22.104715 | 59.980049 |
| 7 | A-6 | 1.0212035 | 1.6569941 | 1.7957931 | 2.0986843 | 3.1840997 | 7.1018682 | 10.520383 | 16.319401 | 29.772141 |
| 7 | A-7 | 1.0035782 | 1.6856107 | 1.7928642 | 1.9230206 | 2.4930627 | 4.2025604 | 7.4370332 | 32.286205 | 69.65229 |
| 7 | A-8 | 1.0240089 | 1.6924543 | 1.8205817 | 2.0578825 | 2.9811118 | 6.2165709 | 7.6293249 | 10.692712 | 18.121887 |
| 7 | A-9 | 1.0770626 | 1.8068304 | 1.9185944 | 1.8135651 | 1.7172513 | 2.5167165 | 7.8026223 | 11.727616 | 9.0943117 |
| 7 | A-10 | 1.1702359 | 1.911749 | 2.0887415 | 2.0880172 | 1.9201777 | 3.5644834 | 12.832664 | 22.541807 | 39.371716 |
| 7 | B-3 | 1.7115153 | 2.8360877 | 5.2739825 | 10.156917 | 11.574764 | 20.953615 | 60.286243 | 109.3026 | 215.19522 |
| 7 | B-4 | 1.4310687 | 2.6246188 | 4.7249961 | 9.0851336 | 10.424694 | 18.576777 | 53.956585 | 100.32299 | 214.58441 |
| 7 | B-5 | 1.364761 | 2.4597499 | 4.3031783 | 8.2297668 | 9.7417965 | 12.28695 | 33.231289 | 60.95435 | 119.35155 |
| 7 | B-6 | 2.1621563 | 2.7569559 | 4.1232247 | 7.1164322 | 8.4976711 | 9.7779722 | 23.913721 | 43.472317 | 93.891563 |
| 7 | B-7 | 2.0315685 | 2.7433228 | 3.9159508 | 6.3859391 | 7.4811764 | 9.0405426 | 22.703592 | 40.706772 | 80.968948 |
| 7 | B-8 | 2.3499322 | 2.8952587 | 3.8318398 | 5.8304353 | 6.567441 | 9.2210484 | 24.815819 | 43.687046 | 80.320717 |
| 7 | TA-1 | 1.6321893 | 3.4841197 | 7.4650736 | 14.310628 | 16.183359 | 43.890285 | 133.90196 | 267.4306 | 589.4939 |
| 7 | TA-2 | 1.6424519 | 3.5081601 | 6.985158 | 13.249146 | 16.016163 | 48.696873 | 139.60942 | 264.61371 | 556.76337 |
| 7 | TA-3 | 1.5646884 | 3.480525 | 6.8353963 | 13.069931 | 15.681022 | 44.692905 | 133.97496 | 302.2558 | 652.80884 |
| 7 | TA-4 | 1.4830458 | 3.5457838 | 6.7619061 | 12.694044 | 15.771899 | 47.216694 | 129.14447 | 237.29706 | 439.81778 |
| 7 | TA-5 | 1.6850467 | 4.6488776 | 7.3093038 | 13.344333 | 15.884542 | 44.874947 | 128.39848 | 245.76584 | 527.08813 |
| 7 | TA-6 | 1.584137 | 4.9750819 | 7.1572509 | 13.214664 | 16.055731 | 45.504227 | 130.72577 | 252.50627 | 549.35138 |
| 7 | TA-7 | 1.3341819 | 3.9973779 | 7.0279813 | 13.279472 | 16.134241 | 44.926064 | 150.36058 | 359.85901 | 743.52899 |
| 7 | TA-8 | 1.1795779 | 3.5047071 | 6.8151164 | 12.817674 | 17.26078 | 52.72147 | 150.10898 | 297.01535 | 647.01831 |
| 7 | TH-5 | 1.3743669 | 7.0214777 | 8.4476213 | 14.485129 | 52.242081 | 200.5731 | 714.42529 | 1245.8647 | 2070.7378 |
| 7 | TH-6 | 1.8739759 | 5.0631895 | 7.7616386 | 14.737876 | 62.391743 | 203.65881 | 568.70453 | 1082.8268 | 1881.017 |
| 7 | TH-7 | 1.2887368 | 6.6886673 | 8.087244 | 14.596814 | 53.767189 | 186.20886 | 646.49451 | 1155.2463 | 1971.9135 |
| 7 | TH-8 | 1.2966342 | 9.7176476 | 7.6309638 | 14.706592 | 61.623379 | 199.46382 | 522.64508 | 1010.5159 | 1829.5881 |
| 7 | TH-9 | 1.2857325 | 9.6646557 | 8.8322821 | 14.879434 | 43.722687 | 161.92149 | 620.10419 | 1124.6536 | 1939.0081 |
| 7 | TH-10 | 1.3763987 | 7.0238276 | 8.256465 | 14.433107 | 52.143791 | 209.8363 | 730.79541 | 1269.7239 | 2111.134 |
| 7 | IP-1 | 3.4234371 | 4.9127965 | 8.6379414 | 14.908335 | 23.850397 | 80.080986 | 234.64799 | 413.54333 | 931.3103 |
| 7 | IP-2 | 3.9624932 | 4.5147486 | 8.3223715 | 14.896773 | 22.860714 | 76.502045 | 241.88625 | 521.27319 | 1109.174 |
| 7 | IP-3 | 5.5752754 | 4.660079 | 8.1334743 | 14.448935 | 25.01697 | 84.087784 | 281.47946 | 587.30005 | 1196.7517 |
| 7 | IP-4 | 5.7947345 | 4.6353941 | 8.0317469 | 14.58484 | 25.371687 | 85.493347 | 299.40445 | 615.3114 | 1235.72 |
| 7 | IP-5 | 5.4129915 | 5.0848584 | 8.7838478 | 15.033156 | 25.32937 | 85.265182 | 248.60072 | 531.51331 | 1131.7911 |
| 7 | IP-6 | 3.4041092 | 4.5099564 | 7.9175787 | 13.632281 | 28.451691 | 94.902687 | 276.76282 | 572.53888 | 1196.533 |
| 7 | IP-7 | 3.2968762 | 4.3794899 | 7.8822885 | 13.594273 | 28.324898 | 96.571205 | 290.28195 | 602.42609 | 1227.4407 |
| 7 | IP-8 | 4.3549337 | 4.2857504 | 7.7576189 | 13.829926 | 27.726192 | 90.979446 | 257.68091 | 510.80737 | 1106.1797 |
| 7 | P-1 | 2.1411052 | 5.5324831 | 8.6443481 | 16.585964 | 82.407417 | 383.09375 | 1089.9769 | 1703.3809 | 2852.2241 |
| 7 | P-2 | 2.5079167 | 5.6423793 | 8.1412544 | 17.892174 | 89.832733 | 302.93918 | 985.48926 | 1577.5571 | 2672.217 |
| 7 | P-3 | 3.1223307 | 5.6732979 | 8.3687563 | 18.144022 | 90.641747 | 293.23007 | 968.52063 | 1568.3651 | 2668.4531 |
| 7 | P-4 | 3.4108844 | 5.6903329 | 8.1585255 | 18.411711 | 93.667557 | 360.31998 | 1088.1514 | 1701.6904 | 2839.7295 |
| 7 | P-5 | 2.6238897 | 5.5811648 | 8.0853987 | 18.806532 | 94.760811 | 310.05307 | 1012.8043 | 1629.2661 | 2766.5576 |
| 7 | TA'-4 | 1.4731603 | 4.0121036 | 8.800478 | 17.196249 | 18.545233 | 60.700512 | 233.93761 | 547.76801 | 1013.2968 |
| 7 | TA'-5 | 1.4130735 | 3.9817266 | 8.5546436 | 16.808428 | 18.303825 | 60.039497 | 233.46776 | 541.94208 | 997.17017 |
| 7 | TA'-6 | 1.3562429 | 3.915396 | 8.8153782 | 17.989231 | 18.827614 | 49.550106 | 257.40634 | 577.06927 | 1082.0089 |
| 7 | TA'-7 | 1.3157471 | 3.8969305 | 8.6293421 | 17.463984 | 18.29538 | 46.046219 | 149.51035 | 364.19205 | 739.65948 |
| 7 | TA'-8 | 1.3217226 | 3.8497443 | 9.2436228 | 19.205339 | 20.116348 | 39.055145 | 138.20438 | 363.24384 | 733.77362 |
| 7 | TH'-6 | 1.7549399 | 4.9417901 | 9.3693895 | 15.756805 | 37.586456 | 144.2538 | 501.82175 | 968.75336 | 1674.1924 |
| 7 | TH'-7 | 1.9214555 | 4.9444304 | 9.2957773 | 15.766685 | 37.856812 | 141.37808 | 526.71008 | 995.16339 | 1741.4901 |
| 7 | TH'-8 | 1.310662 | 4.7307415 | 9.5138779 | 16.213486 | 34.535797 | 137.29861 | 473.1261 | 933.08032 | 1626.4039 |
| 7 | TH'-9 | 1.9303708 | 4.9495583 | 9.0740795 | 15.103683 | 42.237865 | 153.61475 | 483.7182 | 941.56134 | 1674.2446 |
| 7 | TH'-10 | 1.3266855 | 4.5086646 | 9.4105959 | 16.724726 | 27.76589 | 104.8006 | 308.15598 | 671.33289 | 1264.5748 |
| 8 | OF-1 | 1.1090307 | 13.08075 | 14.461871 | 15.620664 | 23.34186 | 96.098701 | 557.22455 | 1456.5634 | 2247.7546 |
| 8 | OF-2 | 1.1772696 | 13.33648 | 14.867057 | 16.132931 | 24.079592 | 73.551155 | 518.03235 | 1470.465 | 2182.699 |
| 8 | OF-3 | 0.8128793 | 13.346413 | 14.815743 | 15.693262 | 23.329288 | 44.764034 | 283.64709 | 832.89282 | 1588.0487 |
| 8 | OF-4 | 0.7517473 | 13.370901 | 14.599569 | 15.303355 | 22.313288 | 57.431286 | 325.15372 | 969.39886 | 1757.1683 |
| 8 | OF-5 | 1.0802875 | 13.396352 | 14.609174 | 15.193395 | 21.98826 | 101.11995 | 581.7157 | 1492.4351 | 2231.3923 |
| 8 | OF-6 | 1.0203671 | 14.194682 | 15.951742 | 17.134306 | 25.687347 | 126.32223 | 598.1394 | 1480.7552 | 2181.345 |
| 8 | OF-7 | 1.9243224 | 14.227057 | 15.945669 | 16.657303 | 24.622749 | 273.59311 | 1365.2528 | 2257.9045 | 3163.9292 |
| 8 | OF-8 | 2.6363125 | 13.759758 | 14.77198 | 15.216839 | 21.398455 | 201.77287 | 905.66644 | 1676.8318 | 2538.4949 |
| 8 | OF-9 | 1.6566715 | 12.967568 | 13.56181 | 18.514288 | 18.707226 | 149.59595 | 695.57654 | 1695.2006 | 2463.9189 |
| 8 | OF-10 | 1.1927066 | 14.039541 | 14.802034 | 14.901294 | 20.577919 | 427.41602 | 1679.1023 | 2673.9763 | 3680.1626 |
| 8 | TA1-5 | 0.8910515 | 0.9031776 | 0.9848818 | 1.0877939 | 1.1375194 | 1.1888481 | 1.1733099 | 1.3087344 | 2.204879 |
| 8 | TA1-6 | 0.3130924 | 0.3021306 | 0.4011813 | 0.489305 | 0.4790923 | 0.5801626 | 0.4839272 | 0.7295815 | 1.8676555 |
| 8 | TA1-7 (Ground) | 2.0061164 | 2.1614788 | 7.3203807 | 5.6652741 | 16.529709 | 13.161063 | 33.779984 | 73.074852 | 108.83717 |
| 8 | TA1-8 (Common) | 2.0064301 | 2.1621792 | 7.2693887 | 5.6040797 | 16.384903 | 13.034085 | 33.551479 | 72.817665 | 108.82765 |
| 8 | TA1-9 | 0.3473597 | 0.3513196 | 0.5430034 | 0.8416036 | 1.6399666 | 1.3388447 | 2.8335037 | 5.4727387 | 8.4649153 |
| 8 | TA1-10 | 0.7136844 | 0.6900778 | 0.8707271 | 0.9458333 | 1.3786149 | 1.0628616 | 1.8760124 | 3.486316 | 5.7253432 |
| 8 | TA1-11 | 0.6904316 | 0.699405 | 0.9122679 | 1.1351167 | 1.8825783 | 1.4501606 | 2.8616667 | 5.3232203 | 8.3337402 |
| 8 | TA1-12 | 0.7956522 | 0.7450215 | 0.9210911 | 1.3436942 | 2.1230621 | 1.7325701 | 3.2630656 | 5.6871471 | 8.8574953 |
| 8 | TA2-4 | 0.8133419 | 0.9509922 | 1.355485 | 3.3039386 | 4.5630946 | 6.3884339 | 9.3564787 | 11.345562 | 12.52239 |
| 8 | TA2-5 | 0.4745238 | 0.5414755 | 0.7068803 | 3.4134593 | 4.9988246 | 7.0644302 | 10.620749 | 13.555204 | 15.299041 |
| 8 | TA2-6 | 0.4191357 | 0.4356681 | 0.6011305 | 3.0173333 | 4.2691092 | 6.4105763 | 9.4087543 | 11.612407 | 12.575831 |
| 8 | TA2-7 | 0.3033559 | 0.3526591 | 0.5230338 | 2.6378083 | 3.7351334 | 5.8318267 | 8.5060635 | 10.39432 | 10.878121 |
| 8 | TA2-8 | 0.3320835 | 0.3503444 | 0.5039545 | 2.0117719 | 2.765058 | 4.7540154 | 6.7960253 | 8.0491409 | 7.9443097 |
| 8 | TA2-9 | 0.5003137 | 0.5060957 | 0.6439761 | 1.5957646 | 2.1215069 | 4.1021428 | 5.8358116 | 7.010551 | 6.6524105 |
| 8 | TA2-10 | 1.0378067 | 0.9637299 | 1.081848 | 1.6673393 | 2.0480392 | 4.0279455 | 5.60882 | 6.7169566 | 6.1898255 |
| 8 | TA2-11 | 0.861187 | 0.8694418 | 1.0163093 | 1.4968072 | 1.7631319 | 3.9486587 | 5.3890195 | 6.126328 | 5.1324892 |
| 8 | TA2-12 | 0.9063743 | 0.8714277 | 0.9918401 | 1.683499 | 2.1117096 | 4.7639694 | 6.5965738 | 7.7228518 | 6.6607885 |
| 8 | TH1-5 | 1.0753685 | 1.1056795 | 1.3339305 | 5.4205227 | 7.9140759 | 11.828931 | 17.806547 | 22.940474 | 25.333326 |
| 8 | TH1-6 | 1.0172209 | 1.0363185 | 1.2606788 | 5.274528 | 7.7047181 | 11.759019 | 17.555332 | 22.517054 | 24.226957 |
| 8 | TH1-7 | 0.7968475 | 0.8750386 | 1.2909609 | 4.9898853 | 7.3010845 | 11.376323 | 17.143795 | 22.386942 | 24.701509 |
| 8 | TH1-8 | 0.4917692 | 0.5284417 | 0.7797923 | 4.3864536 | 6.3745852 | 10.517137 | 15.69611 | 20.018307 | 21.22497 |
| 8 | TH1-9 | 0.5246469 | 0.5527598 | 0.7660595 | 3.7095587 | 5.3167138 | 9.3480349 | 13.801917 | 17.397104 | 18.113033 |
| 8 | TH1-10 | 0.6482245 | 0.7422753 | 0.9297641 | 3.5766959 | 5.2288022 | 9.3478422 | 13.984501 | 17.998142 | 18.621395 |
| 8 | TH1-11 | 1.0122745 | 1.0369726 | 1.1809726 | 3.3146019 | 4.5690084 | 8.740922 | 12.737079 | 15.566351 | 15.003475 |
| 8 | TH1-12 | 0.9493912 | 0.9576206 | 1.1547627 | 3.3188932 | 4.6546407 | 9.0666151 | 13.25935 | 16.436588 | 16.054054 |
| 8 | TH-5 | 1.1178818 | 1.1569173 | 1.3818072 | 7.480638 | 11.071135 | 16.534464 | 24.99452 | 32.062977 | 34.581039 |
| 8 | TH-6 | 1.2329788 | 1.2191657 | 1.4486727 | 7.4374189 | 11.187717 | 16.942083 | 25.783581 | 33.568726 | 36.594227 |
| 8 | TH-7 | 1.0769999 | 1.0812639 | 1.3169122 | 7.1444874 | 10.617806 | 16.532652 | 24.981335 | 32.358513 | 34.855263 |
| 8 | TH-8 | 0.7587082 | 0.7796506 | 1.1070484 | 6.8335385 | 10.152264 | 16.246559 | 24.432673 | 31.152079 | 32.610348 |
| 8 | TH-9 | 0.6499066 | 0.7287993 | 1.0440329 | 6.4302979 | 9.4673624 | 15.659752 | 23.373085 | 29.361204 | 30.247129 |
| 8 | TH-10 | 0.7320743 | 0.7033848 | 1.0008348 | 5.8043447 | 8.6012096 | 14.671536 | 21.93539 | 27.667126 | 28.265995 |
| 8 | TH-11 | 0.9904927 | 0.8541449 | 1.0928375 | 5.4646573 | 8.063324 | 14.150865 | 21.134016 | 26.803768 | 27.722904 |
| 8 | TH-12 | 1.1578023 | 1.0319949 | 1.2206562 | 5.4404321 | 7.95258 | 14.303615 | 21.152901 | 26.398193 | 26.412849 |
| 8 | OH-1 | 0.9569715 | 1.0510589 | 1.470086 | 2.2004273 | 2.9724422 | 3.7596653 | 5.4517984 | 6.7646809 | 7.5237079 |
| 8 | OH-2 | 0.779074 | 0.9613062 | 1.3650993 | 3.1833217 | 4.523931 | 6.1431885 | 9.1576462 | 11.808467 | 13.207903 |
| 8 | OH-3 | 0.9187231 | 1.0605998 | 1.2467921 | 3.9384007 | 5.535615 | 7.8750868 | 11.526664 | 14.198391 | 15.537951 |
| 8 | OH-4 | 1.945062 | 1.9304562 | 2.0289493 | 4.9328427 | 7.1102519 | 9.8113174 | 14.824608 | 19.285572 | 22.162144 |
| 8 | OH-5 | 1.6931119 | 1.7447753 | 1.7716649 | 5.7372007 | 8.5944767 | 11.991553 | 18.258942 | 24.411846 | 27.96892 |
| 8 | OH-6 | 0.8509779 | 0.9749824 | 1.1963283 | 6.3583055 | 9.3712568 | 13.709586 | 20.424084 | 25.74485 | 27.832767 |
| 8 | OH-7 | 0.9378886 | 1.0098916 | 1.3315907 | 7.5444117 | 11.267159 | 16.40852 | 24.804197 | 31.955254 | 34.959904 |
| 8 | OH-8 | 1.0122631 | 1.0573392 | 1.3450458 | 8.109127 | 11.979818 | 17.634134 | 26.203491 | 32.252106 | 33.376842 |
| 8 | OH-9 | 1.241758 | 1.2067785 | 1.5463599 | 9.2692347 | 14.035996 | 20.347195 | 31.098457 | 40.609612 | 44.87516 |
| 8 | OH-10 | 1.280003 | 1.3173707 | 1.6441001 | 9.6547289 | 14.434607 | 21.207169 | 31.864868 | 39.996059 | 42.269096 |
| 8 | OH-11 | 1.4963204 | 1.3977048 | 1.7791195 | 10.11015 | 15.113631 | 22.183338 | 33.22377 | 41.558437 | 43.058617 |
| 8 | OH-12 | 1.7054362 | 1.6501898 | 2.0217986 | 10.688821 | 16.011723 | 23.432146 | 34.937252 | 42.954041 | 43.837299 |
| 8 | OH-13 | 1.1955736 | 1.2723569 | 1.739663 | 11.094816 | 16.574286 | 24.363935 | 36.203983 | 43.751728 | 43.821033 |
| 8 | OH-14 | 0.872156 | 0.8207259 | 1.4414757 | 11.294753 | 16.927694 | 25.107172 | 37.243591 | 45.08617 | 44.897346 |
| 8 | OH-15 | 0.8780485 | 0.8044057 | 1.4794943 | 11.955735 | 17.888977 | 26.704437 | 39.235435 | 46.224903 | 44.381416 |
| 9 | OF'-1 | 2.8060145 | 3.4389083 | 25.058893 | 58.436924 | 88.841492 | 86.220047 | 75.101227 | 119.65094 | 120.53316 |
| 9 | OF'-2 | 3.2939851 | 3.8134158 | 24.78417 | 58.084362 | 89.967377 | 87.469231 | 77.536057 | 125.15799 | 124.68453 |
| 9 | OF'-3 | 2.6788862 | 3.3974669 | 24.401379 | 57.394474 | 90.154175 | 87.865219 | 78.777863 | 128.54636 | 128.41403 |
| 9 | OF'-4 (Broken) | 11.686199 | 99.499687 | 109.3007 | 103.85461 | 105.08943 | 93.641571 | 105.99679 | 123.15001 | 135.28459 |
| 9 | OF'-5 | 1.8994514 | 3.1063368 | 23.554674 | 55.153988 | 85.626358 | 83.438988 | 73.903099 | 119.05511 | 118.478 |
| 9 | OF'-6 | 2.6278977 | 3.7106988 | 23.3433 | 54.931881 | 87.862099 | 85.873436 | 77.901962 | 128.50635 | 129.2207 |
| 9 | OF'-7 | 2.8997517 | 3.5301559 | 23.09449 | 54.364773 | 86.440071 | 84.47419 | 76.332947 | 125.4302 | 125.63522 |
| 9 | OF'-8 | 2.8635271 | 3.5487514 | 22.863638 | 53.834423 | 85.737572 | 83.912712 | 75.997803 | 125.11796 | 125.57551 |
| 9 | OF'-9 | 3.1281333 | 3.9186912 | 22.867189 | 53.638756 | 85.054512 | 83.113792 | 74.929222 | 122.67287 | 122.49434 |
| 9 | OF'-10 | 2.1894171 | 3.1881719 | 23.03698 | 54.033985 | 85.930466 | 84.059006 | 75.883202 | 124.44073 | 124.56935 |
| 9 | TA1'-4 | 1.1444685 | 1.3668501 | 8.9277124 | 21.241161 | 37.796379 | 36.784058 | 37.19371 | 68.238388 | 77.333534 |
| 9 | TA1'-5 | 1.0460057 | 1.2917578 | 9.0545101 | 21.608078 | 38.543667 | 37.600998 | 38.029388 | 69.91806 | 79.393394 |
| 9 | TA1'-6 | 0.954141 | 1.2496781 | 9.2737694 | 22.287033 | 39.889107 | 39.000027 | 39.498333 | 72.809624 | 82.973183 |
| 9 | TA1'-7 | 0.9029714 | 1.2287339 | 9.4488525 | 22.770735 | 40.682903 | 39.845596 | 40.245472 | 73.984566 | 84.156769 |
| 9 | TA1'-8 | 0.9122485 | 1.2340136 | 9.4703541 | 22.85438 | 40.873909 | 40.093399 | 40.483017 | 74.452248 | 84.807648 |
| 9 | TA1'-9 | 1.1142739 | 1.4085811 | 9.5674372 | 23.099379 | 41.315533 | 40.583832 | 40.974773 | 75.297699 | 85.823914 |
| 9 | TA1'-10 | 1.3125092 | 1.6102911 | 9.5960531 | 23.039589 | 41.136734 | 40.448242 | 40.810436 | 74.991852 | 85.472672 |
| 9 | TA1'-11 | 1.2039077 | 1.441662 | 9.198514 | 22.183168 | 39.691837 | 39.084229 | 39.374187 | 72.443604 | 82.6409 |
| 9 | TA1'-12 | 1.2812288 | 1.463856 | 9.0002928 | 21.715607 | 38.938473 | 38.388416 | 38.749878 | 71.381126 | 81.635132 |
| 9 | TA2'-4 | 1.0668341 | 1.203307 | 6.6260719 | 15.634704 | 28.04771 | 27.286598 | 27.859341 | 51.504818 | 58.813595 |
| 9 | TA2'-5 | 0.9881701 | 1.1200477 | 6.6496205 | 15.807252 | 28.318104 | 27.577335 | 28.110693 | 51.910809 | 59.244217 |
| 9 | TA2'-6 | 0.9638368 | 1.1052374 | 6.9533281 | 16.686129 | 30.019684 | 29.303545 | 29.909506 | 55.412868 | 63.474751 |
| 9 | TA2'-7 | 0.8640072 | 1.0489063 | 7.2694178 | 17.495554 | 31.549305 | 30.880745 | 31.4737 | 58.350323 | 66.957253 |
| 9 | TA2'-8 | 0.8696054 | 1.0486887 | 7.3786411 | 17.783934 | 31.997904 | 31.396898 | 31.902945 | 59.028389 | 67.673584 |
| 9 | TA2'-9 | 1.1778761 | 1.3472742 | 7.2383394 | 17.340681 | 31.320097 | 30.81336 | 31.342201 | 58.118534 | 66.917473 |
| 9 | TA2'-10 | 1.3990505 | 1.5404983 | 7.1021724 | 16.941738 | 30.674513 | 30.216064 | 30.780041 | 57.061237 | 65.813248 |
| 9 | TA2'-11 | 1.3442751 | 1.5615035 | 6.9679794 | 16.5434 | 29.93372 | 29.542496 | 30.031336 | 55.702583 | 64.275772 |
| 9 | TA2'-12 | 1.347383 | 1.5106936 | 6.7938228 | 16.244028 | 29.424984 | 29.099703 | 29.572857 | 54.917473 | 63.457825 |
| 9 | TH1'-4 | 1.0069573 | 1.1526308 | 5.9225044 | 13.963575 | 25.16464 | 24.439062 | 25.047646 | 46.424583 | 53.194256 |
| 9 | TH1'-5 | 1.2870618 | 1.386309 | 5.9974813 | 14.14275 | 25.486475 | 24.82378 | 25.407669 | 47.047123 | 54.015549 |
| 9 | TH1'-6 | 1.199206 | 1.3031394 | 6.0231881 | 14.232949 | 25.645828 | 25.058905 | 25.608019 | 47.521336 | 54.516155 |
| 9 | TH1'-7 | 0.8738652 | 1.0293068 | 6.23032 | 14.897252 | 26.879316 | 26.300144 | 26.853802 | 49.776237 | 57.151688 |
| 9 | TH1'-8 | 0.9870025 | 1.1115158 | 6.5584192 | 15.725839 | 28.404312 | 27.905111 | 28.446003 | 52.787647 | 60.753044 |
| 9 | TH1'-9 | 1.7006687 | 1.8858407 | 6.6570668 | 15.758148 | 28.399279 | 27.916586 | 28.494192 | 52.886509 | 60.935863 |
| 9 | TH1'-10 | 1.6641322 | 1.8548913 | 6.59973 | 15.571006 | 28.072104 | 27.656683 | 28.166576 | 52.241001 | 60.258904 |
| 9 | TH1'-11 | 1.5804647 | 1.8977687 | 6.549314 | 15.354265 | 27.820284 | 27.467405 | 28.007933 | 52.034306 | 60.241768 |
| 9 | TH1'-12 | 1.4295241 | 1.517905 | 6.3682189 | 15.161328 | 27.471966 | 27.240602 | 27.651897 | 51.297131 | 59.336823 |
| 9 | TH'-5 | 1.2026981 | 1.219678 | 4.3898187 | 10.149585 | 18.26862 | 17.720844 | 18.256081 | 33.797161 | 38.757286 |
| 9 | TH'-6 | 1.4794933 | 1.3349187 | 4.4688768 | 10.387633 | 18.69306 | 18.216007 | 18.686741 | 34.678795 | 39.715176 |
| 9 | TH'-7 | 0.9869932 | 1.0146667 | 4.684063 | 11.09387 | 20.069315 | 19.603502 | 20.124603 | 37.418621 | 43.031654 |
| 9 | TH'-8 | 1.0136182 | 1.0466232 | 5.1938868 | 12.386276 | 22.474669 | 22.054718 | 22.579895 | 42.007244 | 48.472382 |
| 9 | TH'-9 | 1.1448439 | 1.3314449 | 5.2810936 | 12.559298 | 22.878466 | 22.52368 | 23.020775 | 42.81601 | 49.556469 |
| 9 | TH'-10 | 1.7570097 | 1.8103361 | 5.3463783 | 12.377708 | 22.396097 | 22.143248 | 22.587584 | 42.029064 | 48.681034 |
| 9 | TH'-11 | 1.4800013 | 1.615459 | 5.2409148 | 12.226184 | 22.144571 | 21.938881 | 22.363144 | 41.646164 | 48.303131 |
| 9 | TH'-12 | 1.2258141 | 1.3091856 | 5.2013035 | 12.309788 | 22.384251 | 22.235216 | 22.633348 | 42.103687 | 48.840248 |
| 9 | OH'-1 | 1.2655216 | 1.4377966 | 8.2975111 | 19.436951 | 34.806671 | 33.778454 | 34.381481 | 63.45557 | 72.240379 |
| 9 | OH'-2 | 1.2671126 | 1.3929273 | 7.5726218 | 17.721107 | 31.788103 | 30.867262 | 31.501991 | 58.251598 | 66.439697 |
| 9 | OH'-3 | 1.4099722 | 1.4404609 | 6.8366728 | 15.902368 | 28.502993 | 27.680044 | 28.290936 | 52.307899 | 59.67347 |
| 9 | OH'-4 | 1.165454 | 1.3209338 | 6.327312 | 14.758857 | 26.503962 | 25.775846 | 26.359686 | 48.814831 | 55.763378 |
| 9 | OH'-5 | 1.7311914 | 1.9264683 | 6.1911664 | 13.015223 | 23.349741 | 22.67761 | 23.123581 | 42.942993 | 48.957279 |
| 9 | OH'-6 | 1.5682491 | 1.63397 | 6.0513515 | 11.634924 | 20.936176 | 20.313257 | 20.780558 | 38.522694 | 44.025658 |
| 9 | OH'-7 | 1.3722142 | 1.3576187 | 4.385191 | 9.4581957 | 17.035555 | 16.518341 | 16.954863 | 31.350758 | 35.799988 |
| 9 | OH'-8 | 1.2203948 | 1.2203134 | 3.6041145 | 7.6946635 | 13.822241 | 13.350302 | 13.781726 | 25.48439 | 29.049555 |
| 9 | OH'-9 | 1.0089457 | 0.9830818 | 3.0240018 | 6.6360369 | 11.836968 | 11.430379 | 11.807032 | 21.779696 | 24.843386 |
| 9 | OH'-10 | 1.0570109 | 0.9930719 | 2.5581336 | 5.4708924 | 9.7527037 | 9.4134779 | 9.7343998 | 17.925495 | 20.482731 |
| 9 | OH'-11 | 0.7530831 | 0.6815518 | 2.1051767 | 4.5586653 | 8.1874037 | 7.8775148 | 8.1637363 | 15.068599 | 17.21537 |
| 9 | OH'-12 | 0.6482056 | 0.5810571 | 1.6896211 | 3.6242766 | 6.4944363 | 6.2466407 | 6.4666953 | 11.95475 | 13.648385 |
| 9 | OH'-13 | 0.5547279 | 0.4935867 | 1.0436081 | 2.275177 | 4.0593295 | 3.8976414 | 4.0527177 | 7.527595 | 8.6369762 |
| 9 | OH'-14 (Ground) | 9.7745142 | 77.986488 | 141.05324 | 100.53645 | 105.20273 | 101.07716 | 106.5034 | 123.81504 | 134.01369 |
| 9 | OH'-15 (Common) | 10.689168 | 91.757484 | 124.74583 | 87.312294 | 111.48003 | 114.3373 | 114.34129 | 131.23138 | 155.11032 |
| 10 | B-3 | 1.4802008 | 2.4604368 | 10.359747 | 18.323195 | 24.77092 | 30.122614 | 26.274757 | 54.285904 | 211.6207 |
| 10 | B-4 | 1.4235756 | 2.454519 | 10.571624 | 18.737392 | 24.879755 | 29.027531 | 27.717678 | 15.913002 | 106.00272 |
| 10 | B-5 | 1.6787779 | 2.7589827 | 11.032366 | 19.003653 | 23.475523 | 24.154135 | 39.98428 | 282.10089 | 860.77148 |
| 10 | B-6 | 1.8060064 | 2.8101327 | 11.328696 | 20.036169 | 25.926367 | 30.594158 | 17.677473 | 262.14667 | 855.5957 |
| 10 | B-7 | 2.0230155 | 2.9238813 | 11.415594 | 19.901327 | 25.324799 | 24.168505 | 92.158646 | 523.47748 | 1053.387 |
| 10 | B-8 | 2.0049443 | 3.0470653 | 11.324296 | 19.774059 | 25.445925 | 32.012222 | 20.414785 | 261.3743 | 904.10541 |
| 10 | TH-5 | 0.9948195 | 2.0459666 | 2.0875528 | 2.4671054 | 5.2871847 | 11.653136 | 22.262392 | 36.72007 | 56.352085 |
| 10 | TH-6 | 0.7302602 | 1.8846204 | 1.9244725 | 2.4592345 | 5.2386117 | 10.535704 | 18.90053 | 30.427023 | 44.649231 |
| 10 | TH-7 (Ground) | 2.0778461 | 2.3412254 | 2.3472497 | 2.4278057 | 2.5265999 | 2.9052522 | 4.0617399 | 7.5118141 | 15.918041 |
| 10 | TH-8 (Common) | 2.0868096 | 2.3356395 | 2.3384333 | 2.4177396 | 2.5187781 | 2.9326603 | 4.2493815 | 8.0058985 | 16.986605 |
| 10 | TH-9 | 0.6841077 | 1.8618295 | 2.4224761 | 4.6400847 | 10.069191 | 21.248865 | 38.263729 | 58.990265 | 87.6604 |
| 10 | TH-10 | 0.8306436 | 1.9367 | 2.1682596 | 3.6916542 | 8.2448521 | 17.477217 | 31.403904 | 50.314678 | 74.351334 |
| 10 | TH-11 | 1.245068 | 2.1278605 | 2.1955802 | 3.3680043 | 7.8551273 | 17.958673 | 33.645233 | 54.666019 | 84.236008 |
| 10 | TH-12 | 1.5570208 | 2.4408829 | 2.4958463 | 3.1893992 | 7.4133596 | 17.382301 | 33.377144 | 56.59206 | 87.831612 |
| 10 | P-1 | 1.3754154 | 2.2341065 | 4.6543489 | 6.6983252 | 5.9822721 | 4.1954465 | 10.661182 | 27.568493 | 49.73811 |
| 10 | P-2 | 2.1137354 | 2.5212383 | 4.4953432 | 5.9249668 | 4.5401282 | 4.5026441 | 14.845768 | 34.030357 | 58.170444 |
| 10 | P-3 | 2.118078 | 2.6049299 | 4.6777964 | 6.3460312 | 5.368484 | 4.3068366 | 12.804503 | 31.835484 | 58.767277 |
| 10 | P-4 | 1.8015308 | 2.3923819 | 4.9775109 | 6.8844872 | 5.5782838 | 4.8187666 | 15.582813 | 35.993237 | 60.350384 |
| 10 | P-5 | 1.3691275 | 2.2701664 | 5.4734931 | 7.9800692 | 7.5572014 | 5.3851595 | 11.28839 | 29.45587 | 51.789318 |
| 10 | P-6 | 1.5589734 | 2.4312365 | 6.1710649 | 9.1099014 | 9.0879011 | 6.900835 | 10.151566 | 29.346212 | 54.912491 |
| 10 | P-7 | 2.3056738 | 2.7898533 | 6.5460095 | 9.6829796 | 9.7841082 | 7.5022454 | 10.721937 | 29.345739 | 53.413841 |
| 10 | P-8 | 2.3928454 | 2.9008193 | 6.7709475 | 9.9449005 | 9.7709332 | 7.6902857 | 14.028777 | 36.2906 | 63.723679 |
| 10 | PO-1 | 1.3839059 | 2.3317444 | 4.6880202 | 6.2242613 | 4.8131199 | 4.943881 | 15.711171 | 34.390263 | 58.786026 |
| 10 | PO-2 | 1.5475289 | 2.4064724 | 4.8487983 | 6.4490952 | 4.8315506 | 5.3734818 | 17.233379 | 37.055782 | 60.566463 |
| 10 | PO-3 | 1.6334648 | 2.5466361 | 4.848341 | 6.2416759 | 4.5994549 | 6.5894985 | 19.980919 | 41.199066 | 67.753258 |
| 10 | PO-4 | 1.7805272 | 2.6208453 | 5.2102571 | 6.8506656 | 5.3046212 | 5.4857478 | 17.119856 | 37.80143 | 63.415096 |
| 10 | PO-5 | 1.6424183 | 2.4730799 | 5.2441754 | 7.2540421 | 6.0704522 | 5.229177 | 15.402523 | 34.46283 | 57.753761 |
| 10 | PO-6 | 2.1493478 | 2.7604818 | 5.5463324 | 7.7606001 | 6.9797869 | 5.3552217 | 12.880836 | 34.495304 | 64.41227 |
| 10 | PO-7 | 2.1425524 | 2.8129342 | 5.693377 | 7.8901997 | 6.7120934 | 6.0100327 | 17.363163 | 38.07827 | 63.228333 |
| 10 | PO-8 | 1.4601383 | 2.4222467 | 7.6608195 | 11.887029 | 12.408719 | 10.715021 | 9.9786148 | 29.129282 | 57.141823 |
| 10 | TO-1 | 4.9678102 | 4.938406 | 12.408211 | 20.650856 | 23.545162 | 22.803627 | 13.364554 | 38.142708 | 90.178612 |
| 10 | TO-2 | 4.4279575 | 5.0017748 | 12.591208 | 21.001156 | 23.608358 | 21.797653 | 12.950973 | 43.145554 | 94.64624 |
| 10 | TO-3 | 3.6494887 | 4.2602429 | 12.497778 | 20.641659 | 23.181026 | 21.574566 | 12.813407 | 39.474926 | 87.954163 |
| 10 | TO-4 | 1.7856997 | 2.7617288 | 12.615056 | 20.696796 | 22.64517 | 20.229982 | 12.981336 | 45.296326 | 97.732918 |
| 10 | TO-5 | 1.4285171 | 2.5357499 | 13.495347 | 21.809223 | 22.679811 | 17.549843 | 18.201284 | 61.909527 | 122.07166 |
| 10 | TO-6 | 1.7402266 | 2.8029225 | 14.880679 | 24.779514 | 27.837986 | 25.808838 | 14.901735 | 48.331657 | 110.0091 |
| 10 | TO-7 | 2.2292874 | 3.1900053 | 15.297051 | 25.436806 | 28.882591 | 26.944582 | 15.410759 | 48.546822 | 112.61753 |
| 10 | TO-8 | 1.9928941 | 3.1806343 | 14.974997 | 23.961821 | 23.947527 | 17.183781 | 24.828465 | 80.616272 | 154.79097 |
| 10 | TO-9 | 1.722178 | 2.9262784 | 15.464167 | 25.222265 | 26.983536 | 21.892271 | 18.332497 | 67.472061 | 137.67914 |
| 10 | TO-10 | 1.8255321 | 2.9226611 | 15.434248 | 24.836008 | 25.243477 | 18.584379 | 24.218897 | 79.838615 | 154.32018 |
| 10 | V-1 | 2.87606 | 3.5886261 | 3.2647321 | 3.3835065 | 6.2551045 | 13.576996 | 27.197847 | 44.969147 | 69.564934 |
| 10 | V-2 | 2.3565762 | 2.9831839 | 2.8162088 | 2.9690096 | 6.672277 | 14.186471 | 27.826971 | 45.889668 | 69.072029 |
| 10 | V-3 | 2.0862284 | 2.9330461 | 2.8249688 | 2.898406 | 6.2296882 | 13.810202 | 27.750179 | 45.540802 | 69.055534 |
| 10 | V-4 | 1.5886115 | 2.454423 | 2.6505363 | 2.5088668 | 5.1420794 | 11.68466 | 24.099228 | 41.082985 | 62.494385 |
| 10 | V-5 | 1.5708565 | 2.4206617 | 2.8527582 | 2.6905015 | 4.9214044 | 12.808246 | 27.825039 | 47.301323 | 72.315262 |
| 10 | V-6 | 1.760574 | 2.502665 | 3.3332298 | 3.1930933 | 4.7401547 | 12.697176 | 28.5151 | 50.09277 | 76.164238 |
| 10 | V-7 | 2.1191533 | 2.8516715 | 3.9657526 | 3.9752419 | 4.2508159 | 11.380354 | 27.040339 | 47.653072 | 72.747307 |
| 10 | V-8 | 1.9699935 | 2.7470288 | 3.8784635 | 3.9847498 | 3.9981 | 10.414231 | 25.152958 | 45.972626 | 71.696892 |
| 10 | V-9 | 2.3447366 | 3.1738198 | 4.724925 | 4.8841934 | 4.5519109 | 7.9891829 | 20.241386 | 37.704136 | 59.981934 |
| 10 | V-10 | 2.4760253 | 3.1818855 | 4.5807757 | 5.0983863 | 4.2849221 | 6.2112975 | 16.943415 | 34.692402 | 58.86792 |
| 10 | B'-3 | 2.0599687 | 2.9936764 | 14.963936 | 22.925003 | 15.323984 | 122.84785 | 769.40625 | 1738.3905 | 2689.8914 |
| 10 | B'-4 | 1.6404066 | 2.8496649 | 14.863264 | 20.289785 | 15.341847 | 122.06917 | 681.8399 | 1869.8289 | 2970.7092 |
| 10 | B'-5 | 1.609539 | 2.9006646 | 16.50292 | 24.160641 | 30.309698 | 310.64108 | 1425.5181 | 2466.5579 | 3605.2346 |
| 10 | B'-6 | 1.6717931 | 2.9210782 | 16.107721 | 20.459345 | 46.335522 | 367.78198 | 1665.5269 | 2861.135 | 4121.187 |
| 10 | B'-7 | 1.9824773 | 3.1652021 | 17.498466 | 21.297813 | 67.415459 | 511.92154 | 1922.4807 | 3174.1563 | 4521.9146 |
| 10 | B'-8 | 17.444782 | 48.201664 | 49.061092 | 50.045193 | 58.298443 | 62.358883 | 77.22364 | 76.665909 | 299.60162 |
| 10 | TH'-5 | 2.1403034 | 2.6723258 | 2.7504766 | 4.0673375 | 8.720953 | 15.014805 | 26.094166 | 40.930859 | 66.506866 |
| 10 | TH'-6 | 1.5875297 | 2.3751519 | 2.4303851 | 3.8500779 | 8.5784616 | 14.068659 | 24.409983 | 39.806049 | 66.610878 |
| 10 | TH'-7 | 1.6554085 | 2.4332156 | 2.579335 | 4.1697559 | 9.3680105 | 16.06344 | 28.036062 | 43.945026 | 72.057007 |
| 10 | TH'-8 | 1.7123628 | 2.4041672 | 2.528666 | 4.2911429 | 9.8985014 | 16.856689 | 29.441286 | 46.51931 | 76.030052 |
| 10 | TH'-9 | 1.5177945 | 2.2955232 | 2.6203477 | 4.9634442 | 11.241813 | 19.576273 | 33.623253 | 51.097702 | 82.794106 |
| 10 | TH'-10 | 2.0656948 | 2.304666 | 2.3099921 | 2.3957555 | 2.4868433 | 2.8217733 | 3.6588469 | 6.6548986 | 14.874359 |
| 10 | TH'-11 | 1.6592746 | 2.3533928 | 2.8633578 | 5.6267734 | 12.539835 | 21.970478 | 37.737354 | 56.938095 | 90.241867 |
| 10 | TH'-12 | 1.6322833 | 2.3626688 | 2.7943113 | 5.4212341 | 12.653061 | 22.529438 | 39.185616 | 60.674877 | 95.810707 |
| 10 | PO'-1 | 1.7210932 | 2.4989424 | 4.2179484 | 5.7816091 | 6.2123756 | 9.257225 | 19.933475 | 40.87212 | 75.024704 |
| 10 | PO'-2 | 2.0803995 | 2.7081544 | 4.3421564 | 5.8467646 | 6.3807755 | 9.5061922 | 19.739298 | 41.948299 | 81.486931 |
| 10 | PO'-3 | 2.7105038 | 2.9836702 | 4.4277496 | 5.7921839 | 6.641386 | 11.329895 | 24.394426 | 47.648403 | 84.8946 |
| 10 | PO'-4 | 2.4347396 | 2.7769535 | 4.4863949 | 5.8836617 | 7.1433077 | 13.312504 | 29.138762 | 56.254913 | 97.67791 |
| 10 | PO'-5 | 2.207233 | 2.7201617 | 4.4482012 | 6.2152834 | 7.5928512 | 12.186021 | 24.618002 | 51.692127 | 103.33815 |
| 10 | PO'-6 | 1.6903609 | 2.4650309 | 5.2697301 | 8.2212753 | 12.592231 | 27.79141 | 60.356136 | 116.80498 | 225.23112 |
| 10 | V'-1 | 3.6633112 | 4.4379134 | 7.3025336 | 10.134021 | 9.9750681 | 9.2858677 | 15.354259 | 38.824413 | 74.29174 |
| 10 | V'-2 | 4.3609166 | 5.5885072 | 7.7220907 | 10.667781 | 10.96393 | 10.771141 | 12.458405 | 32.459846 | 63.850899 |
| 10 | V'-3 | 4.3668952 | 5.7322059 | 7.6301022 | 10.280628 | 10.592499 | 10.371899 | 13.391397 | 33.267284 | 64.274811 |
| 10 | V'-4 | 6.6726999 | 8.8034039 | 10.241904 | 11.413656 | 12.296538 | 12.188129 | 15.293202 | 35.111256 | 65.953484 |
| 10 | V'-5 | 4.2340589 | 5.3700933 | 7.6566243 | 9.562314 | 9.8628635 | 9.5869713 | 14.941914 | 37.972588 | 79.847206 |
| 10 | V'-6 | 3.6372855 | 4.2120209 | 6.2031918 | 8.4008579 | 8.1208553 | 8.5327959 | 17.269098 | 40.315498 | 75.294449 |
| 10 | V'-7 | 2.4380856 | 3.0260124 | 5.3231373 | 7.1358547 | 6.8099065 | 8.813117 | 21.292234 | 44.953423 | 80.384361 |
| 10 | V'-8 | 2.2258651 | 3.079468 | 5.2734566 | 7.1822577 | 6.8576097 | 8.7317648 | 20.48527 | 43.947697 | 79.506706 |
| 10 | V'-9 | 2.2122145 | 3.2028291 | 4.9455857 | 6.668623 | 6.6044512 | 8.6029291 | 19.345005 | 40.780132 | 73.786911 |
| 10 | V'-10 | 2.1368949 | 2.9188323 | 4.7425628 | 6.4685192 | 6.4517155 | 8.8003464 | 19.688751 | 41.70208 | 75.050293 |
| 10 | V'-11 | 2.1814358 | 3.0265553 | 4.7322803 | 6.3883901 | 6.6979866 | 8.4343367 | 16.969803 | 40.279079 | 83.342491 |
| 10 | V'-12 | 1.9217122 | 2.6645617 | 4.4551129 | 5.9364095 | 6.4575038 | 10.29807 | 22.842199 | 46.393505 | 83.97657 |
| 11 | OF-1 | 1.8865403 | 2.8905497 | 21.410444 | 42.805 | 65.16581 | 89.668297 | 112.60616 | 131.59303 | 148.57423 |
| 11 | OF-2 | 1.6417401 | 2.5256224 | 21.429802 | 42.93964 | 65.631226 | 90.373764 | 113.47987 | 132.6747 | 149.7841 |
| 11 | OF-3 | 1.6289452 | 2.5186663 | 21.526592 | 43.219585 | 66.063858 | 91.184326 | 114.59306 | 133.81514 | 151.15671 |
| 11 | OF-4 | 1.9236188 | 2.7025704 | 21.69063 | 43.536427 | 66.694931 | 92.196037 | 115.94549 | 135.35925 | 152.7697 |
| 11 | OF-5 | 1.8541605 | 2.7526162 | 21.623798 | 43.396667 | 66.249046 | 91.420319 | 114.34544 | 132.62215 | 148.70638 |
| 11 | OF-6 | 1.8942945 | 2.7573619 | 21.858612 | 43.787899 | 66.923218 | 92.220879 | 114.8968 | 132.54082 | 148.04839 |
| 11 | OF-7 | 2.1528897 | 2.925844 | 21.749031 | 43.677151 | 66.84726 | 92.267532 | 115.0873 | 132.78833 | 148.42331 |
| 11 | OF-8 | 2.5183432 | 3.0855813 | 21.977571 | 44.288506 | 67.803383 | 93.746407 | 117.14134 | 135.41899 | 151.37529 |
| 11 | OF-9 | 2.3554432 | 3.0741241 | 22.292309 | 44.947052 | 68.388336 | 93.840897 | 115.9234 | 132.08025 | 145.5441 |
| 11 | OF-10 | 2.5446551 | 3.1407042 | 22.475533 | 45.209469 | 68.937431 | 94.746284 | 117.16993 | 133.83182 | 147.6154 |
| 11 | OF-11 | 1.948281 | 2.727649 | 22.627178 | 45.695808 | 69.950394 | 96.599472 | 120.18241 | 138.12122 | 153.55898 |
| 11 | OF-12 | 1.740805 | 2.6389484 | 23.097322 | 46.681889 | 71.464432 | 98.688095 | 122.5867 | 140.40781 | 155.73247 |
| 11 | TA-4 | 1.2184176 | 1.1578445 | 1.2607422 | 1.4505833 | 1.409422 | 1.3797078 | 1.5403041 | 2.0462983 | 2.2946882 |
| 11 | TA-5 | 0.6306189 | 0.5939307 | 0.6686858 | 0.8167285 | 0.7071264 | 0.6424706 | 0.6799102 | 0.9690695 | 1.142417 |
| 11 | TA-6 | 0.2979604 | 0.3305018 | 0.3721518 | 0.4381375 | 0.3752178 | 0.3422747 | 0.3566363 | 0.481701 | 0.5467544 |
| 11 | TA-7 (Ground) | 21.761848 | 89.001541 | 124.57545 | 112.74143 | 120.6088 | 120.13464 | 117.0722 | 103.35121 | 121.62875 |
| 11 | TA-8 (Common) | 21.639238 | 123.29594 | 119.65733 | 110.70512 | 128.76495 | 118.3825 | 121.44195 | 116.7673 | 117.71769 |
| 11 | TA-9 | 1.0754793 | 1.0720083 | 1.3765881 | 1.9183553 | 2.4395912 | 3.0430613 | 3.8108599 | 4.6515384 | 5.6301932 |
| 11 | TA-10 | 1.2266667 | 1.2124919 | 1.702764 | 2.5097599 | 3.3126142 | 4.2110677 | 5.3023238 | 6.7170429 | 7.8244553 |
| 11 | TA-11 | 0.9483635 | 0.9761593 | 1.7849303 | 2.7998552 | 3.8331921 | 4.8381395 | 6.1743193 | 7.9313998 | 9.3818398 |
| 11 | TA-12 | 1.2640846 | 1.316506 | 2.5872376 | 4.1633215 | 5.7954774 | 7.2454782 | 9.2635832 | 11.763488 | 13.921206 |
| 11 | TH-5 | 1.0721614 | 1.0598434 | 3.6663473 | 7.554811 | 11.927427 | 17.230577 | 21.95808 | 25.557734 | 29.27602 |
| 11 | TH-6 | 1.2807947 | 1.2962061 | 4.3501558 | 8.8065462 | 13.773396 | 19.771797 | 25.234152 | 29.513702 | 34.030121 |
| 11 | TH-7 | 1.093652 | 1.1860777 | 4.7795534 | 9.7346716 | 15.211951 | 21.539282 | 27.464474 | 32.487087 | 37.30275 |
| 11 | TH-8 | 0.7269996 | 0.8010046 | 4.830905 | 9.7461767 | 15.147993 | 21.36956 | 27.230862 | 32.387272 | 37.308468 |
| 11 | TH-9 | 0.7539449 | 0.8334346 | 5.1849785 | 10.308645 | 15.920172 | 22.215408 | 28.332552 | 33.927814 | 39.31472 |
| 11 | TH-10 | 1.3663232 | 1.4767168 | 6.050487 | 11.773355 | 18.076469 | 25.072809 | 31.956331 | 38.333042 | 44.645267 |
| 11 | TH-11 | 1.5931885 | 1.8892584 | 6.578599 | 12.65309 | 19.24437 | 26.542976 | 33.767445 | 40.625797 | 47.380798 |
| 11 | TH-12 | 1.7892678 | 1.8867035 | 7.0206332 | 13.413967 | 20.440752 | 28.082672 | 35.786938 | 43.248833 | 50.351021 |
| 11 | OF'-1 | 1.8546125 | 3.2313538 | 27.771666 | 55.454731 | 84.28965 | 115.47075 | 144.29225 | 167.85458 | 188.65445 |
| 11 | OF'-2 | 1.5007794 | 3.0309424 | 27.889183 | 55.487446 | 84.113396 | 114.90932 | 142.89801 | 165.35933 | 184.49342 |
| 11 | OF'-3 | 1.8168929 | 3.1623664 | 28.022568 | 55.700584 | 84.425468 | 115.42012 | 143.58298 | 166.01195 | 185.27728 |
| 11 | OF'-4 | 1.6064545 | 3.0717645 | 28.317888 | 56.288746 | 85.196983 | 116.68792 | 145.06723 | 167.81476 | 186.91624 |
| 11 | OF'-5 | 1.7330301 | 3.1122043 | 29.047756 | 57.607121 | 87.068237 | 119.23392 | 147.84784 | 170.53128 | 189.37082 |
| 11 | OF'-6 | 1.7339023 | 3.2484574 | 29.659475 | 58.695614 | 88.47187 | 120.68903 | 148.84836 | 170.61798 | 187.83223 |
| 11 | OF'-7 | 2.178354 | 3.4727759 | 30.404854 | 60.044453 | 90.574005 | 123.86962 | 152.94762 | 175.47174 | 193.29973 |
| 11 | OF'-8 | 2.2242429 | 3.6156311 | 30.730663 | 60.619629 | 91.270035 | 124.79771 | 153.81075 | 175.84995 | 193.15295 |
| 11 | OF'-9 | 1.6404347 | 3.3189168 | 30.99719 | 61.037746 | 91.789551 | 125.30642 | 153.94824 | 175.59956 | 192.14386 |
| 11 | OF'-10 | 1.4237099 | 3.2866945 | 32.12352 | 63.238659 | 94.860237 | 129.23755 | 158.16808 | 179.38643 | 194.90857 |
| 11 | OF'-11 | 2.397542 | 3.8665814 | 32.897778 | 64.531105 | 96.515427 | 131.0509 | 159.2608 | 179.3512 | 192.75366 |
| 11 | OF'-12 | 2.4715025 | 4.2185369 | 33.691605 | 65.918953 | 98.410782 | 133.1114 | 160.92847 | 179.70966 | 191.59572 |
| 11 | B'-4 | 1.4662659 | 2.1299615 | 16.203047 | 30.924208 | 45.693832 | 63.477547 | 80.43399 | 95.942009 | 107.94312 |
| 11 | B'-5 | 1.2064774 | 2.0011711 | 17.051779 | 32.641006 | 48.391129 | 67.457596 | 85.526672 | 101.9406 | 114.80267 |
| 11 | B'-6 | 1.2256459 | 2.0717025 | 17.592831 | 33.673195 | 49.929077 | 69.619606 | 88.05024 | 104.5849 | 117.3915 |
| 11 | B'-7 | 1.4161931 | 2.1928921 | 17.965054 | 34.354851 | 50.946182 | 71.10746 | 89.866119 | 106.57861 | 119.47668 |
| 11 | B'-8 | 1.7823036 | 2.4449484 | 18.280287 | 34.909111 | 51.707222 | 72.151016 | 90.959251 | 107.49371 | 120.13745 |
| 11 | TA'-5 | 1.2367706 | 1.5000373 | 5.9660988 | 9.7501526 | 12.967634 | 16.848133 | 21.204611 | 27.51144 | 30.465694 |
| 11 | TA'-6 | 1.0876245 | 1.3515158 | 6.3131814 | 10.412371 | 13.863582 | 18.149261 | 22.874851 | 29.470293 | 32.66489 |
| 11 | TA'-7 | 1.0813093 | 1.3350081 | 6.6949053 | 11.121822 | 14.863474 | 19.56665 | 24.665129 | 31.624109 | 34.993507 |
| 11 | TA'-8 | 1.1405344 | 1.3652668 | 7.0721564 | 11.797039 | 15.780645 | 20.885338 | 26.323065 | 33.601254 | 37.170734 |
| 11 | TA'-9 | 1.3018334 | 1.4759274 | 7.1549225 | 11.865884 | 15.790303 | 20.918976 | 26.379343 | 33.665173 | 37.114868 |
| 11 | TA'-10 | 1.3499341 | 1.4749268 | 7.0313039 | 11.564749 | 15.228065 | 20.208887 | 25.505999 | 32.762581 | 35.930038 |
| 11 | TA'-11 | 1.472351 | 1.5336024 | 6.9521213 | 11.338892 | 14.793365 | 19.652906 | 24.795128 | 31.967091 | 34.915386 |
| 11 | TA'-12 | 1.41623 | 1.4947628 | 6.898313 | 11.146336 | 14.398341 | 19.195192 | 24.196871 | 31.253004 | 33.913136 |
| 11 | TH'-5 | 1.7701024 | 2.1112025 | 2.8348675 | 3.7632499 | 5.3230972 | 8.5925283 | 11.104734 | 13.089466 | 14.353566 |
| 11 | TH'-6 | 1.6568248 | 1.9231578 | 3.0041912 | 3.764611 | 5.2283597 | 8.3598957 | 10.875424 | 13.101728 | 14.338527 |
| 11 | TH'-7 | 1.3262868 | 1.4629997 | 3.142838 | 3.9587007 | 5.1604977 | 8.0653887 | 10.501993 | 13.359642 | 14.301357 |
| 11 | TH'-8 | 1.3196255 | 1.3578929 | 3.5567324 | 4.5675716 | 5.5535102 | 8.2123775 | 10.661415 | 14.188174 | 14.818126 |
| 11 | TH'-9 | 1.3990126 | 1.3868182 | 3.7723081 | 4.881403 | 5.888453 | 8.635705 | 11.243873 | 15.154727 | 15.76094 |
| 11 | TH'-10 | 1.6007172 | 1.6206691 | 3.8067129 | 4.876462 | 5.9853888 | 8.9898872 | 11.738896 | 15.632103 | 16.335979 |
| 11 | TH'-11 | 1.4951801 | 1.5267516 | 3.6762915 | 4.7029476 | 6.1270614 | 9.4349661 | 12.415788 | 16.33625 | 17.274412 |
| 11 | TH'-12 | 1.3060589 | 1.3655468 | 3.5584753 | 4.559257 | 6.3880324 | 10.001209 | 13.16493 | 16.991762 | 18.292973 |
| 11 | P'-1 | 1.8806478 | 1.9101256 | 3.8575568 | 9.8152542 | 18.09679 | 26.092945 | 32.645664 | 35.466602 | 42.46859 |
| 11 | P'-2 | 1.7590896 | 1.7747298 | 3.7939913 | 9.8062344 | 18.332064 | 26.290512 | 32.877213 | 35.760548 | 42.814751 |
| 11 | P'-3 | 1.8416157 | 2.0269463 | 4.0031338 | 10.109569 | 18.635468 | 26.431225 | 32.786068 | 35.429344 | 41.746525 |
| 11 | P'-4 | 1.9991337 | 2.162586 | 4.1846657 | 10.385617 | 19.275095 | 27.349739 | 34.191257 | 37.525852 | 44.57793 |
| 11 | P'-5 | 2.4843011 | 2.6500366 | 4.6953821 | 10.970432 | 20.079769 | 28.278257 | 35.22543 | 38.400177 | 45.800037 |
| 11 | P'-6 | 1.9442209 | 2.0753312 | 4.582881 | 11.80483 | 21.593927 | 29.861912 | 36.802841 | 40.001747 | 47.130543 |
| 11 | P'-7 | 1.5944868 | 1.528447 | 6.1995835 | 16.103941 | 27.978607 | 35.197624 | 42.2015 | 54.616291 | 73.85331 |
| 11 | P'-8 | 2.9020245 | 1.8161212 | 12.89409 | 33.450695 | 57.572498 | 67.585968 | 78.235222 | 93.305618 | 121.71738 |

“Ground”, “Common”, and “Broken” meant Ground reference contact, Common reference contact, and Broken contact, respectively.

A:L-ORB→ACC, left orbital frontal cortex→anterior cingulate cortex;

FF:L-lFFG→mFFG, left lateral fusiform gyrus→medial fusiform gyrus;

IP:L-lPoCG→mPoCG, left-lateral postcentral gyrus→medial postcentral gyrus;

IP':R-lPoCG→mPoCG, right-lateral postcentral gyrus→medial postcentral gyrus;

O:L-lTPO→mTPO, left-lateral temporal occipital junction→medial temporal occipital junction;

OF:L-lORB→mORB, left-lateral orbital frontal cortex→medial orbital frontal cortex;

OF':R-lORB→mORB, right-lateral orbital frontal cortex→medial orbital frontal cortex;

OH:L-MOG→PreHIP, left-middle occipital gyrus→pre-hippocampus;

OH':R-MOG→PreHIP, right-middle occipital gyrus→pre-hippocampus;

P:L-lMTG→mMTG, left-lateral middle temporal gyrus→medial middle temporal gyrus;

P':R-lMTG→mMTG, right-lateral middle temporal gyrus→medial middle temporal gyrus;

P1:L-lPreMTG→mPreMTG, left lateral pre-middle temporal gyrus→medial pre-middle temporal gyrus;

P2:L-lPoMTG→mPoMTG, left lateral post-middle temporal gyrus→medial post-middle temporal gyrus;

P1':R-lPreMTG→mPreMTG, right-lateral pre-middle temporal gyrus→medial pre-middle temporal gyrus;

P2':R-lPoMTG→mPoMTG, right-lateral post-middle temporal gyrus→medial post-middle temporal gyrus;

PO:L-lSMG→mSMG, left-lateral supramarginal gyrus→medial supramarginal gyrus;

PO':R-lSMG→mSMG; right-lateral supramarginal gyrus→medial supramarginal gyrus;

PO1:L-lPreSMG→mPreSMG, left-lateral pre-supramarginal gyrus→medial pre-supramarginal gyrus;

PO1':R-lPreSMG→mPreSMG, right-lateral pre-supramarginal gyrus→medial pre-supramarginal gyrus;

PO2:L-lPoSMG→mPoSMG, left-lateral post-supramarginal gyrus→medial post-supramarginal gyrus;

PO2':R-lPoSMG→mPoSMG, right-lateral post-supramarginal gyrus→medial post-supramarginal gyrus;

SM:L-lSMA→mSMA, left-lateral supplementary motor area→medial supplementary motor area;

TO:L-lMOG→mMOG, left-lateral middle occipital gyrus→medial middle occipital gyrus;

V:L-lSPG→mSPG, left-lateral superior parietal gyrus→medial superior parietal gyrus;

V':R-lSPG→mSPG, right-lateral superior parietal gyrus→medial superior parietal gyrus;

W:L-lSTG→mSTG, left-lateral superior temporal gyrus→medial superior temporal gyrus.

**Table S6.** The linear regression equations on the between the local field potentials in hippocampus, insula, and amygdala with the extracranial currents in an increase

|  | **Sub.No.** | **Brain regions** | | **Linear regression equations** | ***p* value** |
| --- | --- | --- | --- | --- | --- |
| **Hippocampus** | Sub.1 | L-Hip | | Y = 13.72*X + 0.7215 | <0.001 |
|  | Sub.1 | R-Hip | | Y = 12.09*X + 0.3560 | <0.001 |
|  | Sub.2 | R-Hip | | Y = 464.6*X - 1248 | <0.001 |
|  | Sub.3 | L-Hip | | Y = 0.08857*X + 2.017 | <0.001 |
|  | Sub.4 | L-Hip | | Y = 16.16*X - 23.07 | <0.001 |
|  | Sub.4 | R-Hip | | Y = 15.96*X - 22.86 | <0.001 |
|  | Sub.6 | L-Hip | | Y = 4.057*X - 7.830 | <0.001 |
|  | Sub.7 | L-Hip | | Y = 113.8*X - 350.9 | 0.003 |
|  | Sub.7 | R-Hip | | Y = 90.18*X - 281.5 | 0.005 |
|  | Sub.8 | L-Hip | | Y = 2.277*X - 1.955 | <0.001 |
|  | Sub.9 | R-Hip | | Y = 2.494*X - 0.08160 | <0.001 |
|  | Sub.10 | L-Hip | | Y = 4.379*X - 10.20 | 0.001 |
|  | Sub.10 | R-Hip | | Y = 4.440*X - 9.062 | 0.001 |
|  | Sub.11 | L-Hip | | Y = 1.702*X + 0.3099 | <0.001 |
|  | Sub.11 | R-Hip | | Y = 0.9538*X + 1.740 | <0.001 |
| **Insula** | Sub.1 | L-Ins | | Y = 8.515*X - 1.754 | <0.001 |
|  | Sub.1 | R-Ins | | Y = 2.028*X + 3.445 | 0.001 |
|  | Sub.2 | R-Ins | | Y = 204.3*X - 616.2 | 0.002 |
|  | Sub.3 | L-Ins | | Y = 5.558*X - 5.096 | <0.001 |
|  | Sub.5 | L-Ins | | Y = 50.99*X - 154.4 | 0.003 |
|  | Sub.6 | L-Ins | | Y = 72.91*X - 218.7 | 0.002 |
|  | Sub.7 | L-Ins | | Y = 12.65*X - 36.01 | 0.007 |
|  | Sub.10 | L-Ins | | Y = 18.18*X - 57.43 | 0.053 |
|  | Sub.10 | R-Ins | | Y = 124.9*X - 402.5 | 0.006 |
|  | Sub.11 | R-Ins | | Y = 6.962*X - 3.576 | <0.001 |
| **Amygdala** | Sub.1 | | L-Amy | Y = 11.98*X - 1.247 | <0.001 |
|  | Sub.2 | | R-Amy | Y = 359.9*X - 1009 | <0.001 |
|  | Sub.3 | | L-Amy | Y = 1.913*X - 1.304 | <0.001 |
|  | Sub.5 | | L-Amy | Y = 0.1190*X + 1.449 | <0.001 |
|  | Sub.6 | | L-Amy | Y = 2.338*X - 5.424 | <0.001 |
|  | Sub.7 | | R-Amy | Y = 39.55*X - 123.3 | 0.007 |
|  | Sub.8 | | L-Amy | Y = 0.4493*X + 0.8573 | <0.001 |
|  | Sub.9 | | R-Amy | Y = 4.267*X - 2.541 | <0.001 |
|  | Sub.11 | | L-Amy | Y = 0.04798*X + 1.789 | 0.005 |
|  | Sub.11 | | R-Amy | Y = 1.485*X + 0.4999 | <0.001 |

Amy, amygdala; Hip, hippocampus; Ins, insula; L, left; No., number; R, right; Sub, subject.

**Table S7.** Correlations of the local field potentials of all contacts with the alternating currents in all 11 subjects (*p* value).

The correlations between the changes of local field potential in all contacts and extracranial alternating currents were examined by linear regression analysis. The results showed that most contacts had linear correlations between the changes of local field potential and the increase of transcranial alternating currents (*p* < 0.05), except for 8 ground contacts, 8 common contacts, 3 broken contacts, 6 contacts close to the cerebrospinal fluid (the 16^th^ contact of the OF/L-lORB→mORB electrode in Subject 1, the 8th contact of the OF'/R-lORB→mORB electrode in Subject 2, the 8^th^ and 10^th^ contacts of OF/L-lORB→mORB electrode in Subject 7, the 1^st^ contact of the B/L-Broca’s area→INS electrode in Subject 10, and the 1^st^ contact of the TA/L-MTG→AMY electrode in Subject 11), 1 contact in the sulcus (the 9^th^ contact of the OF/L-lORB→mORB electrode in Subject 7), and 1 contact close to ground contact (the 5^th^ contact was too close to the 6^th^ contact, as ground contact, of TH/ L-MTG→HIP electrode in Subject 3).

**Subject No.1**

| Contact | OF | A | B | TA | SM | TH | PO | IP | P | OF' | B' | TH' | IP' |
| --- | --- | --- | --- | --- | --- | --- | --- | --- | --- | --- | --- | --- | --- |
| 1 | <0.0001 | <0.0001 | <0.0001 | <0.0001 | <0.0001 | <0.0001 | <0.0001 | <0.0001 | <0.0001 | <0.0001 | 0.0011 | <0.0001 | <0.0001 |
| 2 | <0.0001 | <0.0001 | <0.0001 | <0.0001 | <0.0001 | <0.0001 | <0.0001 | <0.0001 | <0.0001 | <0.0001 | 0.0013 | <0.0001 | <0.0001 |
| 3 | <0.0001 | <0.0001 | <0.0001 | <0.0001 | <0.0001 | <0.0001 | <0.0001 | <0.0001 | <0.0001 | <0.0001 | 0.0015 | <0.0001 | <0.0001 |
| 4 | <0.0001 | <0.0001 | <0.0001 | <0.0001 | <0.0001 | <0.0001 | <0.0001 | <0.0001 | <0.0001 | <0.0001 | 0.0018 | <0.0001 | <0.0001 |
| 5 | <0.0001 | <0.0001 | <0.0001 | <0.0001 | <0.0001 | <0.0001 | <0.0001 | <0.0001 | <0.0001 | <0.0001 | 0.0025 | <0.0001 | <0.0001 |
| 6 | <0.0001 | <0.0001 | <0.0001 | <0.0001 | <0.0001 | <0.0001 | <0.0001 | <0.0001 | <0.0001 | <0.0001 | 0.0043 | <0.0001 | <0.0001 |
| 7 | <0.0001 | <0.0001 | <0.0001 | <0.0001 | <0.0001 | <0.0001 | <0.0001 | <0.0001 | <0.0001 | 0.0003 | 0.0092 | <0.0001 | <0.0001 |
| 8 | 0.0002 | <0.0001 | <0.0001 | <0.0001 | <0.0001 | <0.0001 | <0.0001 | <0.0001 | <0.0001 | 0.0004 | 0.0167 | <0.0001 | <0.0001 |
| 9 | 0.0002 | 0.0001 | <0.0001 | <0.0001 |  | <0.0001 |  |  |  | 0.0006 |  | <0.0001 | <0.0001 |
| 10 | <0.0001 | <0.0001 | <0.0001 | <0.0001 |  | <0.0001 |  |  |  | 0.0014 |  | <0.0001 | <0.0001 |
| 11 | <0.0001 | <0.0001 |  | <0.0001 |  | <0.0001 |  |  |  | 0.0071 |  | <0.0001 |  |
| 12 | <0.0001 | <0.0001 |  | <0.0001 |  | <0.0001 |  |  |  | 0.0002 |  | <0.0001 |  |
| 13 | <0.0001 |  |  |  |  |  |  |  |  | 0.0030 |  |  |  |
| 14 | <0.0001 |  |  |  |  |  |  |  |  | 0.0027 |  |  |  |
| 15 | 0.0005 |  |  |  |  |  |  |  |  | 0.0033 |  |  |  |
| 16 | 0.0780 |  |  |  |  |  |  |  |  | 0.0154 |  |  |  |

**Subject No.2**

| Contact | OF' | B' | TA' | TH1' | TH' | P' | OH' |
| --- | --- | --- | --- | --- | --- | --- | --- |
| 1 | 0.0026 | 0.0017 | 0.0008 | 0.0006 | 0.0005 | 0.0004 | 0.0006 |
| 2 | 0.0020 | 0.0028 | 0.0010 | 0.0006 | 0.0003 | 0.0003 | 0.0004 |
| 3 | 0.0036 | 0.0024 | 0.0005 | 0.0005 | 0.0005 | 0.0004 | 0.0008 |
| 4 | 0.0011 | 0.0088 | 0.0021 | 0.0008 | 0.0004 | 0.0002 | 0.0008 |
| 5 | 0.0027 | 0.0053 | 0.0012 | 0.0007 | 0.0004 | 0.0003 | 0.0002 |
| 6 | 0.0028 | 0.0060 | 0.0011 | 0.0005 | 0.0005 | 0.0004 | 0.0002 |
| 7 | 0.0024 | 0.0066 | 0.0006 | 0.0009 | 0.0005 | 0.0003 | 0.0001 |
| 8 | 0.0799 | 0.0072 | 0.0011 | 0.0004 | 0.0009 | 0.0003 | 0.0003 |
| 9 | <0.0001 |  | 0.0015 | 0.0006 | 0.0006 | 0.0004 | 0.0003 |
| 10 | 0.0002 |  | 0.0008 | 0.0004 | 0.0004 | 0.0002 | 0.0003 |
| 11 | 0.0153 |  | 0.0007 | 0.0005 | 0.0003 | 0.0004 | 0.0003 |
| 12 | 0.0032 |  | 0.0006 | 0.0006 | 0.0005 | 0.0002 | 0.0002 |

**Subject No.3**

| Contact | OF | A | B | TA | TH | P | PO |
| --- | --- | --- | --- | --- | --- | --- | --- |
| 1 | <0.0001 | <0.0001 | <0.0001 | <0.0001 | <0.0001 | <0.0001 | <0.0001 |
| 2 | <0.0001 | <0.0001 | <0.0001 | <0.0001 | 0.0139 | 0.0007 | <0.0001 |
| 3 | <0.0001 | <0.0001 | <0.0001 | <0.0001 | <0.0001 | 0.0009 | <0.0001 |
| 4 | <0.0001 | <0.0001 | <0.0001 | <0.0001 | <0.0001 | 0.0015 | <0.0001 |
| 5 | <0.0001 | <0.0001 | <0.0001 | <0.0001 | 0.0617 | 0.0089 | <0.0001 |
| 6 | <0.0001 | <0.0001 | <0.0001 | <0.0001 | Ground | 0.0197 | <0.0001 |
| 7 | <0.0001 | <0.0001 | <0.0001 | <0.0001 | Common | 0.0465 | <0.0001 |
| 8 | <0.0001 | <0.0001 | <0.0001 | <0.0001 | <0.0001 | 0.0005 | <0.0001 |
| 9 | <0.0001 | <0.0001 |  | <0.0001 | <0.0001 |  |  |
| 10 | <0.0001 | <0.0001 |  | <0.0001 | <0.0001 |  |  |
| 11 | <0.0001 | <0.0001 |  | <0.0001 | <0.0001 |  |  |
| 12 | <0.0001 | <0.0001 |  | <0.0001 | <0.0001 |  |  |
| 13 | <0.0001 |  |  |  |  |  |  |
| 14 | <0.0001 |  |  |  |  |  |  |
| 15 | <0.0001 |  |  |  |  |  |  |
| 16 | <0.0001 |  |  |  |  |  |  |

**Subject No.4**

| Contact | A | TH | PO1 | PO2 | P | TH' | PO1' | PO2' | P1' | P2' |
| --- | --- | --- | --- | --- | --- | --- | --- | --- | --- | --- |
| 1 | <0.0001 | <0.0001 | <0.0001 | <0.0001 | <0.0001 | <0.0001 | <0.0001 | <0.0001 | <0.0001 | <0.0001 |
| 2 | 0.0002 | <0.0001 | <0.0001 | <0.0001 | <0.0001 | <0.0001 | <0.0001 | <0.0001 | <0.0001 | <0.0001 |
| 3 | <0.0001 | <0.0001 | <0.0001 | <0.0001 | <0.0001 | <0.0001 | <0.0001 | <0.0001 | <0.0001 | <0.0001 |
| 4 | <0.0001 | <0.0001 | <0.0001 | <0.0001 | <0.0001 | Broken | <0.0001 | <0.0001 | <0.0001 | 0.0006 |
| 5 | <0.0001 | <0.0001 | <0.0001 | <0.0001 | <0.0001 | <0.0001 | <0.0001 | <0.0001 | <0.0001 | <0.0001 |
| 6 | <0.0001 | <0.0001 | <0.0001 | <0.0001 | <0.0001 | <0.0001 | <0.0001 | <0.0001 | <0.0001 | <0.0001 |
| 7 | <0.0001 | <0.0001 | <0.0001 | <0.0001 | <0.0001 | <0.0001 | <0.0001 | <0.0001 | <0.0001 | <0.0001 |
| 8 | <0.0001 | <0.0001 | <0.0001 | <0.0001 | <0.0001 | <0.0001 | <0.0001 | <0.0001 | <0.0001 | <0.0001 |
| 9 | <0.0001 |  |  | <0.0001 |  |  |  |  |  |  |
| 10 | <0.0001 |  |  | <0.0001 |  |  |  |  |  |  |

**Subject No.5**

| Contact | A | B | TA | C | P1 | P2 | FF |
| --- | --- | --- | --- | --- | --- | --- | --- |
| 1 | 0.0023 | 0.0035 | 0.0023 | <0.0001 | <0.0001 | <0.0001 | <0.0001 |
| 2 | 0.0016 | 0.0034 | 0.0060 | <0.0001 | <0.0001 | <0.0001 | <0.0001 |
| 3 | 0.0019 | 0.0030 | 0.0045 | <0.0001 | <0.0001 | <0.0001 | 0.0001 |
| 4 | 0.0019 | 0.0027 | 0.0022 | <0.0001 | <0.0001 | <0.0001 | 0.0012 |
| 5 | 0.0014 | 0.0021 | 0.0016 | <0.0001 | <0.0001 | <0.0001 | 0.0019 |
| 6 | 0.0013 | 0.0022 | 0.0036 | <0.0001 | <0.0001 |  |  |
| 7 | 0.0010 | 0.0016 | Ground | 0.0002 | <0.0001 |  |  |
| 8 | 0.0008 | 0.0019 | Common | 0.0002 | 0.0030 |  |  |
| 9 | 0.0010 | 0.0015 | 0.0007 | 0.0009 |  |  |  |
| 10 | 0.0007 | 0.0012 | 0.0049 | 0.0004 |  |  |  |
| 11 | 0.0006 |  | 0.0001 |  |  |  |  |
| 12 | 0.0006 |  | <0.0001 |  |  |  |  |
| 13 | 0.0003 |  | <0.0001 |  |  |  |  |
| 14 | 0.0003 |  | <0.0001 |  |  |  |  |
| 15 | <0.0001 |  | <0.0001 |  |  |  |  |

**Subject No.6**

| Contact | OF | B | TA | TH | PO | W | FF | P | O |
| --- | --- | --- | --- | --- | --- | --- | --- | --- | --- |
| 1 | 0.0017 | 0.0032 | 0.0012 | 0.0006 | <0.0001 | 0.0002 | 0.0003 | 0.0002 | 0.0003 |
| 2 | 0.0014 | 0.0021 | 0.0008 | 0.0005 | 0.0004 | 0.0001 | 0.0003 | 0.0002 | 0.0006 |
| 3 | 0.0014 | 0.0025 | 0.0003 | 0.0004 | 0.0115 | 0.0001 | 0.0003 | 0.0002 | 0.0003 |
| 4 | 0.0014 | 0.0024 | 0.0003 | 0.0004 | 0.0113 | 0.0004 | 0.0003 | 0.0002 | 0.0003 |
| 5 | 0.0012 | 0.0019 | Ground | 0.0004 | 0.0023 | <0.000 | 0.0002 | 0.0002 | 0.0003 |
| 6 | 0.0012 | 0.0016 | Common | 0.0003 | Broken | 0.0021 | 0.0003 | 0.0003 | 0.0003 |
| 7 | 0.0012 | 0.0014 | <0.0001 | 0.0003 | 0.0009 | <0.000 | 0.0003 | 0.0003 | 0.0004 |
| 8 | 0.0012 | 0.0016 | <0.0001 | 0.0003 | 0.0016 | <0.0001 | 0.0002 | 0.0003 | 0.0005 |
| 9 | 0.0011 | 0.0019 | <0.0001 | 0.0003 | 0.0028 | <0.0001 | 0.0003 | 0.0002 | 0.0010 |
| 10 | 0.0010 | 0.0018 | 0.0032 | 0.0003 | 0.0035 | 0.0078 | 0.0003 | 0.0003 | 0.0004 |
| 11 | 0.0009 |  | 0.0006 | 0.0004 |  | 0.0037 |  | 0.0003 |  |
| 12 | 0.0008 |  | 0.0010 | 0.0003 |  | 0.0007 |  | 0.0003 |  |

**Subject No.7**

| Contact | OF | A | B | TA | TH | IP | P | TA' | TH' |
| --- | --- | --- | --- | --- | --- | --- | --- | --- | --- |
| 1 | 0.0036 | 0.0045 | 0.0067 | 0.0087 | 0.0036 | 0.0077 | 0.0022 | 0.0083 | 0.0048 |
| 2 | 0.0094 | 0.0049 | 0.0066 | 0.0069 | 0.0032 | 0.0089 | 0.0026 | 0.0076 | 0.0052 |
| 3 | 0.0183 | 0.0053 | 0.0053 | 0.0096 | 0.0037 | 0.0078 | 0.0028 | 0.0059 | 0.0044 |
| 4 | 0.0292 | 0.0072 | 0.0072 | 0.0043 | 0.0033 | 0.0074 | 0.0023 | 0.0073 | 0.0045 |
| 5 | 0.0033 | 0.0210 | 0.0051 | 0.0075 | 0.0032 | 0.0086 | 0.0027 | 0.0071 | 0.0041 |
| 6 | Ground | 0.0020 | 0.0073 | 0.0080 | 0.0037 | 0.0077 |  | 0.0076 | 0.0043 |
| 7 | Common | 0.0178 | 0.0057 | 0.0094 | 0.0035 | 0.0073 |  | 0.0091 | 0.0044 |
| 8 | 0.9942 | 0.0008 | 0.0046 | 0.0081 | 0.0042 | 0.0080 |  | 0.0100 | 0.0046 |
| 9 | 0.5048 | 0.0034 |  |  | 0.0040 |  |  |  | 0.0045 |
| 10 | 0.8856 | 0.0055 |  |  | 0.0032 |  |  |  | 0.0062 |

**Subject No.8**

| Contact | OF | TA1 | TA2 | TH1 | TH | OH |
| --- | --- | --- | --- | --- | --- | --- |
| 1 | 0.0064 | <0.0001 | <0.0001 | <0.0001 | <0.0001 | <0.0001 |
| 2 | 0.0069 | 0.0001 | <0.0001 | <0.0001 | <0.0001 | <0.0001 |
| 3 | 0.0112 | 0.0001 | <0.0001 | <0.0001 | <0.0001 | <0.0001 |
| 4 | 0.0101 | 0.0146 | <0.0001 | <0.0001 | <0.0001 | <0.0001 |
| 5 | 0.0059 | 0.0090 | <0.0001 | <0.0001 | <0.0001 | <0.0001 |
| 6 | 0.0052 | 0.0222 | <0.0001 | <0.0001 | <0.0001 | <0.0001 |
| 7 | 0.0025 | Ground | <0.0001 | <0.0001 | <0.0001 | <0.0001 |
| 8 | 0.0034 | Common | <0.0001 | <0.0001 | <0.0001 | <0.0001 |
| 9 | 0.0049 | 0.0020 | <0.0001 | <0.0001 | <0.0001 | <0.0001 |
| 10 | 0.0019 | 0.0051 | <0.0001 | <0.0001 | <0.0001 | <0.0001 |
| 11 |  | 0.0028 | 0.0002 | <0.0001 | <0.0001 | <0.0001 |
| 12 |  | 0.0020 | 0.0001 | <0.0001 | <0.0001 | <0.0001 |
| 13 |  |  |  |  |  | <0.0001 |
| 14 |  |  |  |  |  | <0.0001 |
| 15 |  |  |  |  |  | <0.0001 |

**Subject No.9**

| Contact | OF' | TA1' | TA2' | TH1' | TH' | OH' |
| --- | --- | --- | --- | --- | --- | --- |
| 1 | <0.0001 | <0.0001 | <0.0001 | <0.0001 | <0.0001 | <0.0001 |
| 2 | <0.0001 | <0.0001 | <0.0001 | <0.0001 | <0.0001 | <0.0001 |
| 3 | <0.0001 | <0.0001 | <0.0001 | <0.0001 | <0.0001 | <0.0001 |
| 4 | Broken | <0.0001 | <0.0001 | <0.0001 | <0.0001 | <0.0001 |
| 5 | <0.0001 | <0.0001 | <0.0001 | <0.0001 | <0.0001 | <0.0001 |
| 6 | <0.0001 | <0.0001 | <0.0001 | <0.0001 | <0.0001 | <0.0001 |
| 7 | <0.0001 | <0.0001 | <0.0001 | <0.0001 | <0.0001 | <0.0001 |
| 8 | <0.0001 | <0.0001 | <0.0001 | <0.0001 | <0.0001 | <0.0001 |
| 9 | <0.0001 | <0.0001 | <0.0001 | <0.0001 | <0.0001 | <0.0001 |
| 10 | <0.0001 | <0.0001 | <0.0001 | <0.0001 | <0.0001 | <0.0001 |
| 11 |  | <0.0001 | <0.0001 | <0.0001 | <0.0001 | <0.0001 |
| 12 |  | <0.0001 | <0.0001 | <0.0001 | <0.0001 | <0.0001 |
| 13 |  |  |  |  |  | <0.0001 |
| 14 |  |  |  |  |  | Ground |
| 15 |  |  |  |  |  | Common |

**Subject No.10**

| Contact | B | TH | P | PO | TO | V | B' | TH' | PO' | V' |
| --- | --- | --- | --- | --- | --- | --- | --- | --- | --- | --- |
| 1 | 0.0690 | 0.0013 | 0.0089 | 0.0063 | 0.0136 | 0.0018 | 0.0121 | 0.0019 | 0.0052 | 0.0097 |
| 2 | 0.0352 | 0.0010 | 0.0076 | 0.0055 | 0.0128 | 0.0014 | 0.0038 | 0.0013 | 0.0067 | 0.0112 |
| 3 | 0.0246 | 0.0010 | 0.0100 | 0.0050 | 0.0118 | 0.0015 | 0.0053 | 0.0011 | 0.0046 | 0.0104 |
| 4 | 0.0245 | 0.0011 | 0.0067 | 0.0063 | 0.0109 | 0.0017 | 0.0070 | 0.0011 | 0.0038 | 0.0120 |
| 5 | 0.0327 | 0.0012 | 0.0075 | 0.0061 | 0.0094 | 0.0018 | 0.0027 | 0.0011 | 0.0067 | 0.0135 |
| 6 | 0.0381 | 0.0008 | 0.0090 | 0.0103 | 0.0109 | 0.0021 | 0.0026 | 0.0014 | 0.0047 | 0.0086 |
| 7 | 0.0177 | Ground | 0.0082 | 0.0058 | 0.0114 | 0.0025 | 0.0020 | 0.0011 |  | 0.0058 |
| 8 | 0.0400 | Common | 0.0071 | 0.0081 | 0.0095 | 0.0029 | 0.0335 | 0.0010 |  | 0.0061 |
| 9 |  | 0.0006 |  |  | 0.0105 | 0.0038 |  | 0.0008 |  | 0.0059 |
| 10 |  | 0.0007 |  |  | 0.0093 | 0.0061 |  | 0.0206 |  | 0.0057 |
| 11 |  | 0.0011 |  |  |  |  |  | 0.0006 |  | 0.0099 |
| 12 |  | 0.0014 |  |  |  |  |  | 0.0007 |  | 0.0050 |

**Subject No.11**

| Contact | OF | TA | TH | OF' | B' | TA' | TH' | P' |
| --- | --- | --- | --- | --- | --- | --- | --- | --- |
| 1 | <0.0001 | 0.1245 | <0.0001 | <0.0001 | <0.0001 | <0.0001 | <0.0001 | <0.0001 |
| 2 | <0.0001 | 0.0055 | <0.0001 | <0.0001 | <0.0001 | <0.0001 | <0.0001 | <0.0001 |
| 3 | <0.0001 | 0.0080 | <0.0001 | <0.0001 | <0.0001 | <0.0001 | <0.0001 | <0.0001 |
| 4 | <0.0001 | 0.0014 | <0.0001 | <0.0001 | <0.0001 | <0.0001 | <0.0001 | <0.0001 |
| 5 | <0.0001 | 0.0174 | <0.0001 | <0.0001 | <0.0001 | <0.0001 | <0.0001 | <0.0001 |
| 6 | <0.0001 | 0.0196 | <0.0001 | <0.0001 | <0.0001 | <0.0001 | <0.0001 | <0.0001 |
| 7 | <0.0001 | Ground | <0.0001 | <0.0001 | <0.0001 | <0.0001 | <0.0001 | <0.0001 |
| 8 | <0.0001 | Common | <0.0001 | <0.0001 | <0.0001 | <0.0001 | <0.0001 | <0.0001 |
| 9 | <0.0001 | <0.0001 | <0.0001 | <0.0001 |  | <0.0001 | <0.0001 |  |
| 10 | <0.0001 | <0.0001 | <0.0001 | <0.0001 |  | <0.0001 | <0.0001 |  |
| 11 | <0.0001 | <0.0001 | <0.0001 | <0.0001 |  | <0.0001 | <0.0001 |  |
| 12 | <0.0001 | <0.0001 | <0.0001 | <0.0001 |  | <0.0001 | <0.0001 |  |

*p* values of contacts were obtained by linear regression analysis.

“Ground”, “Common”, and “Broken” meant Ground reference contact, Common reference contact, and Broken contact, respectively.

The highlighted blue color signified that *p* values were ≥0.05.

A:L-ORB→ACC, left orbital frontal cortex→anterior cingulate cortex;

B:L-Broca’s area→INS, left-Broca’s area→insula;

B':R-Broca’s area→INS, right-Broca’s area→insula;

C:L-PreCG→INS; left precentral gyrus→Insula;

FF:L-lFFG→mFFG, left lateral fusiform gyrus→medial fusiform gyrus;

IP:L-lPoCG→mPoCG, left-lateral postcentral gyrus→medial postcentral gyrus;

IP':R-lPoCG→mPoCG, right-lateral postcentral gyrus→medial postcentral gyrus;

O:L-lTPO→mTPO, left-lateral temporal occipital junction→medial temporal occipital junction;

OF:L-lORB→mORB, left-lateral orbital frontal cortex→medial orbital frontal cortex;

OF':R-lORB→mORB, right-lateral orbital frontal cortex→medial orbital frontal cortex;

OH:L-MOG→PreHIP, left-middle occipital gyrus→pre-hippocampus;

OH':R-MOG→PreHIP, right-middle occipital gyrus→pre-hippocampus;

P:L-lMTG→mMTG, left-lateral middle temporal gyrus→medial middle temporal gyrus;

P':R-lMTG→mMTG, right-lateral middle temporal gyrus→medial middle temporal gyrus;

P1:L-lPreMTG→mPreMTG, left lateral pre-middle temporal gyrus→medial pre-middle temporal gyrus;

P2:L-lPoMTG→mPoMTG, left lateral post-middle temporal gyrus→medial post-middle temporal gyrus;

P1':R-lPreMTG→mPreMTG, right-lateral pre-middle temporal gyrus→medial pre-middle temporal gyrus;

P2':R-lPoMTG→mPoMTG, right-lateral post-middle temporal gyrus→medial post-middle temporal gyrus;

PO:L-lSMG→mSMG, left-lateral supramarginal gyrus→medial supramarginal gyrus;

PO':R-lSMG→mSMG; right-lateral supramarginal gyrus→medial supramarginal gyrus;

PO1:L-lPreSMG→mPreSMG, left-lateral pre-supramarginal gyrus→medial pre-supramarginal gyrus;

PO1':R-lPreSMG→mPreSMG, right-lateral pre-supramarginal gyrus→medial pre-supramarginal gyrus;

PO2:L-lPoSMG→mPoSMG, left-lateral post-supramarginal gyrus→medial post-supramarginal gyrus;

PO2':R-lPoSMG→mPoSMG, right-lateral post-supramarginal gyrus→medial post-supramarginal gyrus;

SM:L-lSMA→mSMA, left-lateral supplementary motor area→medial supplementary motor area;

TA:L-MTG→AMY, left-middle temporal gyrus→amygdala;

TA':R-MTG→AMY, right-middle temporal gyrus→amygdala;

TA1:L-MTG→PreAMY, left-middle temporal gyrus→pre-amygdala;

TA1':R-MTG→PreAMY, right-middle temporal gyrus→pre-amygdala;

TA2:L-MTG→PoAMY, left-middle temporal gyrus→post-amygdala;

TA2':R-MTG→PoAMY, right-middle temporal gyrus→post-amygdala;

TH:L-MTG→HIP, left-middle temporal gyrus→hippocampus;

TH':R-MTG→HIP, right-middle temporal gyrus→hippocampus;

TH1:L-MTG→PreHIP, left-middle temporal gyrus→pre-hippocampus;

TH1':R-MTG→PreHIP, right-middle temporal gyrus→pre-hippocampus;

TO:L-lMOG→mMOG, left-lateral middle occipital gyrus→medial middle occipital gyrus;

V:L-lSPG→mSPG, left-lateral superior parietal gyrus→medial superior parietal gyrus;

V':R-lSPG→mSPG, right-lateral superior parietal gyrus→medial superior parietal gyrus;

W:L-lSTG→mSTG, left-lateral superior temporal gyrus→medial superior temporal gyrus.
